# Estimating national-level measles case–fatality ratios in low-income and middle-income countries: an updated systematic review and modelling study

**DOI:** 10.1016/S2214-109X(23)00043-8

**Published:** 2023-03-14

**Authors:** Alyssa N Sbarra, Jonathan F Mosser, Mark Jit, Matthew Ferrari, Rebecca E Ramshaw, Patrick O'Connor, L Kendall Krause, Emma L B Rogowski, Allison Portnoy

**Affiliations:** a Department of Infectious Disease Epidemiology, London School of Hygiene & Tropical Medicine, London, UK; b Institute of Health Metrics and Evaluation, University of Washington, Seattle, WA, USA; c Department of Health Metrics Sciences, University of Washington, Seattle, WA, USA; d Department of Biology, The Pennsylvania State University, University Park, PA, USA; e Department of Immunization, Vaccines, and Biologicals, WHO, Geneva, Switzerland; f Global Development Division, Bill & Melinda Gates Foundation, Seattle, WA, USA; g Center for Health Decision Science, Harvard T H Chan School of Public Health, Boston, MA, USA

## Abstract

**Background:**

To understand the current measles mortality burden, and to mitigate the future burden, it is crucial to have robust estimates of measles case fatalities. Estimates of measles case–fatality ratios (CFRs) that are specific to age, location, and time are essential to capture variations in underlying population-level factors, such as vaccination coverage and measles incidence, which contribute to increases or decreases in CFRs. In this study, we updated estimates of measles CFRs by expanding upon previous systematic reviews and implementing a meta-regression model. Our objective was to use all information available to estimate measles CFRs in low-income and middle-income countries (LMICs) by country, age, and year.

**Methods:**

For this systematic review and meta-regression modelling study, we searched PubMed on Dec 31, 2020 for all available primary data published from Jan 1, 1980 to Dec 31, 2020, on measles cases and fatalities occurring up to Dec 31, 2019 in LMICs. We included studies that previous systematic reviews had included or which contained primary data on measles cases and deaths from hospital-based, community-based, or surveillance-based reports, including outbreak investigations. We excluded studies that were not in humans, or reported only data that were only non-primary, or on restricted populations (eg, people living with HIV), or on long-term measles mortality (eg, death from subacute sclerosing panencephalitis), and studies that did not include country-level data or relevant information on measles cases and deaths, or were for a high-income country. We extracted summary data on measles cases and measles deaths from studies that fitted our inclusion and exclusion criteria. Using these data and a suite of covariates related to measles CFRs, we implemented a Bayesian meta-regression model to produce estimates of measles CFRs from 1990 to 2019 by location and age group. This study was not registered with PROSPERO or otherwise.

**Findings:**

We identified 2705 records, of which 208 sources contained information on both measles cases and measles deaths in LMICS and were included in the review. Between 1990 and 2019, CFRs substantially decreased in both community-based and hospital-based settings, with consistent patterns across age groups. For people aged 0–34 years, we estimated a mean CFR for 2019 of 1·32% (95% uncertainty interval [UI] 1·28–1·36) among community-based settings and 5·35% (5·08–5·64) among hospital-based settings. We estimated the 2019 CFR in community-based settings to be 3·03% (UI 2·89–3·16) for those younger than 1 year, 1·63% (1·58–1·68) for age 1–4 years, 0·84% (0·80–0·87) for age 5–9 years, and 0·67% (0·64–0·70) for age 10–14 years.

**Interpretation:**

Although CFRs have declined between 1990 and 2019, there are still large heterogeneities across locations and ages. One limitation of this systematic review is that we were unable to assess measles CFR among particular populations, such as refugees and internally displaced people. Our updated methodological framework and estimates could be used to evaluate the effect of measles control and vaccination programmes on reducing the preventable measles mortality burden.

**Funding:**

Bill & Melinda Gates Foundation; Gavi, the Vaccine Alliance; and the US National Institutes of Health.

## Introduction

In 2019, more than 207 500 deaths were estimated to be attributable to measles.^1^ However, the exact figure cannot be measured directly because of an absence of reliable data on measles mortality from most high-burden settings. Instead, measles mortality is usually estimated by combining incidence and case–fatality ratio (CFR) estimates.^2^ Therefore, an accurate understanding of CFRs across different times and geographies is essential for the estimation of measles mortality burden. Additionally, a robust understanding of country-level CFRs can help to identify opportunities to strengthen health systems and to inform assessments of the effectiveness of vaccination programmes. Cohort-based and cross-sectional studies and outbreak investigations provide literature reports of CFRs but are often limited to specific settings and years.^3^

**Research in context**

**Evidence before this study**

We searched PubMed on Dec 6, 2022, for systematic reviews published from Jan 1, 1980, to Dec 6, 2022, using the search terms “measles” AND “case fatality”. We included studies if they were a systematic review of measles case–fatality ratios (CFRs). We excluded studies that were not systematic reviews or did not contain information about measles or CFRs. We identified two previous systematic reviews that have synthesised individual studies of measles CFRs. The first of these reviews, by Wolfson and colleagues, was published in 2009 and used 58 community-based studies in 29 countries to provide global estimates of measles CFR. Wolfson and colleagues published a descriptive analysis suggesting global estimates of CFR with a mean of 3·3%, a median of 3·9%, and range from 0 to 40·1%. For outbreak investigations, results suggested a median CFR of 5·2% (95% CI 2·6–11·6). These results were the first figures of measles CFRs beyond single country–year studies, reports, and investigations; however, this review only included community-based studies, did not produce estimates for other locations or years, and did not stratify by other underlying determinants of mortality, such as the income level of each country.

The second review, by Portnoy and colleagues, was published in 2019 and included data from 1980 to 2016 from low-income and middle-income countries; studies included reports from both community-based (n=85) and hospital-based (n=39) settings. Following the review, the authors used a log-linear prediction model with a select set of covariates, generally understood to be related to measles CFR (eg, previous vaccination history [with first dose of measles-containing vaccine coverage used as a proxy] and estimated measles attack rate) and indirectly associated with measles CFR (eg, mortality in children younger than 5 years [hereafter referred to as under-5 mortality], total fertility rate, proportion of population living in urban areas, and population density). The authors reported predicted CFR stratified by year, World Bank country-development status, under-5 mortality, care setting (community *vs* hospital), age (younger than 5 years *vs* 5 years or older), and calendar year from 1990 to 2030. Results predicted a mean CFR of 2·2% (95% CI 0·7–4·5) for years 1990–2015, with stratification for studies based in the community (CFR 1·5% [0·5–3·1]) and hospitals (CFR 2·9% [0·9–6·0]).

**Added value of this study**

Our study produced estimates specific to age, geographical location, and year of measles CFR (from 1990 to 2019) by building on previous estimates in three ways. Our study updated the existing body of evidence to include data published up to Dec 31, 2020, cases occurring up to Dec 31, 2019, and from non-English language studies. Our study incorporated an explicit conceptual framework based on a literature review and expert consultation to identify a suite of covariates shown to be related to measles CFR at the population level. We used a Bayesian meta-regression model with a flexible spline component, to improve capture of variation in CFR by age.

**Implications of all the available evidence**

This model, along with the corresponding estimates, can contribute to a deeper understanding of measles CFR and allow for an increasingly robust assessment of vaccination programmes and other interventions to reduce measles mortality burden.

Previous work has reviewed the available published data on measles CFRs.^3^ An additional study^4^ also modelled estimates of measles CFRs for low-income and middle-income countries (LMICs) among children younger than 5 years (hereafter referred to as children under 5) and those aged 5 years or older, in both community-based and hospital-based settings. Time-varying estimates of CFRs are crucial for understanding patterns of measles mortality across time and location and have been instrumental in understanding acute measles deaths and the effect of various vaccination scenarios.^5^ Despite being a major advancement, these previously published estimates do not include CFR data from after 2016 or an underlying conceptual model for the relation between the CFR and associated covariates.^6^

Additionally, CFR estimates stratified by broad age categories might obscure important variation within age groups, particularly for young children. Both previous systematic review studies^5^, ^6^ showed higher CFRs in children under 5 compared with those aged 5 years or older. However, there are likely to be crucial age-specific variations between infants (aged ≤1 year) and young children (aged 1–4 years), related to maternal antibody presence, immune-system maturation, and vaccination status, among other factors, which go uncaptured in a composite estimate of CFR among all children under 5.^7^ Given that measles incidence tends to be highest among young, unvaccinated children,^8^ an accurate understanding of CFRs among these ages is crucial for understanding the measles mortality burden and developing targeted interventions, such as vaccination campaigns.

Our objective was to use all information available to estimate measles CFRs in LMICs by country, age, and year. As such, we did a full literature review of measles CFR data representing both community and hospital cases in LMICs. We expanded on previous reviews by including data from non-English studies, examining all studies for granular age data, and extending the scope to include data published up to 2020 (representing cases occurring up to Dec 31, 2019). Additionally, we developed a Bayesian meta-regression model to produce location-specific, year-specific, and age-specific estimates of CFR from 1990 to 2019.

## Methods

### Search strategy and selection criteria

We did a systematic review to extend previously published systematic literature reviews^3^, ^4^ on measles CFRs in LMICs to include cases occurring up to Dec 31, 2019, and studies not published in English. To do so, we searched PubMed on Dec 31, 2020, for primary data published from Jan 1, 1980, to Dec 31, 2020, using the search string: (measles[MeSH Terms] OR measles) AND (mortality[MeSH Terms] OR mortality OR “case fatality rate” OR “case fatality ratio” OR “case fatality”). In addition to the literature search, we added studies from previous systematic reviews^3^, ^4^ and the Global Burden of Diseases, Injuries, and Risk Factors Study (GBD)^9^ before deduplicating and screening studies. We screened the study titles and abstracts from the search results and then reviewed the full-text versions of, and extracted summary data from, each study that passed application of our inclusion and exclusion criteria. We included studies in any language if they were included in previous systematic reviews or if, upon screening, they contained primary data on measles cases and deaths from hospital-based, community-based, or surveillance-based reports, including outbreak investigations. We excluded studies if they were not in humans, contained only non-original or non-primary data (ie, reported on the outcomes of another study), reported on data from global or regional surveillance (rather than country-level data), or did not contain relevant information on measles cases and deaths. Additionally, as with the previous reviews, we excluded studies that: reported measles cases and deaths among only restricted populations (eg, communities of internally displaced people or people living with HIV); reported on only long-term measles mortality (eg, death from subacute sclerosing panencephalitis); or were for a high-income country, as defined by the World Bank country income classification in 2017.

We extracted the following data from each study: number of measles cases, number of measles deaths, study year, age, geographical location, and setting (hospital *vs* community; outbreak *vs* non-outbreak). If reported in the study, we also extracted laboratory confirmation of cases and the length of time required after onset of rash for a death to be considered attributable to measles. Data were extracted in a Microsoft Excel 2016 workbook. For each study, we computed annual age-specific CFRs; we included all suspected measles cases and considered all deaths within 30 days of rash onset, unless cases or acute deaths were defined otherwise in individual studies.

On Nov 29, 2022, to determine the evidence available to assess CFRs during the COVID-19 pandemic, we ran our search again, for publications from Jan 1, 2020, to Nov 28, 2022, using the same search string and inclusion and exclusion criteria for screening articles.

### Data analysis

An overview of our entire covariate selection and modelling process can be found in the appendix (p 37). Previous work identified measles incidence and age to be crucial covariates when assessing measles CFR.^3^, ^4^, ^6^ We also selected additional covariates to analyse on the basis of a literature review and expert consultation^6^ that identified five possible underlying mechanisms that contribute to systematic increases or decreases in measles CFR (ie, health-system access and care-seeking behaviours, health-system quality, nutritional status, measles control and epidemiology, and risk of secondary infection) and related population-level indicators with evidence of an association with CFR (ie, mean household size, educational attainment, coverage of measles-containing vaccine first dose [MCV1] and second dose [MCV2], HIV prevalence, extent of health-care availability, stunting prevalence, surrounding conflict, travel time to nearest health-care facility, under-5 mortality, underweight prevalence, vitamin A deficiency prevalence, vitamin A treatment prevalence, and wasting prevalence).

We used previous estimates of country-specific annual incidences of measles that were generated using a semi-mechanistic, stochastic model fitted to observed annual case data.^10^ For remaining covariates, we searched databases of health-related indicators (from WHO, the UN, World Bank, and GBD^9^) to identify possible covariate sets that could be used to represent each indicator. For the following indicators, we were able to find an appropriate covariate set available for nearly all (≥90%) countries and years from 1980 to 2019: HIV prevalence,^9^ MCV1 coverage,^11^ under-5 mortality rate,^12^ vitamin A deficiency prevalence,^9^ and wasting prevalence.^9^ If a covariate set was not available, we identified a proxy covariate set if an appropriate alternative existed on the basis of expert group review. Proxy covariate sets, identified by expert consultation, include the following (appendix p 29): gross domestic product per person^12^ (for level of health care available), maternal education^9^ (for educational attainment), proportion living in an urban setting^12^ and total fertility rate^12^ (for mean household size), and mortality rate due to war and terrorism^9^ (for surrounding conflict). For vitamin A treatment, we were unable to identify an appropriate proxy covariate set; therefore, vitamin A treatment was excluded as a covariate in our model.

If country-level data for specific years were missing in covariate sets, we either computed an interpolated or projected value if there were fewer than 20% of years missing per country for the covariate, or used the GBD regional mean of covariate values for a country if there was at least 20% missingness. For covariates with less than 20% missingness per country, we linearly interpolated missing years using values from adjacent available years of covariate values. If missing covariate values were at the beginning or end of the covariate time series, to complete the full time series we used an annualised rate of change, weighted exponentially, to compute the projected covariate values either forwards or backwards in time, such that weights were more representative of years in the time series that were closer to either the most recent year for forwards projections or the earliest year for backwards projections. For wasting prevalence specifically, which was available from only 1990 onwards, we held the 1990 value constant from 1980 to 1990. We assumed that all covariates took their values in 1980 for pre-1980 years.

We did a two-step data analysis of the covariate sets to determine the strength of relationship and predictive capability of each covariate in describing underlying trends in CFR. Covariates were grouped into five mechanisms, as described previously.^6^ To examine the correlation of covariates, we calculated the pairwise correlation of each of the covariates in each mechanistic group. If there was a correlation greater than 0·8 for any pairwise comparison, covariates were removed sequentially on the basis of the mean highest collinearity between all other covariates in the mechanistic group. As the second step, we did a simple linear regression of the remaining covariates per mechanistic group with the CFR dataset. Covariates were removed as uninformative if they had a p value greater than twice the mean p value across all covariates (ie, >0·33). The final list of covariates selected for inclusion were: age, a categorical indicator for community versus hospital studies, measles incidence, mortality rate due to war and terrorism, maternal education, gross domestic product per person, HIV prevalence, MCV1 coverage, total fertility rate, under-5 mortality, proportion living in urban settings, vitamin A deficiency, and wasting prevalence (appendix pp 3–6).

Finally, we selected a transformation (log, logit, or untransformed) for each covariate by fitting separate linear regressions with each version of the transformed covariate as a predictor and an outcome of logit CFR. Transformation was selected on the basis of the corresponding model with the lowest Akaike information criterion score.^13^ Then, to improve model stability, we standardised each transformed covariate by subtracting the mean of the transformed covariate and dividing by the standard deviation.

Some included studies reported deaths aggregated into large age bands, which could bias results if mortality is higher in the lower end of the age band. To reduce this bias, we fitted the model to the data in two stages. First, we fitted a model to only the data for which there was age granularity representing groupings that were 5 years wide or narrower; data used in this model included ages 0–34 years. This model used the granular data for age and transformed and standardised covariate values for each study midpoint year and fit a Bayesian fixed-effects meta-regression model^14^ with the outcome variable as the logit CFR (for details on model selection see the appendix pp 7–9). We computed SE in logit space per study using the delta method transformation^15^, ^16^, ^17^ and used these values as weights in the meta-regression. To represent the relationship between logit CFR and age, we used a quadratic spline with five knots, with three internal knots, placed uniformly on the basis of data density (ie, equal proportions of input data represented between each knot) resulting in internal knots placed at ages 0·68, 1·31, and 3·83 years. Next, we split cases and deaths from each input data source reporting age bins wider than 1 year differentially on the basis of estimates of country-specific and age-specific incidence^10^ and the overall relative age pattern of CFR estimated in the first stage model. We then recalculated logit of CFR and SE per the newly adjusted number of deaths and cases per new granular age group.

In a second-stage model, we used the same general model formula described earlier, maintaining the spline knot locations identified in the first stage model, and fit our outcome of logit CFR to all data after age splitting (appendix pp 7–9). To ensure the correct direction of association between each covariate and CFR, defined as the direction described in a previous publication,^6^ we placed priors on each regression coefficient. We generated 1000 samples of the regression coefficients from their fitted joint posterior distribution and predicted country-specific and age-specific CFRs in LMICs from 1990 to 2019. We assumed CFRs varied up to age 34 years (the maximum age for which we had age-specific data) and held the CFR constant for older ages.

To understand the effect of modelling changes, adding new covariates, and updating our dataset on our estimates of CFR relative to those produced by Portnoy and colleagues,^4^ we did a decomposition analysis that compared the statistical performance of this new modelling framework to that of Portnoy and colleagues (appendix pp 9–10). Additionally, we computed in-sample and 5-fold out-of-sample cross-validation metrics to assess model performance. The mean error was 0·0035 from in-sample validation and 0·0011 from the out-of-sample validation (appendix p 34). We produced mean estimates of CFR at the level of age, region, or year by using the case-weighted mean of CFR estimates specific to age, location, or year.

We ran sensitivity analyses that excluded studies without information on laboratory confirmation of cases and also without a death definition (appendix p 10). Finally, in an illustrative example of an application of our model, we additionally predicted results for a scenario in which there had been no vaccine introduction (ie, MCV1 coverage was 0% in all countries and years, and incidence values also reflect an absence of vaccination). Methods for estimating incidence in a no-vaccination scenario have been described at length elsewhere.^18^ We performed all analyses and produced all figures within the R computing environment (version 5.4).

This study was exempt from institutional ethics approval as only publicly available data were used.

This study was not registered with PROSPERO or otherwise. This systematic review follows PRISMA guidelines.

### Role of the funding source

An author of this study (LKK) was an employee of the Bill & Melinda Gates Foundation and had a role in the writing of this report and the decision to submit the paper for publication. Gavi, the Vaccine Alliance and the US National Institutes of Health had no role in study design, data collection, data analysis, data interpretation, the writing of the report, or the decision to submit the paper for publication.

## Results

**Figure 1:**
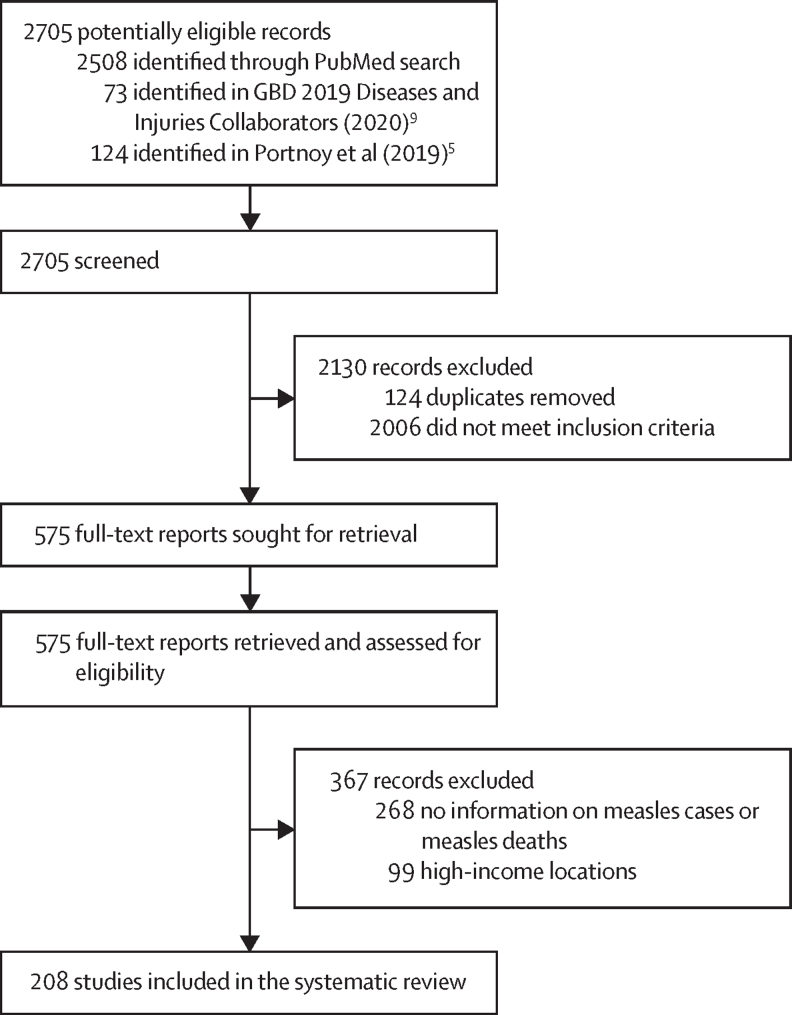
Study selection

Our search identified 2705 records, of which 2130 records were excluded after screening, because they were duplicates or did not meet our inclusion criteria (figure 1). We assessed 575 full-text reports and, after applying inclusion and exclusion criteria, excluded 367 records. We extracted information on measles cases and deaths from 208 studies (appendix pp 18–28). 175 of these studies contained observations from community-based settings and 66 contained observations from hospital-based settings. 126 studies contained granular information on age (ie, at least one age group that was no wider than 5 years), 67 studies presented information without any age granularity, and 15 studies reported age by groups that were wider than 5 years. Overall, 57 unique age groups were represented among the included sources. 88 sources provided information on laboratory confirmation of cases and 84 sources provided information on a definition for a measles-related death.

Information on cases and deaths from before 1980 was available in 44 studies, for 1980–89 in 119 studies, for 1990–99 in 84 studies, for 2000–09 in 67 studies, and for 2010–19 in 71 studies. 75 countries were represented among sources.

Among 1 817 931 cases included among the sources, the crude mean CFR was 5·70% (SD 7·03) and the median CFR was 2·73% (IQR 0·86–7·99). The crude mean CFR was 8·50% (SD 8·35) among hospital-based studies and 4·42% (5·94) among community-based studies.

The mean estimated community-based CFR among people aged 0–34 years for 1990 was 2·60% (95% uncertainty interval [UI] 2·52–2·69) and was 1·32% (1·28–1·36) for 2019.

Among hospital-based settings, the mean estimated CFR across all locations was 10·13% (95% UI 9·67–10·60) in 1990 and 5·35% (5·08–5·64) in 2019. In all regions, estimated CFRs decreased from 1990 to 2019 in both community-based and hospital-based settings (table 1). Across all regions, CFRs were estimated to be highest in the sub-Saharan Africa region in 2019 in both community-based and hospital-based settings.

**Table 1:** Mean estimated case-weighted measles case–fatality ratio by year, setting, and region

	**1990**		**2000**		**2010**		**2019**	
	Community-based	Hospital-based	Community-based	Hospital-based	Community-based	Hospital-based	Community-based	Hospital-based
All locations	2·60% (2·52–2·69)	10·13% (9·67–10·60)	2·16 % (2·10–2·22)	8·48% (8·12–8·85)	1·39% (1·33–1·44)	5·64% (5·33–5·98)	1·32% (1·28–1·36)	5·35% (5·08–5·64)
North Africa and the Middle East	3·33% (3·14–3·53)	12·53% (11·75–13·33)	3·28% (3·08–3·49)	12·32% (11·56–13·13)	2·23% (2·08–2·38)	8·78% (8·18–9·42)	0·83% (0·79–0·86)	3·42% (3·19–3·65)
Sub-Saharan Africa	3·63% (3·50–3·75)	13·63% (13·09–14·19)	3·05% (2·97–3·15)	11·75% (11·30–12·23)	2·10% (2·04–2·17)	8·34% (7·92–8·75)	1·92% (1·86–1·97)	7·67% (7·31–8·07)
Central Europe, eastern Europe, and central Asia	0·66% (0·60–0·71)	2·79% (2·52–3·09)	0·28% (0·26–0·31)	1·22% (1·10–1·35)	0·15% (0·14–0·16)	0·64% (0·58–0·71)	0·18% (0·16–0·20)	0·79% (0·70–0·89)
South Asia	3·16% (3·03–3·29)	12·34% (11·72–12·99)	2·23% (2·08–2·27)	8·79% (8·32–9·27)	1·51% (1·44–1·57)	6·18% (5·82–6·57)	0·82% (0·78–0·86)	3·45% (3·24–3·69)
Latin America	1·81% (1·69–1·92)	7·34% (6·76–7·94)	0·87% (0·82–0·93)	3·66% (3·34–3·98)	0·52% (0·48–0·56)	2·21% (1·99–2·43)	0·35% (0·32–0·39)	1·50% (1·34–1·67)
Southeast Asia, east Asia, and Oceania	0·92% (0·88–0·97)	3·83% (3·58–4·09)	0·65% (0·61–0·67)	2·73% (2·54–2·93)	0·40% (0·38–0·42)	1·71% (1·59–1·85)	0·37% (0·34–0·39)	1·56% (1·45–1·68)

Data are % (95% uncertainty interval).

**Figure 2:**
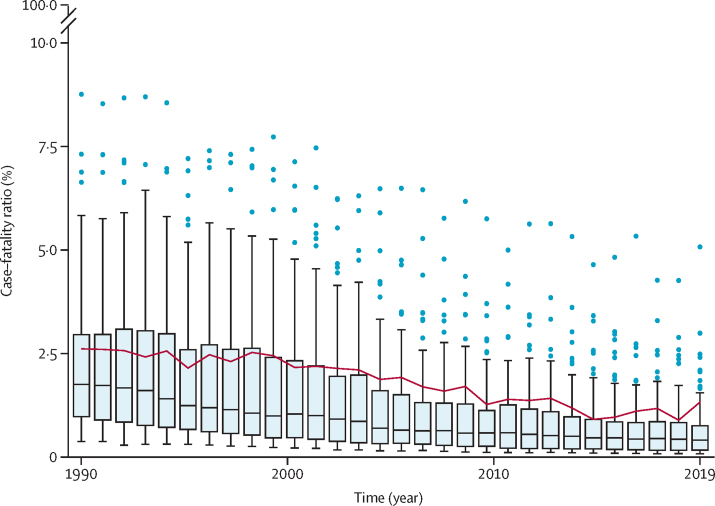
Box plots of estimated country-specific, community-based measles case–fatality rates, by year Horizontal lines represent the median case–fatality ratio, boxes represent the interquartile range, and the whiskers (thin lines) represent adjacent values that are (by convention) within 1·5 times the interquartile range. Dots represent patients outside of the adjacent values, known as outliers. The red line shows the case-weighted mean case–fatality ratio for low-income and middle-income countries, by year.

The median country-specific case-weighted CFR estimates and range by country decreased across the study period (figure 2). All estimated LMIC CFRs decreased from 1990 to 2019. Because mean CFR estimates had been case-weighted, country-specific and year-specific mean values were influenced by the underlying distribution of the ages of people with measles within that specific country and year; a relative distribution of these ages is shown in the appendix (p 41). Age-standardised CFR estimates, which showed that declining CFR trends persisted after age standardisation, can be found in the appendix (p 10), as can country-specific CFR results (pp 46–101) and validation metrics (p 34).

**Figure 3:**
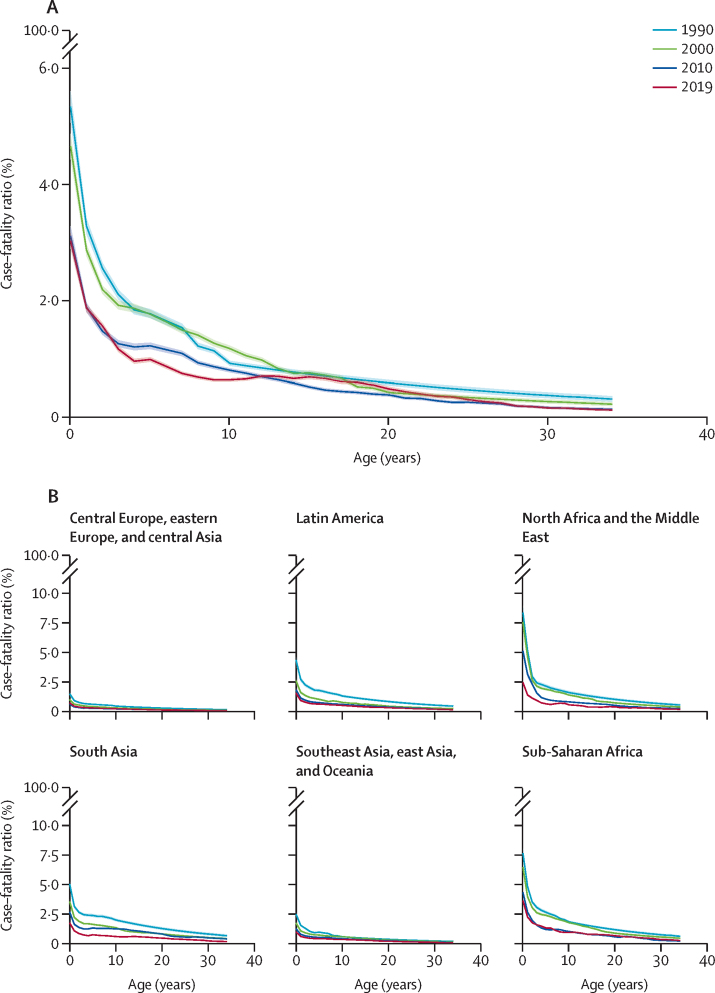
Estimated age-specific, community-based, case-weighted measles case–fatality ratio, by age, year, and location Shaded areas indicate the 95% CI. (A) Estimated age-specific, community-based, case-weighted measles case–fatality ratio for people aged 0–34 years, living in low-income and middle-income countries, for 1990, 2000, 2010, and 2019. (B) Estimated age-specific, community-based, case-weighted measles case–fatality ratio for people aged 0–34 years, for 1990, 2000, 2010, and 2019, by region.

We estimated CFR to be highest among children younger than 1 year and to decline monotonically as age increased (figure 3A). This general trend was consistent across regions and time (figure 3B). For 2019 across LMICs in community-based settings, we estimated that the CFR among children younger than 1 year was 3·03% (95% UI 2·89–3·16), was 1·63% (1·58–1·68) for ages 1–4 years, 0·84% (0·80–0·87) for ages 5–9 years, and was 0·67% (0·64–0·70) for ages 10–14 years. Among LMIC hospital-based settings in 2019, the estimated CFR was 5·33% (95% UI 5·06–5·59) for children younger than 1 year, 2·80% (2·70–2·90) for ages 1–4 years, 1·50% (1·44–1·57) for ages 5–9 years, and 0·87% (0·83–0·91) for ages 10–14 years.

When we projected CFR in a no-vaccination scenario, estimates by region, year, and care setting were larger than the baseline vaccination scenario (table 2). As a result of these differences in CFR and incidence, we estimated that from years 1990 to 2019, there have been approximately 71 million deaths averted attributable to measles vaccination in these LMICs. In 2019, there were 46·3 deaths averted per 100 000 people.

**Table 2:** Mean estimated case-weighted measles case–fatality ratio for a no-vaccination scenario, by year, setting, and region

	**1990**		**2000**		**2010**		**2019**	
	Community-based	Hospital-based	Community-based	Hospital-based	Community-based	Hospital-based	Community-based	Hospital-based
All locations	4·06% (3·84–4·29)	15·15% (14·43–15·94)	3·17% (2·99–3·36)	12·05% (11·45–12·77)	2·34% (2·19–2·51)	9·20% (8·65–9·84)	2·08% (1·94–2·23)	8·24% (7·75–8·86)
North Africa and the Middle East	5·04% (4·66–5·48)	18·26% (17·03–19·65)	4·51%4·19–4·90)	16·45% (15·39–17·67)	3·52% (3·25–3·82)	13·25% (12·33–14·29)	1·83% (1·68–1·99)	7·19% (6·65–7·79)
Sub-Saharan Africa	5·32% (5·04–5·62)	19·03% (18·30–19·87)	4·15% (3·93–4·41)	15·41% (14·73–16·17)	3·67% (3·46–3·92)	13·79% (13·07–14·65)	2·77% (2·59–2·96)	10·77% (10·16–11·51)
Central Europe, eastern Europe, and central Asia	1·97% (1·78–2·14)	7·97% (7·31–8·61)	0·56% (0·50–0·63)	2·39% (2·14–2·67)	0·37% (0·33–0·42)	1·57% (1·39–1·77)	0·33% (0·29–0·38)	1·42% (1·25–1·62)
South Asia	4·50% (4·25–4·76)	16·88% (16·03–17·79)	3·42% (3·20–3·65)	13·21% (12·46–14·05)	2·49% (2·31–2·67)	9·89% (9·25–10·63)	1·61% (1·49–1·74)	6·57% (6·11–7·11)
Latin America	2·75% (2·48–3·01)	10·80% (9·84–11·84)	1·88% (1·68–2·07)	7·58% (6·81–8·41)	1·24% (1·08–1·40)	5·12% (4·50–5·75)	0·88% (0·77–1·01)	3·66% (3·18–4·16)
Southeast Asia, east Asia, and Oceania	1·64% (1·52–1·78)	6·68% (6·21–7·21)	1·16% (1·06–1·26)	4·79% (4·40–5·21)	0·83% (0·75–0·91)	3·45% (3·13–3·79)	0·75% (0·68–0·83)	3·15% (2·86–3·47)

Data are % (95% uncertainty interval).

On re-running our search on Nov 29, 2022, we identified 308 studies published from Jan 1, 2020, to Nov 28, 2022. After screening using the same criteria as before, we found 27 studies for full-text review, of which only two were published studies on measles CFRs during the pandemic period: one in South Sudan (CFR 1·16%)^19^ and another in Ethiopia (7·14%).^20^ Authors of both studies noted various factors that were likely to have affected CFR estimates, including the under-reporting of deaths in the community studied in South Sudan and a high prevalence of malnutrition in the community in Ethiopia. Neither study quantified directly CFR changes caused by the COVID-19 pandemic or mentioned specifically any effect of the pandemic on the country reporting system or surveillance capacity.

## Discussion

Until 2019, there was only one systematic review of measles CFR, which was limited to community-based settings and did not examine temporal changes in CFR.^3^ In 2019, an updated systematic review^4^ expanded the literature by including CFRs among hospital-based settings, and used a time-varying model to estimate CFR by location and year. Although more comprehensive than the first review, this updated review did not include age-specificity beyond variation among ages under 5 years versus 5 years or older, and the covariates included were not selected via a transparent, systematic process. Our new study addresses these shortcomings by: updating the former literature searches; basing covariate selection on widespread expert consultation, a literature review, and selection through a statistical process; and accounting for the distribution of age in the modelling process. Our study included 40 new sources from 21 new countries. We statistically tested for the inclusion of new covariates with a known relation to measles CFR, such as vitamin A deficiency prevalence. Community-based settings had lower CFRs than hospital-based settings. Higher measles incidence, under-5 mortality, the proportion of people living in urban settings, and vitamin A deficiency prevalence were associated with higher CFRs, whereas higher levels of maternal education and MCV1 coverage were associated with lower CFRs.

We estimated CFRs in people aged 0–34 years in community-based settings to be 2·60% (95% UI 2·52–2·69) and to have declined to 1·32% (95% UI 1·28–1·36) by 2019. We estimated higher CFRs in hospital-based settings relative to community-based settings, which was consistent with previous findings^4^ that probably observed the most severe cases, which required hospitalisation. We estimated infants to have the highest CFRs. In this age group, the risk of infection is influenced by the persistence of maternal antibodies, which in turn depend on gestational age and underlying maternal immunity rates.^7^ On an individual level, the presence of maternal antibodies might also mitigate the severity of infection, potentially leading to lower CFRs than in absence of these antibodies. In our analysis, after controlling for study-level covariates, our model suggested population-level CFR decreases monotonically with age, consistent with previous studies,^21^ which might be because infants who acquire measles do not have sufficient maternal antibodies to prevent infection. Increasingly detailed and robust data collection in these youngest ages will be crucial for assessing this relationship further.

In an illustrative example of an application of our findings, we also estimated CFRs for a no-vaccination scenario that reflected both 0% MCV coverage and the corresponding measles incidence values if there was no vaccination. We used these CFR estimates to estimate a metric of the number of deaths averted owing to vaccination, which is similar to indicators that are used to assess the effectiveness of vaccination programmes and, through this example, we show our model can be used for such evaluations.

The COVID-19 pandemic probably affected measles CFRs. Reported measles incidence in most countries decreased, beginning in 2020, following lockdowns and physical-distancing measures. Reduced incidence is generally associated with reduced CFRs; however, this relationship might have been countered by important changes in other underlying drivers of case fatality, such as nutritional status^22^ and health-system quality and access.^23^

Limitations in data availability currently prevent reliable CFR estimation during the pandemic period. Additional data (on both the initial pandemic period and on the period afterwards, as health systems continue to rebound from long-lasting pandemic effects) will be published in the coming months and years. It will be important to monitor this evidence to assess the effects of the pandemic on health-system capacity, nutrition, and other factors related to case fatality, especially as the risk of widespread outbreaks could increase with ongoing disruptions to vaccination systems and increased numbers of susceptible people globally.

This study has several limitations. We did not include studies representing particular populations that might be especially susceptible to both measles infection and increased case fatality, such as refugees and internally displaced people; unfortunately, there are too few data on these subpopulations to accurately assess their current situation. We assumed that the CFRs presented in each study were nationally representative, which might have biased the relationship between CFR and national-level covariates. Furthermore, the included studies were heterogeneous in design and setting and, despite the inclusion of UIs, additional uncertainty owing to heterogeneity in the original data might have remained. Also, we assumed that the age distribution of people with measles in those studies that did not report age specificity followed the same relative age distribution of cases estimated nationally in that country and year during our age-splitting process.

We were constrained by the small number of studies that both reported laboratory confirmed cases and defined death attributable to measles. We therefore included all available studies in our analysis, regardless of whether they did either, to avoid compositional bias stemming from differences in study-level demographics in our estimates. Also, owing to data limitations, we were unable to estimate CFR differentially by sex or gender and race or ethnicity.

We were not able to incorporate uncertainty from our covariates and model specification. As such, our uncertainty estimates only reflect uncertainty in the CFR modelling process itself, without any additional factors. Also, for all covariates that passed our analysis checks, we included each in our modelling framework with priors to govern the direction of association estimated by the model. In this process, four covariates (mortality rate due to war and terrorism, wasting prevalence, HIV prevalence, and gross domestic product per person) no longer contributed statistically significantly to our model, so were removed. We emphasise that these covariates might still be related to measles CFR; their exclusion was a result of the underlying collinearity of our covariates that suggested little added predictive benefit to their inclusion in the final model. We developed our model for projection rather than for inference. We did not test for interactions between covariates. Also, we did not examine individual-level relationships between CFR and the covariates included in our modelling framework, but instead assessed population-level trends for use in population-level modelling, so the presented associations between covariates and CFR should not be considered causal.

Our study improved upon previous estimates of measles CFR by incorporating new data sources, systematically identifying covariates, and including improved age-specific variation. These estimates might aid in future assessments of measles mortality and vaccination programmes by decision makers at the global and country level.

## Data sharing

This study complies with the Guidelines for Accurate and Transparent Health Estimates Reporting.^24^ The results of this study are supported by extracted publicly available data; for sources see the appendix (pp 18–28) and https://zenodo.org/record/7633577#.Y-kbkuzMIV8. Covariate sets are available from their original sources.^9^, ^10^, ^11^, ^12^ Estimates of measles CFR by country, year, and age can be found at https://zenodo.org/record/7633577#.Y-kbkuzMIV8, and the R computer code can be found at https://zenodo.org/record/7633577#.Y-kbkuzMIV8.

## Declaration of interests

ANS, JFM, MJ, MF, RER, ELBR, and AP received funding for this work from, and LKK is employed by, the Bill & Melinda Gates Foundation. ANS received grant support from the US National Institutes of Health (NIH; F31AI167535). ANS, JFM, and MJ received grant support from Gavi. MF received grant support from WHO. PO declares no competing interests. The content of this work is the sole responsibility of the authors and does not represent the official views of the NIH.
