# Supplementary material for: Estimating national-level measles case–fatality ratios in low-income and middle-income countries: an updated systematic review and modelling study
Source: Lancet Glob Health. 2023 Mar 14;11(4):e516–24. doi: 10.1016/S2214-109X(23)00043-8 (PMC10030458; doi:10.1016/S2214-109X(23)00043-8)
Supplement: Supplementary appendix [file mmc1.pdf]

THE LANCET
Global Health

Supplementary appendix

This appendix formed part of the original submission and has been peer reviewed. We post it as supplied by the authors.

Supplement to: Sbarra AN, Mosser JF, Jit M, et al. Estimating national-level measles case–fatality ratios in low-income and middle-income countries: an updated systematic review and modelling study. *Lancet Glob Health* 2023; **11:** e516–24.

Supplementary Information: Estimating national-level measles case fatality ratios in low- and middle-income countries: an updated systematic review and modelling study

Section 1. Covariate selection via statistical analysis 3

Section 2. Model selection 7

Section 3. Final covariate processing and model structure 7

Section 4. Decomposition analysis for validating changes to model structure, covariates, and input data 9

Section 5. Supplementary Results 10

Section 5. Supplementary Tables 11

Supplementary Table 1. GATHER compliance checklist. 11

Supplementary Table 2. PRISMA compliance checklist. 13

Supplementary Table 3. PRISMA abstract compliance checklist 17

Supplementary Table 4. Input data sources for final model. 18

Supplementary Table 5. Proxy covariate sets used for analysis. 29

Supplementary Table 6. Covariate set values by country in 2019 30

Supplementary Table 7. Second step model fitted meta-regression coefficients. 33

Supplementary Table 8. In-sample validation metrics from first stage decomposition analysis ... 33

Supplementary Table 9. Out-of-sample validation metrics from first stage decomposition analysis. 33

Supplementary Table 10. In-sample validation metrics from second stage decomposition analysis. 33

Supplementary Table 11. Out-of-sample validation metrics from first and second stage decomposition analysis 34

Supplementary Table 12. In-sample (IS) and out-of-sample (OOS) validation metrics from final model using age split input data for comparison. 34

Supplementary Table 13. In-sample (IS) and out-of-sample (OOS) validation metrics from final model using original (pre-age split) data for comparison. 34

Supplementary Table 14. Age-specific CFR by region in 2019. 35

Section 7. Supplementary Figures 37

Supplementary Figure 1. Overview of modeling process. 37

Supplementary Figure 2. Recent data available by country. 38

Supplementary Figure 3. Relative age pattern from first-stage model with 4 knots (with 2 internal). 39

Supplementary Figure 4. Relative age pattern from first-stage model with 5 knots (with 3 internal). 39

Supplementary Figure 5. Relationship between age of input data and standardized measles incidence from country-year input data was collected. 40

Supplementary Figure 6. Relationship between age of input data and standardized first-dose measles-containing vaccine (MCV1) coverage from country-year input data was collected. 41

Supplementary Figure 7. Mean age of measles cases by country and year. 41

Supplementary Figure 8. Standardized and unstandardized estimates of case-weighted measles CFR across all countries from 1990 to 2019. 42

Supplementary Figure 9a-d. Age-specific, community-based, case-weighted case fatality ratio (CFR) estimates among 0–14-year-olds for low- and middle-income countries – for single years 1990, 2000, 2010, and 2019. 43

Supplementary Figure 10a-d. Age-specific, community-based, case-weighted case fatality ratio (CFR) (CFR) estimates among 0–14-year-olds, by region – for single years 1990, 2000, 2010, and 2019. 45

Supplementary Figure 11. Heat maps of age- and year-specific CFR by community- and hospital-based settings by country. 46

Supplementary Figure 12. Distribution of CFR values for studies providing information on laboratory confirmation of cases (1) versus not providing information on laboratory confirmation of cases (0). 102

Supplementary Figure 13. Distribution measles incidence values used for covariates of country-years for studies providing information on laboratory confirmation of cases (1) versus not providing information on laboratory confirmation of cases (0). 102

Supplementary Figure 14. Distribution MCV1 coverage values used for covariates of country-years for studies providing information on laboratory confirmation of cases (1) versus not providing information on laboratory confirmation of cases (0). 102

Supplementary Figure 15. CFR estimates from framework using all studies versus only studies providing information on laboratory confirmation of cases, for select years. 103

Supplementary Figure 16. CFR estimates from framework using all studies versus only studies providing definition of death attributable to measles, for select years. 103

References ***105***

Section 1. *Covariate selection via statistical analysis*

Section 1.1. *Rationale for covariate inclusion*

We selected covariates for the remainder of this analysis based on a previous publication that used expert consultation to develop a conceptual framework of mechanisms related to measles CFR and literature review to assess the body of evidence related to population-level factors associated with these mechanisms.

Covariates associated with the underlying mechanism of health care access and care seeking were maternal education, mortality rate due to war and terrorism, and proportion living in urban settings. Each of these individual covariates contribute to the ability for persons to access health care as well as might influence behavior contributing to the decision to seek care, ultimately leading to higher CFR if care is not sought or accessed.

Covariates associated with the underlying mechanism of health care quality were under-5 mortality rate and GDP per capita. Higher under-5 mortality rates or lower GDP per capita might be associated with lower health care quality which might be related to higher CFR.

Covariates associated with the underlying mechanism of risk of secondary infection were HIV prevalence and total fertility rate (TFR). Based on the risk of secondary infection associated with higher HIV prevalence or TFR, CFR might be higher.

Covariates associated with the underlying mechanism of nutritional status were vitamin A deficiency prevalence and wasting prevalence. Higher vitamin A deficiency prevalence or wasting prevalence could be associated with higher CFR.

Covariates associated with the underlying mechanism of general measles control and epidemiology were MCV1 and MCV2 coverage. Lower MCV1 or MCV2 coverage values could be associated with higher CFR.

Section 1.2. *Additional details on covariate interpolation*

The following covariate sets did not require interpolation or use of regional values: education, maternal education, war rate due to mortality and terrorism, health access and quality index, universal health coverage, sociodemographic index, stunting prevalence, wasting prevalence, underweight prevalence, vitamin A deficiency prevalence, HIV prevalence, and MCV2 coverage. For 12 countries, we interpolated covariate values for GDP per capita; we also used regional values in 23 countries. For 7 countries, we interpolated covariate values for under-5 mortality rate; we also used regional values in 2 countries. We used regional covariate values in 6 countries for total fertility rate. We used regional covariate values in 14 countries for MCV1 coverage. For 1 country, we interpolated covariate values for proportion living in urban settings; we also used regional values in 2 countries.

Section 1.3. *Test for collinearity per underlying mechanism*

For the underlying mechanism of “health care access and care seeking”, we tested covariate sets for education, maternal education, proportion living in an urban setting, and mortality rate due to war and terrorism. Education was correlated with maternal education; correlation coefficients shown below. As education was more correlated with the other covariates relative to maternal education, it was removed from further analysis. Covariates moving on to the second step of data analysis for the “health care access and care seeking” mechanism were: maternal education, proportion living in an urban setting, and mortality rate due to war and terrorism.

	Education	Maternal education	Prop. living in urban setting	War mortality rate
Education	1.0	0.9965	0.6539	-0.0648
Maternal education		1.0	0.6523	-0.0645
Prop. living in urban setting			1.0	-0.537
War mortality rate				1.0

For the underlying mechanism of "health care quality", we tested covariate sets for under-5 mortality rate, health access and quality index, universal health coverage, GDP per capita, and sociodemographic index. Under-5 mortality rate, health access and quality index, universal health coverage and sociodemographic index were all correlated with each other; correlation coefficients shown below. Health access and quality index, universal health coverage, and sociodemographic index were more correlated with the other covariates relative to under-5 mortality rate, and so they were removed from further analysis. Covariates moving on to the second step of data analysis for the "health care quality" mechanism were: under-5 mortality rate, and GDP per capita.

	Under-5 mortality rate	Health access and quality index	Universal health coverage	GDP per capita	Sociodemographic Index
Under-5 mortality rate	1.0	-0.8027	-0.8346	-0.3920	-0.8525
Heath access and quality index		1.0	0.9916	0.6428	0.9365
Universal health coverage			1.0	0.6311	0.9417
GDP per capita				1.0	0.6159
Sociodemographic Index					1.0

For the underlying mechanism of "nutritional status", we tested covariate sets for stunting prevalence, wasting prevalence, underweight prevalence, and vitamin A deficiency prevalence. Stunting prevalence, underweight prevalence, and wasting prevalence were correlated with each other; correlation coefficients shown below. Stunting prevalence, and underweight prevalence were more correlated with the other covariates relative to wasting prevalence, and so they were removed from further analysis. Covariates moving on to the second step of data analysis for the "nutritional status" mechanism were: wasting prevalence and vitamin A deficiency prevalence.

	Stunting	Wasting	Underweight	Vitamin A deficiency
Stunting	1.0	0.7597	0.8970	0.6965
Wasting		1.0	0.8927	0.5687
Underweight			1.0	0.6509
Vitamin A deficiency				1.0

For the underlying mechanism of "risk of secondary infection", we tested covariate sets for HIV prevalence and total fertility rate. The covariates were not correlated with each other; correlation coefficient shown below. Both covariates moved on to the second step of data analysis for the "risk of secondary infection" mechanism.

	HIV prevalence	TFR
HIV prevalence	1.0	0.2279
TFR		1.0

For the underlying mechanism of "measles control and epidemiology", we tested covariate sets for MCV1 and MCV2 coverage. The covariates were not correlated with each other; correlation coefficient shown below. Both covariates moved on to the second step of data analysis for the "measles control and epidemiology" mechanism.

	MCV1 coverage	MCV2 coverage
MCV1 coverage	1.0	0.4713
MCV2 coverage		1.0

Section 1.4. *Test for predictive capacity per underlying mechanism*

For the mechanism of "health care access and care seeking", no covariates tested had p-values greater than 0.3 (see below). Therefore, all remaining covariates (maternal education, proportion living in urban setting, and mortality rate due to war and terrorism) were kept as covariate sets for the remainder of the analysis.

	Estimate	P-value
Intercept	0.0876	< 0.0001
Maternal education	-0.0039	0.019
Prop urban	-0.0435	0.12
War mortality rate	13.15116	0.12

For the mechanism of “health care quality”, no covariates tested had p-values greater than 0.3 (see below). Therefore, all remaining covariates (under-5 mortality rate and GDP per capita) were kept as covariate sets for the remainder of the analysis.

	Estimate	P-value
Intercept	-0.0001441	0.99
Under-5 mortality rate	0.0004097	<0.0001
GDP per capita	0.0000007766	0.039

For the mechanism of “nutritional status”, no covariates tested had p-values greater than 0.3 (see below). Therefore, all remaining covariates (wasting prevalence and vitamin A deficiency prevalence) were kept as covariate sets for the remainder of the analysis.

	Estimate	P-value
Intercept	0.0097	0.19
Wasting	0.0901	0.27
Vitamin A deficiency	0.0767	0.13

For the mechanism of “risk of secondary infection”, no covariates tested had p-values greater than 0.3 (see below). Therefore, all remaining covariates (HIV prevalence and total fertility rate) were kept as covariate sets for the remainder of the analysis.

	Estimate	P-value
Intercept	-0.01540	0.17
HIV prevalence	0.2675	0.25
TFR	0.008409	0.0003

For the mechanism of “measles control and epidemiology”, MCV2 coverage had a p-value greater than 0.3 (see below). Therefore, MCV1 coverage was the only covariate sets kept for the remainder of the analysis.

	Estimate	P-value
Intercept	0.1160	<0.0001
MCV1 coverage	-0.1078	<0.0001
MCV2 coverage	0.0021	0.86

Section 2. *Model selection*

Section 2.1. *First stage model with age granular data*

We analyzed the relationship between age and CFR in reported studies with age-specific data both with and without controlling for other covariates. There was a consistent relationship between covariate values and CFR values, particularly for measles incidence and MCV1 coverage (Supplementary Figures 5-6). Taken together, these suggested that the relationship between age and CFR was confounded by these other covariates, and therefore we elected to adjust for other covariates in our first-stage model.

Section 2.2. *Knot selection*

We ran both first and second stage models with both 4 knots (with 2 internal) and 5 knots (with 3 internal) placed uniformly on data density and selected the best performing model based on the lowest Akaike information criterion (AIC) score among results from the second stage model. This process selected the model with 5 knots (AIC: 174907) instead of 4 knots (AIC: 175086).

Section 2.3. *Inclusion of random effects*

We additionally tested the inclusion of random effects in our second stage model, by testing a random effect placed on each study. This approach caused the coefficient for the community versus hospital-setting indicator to become 0, with a non-significant p-value (p-value=1). Because we know these sets of studies (i.e. those from community-based settings and those from hospital-based settings) were collected from different underlying populations with known difference in measles severity, we elected to use a model without the inclusion of random effects.

Section 3. *Final covariate processing and model structure*

For covariates requiring interpolation, we used the following formula:

$$y = y_1 + \frac{(x - x_1)(y_2 - y_1)}{(x_2 - x_1)}$$

Following transformation, covariates were standardized as follows such that μ represents the mean transformed covariate value and σ represents the standard deviation of the transformed covariate value:

$$standardized\ covariate = \frac{transformed\ covariate - \mu}{\sigma}$$

Our final first stage CFR model (that only uses age specific input data) follows the following structure. Using transformed and standardized covariate values for each study midpoint year, we fit a Bayesian fixed-effects meta-regression model[1] with the outcome variable of the logit of CFR. We computed standard error in logit space per study using the delta transformation and used these values as weights in the meta-regression. Before transforming to logit space, CFR ratios equalling 0 were offset to 0.0002202378 and ratios equal to 1 were offset to 0.9999999999999.

Our regression equation is as follows:

$$y_i = X_i(\beta) + \epsilon_i$$

$$\epsilon_i \sim N(0, \Lambda)$$

where y_i is the vector of observations of logit of CFR from the ith study, X_i is a vector of covariates paired with each data observation, β are regression coefficients ($\beta_{community\ indicator}$, $\beta_{incidence}$, $\beta_{mortality\ rate\ due\ to\ war\ and\ terrorism}$, $\beta_{maternal\ education}$, $\beta_{GDP\ per\ capita}$, $\beta_{HIV\ prevalence}$, $\beta_{MCV1\ coverage}$, $\beta_{total\ fertility\ rate}$, $\beta_{under\ 5\ mortality\ rate}$, $\beta_{proportion\ living\ in\ urban\ setting}$, $\beta_{vitamin\ A\ deficiency\ prevalence}$, $\beta_{wasting\ prevalence}$, β_{age}), and ϵ_i are measurement errors with a given covariance Λ. For age, our β coefficient is represented as a function f representing a quadratic spline with 5 knots (3 internal) placed uniformly based on data density at locations 0, 0.68, 1.31, 3.83 and 34 years. This can be represented via the following generalized equation for each data interval i:

$$s_i(x) = a_i x^2 + b_i x + c_i$$

For $x \in [x_i, x_{i+1}]$ and $i = 1, 2, \ldots, n-1$. Data intervals are based on knot locations. Additionally, we included a prior to ensure a right linear tail on our quadratic spline function.

Our final second stage model (that uses all data) is as follows. Model specifications are identical to the first stage as previously defined, except with the following additional priors:

$$\beta_{community\ indicator} \sim Uniform(-\infty, 0)$$
$$\beta_{incidence} \sim Uniform(0, \infty)$$
$$\beta_{mortality\ rate\ due\ to\ war\ and\ terrorism} \sim Uniform(0, \infty)$$
$$\beta_{maternal\ education} \sim Uniform(-\infty, 0)$$
$$\beta_{GDP\ per\ capita} \sim Uniform(-\infty, 0)$$
$$\beta_{HIV\ prevalence} \sim Uniform(0, \infty)$$
$$\beta_{MCV1\ coverage} \sim Uniform(-\infty, 0)$$
$$\beta_{total\ fertility\ rate} \sim Uniform(0, \infty)$$
$$\beta_{under\ 5\ mortality\ rate} \sim Uniform(0, \infty)$$
$$\beta_{proportion\ living\ in\ urban\ setting} \sim Uniform(0, \infty)$$
$$\beta_{vitamin\ A\ deficiency\ prevalence} \sim Uniform(0, \infty)$$
$$\beta_{wasting\ prevalence} \sim Uniform(0, \infty)$$

Priors in this work were only used to impose directionality on covariates, such that the direction of association estimated was consistent with the observed relationship in the literature identified by previous literature review.[2] Therefore, we did not update the priors at any point in this analysis as these directions of association are fixed.

Following our first-stage model, we used the following method to age-split our input data that was reported from sources in age groups wider than 1 year. For the given age range, we computed the proportion of cases for each single age year within the age range given overall age incidence. We then split the number of reported cases per study based on those proportions to generate single age year specific case counts.

Using the total number of deaths reported in the study for the entire age range, we then used the following algorithm:

$$X = \frac{D}{\sum_{a=b}^{B}(C_a * R_a)}$$

, where D was the total number of deaths reported for the age range per study, was the total number of C_a is the number of age-split cases per age a, and R_a was the reference proportion which was calculated taking the ratio of predicted age specific CFR from our first stage-model relative to the CFR among 0-year-olds CFR_a/C_0.Then, we use the following to compute our adjusted CFR ($aCFR$) and adjusted number of deaths (aD_a) per single age year a to use as input data in our model:

$$aCFR_a = X * R_a$$

$$aD_{a=} aCFR_a * C_a$$

We then use our second stage model (similar in specifications) to produce final estimates of age-, year-, and location-specific CFR using our age-split input data.

In model fitting, we use linear point optimization via cyipopt[3] described in detail in the technical documentation[1] to the methods used in this paper. Therefore, as MCMC or another sampling algorithm was not used, a burn-in period was not applicable to our analysis. Since we used a numerical optimization technique[1] to fit our model, we do not need to perform replication tests as would be needed to assess stability from a model fit using MCMC. We generated 1000 posterior samples to allow for robust calculations for various uncertainty intervals. We calculated 95% uncertainty intervals (UI) for all estimates.

Section 4. Decomposition analysis for validating changes to model structure, covariates, and input data

To increase the robustness and rigor of measles CFR modeling, we considered various updates to the model structure, covariates, and input data sources relative to the model previously published by Portnoy et al.[4] With updates to each component (model structure, covariates, and input data), we tracked the overall change in model performance to ensure updates were statistically beneficial in the estimation of measles CFR. Specific steps and validation at each step are described in each subsequent section.

***Section 4.1.** First stage, updates to model structure*

We made the following sequential adaptations to the log-linear model published previously:

- Model 0: Generalized linear model, with log link and cases as weights
- Model 1.A: Generalized linear model, with log(CFR) as outcome and cases as weights
- Model 1.B: Generalized linear model, with logit(CFR) as outcome and cases as weights
- Model 1.C: Bayesian meta-regression, with logit(CFR) as outcome and standard error as weights

The structure of Model 0 is identical to the model previously published[4], and serves as our baseline. In order to more accurately represent CFR as a ratio bounded between 0 and 1, we first removed the log link from the model and instead log (Model 1.A) then logit (Model 1.B) transformed CFR as our outcome. In order to best capture the underlying uncertainty from the data, we then implemented a Bayesian meta-regression framework using standard errors as weights (Model 1.C). We compared both in- and out-of-sample validation for each model iteration. Model 1.C performed best among both in- and out-of-sample validation exercises across metrics (Supplementary Tables 8-9) yielding generally lower root mean squared error (RMSE), mean error and man absolute error and higher correlation.

***Section 4.2.** Second stage, updates to covariates*

We made the following sequential adaptations to the best model (previously referred to as Model 1.C) from our first decomposition step:

- Model 1: Previously described Model 1.C with original covariates and original data inputs
- Model 2: Previously described Model 1.C with updated covariates and original data inputs

We compared the best model using original covariates and data inputs to a new model fit using the updated covariate set. We compared the performance of these two models to the original model version (Model 0) in Supplementary Tables 10-11. Model 2 performed best across most in- and out-of-sample validation metrics yielding generally lower root mean squared error (RMSE), mean error and mean absolute error and higher correlation.

Section 4.3. *Third stage, updates to input data sources*
We made the following sequential adaptations to the best model from our second decomposition step updates:

- Model 2: Previously described Model 1.C with updated covariates and original data inputs
- Model 3: Previously described Model 1.C with updated covariates and updated data inputs

There were 40 additional new studies added across 21 additional countries. Because the input data sources were changing, we did not compare validation metrics to previous decomposition steps. Full model validation can be found in Supplementary Tables 12-13.

The mean predicted CFR from 1990 to 2015 in the previously published model was 1.5% (95% confidence interval (CI): 0.5 – 3.1%) in community-based settings and 2.9% (95% CI: 0.9 – 6.0%) in hospital-based settings. Our findings had a mean case-weighted CFR from 1990 to 2015 of 2.2% (95% uncertainty interval (UI): 2.1 – 2.2%) in community-based settings and in 8.4% (95% uncertainty interval (UI): 8.1 – 8.8%) in hospital-based settings.

Section 5. *Supplementary Results*

Section 5.1. *Age-standardized results*
Because the age distribution of cases within a country impacts the ability to compare trends across locations, we also computed country-specific age-standardized CFRs using a reference population of the global age pattern of cases from 1990 as well as the general population age distribution from the UN in 1990 (Supplementary Figure 8). Age-standardized estimates of CFR allow users to more directly compare estimates across locations and years.

Section 5.2. *Sensitivity analyses*
We ran sensitivity analyses to investigate the implications of using all studies regardless of if they provided information on laboratory confirmation of cases or a definition of a death attributable to measles. Generally, studies that reported information on laboratory confirmation of cases were from countries and years with lower measles incidence, higher MCV1 coverage, and lower CFRs relative to studies that did not report information on laboratory confirmation (Supplementary Figures 10-11). In a sensitivity analysis excluding first studies without information on laboratory confirmation of cases, we were estimated systematically lower CFRs than when including all studies in our model (Supplementary Figures 13).

Additionally, studies reporting definitions of deaths attributable to measles were most often from hospital-based settings rather than community-based settings (Chi-squared p-value < 0.0001). When excluding studies without information on a death definition, we also estimated systematically lower CFRs than when including all studies in our model (Supplementary Figures 14).

Section 5. Supplementary Tables

Supplementary Table 1. GATHER compliance checklist.

Item number	Checklist item	Reported on page number(s):
**Objectives and funding**		
1	Define the indicator(s), populations (including age, sex, and geographic entities), and time period(s) for which estimates were made.	Introduction (page 4, lines 175-181)
2	List the funding sources for the work.	Acknowledgements (page 11, line 544-545)
**Data inputs**		
For all data inputs from multiple sources that are synthesised as part of the study:		
3	Describe how the data were identified and how the data were accessed.	Methods (pages 4-5, lines 184-192)
4	Specify the inclusion and exclusion criteria. Identify all ad hoc exclusions.	Methods (pages 4-5, lines 192-206)
5	Provide information on all included data sources and their main characteristics. For each data source used, report reference information or contact name/institution, population represented, data collection method, year(s) of data collection, sex and age range, diagnostic criteria or measurement method, and sample size, as relevant.	Supplementary Table 4 (SI pages 18-28)
6	Identify and describe any categories of input data that have potentially important biases (e.g., based on characteristics listed in item 5).	Methods (page 7, lines 327-328); Discussion (page 10, lines 479-496)
For data inputs that contribute to the analysis but were not synthesised as part of the study:		
7	Describe and give sources for any other data inputs.	Methods (page 5, lines 231-238)
For all data inputs:		
8	Provide all data inputs in a file format from which data can be efficiently extracted (e.g., a spreadsheet rather than a PDF), including all relevant meta-data listed in item 5. For any data inputs that cannot be shared because of ethical or legal reasons, such as third-party ownership, provide a contact name or the name of the institution that retains the right to the data.	Data sharing statement (page 11, lines 527-533)
**Data analysis**		
9	Provide a conceptual overview of the data analysis method. A diagram might be helpful.	Supplementary Figure 1 (SI page 37)
10	Provide a detailed description of all steps of the analysis, including mathematical formulae. This description should cover, as relevant, data cleaning, data pre-processing, data adjustments and weighting of data sources, and mathematical or statistical model(s).	Methods (pages 4-7, line 217-333); Supplementary Information Section 3 (SI pages 7-9, lines 183-263)
11	Describe how candidate models were evaluated and how the final model(s) were selected.	Supplementary Information Section 2 (SI page 4, lines 162-182)

12	Provide the results of an evaluation of model performance, if done, as well as the results of any relevant sensitivity analysis.	Methods (page 7, lines 321-323)
13	Describe methods for calculating uncertainty of the estimates. State which sources of uncertainty were, and were not, accounted for in the uncertainty analysis.	Methods (page 7, lines 313-315)
14	State how analytic or statistical source used to generate estimates can be accessed.	Data sharing statement (page 11, lines 527-533)
**Results and discussion**		
15	Provide published estimates in a file format from which data can be efficiently extracted.	Data sharing statement (page 11, lines 527-533)
16	Report a quantitative measure of uncertainty of the estimates (e.g., uncertainty intervals).	Results (page 8, line 368-392)
17	Interpret results in light of existing evidence. If updating a previous set of estimates, describe the reasons for changes in estimates.	Discussion (page 9, lines 431-456)
18	Discuss limitations that affect interpretation of the estimates.	Discussion (page 10, line 479-511)

Supplementary Table 2. PRISMA compliance checklist.

Section and Topic	Item #	Checklist item	Location where item is reported
**TITLE**			
Title	1	Identify the report as a systematic review.	Title (line 2)
**ABSTRACT**			
Abstract	2	See the PRISMA 2020 for Abstracts checklist.	Supplementary Table 3 (SI page 17)
**INTRODUCTION**			
Rationale	3	Describe the rationale for the review in the context of existing knowledge.	Research in context (page 3, lines 103-140)
Objectives	4	Provide an explicit statement of the objective(s) or question(s) the review addresses.	Introduction (page 4, lines 175-181)
**METHODS**			
Eligibility criteria	5	Specify the inclusion and exclusion criteria for the review and how studies were grouped for the syntheses.	Methods (page 4-5, lines 192-206)
Information sources	6	Specify all databases, registers, websites, organisations, reference lists and other sources searched or consulted to identify studies. Specify the date when each source was last searched or consulted.	Methods (page 4, lines 185-186)
Search strategy	7	Present the full search strategies for all databases, registers and websites, including any filters and limits used.	Methods (page 4, lines 185-189)
Selection process	8	Specify the methods used to decide whether a study met the inclusion criteria of the review, including how many reviewers screened each record and each report retrieved, whether they worked independently, and if applicable, details of automation tools used in the process.	Methods (page 4-5, lines 191-215)
Data collection process	9	Specify the methods used to collect data from reports, including how many reviewers collected data from each report, whether they worked independently, any processes for obtaining or confirming data from study investigators, and if applicable, details of automation tools used in the process.	Contributors (page 11, lines 521-522)
Data items	10a	List and define all outcomes for which data were sought. Specify whether all results that were compatible with each outcome domain in each study were sought (e.g. for all measures, time points, analyses), and if not, the methods used to decide which results to collect.	Methods (page 5, lines 208-215)
	10b	List and define all other variables for which data were sought (e.g. participant and intervention characteristics, funding sources). Describe any assumptions made about any missing or unclear information.	Methods (page 5, lines 208-215)

Section and Topic	Item #	Checklist item	Location where item is reported
Study risk of bias assessment	11	Specify the methods used to assess risk of bias in the included studies, including details of the tool(s) used, how many reviewers assessed each study and whether they worked independently, and if applicable, details of automation tools used in the process.	Methods (page 5, lines 208-215)
Effect measures	12	Specify for each outcome the effect measure(s) (e.g. risk ratio, mean difference) used in the synthesis or presentation of results.	Methods (page 5, lines 208-215)
Synthesis methods	13a	Describe the processes used to decide which studies were eligible for each synthesis (e.g. tabulating the study intervention characteristics and comparing against the planned groups for each synthesis (item #5)).	Methods (pages 6-7, lines 285-325)
	13b	Describe any methods required to prepare the data for presentation or synthesis, such as handling of missing summary statistics, or data conversions.	Methods (pages 5-7, lines 208-307)
	13c	Describe any methods used to tabulate or visually display results of individual studies and syntheses.	Methods (page 5, lines 212-215)
	13d	Describe any methods used to synthesize results and provide a rationale for the choice(s). If meta-analysis was performed, describe the model(s), method(s) to identify the presence and extent of statistical heterogeneity, and software package(s) used.	Methods (pages 6-7, lines 285-325)
	13e	Describe any methods used to explore possible causes of heterogeneity among study results (e.g. subgroup analysis, meta-regression).	Methods (pages 6-7, lines 285-325)
	13f	Describe any sensitivity analyses conducted to assess robustness of the synthesized results.	Methods (page 7, lines 327-334)
Reporting bias assessment	14	Describe any methods used to assess risk of bias due to missing results in a synthesis (arising from reporting biases).	Methods (page 7, lines 327-334)
Certainty assessment	15	Describe any methods used to assess certainty (or confidence) in the body of evidence for an outcome.	Methods (page 7, lines 313-315)
**RESULTS**			
Study selection	16a	Describe the results of the search and selection process, from the number of records identified in the search to the number of studies included in the review, ideally using a flow diagram.	Figure 1
	16b	Cite studies that might appear to meet the inclusion criteria, but which were excluded, and explain why they were excluded.	N/A
Study characteristics	17	Cite each included study and present its characteristics.	Supplementary Table 4 (SI pages 18-28)

Section and Topic	Item #	Checklist item	Location where item is reported
Risk of bias in studies	18	Present assessments of risk of bias for each included study.	Data sharing statement (page 11, lines 528-530)
Results of individual studies	19	For all outcomes, present, for each study: (a) summary statistics for each group (where appropriate) and (b) an effect estimate and its precision (e.g. confidence/credible interval), ideally using structured tables or plots.	Data sharing statement (page 11, lines 528-530)
Results of syntheses	20a	For each synthesis, briefly summarise the characteristics and risk of bias among contributing studies.	Results (pages 7-8, lines 347-366)
	20b	Present results of all statistical syntheses conducted. If meta-analysis was done, present for each the summary estimate and its precision (e.g. confidence/credible interval) and measures of statistical heterogeneity. If comparing groups, describe the direction of the effect.	Results (page 8, lines 368-398); Data sharing statement (page 11, lines 531-533)
	20c	Present results of all investigations of possible causes of heterogeneity among study results.	Results (page 8, lines 368-398); Supplementary Information Section 5.2 (SI page 10, lines 325-337)
	20d	Present results of all sensitivity analyses conducted to assess the robustness of the synthesized results.	Supplementary Information Section 5.2 (SI page 10, lines 325-337)
Reporting biases	21	Present assessments of risk of bias due to missing results (arising from reporting biases) for each synthesis assessed.	Results (pages 7-8, lines 347-366)
Certainty of evidence	22	Present assessments of certainty (or confidence) in the body of evidence for each outcome assessed.	Data sharing statement (page 11, lines 531-533)
**DISCUSSION**			
Discussion	23a	Provide a general interpretation of the results in the context of other evidence.	Discussion (page 9, lines 431-456)
	23b	Discuss any limitations of the evidence included in the review.	Discussion (page 10, lines 479-496)
	23c	Discuss any limitations of the review processes used.	Discussion (page 10, lines 498-511)
	23d	Discuss implications of the results for practice, policy, and future research.	Discussion (pages 9-10, lines 431-456 and 513-516)
**OTHER INFORMATION**			
Registration and protocol	24a	Provide registration information for the review, including register name and registration number, or state	This review was not registered.

Section and Topic	Item #	Checklist item	Location where item is reported
		that the review was not registered.	
	24b	Indicate where the review protocol can be accessed, or state that a protocol was not prepared.	No protocol was prepared for this review.
	24c	Describe and explain any amendments to information provided at registration or in the protocol.	No protocol was prepared for this review.
Support	25	Describe sources of financial or non-financial support for the review, and the role of the funders or sponsors in the review.	Acknowledgements (page 11, lines 544-545)
Competing interests	26	Declare any competing interests of review authors.	Declaration of interest (page 11, lines 535-541)
Availability of data, code and other materials	27	Report which of the following are publicly available and where they can be found: template data collection forms; data extracted from included studies; data used for all analyses; analytic code; any other materials used in the review.	Data sharing statement (page 11, lines 527-533)

Supplementary Table 3. PRISMA abstract compliance checklist.

Section and Topic	Item #	Checklist item	Reported (Yes/No)
**TITLE**			
Title	1	Identify the report as a systematic review.	Title (line 2)
**BACKGROUND**			
Objectives	2	Provide an explicit statement of the main objective(s) or question(s) the review addresses.	Background subsection (lines 24-29)
**METHODS**			
Eligibility criteria	3	Specify the inclusion and exclusion criteria for the review.	Methods subsection lines (31-33)
Information sources	4	Specify the information sources (e.g. databases, registers) used to identify studies and the date when each was last searched.	Methods subsection lines (31-33)
Risk of bias	5	Specify the methods used to assess risk of bias in the included studies.	Methods subsection (lines 31-33)
Synthesis of results	6	Specify the methods used to present and synthesise results.	Methods subsection (lines 33-34)
**RESULTS**			
Included studies	7	Give the total number of included studies and participants and summarise relevant characteristics of studies.	Results subsection (line 36)
Synthesis of results	8	Present results for main outcomes, preferably indicating the number of included studies and participants for each. If meta-analysis was done, report the summary estimate and confidence/credible interval. If comparing groups, indicate the direction of the effect (i.e. which group is favoured).	Results subsection (lines 37-42)
**DISCUSSION**			
Limitations of evidence	9	Provide a brief summary of the limitations of the evidence included in the review (e.g. study risk of bias, inconsistency and imprecision).	Interpretation (line 44)
Interpretation	10	Provide a general interpretation of the results and important implications.	Interpretation (lines 45-46)
**OTHER**			
Funding	11	Specify the primary source of funding for the review.	Funding subsection, lines 48-49
Registration	12	Provide the register name and registration number.	This review was not registered.

Supplementary Table 4. Input data sources for final model.

Citation	ISO3	Midpoint Year	Community indicator	Minimum age (years)	Maximum age (years)
Arya LS, Azamy S, Ghani AR, Singh M. Outcome of measles in Afghanistan. Indian pediatrics. 1981 Feb;18(2):112-6.	AFG	1978	0	0.4167	12
Arya LS, Taana I, Tahiri C, Saidali A, Singh M. Spectrum of complications of measles in Afghanistan: a study of 784 cases. The Journal of tropical medicine and hygiene. 1987 Jun 1;90(3):117-22.	AFG	1981	0	0.3333	12
Choudhry VP, Atmar M, Amin I, Aram GN, Ghani R. Effect of protein energy malnutrition on the immediate outcome of measles. The Indian Journal of Pediatrics. 1987 Sep;54(5):717-22.	AFG	1984	0	0	17
Wakeham PF. Severe measles in Afghanistan. Journal of Tropical Pediatrics and Environmental Child Health. 1978;24(2):87-8.	AFG	1971	1	0	99
Chen RT, Weierbach R, Bisoffi Z, Cutts F, Rhodes P, Ramaroson S, Ntembagara C, Bizimana F. A 'post-honeymoon period' measles outbreak in Muyinga sector, Burundi. International journal of epidemiology. 1994 Feb 1;23(1):185-93.	BDI	1988	1	0	5
Kambiré C, Konde MK, Yaméogo A, Tiendrébéogo SR, Ouédraogo RT, Otten Jr MW, Cairns KL, Zuber PL. Measles incidence before and after mass vaccination campaigns in Burkina Faso. Journal of Infectious Diseases. 2003 May 15;187(Supplement_1):S80-5.	BFA	2000	1	0	99
Kidd S, Ouedraogo B, Kambire C, Kambou JL, McLean H, Kutty PK, Ndiaye S, Fall A, Alleman M, Wannemuehler K, Masresha B. Measles outbreak in Burkina Faso, 2009: a case–control study to determine risk factors and estimate vaccine effectiveness. Vaccine. 2012 Jul 13;30(33):5000-8.	BFA	2009	1	0	99
Sahuguède P, Roisin A, Sanou I, Nacro B, Tall F. Epidémie de rougeole au Burkina Faso: 714 cas hospitalisés à l'hôpital de Bobo-Dioulasso: étude des facteurs de risque. InAnnales de pédiatrie (Paris) 1989 (Vol. 36, No. 4, pp. 244-251).	BFA	1986	0	0	18
World Health Organization. Measles mortality reduction in West Africa, 1996-2002. Weekly Epidemiological Record= Relevé épidémiologique hebdomadaire. 2003;78(45):390-2.	BFA	2002	1	0	99
Bhuiya A, Wojtyniak B, D'Souza S, Nahar L, Shaikh K. Measles case fatality among the under-fives: a multivariate analysis of risk factors in a rural area of Bangladesh. Social science & medicine. 1987 Jan 1;24(5):439-43.	BGD	1980	1	0	4
Francisco AD, Fauveau V, Sarder AM, Chowdhury HR, Chakraborty J, Yunus MD. Measles in rural Bangladesh: issues of validation and age distribution. International journal of epidemiology. 1994 Apr 1;23(2):393-9.	BGD	1989	1	0	5
Fauveau V, Chakraborty J, Sarder AM, Khan MA, Koenig MA. Measles among under-9-month-olds in rural Bangladesh: its significance for age at immunization. Bulletin of the World Health organization. 1991;69(1):67.	BGD	1980	1	0.5	99
Koster FT, Curlin GC, Aziz KM, Haque A. Synergistic impact of measles and diarrhoea on nutrition and mortality in Bangladesh. Bulletin of the World Health Organization. 1981;59(6):901.	BGD	1975	1	0	10
Shahid NS, Clauquin P, Shaikh K, Zimicki S. Long-term complication of measles in rural Bangladesh. The Journal of Tropical Medicine and Hygiene. 1983 Apr 1;86(2):77-80.	BGD	1980	1	0	1
World Health Organization. Expanded Programme on Immunization: Public health importance of measles. Weekly Epidemiological Record. 1986;61(12):89-90.	BGD	1984	1	0	99
Tricou V, Pagonendji M, Manengu C, Mutombo J, Mabo RO, Gouandjika-Vasilache I. Measles outbreak in Northern Central African Republic 3 years after the last national immunization campaign. BMC Infectious Diseases. 2013 Dec;13(1):1-6.	CAF	2011	1	0	99
Aiqiang X, Zijian F, Wenbo X, Lixia W, Wanshen G, Qing X, Haijun S, Lee LA, Xiaofeng L. Active Case-Based Surveillance for Measles in China: Lessons Learned from Shandong and Henan Provinces. Journal of Infectious Diseases. 2003 May 15;187(Supplement_1):S258-63.	CHN	2001	1	0	53
Ji Y, Zhang Y, Xu S, Zhu Z, Zuo S, Jiang X, Lu P, Wang C, Liang Y, Zheng H, Liu Y. Measles resurgence associated with continued circulation of genotype H1 viruses in China, 2005. Virology Journal. 2009 Dec;6(1):1-8.	CHN	2005	1	0	99
	CHN	2013	1	0	99

Ma C, Rodewald L, Hao L, Su Q, Zhang Y, Wen N, Fan C, Yang H, Luo H, Wang H, Goodson JL. Progress Toward Measles Elimination—China, January 2013-June 2019. China CDC Weekly. 2019 Dec;1(2):21.	CHN	2014	1	0	99
	CHN	2015	1	0	99
	CHN	2016	1	0	99
	CHN	2017	1	0	99
	CHN	2018	1	0	99
	CHN	2019	1	0	99
World Health Organization. Expanded Programme on Immunization: Measles outbreak= PROGRAMME ÉLARGI DE VACCINATION: Flambée de rougeole. 1990; 65(49): 379-81.	CHN	1988	1	0	99
Ye Y, Wang W, Wang X, Yu H. The clinical epidemiology of pediatric patients with measles from 2000 to 2009 in Shanghai, China. Clinical Pediatrics. 2011 Oct;50(10):916-22.	CHN	2002	0	0	18
	CHN	2007	0	0	18
Yu X, Wang S, Guan J, Gou A, Liu Q, Jin X, Ghildyal R. Analysis of the cause of increased measles incidence in Xinjiang, China in 2004. The Pediatric infectious disease journal. 2007 Jun 1;26(6):513-8.	CHN	2004	1	0	35
Zhang RQ, Li HB, Li FY, Han LX, Xiong YM. Epidemiological characteristics of measles from 2000 to 2014: Results of a measles catch-up vaccination campaign in xianyang, china. Journal of Infection and Public Health. 2017 Sep 1;10(5):624-9.	CHN	2009	1	0	99
Njim T, Agyingi K, Aminde LN, Atunji EF. Trend in mortality from a recent measles outbreak in Cameroon: a retrospective analysis of 223 measles cases in the Benakuma Health District. The Pan African Medical Journal. 2016;23.	CMR	2015	1	0	99
Fischer PR. Measles in Zaire: 1987. Clinical pediatrics. 1988 May;27(5):234-5.	COD	1986	0	0	15
Gignoux E, Polonsky J, Ciglenecki I, Bichet M, Coldiron M, Thuambe Lwiyo E, Akonda I, Serafini M, Porten K. Risk factors for measles mortality and the importance of decentralized case management during an unusually large measles epidemic in eastern Democratic Republic of Congo in 2013. PloS one. 2018 Mar 14;13(3):e0194276.	COD	2012	1	0	99
Grout L, Minetti A, Hurtado N, François G, Fermon F, Chatelain A, Harczi G, de Dieu Ilunga Ngoie J, N'Goran A, Luquero FJ, Grais RF. Measles in Democratic Republic of Congo: an outbreak description from Katanga, 2010–2011. BMC infectious diseases. 2013 Dec;13(1):1-8.	COD	2010	1	0	30
Kasongo Project Team. Influence of measles vaccination on survival pattern of 7-35-month-old children in Kasongo, Zaire. The Lancet. 1981 Apr 4;317(8223):764-7.	COD	1976	1	0	5
Mancini S, Coldiron ME, Ronsse A, Ilunga BK, Porten K, Grais RF. Description of a large measles epidemic in Democratic Republic of Congo, 2010–2013. Conflict and health. 2014 Dec;8(1):1-8.	COD	2010	1	0	99
	COD	2011	1	0	99
	COD	2012	1	0	99
	COD	2013	1	0	99
N'Goran AA, Ilunga N, Coldiron ME, Grais RF, Porten K. Community-based measles mortality surveillance in two districts of Katanga Province, Democratic Republic of Congo. BMC Research Notes. 2013 Dec;6(1):1-3.	COD	2011	1	0	99
	COD	2011	1	0	14
Pan American Health Organization / World Health Organization. Epidemiological Update: Measles. 18 January 2019, Washington, D.C.: PAHO/WHO; 2019	COL	2018	1	0	99
El Shazly MK, Atta HY, Kishk NA. Poliomyelitis, measles and neonatal tetanus: a hospital based epidemiological study. The Journal of the Egyptian Public Health Association. 1997 Jan 1;72(5-6):527-48.	EGY	1994	0	0	60
Belda K, Tegegne AA, Mersha AM, Bayenessagne MG, Hussein I, Bezabeh B. Measles outbreak investigation in Guji zone of Oromia Region, Ethiopia. The Pan African Medical Journal. 2017;27(Suppl 2).	ETH	2015	1	0.25	30
Gutu MA, Bekele A, Seid Y, Woyessa AB. Epidemiology of measles in Oromia region, Ethiopia, 2007-2016. The Pan African Medical Journal. 2020;37.	ETH	2011	1	0	99
Kalil FS, Gemeda DH, Bedaso MH, Wario SK. Measles outbreak investigation in Ginnir district of Bale zone, Oromia region, Southeast Ethiopia, May 2019. Pan African Medical Journal. 2020 May 14;36(1).	ETH	2018	1	0	99
Lindtjørn B. Severe measles in the Gardulla area of southwest Ethiopia. Journal of tropical pediatrics. 1986;32(5):234-9.	ETH	1981	0	0	25
	ETH	1981	1	0	4
Mitiku K, Bedada T, Masresha BG, Kegne W, Nafo-Traoré F, Tesfaye N, Yigzaw A. Progress in measles mortality reduction in Ethiopia, 2002–2009. The Journal of infectious diseases. 2011 Jul 1;204(suppl_1):S232-8.	ETH	2006	1	0.0833	65
Navarro-Colorado C, Mahamud A, Burton A, Haskew C, Maina GK, Wagacha JB, Ahmed JA, Shetty S, Cookson S, Goodson JL,	ETH	2011	0	0	99

Schilperoord M. Measles outbreak response among adolescent and adult Somali refugees displaced by famine in Kenya and Ethiopia, 2011. The Journal of infectious diseases. 2014 Dec 15;210(12):1863-70.					
Poletti P, Parlamento S, Fayyisaa T, Feyyiss R, Lusiani M, Tsegaye A, Segafredo G, Putoto G, Manenti F, Merler S. The hidden burden of measles in Ethiopia: how distance to hospital shapes the disease mortality rate. BMC medicine. 2018 Dec;16(1):1-2.	ETH	2015	0	0	65
Tariku MK, Misikir SW. Measles outbreak investigation in Artuma Fursi Woreda, Oromia zone, Amhara region, Ethiopia, 2018: a case control study. BMC Research Notes. 2019 Dec;12(1):1-6.	ETH	2018	1	0	99
Güris D, Auerbach SB, Vitek C, Maes E, McCready J, Durand M, Cruz K, Iohp K, Haddock R, Rota J, Rota P. Measles outbreaks in Micronesia, 1991 to 1994. The Pediatric infectious disease journal. 1998 Jan 1;17(1):33-9.	FSM	1992	1	0	19
Bosu WK, Odoom S, Deiter P, Essel-Ahun M. Epidemiology of measles in the Central Region of Ghana: a five-year case review in three district hospitals. East African medical journal. 2003;80(6):312-7.	GHA	1998	0	0	60
Commey JO, Dekyem P. Measles in southern Ghana: 1985-1993. West African Journal of Medicine. 1994 Oct 1;13(4):223-6.	GHA	1989	0	0.25	12
Commey JO, Richardson JE. Measles in Ghana—1973–1982. Annals of tropical paediatrics. 1984 Sep 1;4(3):189-94.	GHA	1977	0	0.3333	14
Dollimore N, Cutts F, Binka FN, Ross DA, Morris SS, George Smith P. Measles incidence, case fatality, and delayed mortality in children with or without vitamin A supplementation in rural Ghana. American journal of epidemiology. 1997 Oct 15;146(8):646-54.	GHA	1990	1	0	7
Hull H, Williams PJ, Oldfield F. Measles mortality and vaccine efficacy in rural West Africa. The Lancet. 1983 Apr 30;321(8331):972-5.	GMB	1981	1	0.25	10
Hull HF. Increased measles mortality in households with multiple cases in the Gambia, 1981. Clinical Infectious Diseases. 1988 Mar 1;10(2):463-7.	GMB	1981	1	0	10
Lamb WH. Epidemic measles in a highly immunized rural West African (Gambian) village. Clinical Infectious Diseases. 1988 Mar 1;10(2):457-62.	GMB	1984	1	0	99
Williams PJ. Effect of measles immunization on child mortality in rural Gambia. Journal of Biosocial Science. 1989;21(S10):95-104.	GMB	1984	1	0	15
Williams PJ, Hull HF. Status of measles in the Gambia, 1981. Reviews of infectious diseases. 1983 May 1;5(3):391-4.	GMB	1981	1	0.25	10
Aaby P, Bukh J, Lisse IM, da Silva MC. Further community studies on the role of overcrowding and intensive exposure on measles mortality. Clinical Infectious Diseases. 1988 Mar 1;10(2):474-7.	GNB	1981	1	0.42	18
Aaby P, Bukh J, Lisse IM, Smits AJ. Introduction of measles into a highly immunised West African community: the role of health care institutions. Journal of Epidemiology & Community Health. 1985 Jun 1;39(2):113-6.	GNB	1981	1	0	99
Aaby P, Bukh J, Lisse IM, Smits AJ. Measles mortality, state of nutrition, and family structure: a community study from Guinea-Bissau. Journal of infectious diseases. 1983 Apr 1;147(4):693-701.	GNB	1979	1	0	18
Aaby P, Knudsen K, Jensen TG, Thirup J, Poulsen A, Sodemann M, da Silva MC, Whittle H. Measles incidence, vaccine efficacy, and mortality in two urban African areas with high vaccination coverage. Journal of infectious diseases. 1990 Nov 1;162(5):1043-8.	GNB	1983	1	0	1
	GNB	1986	1	0	1
Aaby P, Martins C, Bale C, Garly ML, Rodrigues A, Biai S, Lisse IM, Whittle H, Benn CS. Sex differences in the effect of vaccines on the risk of hospitalization due to measles in Guinea-bissau. The Pediatric infectious disease journal. 2010 Apr 1;29(4):324-8.	GNB	2003	0	0.5	5
Aaby P, Bukh J, Lisse IM, da Silva MC. Decline in measles mortality: nutrition, age at infection, or exposure?. Br Med J (Clin Res Ed). 1988 Apr 30;296(6631):1225-8.	GNB	1979	1	0	17
	GNB	1982	1	0	17
Aaby P, Bukh J, Lisse IM, Smits AJ, Gomes J, Fernandes MA, Indi F, Soares M. Determinants of measles mortality in a rural area of Guinea-Bissau: crowding, age, and malnutrition. Journal of tropical pediatrics. 1984 Jun 1;30(3):164-8.	GNB	1980	1	0	20
Martins CL, Garly ML, Balé C, Rodrigues A, Ravn H, Whittle HC, Lisse IM, Aaby P. Protective efficacy of standard Edmonston-Zagreb measles vaccination in infants aged 4.5 months: interim analysis of a randomised clinical trial. Bmj. 2008 Jul 24;337.	GNB	2003	1	0.375	0.375
Aaby P, Bukh J, Lisse IM, Smits AJ. Overcrowding and intensive exposure as determinants of measles mortality. American journal of epidemiology. 1984 Jul 1;120(1):49-63.	GNB	1979	1	0	5

Tollefson JE, Hospedales CJ, White FM. Epidemiological indicators and the epidemiology of measles in the English-speaking Caribbean and Suriname. The West Indian Medical Journal. 1992 Mar 1;41(1):2-7.	GUY	1988	1	0	99
World Health Organization. Measles surveillance: Measles in the Caribbean prior to the elimination campaign. Weekly Epidemiological Record= Relevé épidémiologique hebdomadaire. 1991;66(40):291-4.	GUY	1988	1	0	99
Lubis CP, Pasaribu S, Lubis MM. Morbidity and mortality of tetanus, diphtheria and morbilli (measles) cases (a 1982-1985 study at the Child Health Department, Dr. Pirngadi Hospital, Medan). The Journal of the Singapore Paediatric Society. 1987 Jan 1;29:66-72.	IDN	1982	0	0	15
	IDN	1983	0	0	15
	IDN	1984	0	0	15
	IDN	1985	0	0	15
	IDN	1986	0	0	15
Munir M, Mustadjab I, Wulur FH. Measles and its problems. A clinical analysis of hospitalized patients under 5 years of age. Paediatrica Indonesiana. 1982 Apr 30;22(3-4):49-64.	IDN	1980	0	0	5
Rangkuti SM, Nazir N, Sutanto AH, Lubis A, Siregar H. Measles morbidity and mortality in the Department of Child Health, Dr Pirngadi General Hospital, Medan, in 1973-1977. Paediatrica Indonesiana. 1980;20(7/8):139-44.	IDN	1975	0	0	12
Samsi TK, Ruspandji T, Susanto I, Gunawan K. Risk factors for severe measles. The Southeast Asian Journal of Tropical Medicine and Public Health. 1992 Sep 1;23(3):497-503.	IDN	1984	0	0.5	18
Agarwal DK, Dutta A, Arora RR, Nair MR. Natural history of measles in rural and urban community of Varanasi. Journal of Communicable Diseases. 1976;8(4):289-98.	IND	1974	1	0	18
Ananthakrishnan S, Srinivasan S, Mahadevan S. Vitamin A and post measles complications. Indian pediatrics. 1993;30(4):520-2.	IND	1989	0	0.5	18
Basa S, Das RR, Khan JA. Root-Cause Analytical Survey for Measles Outbreak: Vaccination or Vaccine?-A Study From Madhepura District, Bihar, India. Journal of Clinical and Diagnostic Research: JCDR. 2015 Jun;9(6):SC04.	IND	2008	1	0	12
Basu RN. Measles vaccine—feasiblity, efficacy and complication rates in a multicentric study. The Indian Journal of Pediatrics. 1984 Mar;51(2):139-43.	IND	1982	1	0.75	2
Bhatia R. Measles outbreak in village Tophema in Nagaland. Journal of communicable diseases. 1985;17(2):185-9.	IND	1983	1	1	12
Bose AS, Jafari H, Sosler S, Narula AP, Kulkarni VM, Ramamurty N, Oommen J, Jadi RS, Banpel RV, Henao-Restrepo AM. Case based measles surveillance in Pune: evidence to guide current and future measles control and elimination efforts in India. PLoS One. 2014 Oct 7;9(10):e108786.	IND	2010	1	0	99
Chand P, Rai RN, Chawla U, Tripathi KC, Datta KK. Epidemiology of measles--a thirteen years prospective study in a village. The Journal of Communicable Diseases. 1989 Sep 1;21(3):190-9.	IND	1980	1	0	14
Cherian T, Joseph A, John TJ. Low antibody response in infants with measles and children with subclinical measles virus infection. The Journal of Tropical Medicine and Hygiene. 1984 Feb 1;87(1):27-31.	IND	1979	1	0	5
Dhanoa JA, Cowan BE. Measles in the community-a study in non-hospitalised young children in Punjab. Journal of Tropical Pediatrics. 1982;28(2):59-61.	IND	1980	1	0	2
Gupta BP, Sharma S. Measles outbreak in a rural area near Shimla. Indian Journal of Community Medicine. 2006 Apr 1;31(2):106.	IND	2004	1	0	14
Gupta BP, Swami HM, Bhardwaj AK, Vaidya NK, Sharma CD, Kaushal RK. An outbreak of measles in a remote tribal area of Himachal Pradesh. Indian J Comm Health. 1989;5:25-8.	IND	1986	1	0	14
Jajoo UN, Chhabra S, Gupta OP, Jain AP. Measles epidemic in a rural community near Sevagram (Vidarbha). Indian journal of public health. 1984;28(4):204-7.	IND	1982	1	0	10
John S, Sanghi S, Prasad S, Bose A, George K. Two doses of measles vaccine: are some states in India ready for it?. Journal of tropical pediatrics. 2009 Aug 1;55(4):253-6.	IND	1999	1	0	9
	IND	2006	1	0	18
John TJ, Joseph A, George TI, Radhakrishnan J, Singh RP, George K. Epidemiology and prevention of measles in rural south India. Indian Journal of Medical Research. 1980;72(August):153-8.	IND	1977	1	0	9
Kalita J, Mani VE, Bhoi SK, Misra UK. Spectrum and outcome of acute infectious encephalitis/encephalopathy in an intensive care unit from India. QJM: An International Journal of Medicine. 2017 Mar 1;110(3):141-8.	IND	2013	0	2	85

Lakhanpal U, Rathore MS. Epidemiology of measles in rural area of Punjab. Journal of communicable diseases. 1986;18(3):185-8.	IND	1983	1	0	14
Lobo J, Reddaiah VP, Kapoor SK, Nath LM. Epidemiology of measles in a rural community. The Indian Journal of Pediatrics. 1987 Mar;54(2):261-5.	IND	1984	1	0	9
Mangal N, Shah K, Sitaraman S. Epidemiological study of measles in urban (slum) area of Jaipur. Indian pediatrics. 1990;27(11):1216-7.	IND	1985	1	0	9
Mishra A, Mishra S, Jain P, Bhadoriya RS, Mishra R, Lahariya C. Measles related complications and the role of vitamin A supplementation. The Indian Journal of Pediatrics. 2008 Sep;75(9):887-90.	IND	2004	1	0	18
Murhekar MV, Ahmad M, Shukla H, Abhishek K, Perry RT, Bose AS, Shimpi R, Kumar A, Kaliaperumal K, Sethi R, Selvaraj V. Measles case fatality rate in Bihar, India, 2011–12. Plos one. 2014 May 13;9(5):e96668.	IND	2011	1	0	99
Murhekar MV, Hutin YJ, Ramakrishnan R, Ramachandran V, Biswas AK, Das PK, Gupta SN, Maji D, Martolia HC, Mohan A, Gupte MD. The heterogeneity of measles epidemiology in India: implications for improving control measures. The Journal of infectious diseases. 2011 Jul 1;204(suppl_1):S421-6.	IND	2004	1	0	14
	IND	2005	1	0	14
	IND	2006	1	0	14
NARAIN JP, KHARE S, Rana SR, Banerjee KB. Epidemic measles in an isolated unvaccinated population, India. International journal of epidemiology. 1989 Dec 1;18(4):952-8.	IND	1986	0	0	99
	IND	1986	1	0	99
Phaneendra Rao RS, Kumari J, RAO K, Narasimham VL. Measles in a rural Community. Journal of communicable diseases. 1988;20(2):131-5.	IND	1983	1	0	5
Raoot A, Dewan DK, Dubey AP, Batra RK, Seth S. Measles outbreak in high risk areas of Delhi: epidemiological investigation and laboratory confirmation. The Indian Journal of Pediatrics. 2016 Mar;83(3):200-8.	IND	2014	1	0	99
Ratho RK, Mishra B, Singh T, Rao P, Kumar R. Measles outbreak in a migrant population. Indian J Pediatr. 2005;72(10):893-4.	IND	2003	1	1	25
Ray SK, Mallik S, Munsi AK, Mitra SP, Baur B, Kumar S. Epidemiological study of measles in slum areas of Kolkata. The Indian Journal of Pediatrics. 2004 Jul;71(7):583-6.	IND	1999	1	0	17
Risbud AR, Prasad SR, Mehendale SM, Mawar N, Shaikh N, Umrani UB, Bedekar SS, Banerjee K. Measles outbreak in a tribal population of Thane district, Maharashtra. Indian pediatrics. 1994 May 1;31(5):543-51.	IND	1991	1	0	10
	IND	1992	1	0	10
Satpathy SK, Chakraborty AK. Epidemiological study of measles in Singur, West Bengal. The Journal of Communicable Diseases. 1990 Mar 1;22(1):23-6.	IND	1986	1	0	99
Sharma MK, Bhatia V, Swami H. Outbreak of measles amongst vaccinated children in a slum of Chandigarh. Indian Journal of Medical Sciences. 2004;58(2):47.	IND	2003	1	0	14
Sharma RS. An epidemiological study of measles epidemic in district Bhilwara, Rajasthan. Journal of communicable diseases. 1988;20(4):301-11.	IND	1984	1	0	13
Sharma RS, Kaushic VK, Johri SP, Ray SN. An epidemiological investigation of measles outbreak in Alwar-Rajasthan. Journal of communicable diseases. 1984;16(4):299-303.	IND	1982	1	0	5
Singh J, Kumar A, Rai RN, Khare S, Jain DC, Bhatia R, Datta KK. Widespread outbreaks of measles in rural Uttar Pradesh, India, 1996: high risk areas and groups. Indian pediatrics. 1999 Mar 1;36(3):249-56.	IND	1996	1	0	99
Singh J, Sharma RS, Verghese T. Measles mortality in India: a review of community based studies. The Journal of Communicable Diseases. 1994 Dec 1;26(4):203-14.	IND	1980	1	0	99
	IND	1985	1	0	99
	IND	1992	1	0	14
Swami SS, Chandra S, Dudani IU, Sharma R, Mathur MM. Epidemiology of measles in Western Rajasthan. Journal of communicable diseases. 1987;19(4):370-2.	IND	1980	1	0	14
Thakur JS, Ratho RK, Bhatia SP, Grover R, Issaivanan M, Ahmed B, Parmar V, Swami HM. Measles outbreak in a Periurban area of Chandigarh: need for improving vaccine coverage and strengthening surveillance. The Indian Journal of Pediatrics. 2002 Jan;69(1):33-7.	IND	1998	1	0	99
Vasudev JP, Nandan D, Chandra R, Srivastava BC. Post measles complications in a rural population. Journal of communicable diseases. 1983;15(4):249-52.	IND	1980	1	0	17
Janghorbani M, Parizi MH, Ghorbani K. Measles epidemics in Kerman city, Iran. Public Health. 1993 Mar 1;107(2):79-87.	IRN	1990	1	0.4167	35
Tollefson JE, Hospedales CJ, White FM. Epidemiological indicators and the epidemiology of measles in the English-speaking Caribbean and Suriname. The West Indian Medical Journal. 1992 Mar 1;41(1):2-7.	JAM	1989	1	0	99

World Health Organization. Measles surveillance: Measles in the Caribbean prior to the elimination campaign. Weekly Epidemiological Record= Relevé épidémiologique hebdomadaire. 1991;66(40):291-4.	JAM	1989	1	0	99
Alwar AJ. The effect of protein energy malnutrition on morbidity and mortality due to measles at Kenyatta National Hospital, Nairobi (Kenya). East African medical journal. 1992 Aug 1;69(8):415-8.	KEN	1983	0	0.0385	20
Borus PK, Cumberland P, Sonoiya S, Kombich J, Tukei PM, Cutts FT. Measles trends and vaccine effectiveness in Nairobi, Kenya. East African medical journal. 2003;80(7):361-4.	KEN	1998	0	0	99
Burström B, Abby P, Mutie DM. Validity of measles mortality data using hospital registers and community surveys. International journal of epidemiology. 1995 Jun 1;24(3):625-9.	KEN	1986	0	0	17
	KEN	1986	1	0	17
	KEN	1988	0	0	17
	KEN	1988	1	0	17
Burström B, Aaby P, Mutie DM. Child mortality impact of a measles outbreak in a partially vaccinated rural African community. Scandinavian journal of infectious diseases. 1993 Jan 1;25(6):763-9.	KEN	1987	1	0	99
Burström B, Aaby P, Mutie DM, Kimani G, Bjerregaard P. Severe measles outbreak in western Kenya. East African medical journal. 1992 Aug 1;69(8):419-23.	KEN	1985	1	0	17
Kisangau N, Sergon K, Ibrahim Y, Yonga F, Langat D, Nzunza R, Borus P, Galgalo T, Lowther SA. Progress towards elimination of measles in Kenya, 2003-2016. Pan African Medical Journal. 2018 Sep 28;31(1).	KEN	2009	1	0	99
Mahamud A, Burton A, Hassan M, Ahmed JA, Wagacha JB, Spiegel P, Haskew C, Eidex RB, Shetty S, Cookson S, Navarro-Colorado C. Risk factors for measles mortality among hospitalized Somali refugees displaced by famine, Kenya, 2011. Clinical Infectious Diseases. 2013 Oct 15;57(8):e160-6.	KEN	2011	0	0	99
Menge I, Esamai F, Van Reken D, Anabwani G. Paediatric morbidity and mortality at the Eldoret District Hospital, Kenya. East African medical journal. 1995;72(3):165-9.	KEN	1993	0	0	17
Muller AS, Voorhoeve AM, T'mannetje W, Schulpen TW. The impact of measles in a rural area of Kenya. East African medical journal. 1977;54(7):364-72.	KEN	1975	1	0	14
	KEN	1976	1	0	14
Navarro-Colorado C, Mahamud A, Burton A, Haskew C, Maina GK, Wagacha JB, Ahmed JA, Shetty S, Cookson S, Goodson JL, Schilperoord M. Measles outbreak response among adolescent and adult Somali refugees displaced by famine in Kenya and Ethiopia, 2011. The Journal of infectious diseases. 2014 Dec 15;210(12):1863-70.	KEN	2011	0	0	99
Centers for Disease Control and Prevention (CDC. Accelerated measles control--Cambodia, 1999-2002. MMWR. Morbidity and mortality weekly report. 2003 Jan 10;52(1):4-6.	KHM	1999	1	0	99
Oum S, Chandramohan D, Cairncross S. Community-based surveillance: a pilot study from rural Cambodia. Tropical medicine & international health. 2005 Jul;10(7):689-97.	KHM	2001	1	0	14
Kuroiwa C, Vongphrachanh P, Xayyavong P, Southalack K, Hashizume M, Nakamura S. Measles epidemiology and outbreak investigation using IgM test in Laos. Journal of Epidemiology. 2001;11(6):255-62.	LAO	1994	1	0	99
	LAO	1995	1	0	99
	LAO	1996	1	0	99
	LAO	1997	1	0	99
	LAO	1998	1	0	99
	LAO	1999	1	0	99
	LAO	2000	1	0	99
Nagbe T, Williams GS, Rude JM, Flomo S, Yeabah T, Fallah M, Skrip L, Agbo C, Mahmoud N, Okeibunor JC, Yealue K. Lessons learned from detecting and responding to recurrent measles outbreak in Liberia post Ebola-Epidemic 2016-2017. The Pan African Medical Journal. 2019;33(Suppl 2).	LBR	2016	1	0	99
Lamabadusuriya SP, Jayantha UK. An outbreak of measles in the Southern Province. The Ceylon Medical Journal. 1992 Jun 1;37(2):46-8.	LKA	1989	0	0	17
Premaratna R, Luke N, Perera H, Gunathilake M, Amarasena P, Chandrasena TG. Sporadic cases of adult measles: a research article. BMC research notes. 2017 Dec;10(1):1-6.	LKA	2015	0	18	99
Puvimanasinghe JP, Arambepola CK, Abeysinghe NM, Rajapaksa LC, Kulatilaka TA. Measles outbreak in Sri Lanka, 1999–2000. Journal of Infectious Diseases. 2003 May 15;187(Supplement_1):S241-5.	LKA	1999	1	0	99
World Health Organization (WHO). Expanded programme on immunization. Public health importance of measles: Sri Lanka. Wkly Epidemiol Rec. 1985;60(13): 95-7.	LKA	1983	1	0	99

Nimpa MM, Andrianirinarison JC, Sodjinou VD, Douba A, Masembe YV, Randriatsarafara F, Ramamonjisoa CB, Rafalimanantsoa AS, Razafindratsimandresy R, Ndiaye CF, Rakotonirina J. Measles outbreak in 2018-2019, Madagascar: epidemiology and public health implications. The Pan African Medical Journal. 2020;35.	MDG	2018	1	0	99
Hyde TB, Dayan GH, Langidrik JR, Nandy R, Edwards R, Briand K, Konelios M, Marin M, Nguyen HQ, Khalifah AP, O'leary MJ. Measles outbreak in the Republic of the Marshall Islands, 2003. International journal of epidemiology. 2006 Apr 1;35(2):299-306.	MHL	2003	1	0	49
McIntyre RC, Preblud SR, Polloi AN, Korean MA. Measles and measles vaccine efficacy in a remote island population. Bulletin of the World Health Organization. 1982;60(5):767.	MHL	1977	1	0	30
World Health Organization. Measles mortality reduction in West Africa, 1996-2002. Weekly Epidemiological Record= Relevé épidémiologique hebdomadaire. 2003;78(45):390-2.	MLI	2002	1	0	99
Chin J, Thaung UM. The unchanging epidemiology and toll of measles in Burma. Bulletin of the World Health Organization. 1985;63(3):551.	MMR	1983	0	0	9
	MMR	1983	1	0	9
Khin M, Win S, Aye SS. The impact of national measles immunization programme on measles admissions to the major children's hospital in Yangon. Tropical doctor. 1994 Jul;24(3):141-3.	MMR	1985	0	0	12
	MMR	1989	0	0	12
Lee CT, Hagan JE, Jantsansengee B, Tumurbaatar OE, Altanchimeg S, Yadamsuren B, Demberelsuren S, Tserendorj C, Munkhtogoo O, Badarch D, Gunregjav N. Increase in infant measles deaths during a nationwide measles outbreak—Mongolia, 2015–2016. The Journal of infectious diseases. 2019 Oct 22;220(11):1771-9.	MNG	2015	1	0	0.9999
	MNG	2015	1	0	99
Orsoo O, Saw YM, Sereenen E, Yadamsuren B, Byambaa A, Kariya T, Yamamoto E, Hamajima N. Epidemiological characteristics and trends of a Nationwide measles outbreak in Mongolia, 2015–2016. BMC Public Health. 2019 Dec;19(1):1-0.	MNG	2015	1	0	99
Cliff J, Simango A, Augusto O, Van der Paal L, Biellik R. Failure of targeted urban supplemental measles vaccination campaigns (1997–1999) to prevent measles epidemics in Mozambique (1998–2001). Journal of Infectious Diseases. 2003 May 15;187(Supplement_1):S51-7.	MOZ	1993	1	0	99
	MOZ	1998	1	0	99
Mandomando I, Naniche D, Pasetti MF, Cuberos L, Sanz S, Vallès X, Sigauque B, Macete E, Nhalungo D, Kotloff KL, Levine MM. Assessment of the epidemiology and burden of measles in Southern Mozambique. The American journal of tropical medicine and hygiene. 2011 Jul 7;85(1):146.	MOZ	2002	0	0	18
Boushab BM, Savadogo M, Sow MS, Dao S. Epidemiological, clinical, and prognostic study of the measles in the Aioun regional hospital in Mauritania. Médecine et Santé Tropicales. 2015 Apr 1;25(2):180-3.	MRT	2011	0	1	33
Minetti A, Kagoli M, Katsulukuta A, Huerga H, Featherstone A, Chiotcha H, Noel D, Bopp C, Sury L, Fricke R, Iscla M. Lessons and challenges for measles control from unexpected large outbreak, Malawi. Emerging infectious diseases. 2013 Feb;19(2):202.	MWI	2010	1	0	99
Courtright P, Fine D, Broadhead RL, Misoya L, Vagh M. Abnormal vitamin A cytology and mortality in infants aged 9 months and less with measles. Annals of tropical paediatrics. 2002 Sep 1;22(3):239-43.	MWI	1992	0	0.25	0.8333
Yamaguchi S, Dunga A, Broadhead RL, Brabin BJ. Epidemiology of measles in Blantyre, Malawi: analyses of passive surveillance data from 1996 to 1998. Epidemiology & Infection. 2002 Oct;129(2):361-9.	MWI	1997	1	0	99
Grais RF, Dubray C, Gerstl S, Guthmann JP, Djibo A, Nargaye KD, Coker J, Alberti KP, Cochet A, Ihekweazu C, Nathan N. Unacceptably high mortality related to measles epidemics in Niger, Nigeria, and Chad. PLoS medicine. 2007 Jan;4(1):e16.	NER	2003	1	0	99
Kaninda AV, Legros D, Jataou IM, Malfait P, Maisonneuve M, Paquet C, Moren A. Measles vaccine effectiveness in standard and early immunization strategies, Niger, 1995. The Pediatric infectious disease journal. 1998 Nov 1;17(11):1034-9.	NER	1995	1	0	4
Malfait P, Jataou IM, Jollet MC, Margot A, DE BENOIST AC, Moren A. Measles epidemic in the urban community of Niamey: transmission patterns, vaccine efficacy and immunization strategies, Niger, 1990 to 1991. The Pediatric infectious disease journal. 1994 Jan 1;13(1):38-44.	NER	1990	0	0.5	4.9167
Nandy R, Handzel T, Zaneidou M, Biey J, Coddy RZ, Perry R, Strebel P, Cairns L. Case-fatality rate during a measles outbreak in eastern Niger in 2003. Clinical infectious diseases. 2006 Feb 1;42(3):322-8.	NER	2003	1	0	99
World Health Organization. Expanded Programme on Immunization: High measles case-fatality rates during an outbreak in a rural area. Weekly Epidemiological Record. 1993;68(20):142-5.	NER	1991	1	0	99

Adedoyin MA. The pattern of measles in Ilorin. West African Journal of Medicine. 1990 Apr 1;9(2):103-7.	NGA	1982	0	0	5
	NGA	1983	0	0	5
	NGA	1984	0	0	5
Ahmed PA, Babaniyi IB, Otuneye AT. Review of childhood measles admissions at the National Hospital, Abuja. Nigerian Journal of Clinical Practice. 2010;13(4).	NGA	2003	0	0.5833	10
Babalola OJ, Ibrahim IN, Kusfa IU, Gidado S, Nguku P, Olayinka A, Abubakar A. Measles outbreak investigation in an urban slum of Kaduna Metropolis, Kaduna State, Nigeria, March 2015. Pan African Medical Journal. 2019 Mar 28;32(1).	NGA	2015	1	0	99
Bamgboye EA, Familusi JB. Mortality pattern at a children's emergency ward, University College Hospital, Ibadan, Nigeria. African journal of medicine and medical sciences. 1990 Jun 1;19(2):127-32.	NGA	1982	0	0	17
Byass P, Adedeji MD, Mongdem JG, Zwandor AC, Brew-Graves SH, Clements CJ. Assessment and possible control of endemic measles in urban Nigeria. Journal of Public Health. 1995 Jun 1;17(2):140-5.	NGA	1992	1	0	4
Ekanem EE, Ochigbo SO, Kwagtsule JU. Unprecedented decline in measles morbidity and mortality in Calabar, south-eastern Nigeria. Tropical doctor. 2000 Oct;30(4):207-9.	NGA	1994	0	0	11
Fagbule D, Orifunmishe F. Measles and childhood mortality in semi-urban Nigeria. African journal of medicine and medical sciences. 1988 Sep 1;17(3):181-5.	NGA	1984	0	0.25	7
Faruk AS, Adebowale AS, Balogun MS, Taiwo L, Adeoye O, Mamuda S, Waziri NE. Temporal trend of measles cases and impact of vaccination on mortality in Jigawa State, Nigeria, 2013-2017: a secondary data analysis. The Pan African Medical Journal. 2020;35(Suppl 1).	NGA	2015	1	0	99
Fatiregun AA, Adebowale AS, Fagbamigbe AF. Epidemiology of measles in Southwest Nigeria: an analysis of measles case-based surveillance data from 2007 to 2012. Transactions of the Royal Society of Tropical Medicine and Hygiene. 2014 Mar 1;108(3):133-40.	NGA	2009	1	0	99
Fatiregun AA, Olowookere SA, Abubakar O, Aderibigbe A. Small-scale outbreak of measles in the Irewole local government area of Osun State in Nigeria. Asian Pacific Journal of Tropical Medicine. 2009;2(6):33-6.	NGA	2008	1	0.6667	14
Fetuga MB, Njakanna OF, Ongunfowora OB. A ten-year study of measles admissions in a Nigerian teaching hospital. Nigerian Journal of Clinical Practice. 2007 Sep 14;10(1):41-6.	NGA	1999	0	0.3333	12
Grais RF, Dubray C, Gerstl S, Guthmann JP, Djibo A, Nargaye KD, Coker J, Alberti KP, Cochet A, Ihekweazu C, Nathan N. Unacceptably high mortality related to measles epidemics in Niger, Nigeria, and Chad. PLoS medicine. 2007 Jan;4(1):e16.	NGA	2004	1	0	99
Ibia EO, Asindi AA. Measles in Nigerian children in Calabar during the era of expanded programme on immunization. Tropical and geographical medicine. 1990 Jul 1;42(3):226-32.	NGA	1985	0	0.375	11
Ibrahim BS, Usman R, Mohammed Y, Datti Z, Okunromade O, Abubakar AA, Nguku PM. Burden of measles in Nigeria: a five-year review of casebased surveillance data, 2012-2016. The Pan African Medical Journal. 2019;32(Suppl 1).	NGA	2012	1	0	99
	NGA	2013	1	0	99
	NGA	2014	1	0	99
	NGA	2015	1	0	99
	NGA	2016	1	0	99
Lagunju IA, Orimadegun AE, Oyedemi DG. Measles in Ibadan: a continuous scourge. African journal of medicine and medical sciences. 2005 Dec 1;34(4):383-7.	NGA	2002	0	0.3333	10
Olugbade OT, Adeyemi AS, Adeoti AH, Ilesanmi OS, Gidado SO, Waziri NE, Aworh MK. Measles outbreaks and Supplemental Immunization Activities (SIAs): the Gwagwalada experience, Abuja 2015. The Pan African Medical Journal. 2019;32(Suppl 1).	NGA	2015	1	0	33
Weldegebriel GG, Gasasira A, Harvey P, Masresha B, Goodson JL, Pate MA, Abanida E, Chevez A. Measles resurgence following a nationwide measles vaccination campaign in Nigeria, 2005–2008. The Journal of infectious diseases. 2011 Jul 1;204(suppl_1):S226-31.	NGA	2008	1	0	99
Joshi AB, Luman ET, Nandy R, Subedi BK, Liyanage JB, Wierzba TF. Measles deaths in Nepal: estimating the national case-fatality ratio. Bulletin of the World Health Organization. 2009;87:456-65.	NPL	2004	1	0	99
Sitaula S, Awasthi GR, Thapa JB, Ramaiya A. Measles outbreak among unvaccinated children in Bajura. Journal of Nepal Medical Association. 2010 Oct 1;50(180).	NPL	2010	1	0.75	25
Aurangzeb B, Fatmee A, Waris R, Haider N, Berjees A, Raza SH. Risk factors for mortality among admitted children with complications of	PAK	2015	0	0.75	18

measles in Pakistan–an observational. Journal of the Pakistan Medical Association. 2020 Nov 3:1-4.					
Aurangzeb B, Nisar YB, Hazir T, Burki F, Hassan M. Clinical outcome in children hospitalized with complicated measles. J Coll Physicians Surg Pak. 2005 Sep 1;15(9):547-1.	PAK	2003	0	0.5	12
Murray M, Rasmussen Z. Measles outbreak in a northern Pakistani village: epidemiology and vaccine effectiveness. American journal of epidemiology. 2000 Apr 15;151(8):811-9.	PAK	1990	1	0	13
Anis-ur-Rehman ST, Idris M. Clinical outcome in measles patients hospitalized with complications. J Ayub Med Coll Abbottabad. 2008;20(2):14-6.	PAK	2004	0	0.5	12
Rehman IU, Bukhsh A, Khan TM. Measles in Pakistan: time to make steps towards eradication. Travel medicine and infectious disease. 2017 Jul 1;18:67-9.	PAK	2012	1	0	18
	PAK	2013	1	0	18
Saeed A, Butt ZA, Malik T. Investigation of measles outbreak in a district of Balochistan province, Pakistan. Journal of Ayub Medical College Abbottabad. 2015 Dec 15;27(4):900-3.	PAK	2014	1	0	11
Sniadack DH, Moscoso B, Aguilar R, Heath J, Bellini W, Chiu MC. Measles epidemiology and outbreak response immunization in a rural community in Peru. Bulletin of the World Health Organization. 1999;77(7):545.	PER	1993	1	0	40
Almoradie-Javonillo I, Javonillo T. Profile of a measles epidemic in a remote Philippine barrio. Journal of the Philippine Medical Association. 1984 Apr 30;60(3).	PHL	1983	1	0	14
Bronzwaer SL, De Groot CJ. Risk factors for a complicated disease course in children with measles admitted to a Philippine university hospital. Nederlands Tijdschrift Voor Geneeskunde. 1997 Dec 1;141(51):2492-5.	PHL	1994	0	0	15
Benjamin AL, Dramoi V. Outbreak of measles in the National Capital District, Papua New Guinea in 2001. Papua and New Guinea Medical Journal. 2002 Sep 1;45(3-4):178-84.	PNG	2001	0	0	17
Coakley KJ, Coakley CA, Spooner V, Smith TA, Javati A, Kajoi M. A review of measles admissions and deaths in the paediatric ward of Goroka Base Hospital during 1989. Papua and New Guinea Medical Journal. 1991 Mar 1;34(1):6-12.	PNG	1989	0	0	17
Mgone JM, Mgone CS, Duke TR, Frank DA, Yeka WI. Control measures and the outcome of the measles epidemic of 1999 in the Eastern Highlands Province. Papua and New Guinea medical journal. 2000 Mar 1;43(1-2):91-7.	PNG	1999	0	0	13
Centers for Disease Control and Prevention (CDC. Emergency measles control activities--Darfur, Sudan, 2004. MMWR. Morbidity and mortality weekly report. 2004 Oct 1;53(38):897-9.	SDN	2004	1	0	99
Coronado F, Musa N, Ahmed El Tayeb ES, Haithami S, Dabbagh A, Mahoney F, Nandy R, Cairns L. Retrospective measles outbreak investigation: Sudan, 2004. Journal of tropical pediatrics. 2006 Oct 1;52(5):329-34.	SDN	2003	1	0	99
El Karim O, Salih MA. Morbidity and mortality from measles in an urban community of the Sudan. Annals of Tropical Medicine & Parasitology. 1981 Apr 1;75(2):227-30.	SDN	1975	0	0	18
Ibrahim SA, Mustafa O, Mukhtar MM, Saleh EA, El Mubarak HS, Abdallah A, El-Hassan AM, Osterhaus AD, Groen J, De Swart RL, Zijlstra EE. Measles in suburban Khartoum: an epidemiological and clinical study. Tropical Medicine & International Health. 2002 May;7(5):442-9.	SDN	1998	1	0.4167	14
Sulaiman AA, Elmadhoun WM, Noor SK, Almobarak AO, Bushara SO, Osman MM, Awadalla H, Ahmed MH. An outbreak of measles in gold miners in River Nile State, Sudan, 2011. Eastern Mediterranean Health Journal. 2020 Feb 1;26(2).	SDN	2011	1	0	99
Aaby P, Whittle H, Cisse B, Samb B, Jensen H, Simondon F. The frailty hypothesis revisited: mainly weak children die of measles. Vaccine. 2001 Dec 12;20(5-6):949-53.	SEN	1984	1	0	99
	SEN	1988	1	0	99
	SEN	1992	1	0	99
Aaby P. Influence of cross-sex transmission on measles mortality in rural Senegal. The Lancet. 1992 Aug 15;340(8816):388-91.	SEN	1984	1	0	18
Cisse B, Aaby P, Simondon F, Samb B, Soumare M, Whittle H. Role of schools in the transmission of measles in rural Senegal: implications for measles control in developing countries. American journal of epidemiology. 1999 Feb 15;149(4):295-301.	SEN	1994	1	0.4167	30

Pison G, Bonneuil N. Increased risk of measles mortality for children with siblings among the Fula Bande, Senegal. Clinical Infectious Diseases. 1988 Mar 1;10(2):468-70.	SEN	1985	1	0	11
Pison G. Dynamique d'une population traditionelle: les Peul Bande (Senegal oriental). Institut national d'etudes demographiques. Cahier no. 99. Paris: Presses Universitaires de France, 1982. 1982.	SEN	1977	1	0	19
Samb B, Aaby P, Whittle H, Seck AM, Simondon F. Decline in measles case fatality ratio after the introduction of measles immunization in rural Senegal. American journal of epidemiology. 1997 Jan 1;145(1):51-7.	SEN	1984	1	0	99
	SEN	1988	1	0	99
Sesay T, Denisiuk O, Zachariah R. Paediatric morbidity and mortality in Sierra Leone. Have things changed after the 2014/2015 Ebola outbreak?. F1000Research. 2019;8.	SLE	2013	0	0	5
	SLE	2014	0	0	5
	SLE	2016	0	0	5
Sugerman DE, Fall A, Guigui MT, N'dolie M, Balogun T, Wurie A, Goodson JL. Preplanned national measles vaccination campaign at the beginning of a measles outbreak—Sierra Leone, 2009–2010. The Journal of infectious diseases. 2011 Jul 1;204(suppl_1):S260-9.	SLE	2009	1	0	99
World Health Organization (WHO). Expanded Programme on Immunization: Epidemiology of Measles in a Rural Community. Wkly Epidemiol Rec. 1980;55(12): 85-7.	SOM	1978	1	0	99
Grais RF, Dubray C, Gerstl S, Guthmann JP, Djibo A, Nargaye KD, Coker J, Alberti KP, Cochet A, Ihekweazu C, Nathan N. Unacceptably high mortality related to measles epidemics in Niger, Nigeria, and Chad. PLoS medicine. 2007 Jan;4(1):e16.	TCD	2003	1	0	99
Ndikuyeze A, Cook A, Cutts FT, Bennett S. Priorities in global measles control: report of an outbreak in N'Djamena, Chad. Epidemiology & Infection. 1995 Oct;115(2):309-14.	TCD	1990	1	0	5
World Health Organization. Measles mortality reduction in West Africa, 1996-2002. Weekly Epidemiological Record= Relevé épidémiologique hebdomadaire. 2003;78(45):390-2.	TGO	2002	1	0	99
Ariyasriwatana C, Kalayanarooj S. Severity of measles: a study at the Queen Sirikit National Institute of Child Health. Journal of the Medical Association of Thailand. 2004 Jun 1;87(6):581.	THA	2000	0	0	14
World Health Organization (WHO). Expanded programme on immunization. Measles outbreak among the hill tribes. Wkly Epidemiol Rec. 1985; 60(11): 79.	THA	1984	1	0.5833	25
Burgess W, Mduma B, Josephson GV. Measles in Mbeya, Tanzania-1981-1983. Journal of tropical pediatrics. 1986;32(4):148-53.	TZA	1982	0	0	99
Mafigiri R, Nsubuga F, Ario AR. Risk factors for measles death: Kyegegwa District, western Uganda, February–September, 2015. BMC Infectious Diseases. 2017 Dec;17(1):1-7.	UGA	2015	1	0.3	36
Weeks RM, Barenzi JF, Wayira JR. A low-cost, community-based measles outbreak investigation with follow-up action. Bulletin of the World Health Organization. 1992;70(3):317.	UGA	1990	1	0.4167	12
Espinosa L, Mirinaviciute G. Health crisis in Venezuela: status of communicable diseases and implications for the European Union and European Economic Area, May 2019. Eurosurveillance. 2019 May 30;24(22):1900308.	VEN	2017	1	0	99
	VEN	2018	1	0	99
Pan American Health Organization / World Health Organization. Epidemiological Update: Measles. 18 January 2019, Washington, D.C.: PAHO/WHO; 2019	VEN	2017	1	0	99
Karim SA, Karim QA, Dilraj A, Chamane M. Unsustainability of a measles immunisation campaign-rise in measles incidence within 2 years of the campaign. South African Medical Journal. 1993;83(5).	ZAF	1989	0	0	99
	ZAF	1990	0	0	99
	ZAF	1991	0	0	99
Coetzee S, Morrow BM, Argent AC. Measles in a S outh A frican paediatric intensive care unit: Again!. Journal of Paediatrics and Child Health. 2014 May;50(5):379-85.	ZAF	2010	0	0.42	0.75
Dramowski A, Aucamp M, Bekker A, Mehtar S. Infectious disease exposures and outbreaks at a South African neonatal unit with review of neonatal outbreak epidemiology in Africa. International Journal of Infectious Diseases. 2017 Apr 1;57:79-85.	ZAF	2010	0	0	0.9999
Gibson, IHN, Carmichael, TR & Kustner HG. Measles notifications-the first year. South African Medical Journal. 1982 Jan 1;61(3):84-8.	ZAF	1979	1	0	99
Hussey GD, Klein M. Routine high-dose vitamin A therapy for children hospitalized with measles. Journal of tropical pediatrics. 1993 Dec 1;39(6):342-5.	ZAF	1985	0	0	18
Jeena PM, Wesley AG, Coovadia HM. Infectious diseases at the paediatric isolation units of Clairwood and King Edward VIII hospitals, Durban. South African Medical Journal. 1998;88(7).	ZAF	1985	0	0	18
	ZAF	1986	0	0	18
	ZAF	1987	0	0	18

	ZAF	1988	0	0	18
	ZAF	1989	0	0	18
	ZAF	1990	0	0	18
	ZAF	1991	0	0	18
	ZAF	1992	0	0	18
	ZAF	1993	0	0	18
	ZAF	1994	0	0	18
	ZAF	1995	0	0	18
	ZAF	1996	0	0	18
Le Roux DM, Le Roux SM, Nuttall JJ, Eley BS. South African measles outbreak 2009-2010 as experienced by a paediatric hospital. South African Medical Journal. 2012;102(9):760-4.	ZAF	2009	0	0	18
Loening WE, Coovadia HM. Age-specific occurrence rates of measles in urban, peri-urban, and rural environments: implications for time of vaccination. The Lancet. 1983 Aug 6;322(8345):324-6.	ZAF	1980	0	0	17
McMorrow ML, Gebremedhin G, Van den Heever J, Kezaala R, Harris BN, Nandy R. Measles outbreak in South Africa, 2003-2005. South African Medical Journal. 2009;99(5).	ZAF	2004	1	0	99
	ZAF	2005	1	0	99
Uzicanin A, Eggers R, Webb E, Harris B, Durrheim D, Ogunbanjo G, Isaacs V, Hawkridge A, Biellik R, Strebel P. Impact of the 1996–1997 supplementary measles vaccination campaigns in South Africa. International journal of epidemiology. 2002 Oct 1;31(5):968-76.	ZAF	1989	1	0	99
Centers for Disease Control and Prevention (CDC. Measles incidence before and after supplementary vaccination activities--Lusaka, Zambia, 1996-2000. MMWR. Morbidity and mortality weekly report. 2001 Jun 22;50(24):513-6.	ZMB	1996	0	0	99
	ZMB	1997	0	0	99
	ZMB	1998	0	0	99
	ZMB	1999	0	0	99
Moss WJ, Monze M, Ryon JJ, Quinn TC, Griffin DE, Cutts F. Prospective Study of Measles in Hospitalized, Human Immunodeficiency Virus (HIV)—Infected and HIV—Uninfected Children in Zambia. Clinical infectious diseases. 2002 Jul 15;35(2):189-96.	ZMB	1999	0	0	18
Oshitani H, Mpabalwani M, Kasolo F, Mizuta K, Luo NP, Bhat GJ, Suzuki H, Numazaki Y. Measles infection in hospitalized children in Lusaka, Zambia. Annals of tropical paediatrics. 1995 Jun 1;15(2):167-72.	ZMB	1992	0	0	15
Rolfe M. Measles immunization in the Zambian Copperbelt: cause for concern. Transactions of the Royal Society of Tropical Medicine and Hygiene. 1982 Jan 1;76(4):529-30.	ZMB	1980	1	0	4
Kambarami RA, Nathoo KJ, Nkrumah FK, Pirie DJ. Measles epidemic in Harare, Zimbabwe, despite high measles immunization coverage rates. Bulletin of the World Health Organization. 1991;69(2):213.	ZWE	1988	1	0.1154	30
Marufu T, Siziya S, Tshimanga M, Murugasampillay S, Mason E, Manyume B. Factors associated with measles complications in Gweru, Zimbabwe. East African medical journal. 2001;78(3):135-8.	ZWE	1984	1	0	99
Nsungu M. Measles vaccination status, delay in recognizing measles outbreaks and outbreak outcome. The Central African journal of medicine. 1995 Nov 1;41(11):336-9.	ZWE	1994	0	0	24
Uyirwoth GP. Measles in Mashonaland Central Province: Zimbabwe. East African medical journal. 1993 Jul 1;70(7):455-9.	ZWE	1987	0	0	99
	ZWE	1988	0	0	99
	ZWE	1989	0	0	99

Supplementary Table 5. Proxy covariate sets used for analysis.

Original covariate	Proxy covariate used
Level of health care available	Gross domestic product per capita
Educational attainment	Maternal educations
Mean household size	Proportion living in urban setting<br>Total fertility rate
Surrounding conflict	Mortality rate due to war and terrorism

Supplementary Table 6. Covariate set values by country in 2019.

ISO3	Vitamin A deficiency prevalence	Mortality rate due to war and terrorism	HIV prevalence	Maternal education	Total fertility rate	GDP per capita	Under-5 mortality rate	MCV1 coverage	Proportion living in urban setting	Wasting prevalence	Measles incidence
**AFG**	0.1404	0.0006	0.0003	3.1116	4.3210	9992.3903	60.1000	0.6400	0.2575	0.0638	0.0018
**AGO**	0.1336	0.0000	0.0133	6.4430	5.4420	2612.3470	74.2000	0.5100	0.6618	0.0528	0.0002
**ALB**	0.1462	0.0000	0.0000	10.5183	1.5970	4543.3865	9.7000	0.9500	0.6123	0.0391	0.0008
**ARM**	0.0042	0.0000	0.0004	12.3754	1.7580	4758.5575	11.5000	0.9500	0.6322	0.0337	0.0011
**AZE**	0.0598	0.0000	0.0002	11.6242	1.8100	4758.5575	20.4000	0.9500	0.5603	0.0498	0.0000
**BDI**	0.1587	0.0000	0.0077	4.1233	5.3210	278.2026	56.6000	0.9200	0.1337	0.0553	0.0107
**BEN**	0.2289	0.0000	0.0061	3.9160	4.7670	1201.5614	88.4000	0.6800	0.4786	0.0739	0.0043
**BFA**	0.2216	0.0000	0.0045	1.9187	5.1090	738.2189	87.8000	0.8800	0.2998	0.1257	0.0012
**BGD**	0.0824	0.0000	0.0002	6.5529	2.0110	1581.5675	30.7000	0.9700	0.3741	0.1159	0.0012
**BIH**	0.1323	0.0000	0.0000	11.4522	1.2540	4758.5575	5.9000	0.9500	0.4863	0.0244	0.0021
**BLR**	0.0092	0.0000	0.0017	13.3724	1.3820	4758.5575	3.1000	0.9500	0.7904	0.0139	0.0008
**BLZ**	0.0366	0.0000	0.0045	9.0432	2.2740	4712.8401	12.3000	0.9600	0.4587	0.0321	0.0009
**BOL**	0.0469	0.0000	0.0023	9.3314	2.6880	3317.3709	26.3000	0.7900	0.6977	0.0155	0.0017
**BTN**	0.1027	0.0000	0.0023	3.9344	1.9540	3238.0605	28.6000	0.9700	0.4161	0.0395	0.0009
**CAF**	0.2080	0.0001	0.0244	4.2122	4.6450	418.7217	106.6000	0.4100	0.4177	0.0958	0.0286
**CHN**	0.0501	0.0000	0.0004	10.3300	1.6960	10155.4929	7.9000	0.9900	0.6031	0.0164	0.0002
**CIV**	0.1888	0.0000	0.0194	4.4157	4.5930	2327.7454	80.3000	0.7300	0.5124	0.0606	0.0006
**CMR**	0.2070	0.0000	0.0230	7.8759	4.5060	1449.2775	74.7000	0.6000	0.5697	0.0485	0.0068
**COD**	0.2071	0.0000	0.0046	8.0764	5.8190	512.5863	83.8000	0.6500	0.4505	0.0850	0.0549
**COG**	0.2218	0.0000	0.0197	9.7790	4.3740	1793.0281	52.5000	0.7300	0.6737	0.0581	0.0026
**COL**	0.0449	0.0000	0.0028	9.6068	1.7890	6384.5358	13.6000	0.9500	0.8110	0.0102	0.0001
**COM**	0.1889	0.0000	0.0000	7.4271	4.1380	1284.3523	63.5000	0.9000	0.2916	0.0928	0.0044
**CPV**	0.0069	0.0000	0.0057	6.6588	2.2420	3482.4485	14.9000	0.9800	0.6620	0.0197	0.0021
**CUB**	0.0205	0.0000	0.0021	11.8680	1.6020	8031.0354	5.2000	0.9900	0.7711	0.0141	0.0004
**DJI**	0.0541	0.0000	0.0125	4.9588	2.6760	1898.1839	57.8000	0.8300	0.7792	0.1899	0.0006
**DZA**	0.0428	0.0000	0.0007	8.9292	2.9880	4115.3955	23.3000	0.8000	0.7319	0.0446	0.0004
**ECU**	0.0802	0.0000	0.0023	10.7738	2.4030	5853.8131	13.4000	0.8300	0.6399	0.0196	0.0000
**EGY**	0.0344	0.0000	0.0001	11.3261	3.2800	3964.9871	20.1000	0.9500	0.4273	0.0477	0.0001
**ERI**	0.1807	0.0000	0.0039	4.8723	3.9970	1898.1839	40.6000	0.7580	0.4473	0.1201	0.0008
**ETH**	0.2584	0.0000	0.0069	3.7181	4.1460	799.7951	50.8000	0.5800	0.2123	0.0968	0.0177
**FJI**	0.0773	0.0000	0.0005	11.6860	2.7540	5869.0211	26.9000	0.9600	0.5675	0.0470	0.0010
**FSM**	0.1739	0.0000	0.0011	9.6625	3.0100	2921.1456	25.4000	0.7800	0.2281	0.0452	0.0055
**GEO**	0.0614	0.0000	0.0012	13.2363	2.0550	4773.4233	9.5000	0.9500	0.5904	0.0091	0.0013
**GHA**	0.2136	0.0000	0.0119	8.6681	3.8160	2053.5867	46.4000	0.9200	0.5671	0.0649	0.0028

**GIN**	0.1931	0.0000	0.0093	3.3759	4.6250	945.5074	98.0000	0.4700	0.3650	0.0873	0.0016
**GMB**	0.2132	0.0000	0.0134	5.1083	5.1540	714.5421	51.2000	0.8500	0.6193	0.0806	0.0001
**GNB**	0.2644	0.0000	0.0217	3.6300	4.4020	650.0694	79.6000	0.7900	0.4378	0.0628	0.0002
**GTM**	0.0625	0.0000	0.0009	6.3516	2.8220	4254.0352	24.5000	0.9000	0.5144	0.0105	0.0005
**GUY**	0.0576	0.0000	0.0075	10.6060	2.4400	6478.2877	29.3000	0.9800	0.2669	0.0595	0.0004
**HND**	0.0527	0.0000	0.0004	6.8878	2.4270	2499.4928	16.8000	0.8900	0.5773	0.0128	0.0003
**HTI**	0.1300	0.0000	0.0164	6.3011	2.8870	1373.8831	62.2000	0.6500	0.5619	0.0598	0.0000
**IDN**	0.1468	0.0000	0.0004	9.8638	2.2880	3877.4246	23.8000	0.8800	0.5599	0.1031	0.0014
**IND**	0.1941	0.0000	0.0014	7.5065	2.2020	1965.5393	34.4000	0.9500	0.3447	0.1679	0.0026
**IRN**	0.0233	0.0000	0.0002	10.0437	2.1460	5308.9199	13.4000	0.9900	0.7539	0.0359	0.0001
**IRQ**	0.0777	0.0000	0.0000	10.1375	3.5970	5132.7011	26.1000	0.8200	0.7068	0.0477	0.0004
**JAM**	0.0320	0.0000	0.0044	12.1379	1.9650	5065.3749	13.7000	0.9400	0.5599	0.0264	0.0002
**JOR**	0.0838	0.0000	0.0000	13.1249	2.6910	4133.5498	15.5000	0.8700	0.9120	0.0213	0.0001
**KEN**	0.2275	0.0000	0.0322	8.8740	3.4230	1602.7884	43.0000	0.8900	0.2751	0.0404	0.0003
**KGZ**	0.0682	0.0000	0.0009	12.2232	3.3000	1226.8245	18.3000	0.9500	0.3659	0.0244	0.0006
**KHM**	0.0858	0.0000	0.0045	5.5688	2.4780	5300.9050	26.6000	0.8400	0.2381	0.0830	0.0052
**KIR**	0.1922	0.0000	0.0001	10.1285	3.5300	1505.1552	51.2000	0.9400	0.5484	0.0360	0.0063
**LAO**	0.1423	0.0000	0.0015	5.5657	2.6260	2579.2537	45.7000	0.8300	0.3565	0.0681	0.0003
**LBR**	0.1736	0.0000	0.0088	6.3476	4.2470	1898.1839	80.4000	0.7580	0.5162	0.0478	0.0041
**LKA**	0.0873	0.0000	0.0001	10.8837	2.1880	4228.1492	7.2000	0.9900	0.1859	0.1363	0.0007
**LSO**	0.2039	0.0000	0.1844	9.1413	3.1080	1126.8438	90.9000	0.9000	0.2859	0.0317	0.0019
**MAR**	0.1116	0.0000	0.0010	5.9305	2.3820	3044.9063	19.5000	0.9900	0.6299	0.0282	0.0012
**MDA**	0.0139	0.0000	0.0019	13.6890	1.2690	4758.5575	14.8000	0.9500	0.4273	0.0217	0.0009
**MDG**	0.1778	0.0000	0.0019	7.7934	4.0260	488.9137	51.9000	0.5500	0.3786	0.1056	0.0014
**MHL**	0.1666	0.0000	0.0011	9.6251	2.6453	3612.6023	31.6000	0.8500	0.7742	0.0013	0.0014
**MKD**	0.1158	0.0000	0.0000	11.9490	1.3400	4758.5575	6.8000	0.9500	0.5821	0.0299	0.0013
**MLI**	0.2093	0.0001	0.0062	2.4365	5.7850	815.3791	94.2000	0.7000	0.4314	0.0949	0.0026
**MMR**	0.0891	0.0000	0.0041	6.7895	2.1380	1548.4566	45.2000	0.8400	0.3085	0.0597	0.0016
**MNG**	0.1118	0.0000	0.0001	9.9933	2.8670	4394.9881	16.0000	0.9800	0.6854	0.0122	0.0001
**MOZ**	0.1973	0.0000	0.0691	4.6535	4.7830	598.8137	72.9000	0.8700	0.3653	0.0419	0.0001
**MRT**	0.1653	0.0000	0.0001	6.8802	4.5030	1620.9967	73.0000	0.7500	0.5451	0.1034	0.0001
**MWI**	0.1935	0.0000	0.0623	7.2392	4.1270	401.3927	40.6000	0.9200	0.1717	0.0412	0.0001
**NAM**	0.0746	0.0000	0.0850	9.3781	3.3440	4504.6174	41.9000	0.7580	0.5104	0.0636	0.0011
**NER**	0.2241	0.0000	0.0016	1.5164	6.8240	523.8842	80.3000	0.7900	0.1652	0.1421	0.0167
**NGA**	0.1805	0.0000	0.0102	7.6417	5.3170	2502.6523	116.9000	0.5700	0.5116	0.1068	0.0110
**NIC**	0.0282	0.0000	0.0010	7.6677	2.3770	1982.6286	16.6000	0.9900	0.5876	0.0125	0.0007
**NPL**	0.1110	0.0000	0.0017	5.1189	1.8760	1069.7891	29.3000	0.9200	0.2015	0.0820	0.0035
**PAK**	0.1313	0.0000	0.0012	5.6196	3.4540	1497.9868	67.3000	0.8100	0.3691	0.1305	0.0115
**PER**	0.0722	0.0000	0.0020	9.9372	2.2330	6613.8764	13.3000	0.8500	0.7810	0.0050	0.0005

**PHL**	0.1621	0.0000	0.0024	11.4090	2.5260	3664.7907	27.1000	0.7500	0.4715	0.0702	0.0050
**PNG**	0.0627	0.0000	0.0051	3.9336	3.5200	2816.7188	45.3000	0.3700	0.1325	0.1173	0.0003
**PRK**	0.0868	0.0000	0.0008	11.5175	1.8960	5300.9050	17.3000	0.9800	0.6213	0.0377	0.0004
**PRY**	0.1386	0.0000	0.0011	10.0671	2.4050	5774.1662	19.5000	0.8700	0.6188	0.0118	0.0001
**RWA**	0.1339	0.0000	0.0197	5.5620	3.9900	885.6381	41.9000	0.9600	0.1731	0.0256	0.0157
**SDN**	0.0854	0.0000	0.0026	7.9076	4.3470	1969.1201	58.4000	0.9000	0.3494	0.1290	0.0027
**SEN**	0.2062	0.0000	0.0037	3.7067	4.5560	1384.3970	39.7000	0.8900	0.4765	0.0664	0.0005
**SLB**	0.1085	0.0000	0.0010	5.8482	4.3610	2289.5289	20.0000	0.8100	0.2421	0.0775	0.0007
**SLE**	0.1814	0.0000	0.0088	4.3162	4.1690	649.7603	111.9000	0.7580	0.4248	0.0684	0.0021
**SLV**	0.0942	0.0000	0.0019	7.9788	2.0210	3993.5291	13.3000	0.8200	0.7275	0.0145	0.0005
**SOM**	0.2289	0.0001	0.0021	3.7816	5.9780	1898.1839	118.3000	0.4600	0.4555	0.1367	0.0481
**SRB**	0.1402	0.0000	0.0001	13.4823	1.8695	4758.5575	5.7000	0.9500	0.5626	0.0297	0.0009
**STP**	0.2164	0.0000	0.0001	7.6839	4.2670	1898.1839	17.0000	0.9500	0.7360	0.0452	0.0037
**SWZ**	0.1338	0.0000	0.1904	9.0000	2.9580	3833.2468	48.0000	0.8100	0.2398	0.0157	0.0001
**SYR**	0.0688	0.0005	0.0000	11.3038	2.7710	969.2613	22.2000	0.6500	0.5482	0.1087	0.0006
**TCD**	0.2270	0.0000	0.0075	2.3917	5.6490	660.0699	113.5000	0.4100	0.2328	0.1458	0.0092
**TGO**	0.2067	0.0000	0.0138	5.5127	4.2590	630.7905	66.5000	0.7500	0.4225	0.0561	0.0004
**THA**	0.0729	0.0000	0.0069	10.6705	1.5140	6612.2274	9.0000	0.9600	0.5069	0.0543	0.0007
**TJK**	0.1168	0.0000	0.0005	10.5055	3.5560	1174.0817	33.3000	0.9500	0.2731	0.0691	0.0001
**TKM**	0.0613	0.0000	0.0006	10.1778	2.7400	7692.5787	42.4000	0.9500	0.5205	0.0445	0.0002
**TLS**	0.1079	0.0000	0.0020	7.1972	3.9430	5300.9050	43.7000	0.9007	0.3095	0.1996	0.0003
**TON**	0.1027	0.0000	0.0012	10.9248	3.5180	4652.5886	11.7000	0.9900	0.2311	0.0465	0.0011
**TUN**	0.0131	0.0000	0.0004	9.6807	2.1740	4208.0662	16.9000	0.9800	0.6925	0.0232	0.0002
**TUV**	0.1023	0.0000	0.0011	9.9877	2.6453	5300.9050	22.7000	0.9600	0.6322	0.0284	0.0029
**TZA**	0.1373	0.0000	0.0332	7.1880	4.8320	1071.3501	50.5000	0.8800	0.3450	0.0389	0.0001
**UGA**	0.1485	0.0000	0.0366	7.6903	4.8240	894.5204	45.3000	0.8700	0.2436	0.0411	0.0028
**UKR**	0.0129	0.0000	0.0059	13.9316	1.2280	2425.6345	8.4000	0.9500	0.6947	0.0777	0.0072
**UZB**	0.0532	0.0000	0.0006	12.6813	2.7850	3161.4154	14.8000	0.9500	0.5043	0.0315	0.0003
**VEN**	0.0702	0.0000	0.0033	9.5810	2.2500	13111.5940	24.2000	0.9300	0.8824	0.0341	0.0001
**VNM**	0.0708	0.0000	0.0023	10.0258	2.0500	3250.5675	21.1000	0.9500	0.3663	0.0567	0.0007
**VUT**	0.1474	0.0000	0.0010	7.6942	3.7440	2881.7490	25.6000	0.8000	0.2539	0.0459	0.0003
**WSM**	0.1119	0.0000	0.0010	10.8043	3.8300	4504.9193	17.4000	0.9600	0.1806	0.0414	0.0043
**YEM**	0.1624	0.0004	0.0006	4.7560	3.7000	9992.3903	61.5000	0.6700	0.3727	0.1579	0.0008
**ZAF**	0.1268	0.0000	0.0639	10.9565	2.3810	6125.7353	33.0000	0.8300	0.6686	0.0333	0.0000
**ZMB**	0.1795	0.0000	0.0724	8.9401	4.5590	1348.7384	64.1000	0.9300	0.4407	0.0451	0.0001
**ZWE**	0.1489	0.0000	0.0876	9.7120	3.5310	1414.8291	54.2000	0.8500	0.3221	0.0356	0.0001

Supplementary Table 7. Second step model fitted meta-regression coefficients.

Variable	Coefficient values	p-values
Intercept	-2.1084	<0.0001
Community indicator	-1.4597	<0.0001
Incidence	0.2513	<0.0001
Mortality rate due to war and terrorism	0	1.0
Maternal education	-0.6915	<0.0001
GDP per capita	0	1.0
HIV prevalence	0	1.0
MCV1 coverage	-0.1581	<0.0001
Total fertility	0	1.0
Under 5 mortality rate	0.1140	<0.0001
Proportion living in urban setting	0.4161	<0.0001
Vitamin A deficiency prevalence	0.0652	<0.0001
Wasting prevalence	0	1.0

Supplementary Table 8. In-sample validation metrics from first stage decomposition analysis.

IS	Model 0	Model 1.A	Model 1.B	Model 1.C
Correlation	0.29	0.2785	0.2776	0.3415
RMSE	0.0616	0.0629	0.0631	0.0598
Mean Error	0.0104	0.0218	0.0202	0.0075
Mean Abs. Error	0.0356	0.0354	0.0358	0.0354

Supplementary Table 9. Out-of-sample validation metrics from first stage decomposition analysis.

OOS	Model 0	Model 1.A	Model 1.B	Model 1.C
Correlation	0.2897	0.2783	0.2775	0.3376
RMSE	0.0624	0.0635	0.0641	0.0608
Mean Error	0.0100	0.0200	0.0182	0.0052
Mean Abs. Error	0.0357	0.0357	0.0362	0.0364

Supplementary Table 10. In-sample validation metrics from second stage decomposition analysis.

IS	Model 0	Model 1	Model 2
Correlation	0.29	0.3415	0.3929
RMSE	0.0616	0.0598	0.0591
Mean Error	0.0104	0.0075	-0.0010
Mean Abs. Error	0.0356	0.0354	0.0349

Supplementary Table 11. Out-of-sample validation metrics from first and second stage decomposition analysis.

OOS	Model 0	Model 1	Model 2
Correlation	0.2897	0.3376	0.3833
RMSE	0.0624	0.0608	0.0616
Mean Error	0.0100	0.0052	-0.0022
Mean Abs. Error	0.0357	0.0364	0.0362

Supplementary Table 12. In-sample (IS) and out-of-sample (OOS) validation metrics from final model using age split input data for comparison.

	IS	OOS
Correlation	0.5002	0.4517
RMSE	0.0451	0.0270
Mean Error	0.0035	0.0011
Mean Abs. Error	0.0175	0.0110

Supplementary Table 13. In-sample (IS) and out-of-sample (OOS) validation metrics from final model using original (pre-age split) data for comparison.

	IS	OOS
Correlation	0.4667	0.2754
RMSE	0.0934	0.0820
Mean Error	0.0156	0.0027
Mean Abs. Error	0.0343	0.0325

Supplementary Table 14. Age-specific CFR by region in 2019.

Region	Age Group	Setting	CFR	Lower 95% UI	Upper 95% UI
**Central Europe, Eastern Europe, and Central Asia**	under 1-year-olds	Hospital	3.06%	2.71%	3.41%
		Community	0.72%	0.65%	0.79%
	1- to 4-year-olds	Hospital	1.51%	1.34%	1.68%
		Community	0.35%	0.32%	0.39%
	5- to 9-year-olds	Hospital	1.18%	1.04%	1.32%
		Community	0.27%	0.25%	0.30%
	10- to 14-year-olds	Hospital	0.97%	0.86%	1.09%
		Community	0.22%	0.20%	0.25%
	15-year-olds and older	Hospital	0.39%	0.33%	0.46%
		Community	0.09%	0.08%	0.10%
**Latin America and Caribbean**	under 1-year-olds	Hospital	6.35%	5.72%	7.01%
		Community	1.54%	1.40%	1.67%
	1- to 4-year-olds	Hospital	3.46%	3.11%	3.80%
		Community	0.82%	0.75%	0.89%
	5- to 9-year-olds	Hospital	2.49%	2.25%	2.74%
		Community	0.58%	0.53%	0.63%
	10- to 14-year-olds	Hospital	1.95%	1.75%	2.15%
		Community	0.46%	0.41%	0.50%
	15-year-olds and older	Hospital	0.99%	0.85%	1.13%
		Community	0.23%	0.20%	0.26%
**North Africa and Middle East**	under 1-year-olds	Hospital	9.76%	9.00%	10.55%
		Community	2.45%	2.27%	2.61%
	1- to 4-year-olds	Hospital	4.84%	4.53%	5.19%
		Community	1.17%	1.10%	1.23%
	5- to 9-year-olds	Hospital	2.75%	2.55%	2.95%
		Community	0.65%	0.62%	0.68%
	10- to 14-year-olds	Hospital	2.12%	1.97%	2.29%
		Community	0.50%	0.47%	0.52%
	15-year-olds and older	Hospital	1.10%	0.97%	1.24%
		Community	0.26%	0.23%	0.29%
**South Asia**	under 1-year-olds	Hospital	7.20%	6.67%	7.81%
		Community	1.77%	1.66%	1.88%
	1- to 4-year-olds	Hospital	3.91%	3.67%	4.19%
		Community	0.93%	0.88%	0.97%
	5- to 9-year-olds	Hospital	3.01%	2.82%	3.22%
		Community	0.71%	0.68%	0.75%

	10- to 14-year-olds	Hospital	2.57%	2.39%	2.76%
		Community	0.60%	0.57%	0.64%
	15-year-olds and older	Hospital	2.05%	1.86%	2.25%
		Community	0.48%	0.44%	0.52%
**Southeast Asia, East Asia, and Oceania**	under 1-year-olds	Hospital	4.22%	3.87%	4.61%
		Community	1.01%	0.93%	1.08%
	1- to 4-year-olds	Hospital	2.41%	2.23%	2.60%
		Community	0.57%	0.53%	0.60%
	5- to 9-year-olds	Hospital	1.78%	1.66%	1.92%
		Community	0.42%	0.39%	0.44%
	10- to 14-year-olds	Hospital	1.49%	1.38%	1.60%
		Community	0.35%	0.33%	0.37%
	15-year-olds and older	Hospital	0.70%	0.63%	0.79%
		Community	0.16%	0.15%	0.18%
**Sub-Saharan Africa**	under 1-year-olds	Hospital	13.91%	13.06%	14.72%
		Community	3.65%	3.49%	3.81%
	1- to 4-year-olds	Hospital	8.18%	7.79%	8.62%
		Community	2.03%	1.97%	2.10%
	5- to 9-year-olds	Hospital	5.30%	5.04%	5.59%
		Community	1.28%	1.23%	1.33%
	10- to 14-year-olds	Hospital	3.82%	3.58%	4.06%
		Community	0.91%	0.87%	0.95%
	15-year-olds and older	Hospital	2.88%	2.65%	3.12%
		Community	0.68%	0.63%	0.73%

Section 7. Supplementary Figures

Supplementary Figure 1. Overview of modeling process.

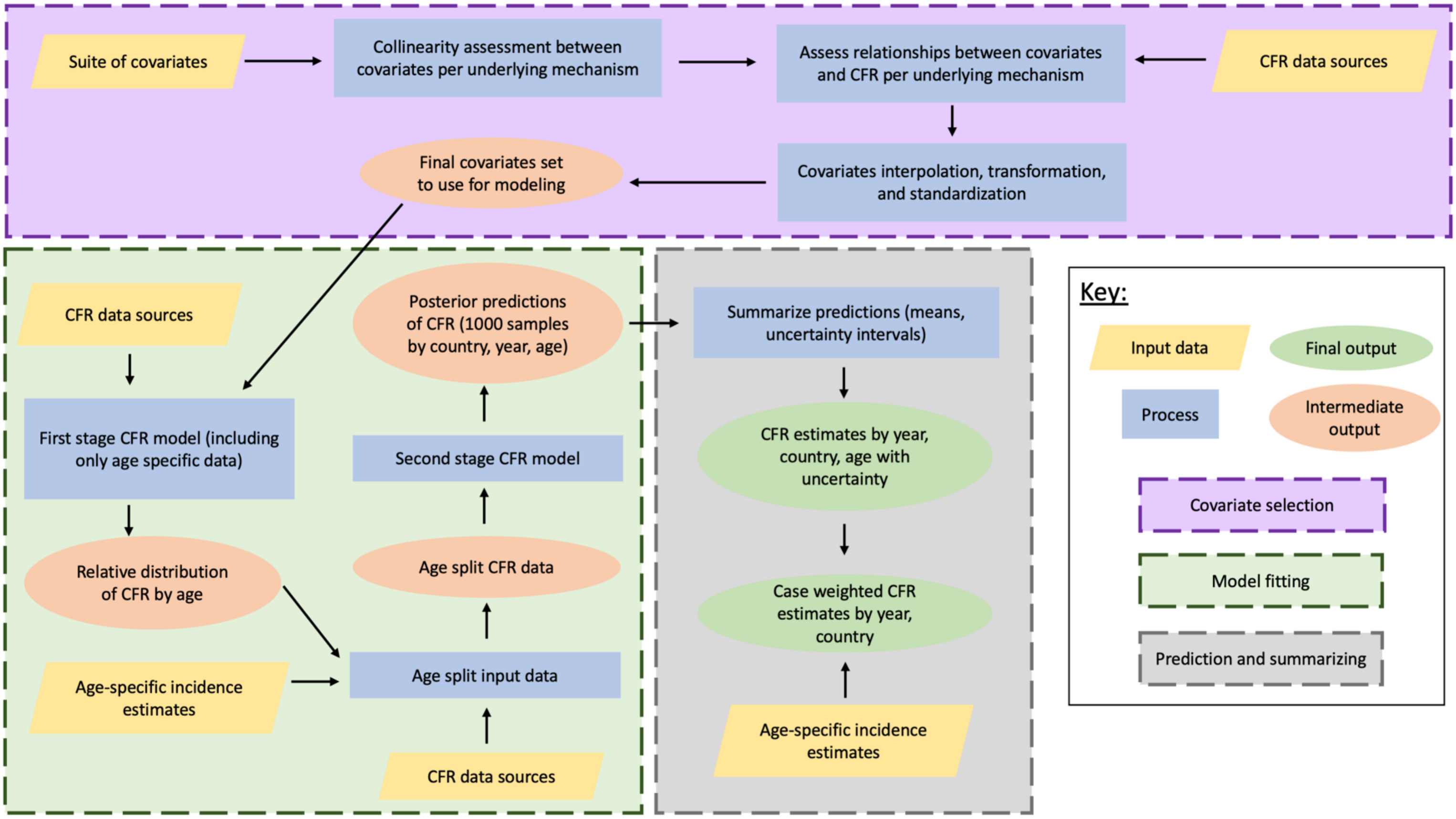

Supplementary Figure 2. Recent data available by country.
For countries with data available, year of most recent data available by country shown in map.

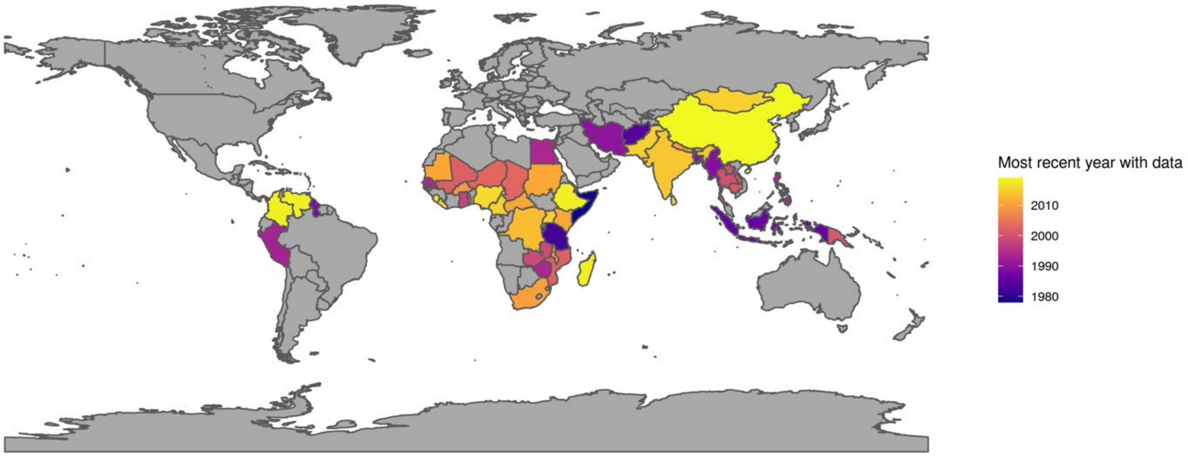

Supplementary Figure 3. Relative age pattern from first-stage model with 4 knots (with 2 internal).

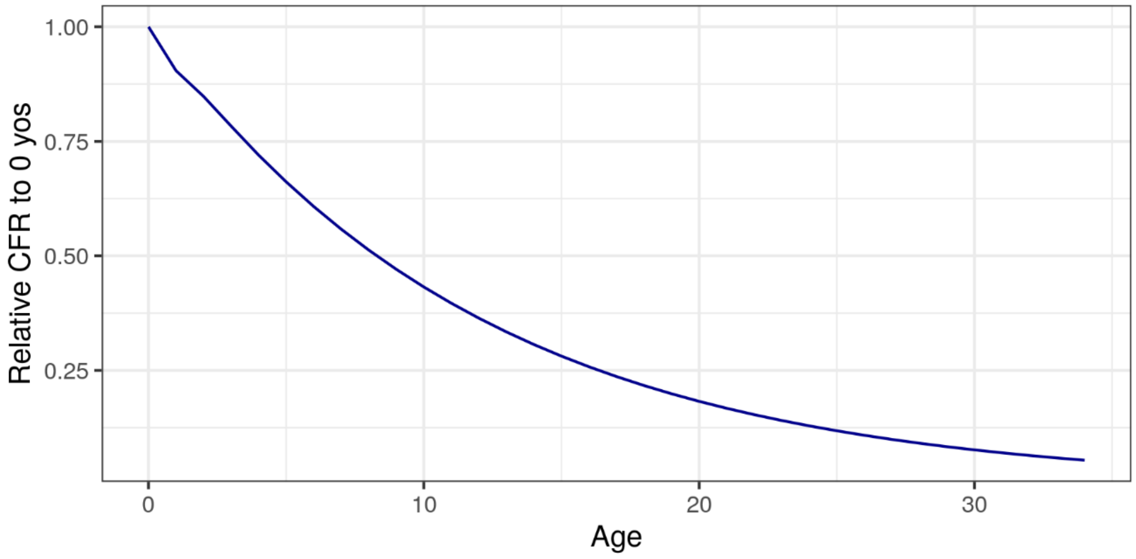

Supplementary Figure 4. Relative age pattern from first-stage model with 5 knots (with 3 internal).

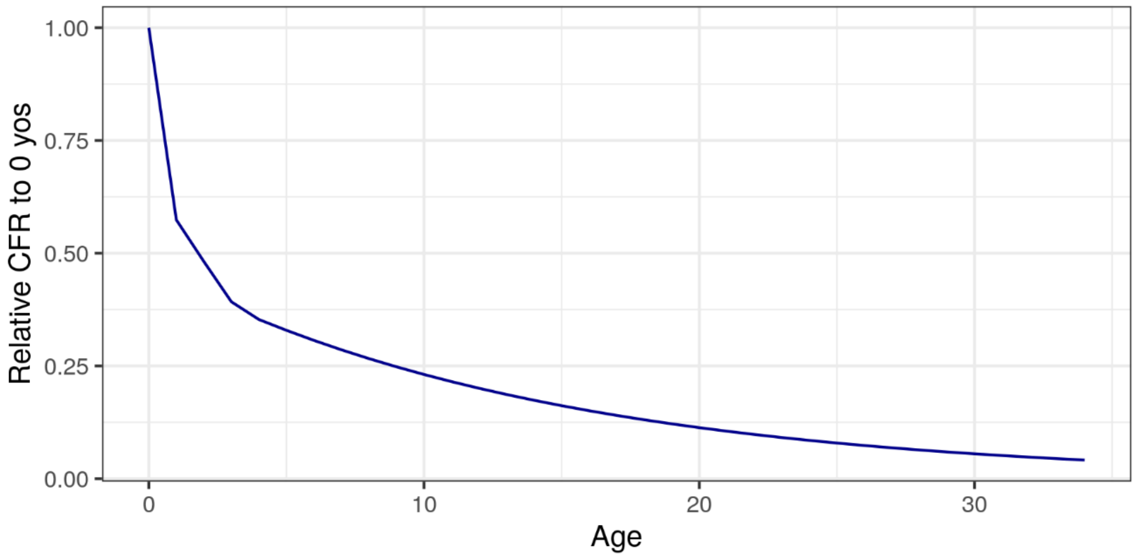

Supplementary Figure 5. Relationship between age of input data and standardized measles incidence from country-year input data was collected.
Grey lines represent a smooth loess curve, and black lines represent a loess curve weighted on standard error of each input data.

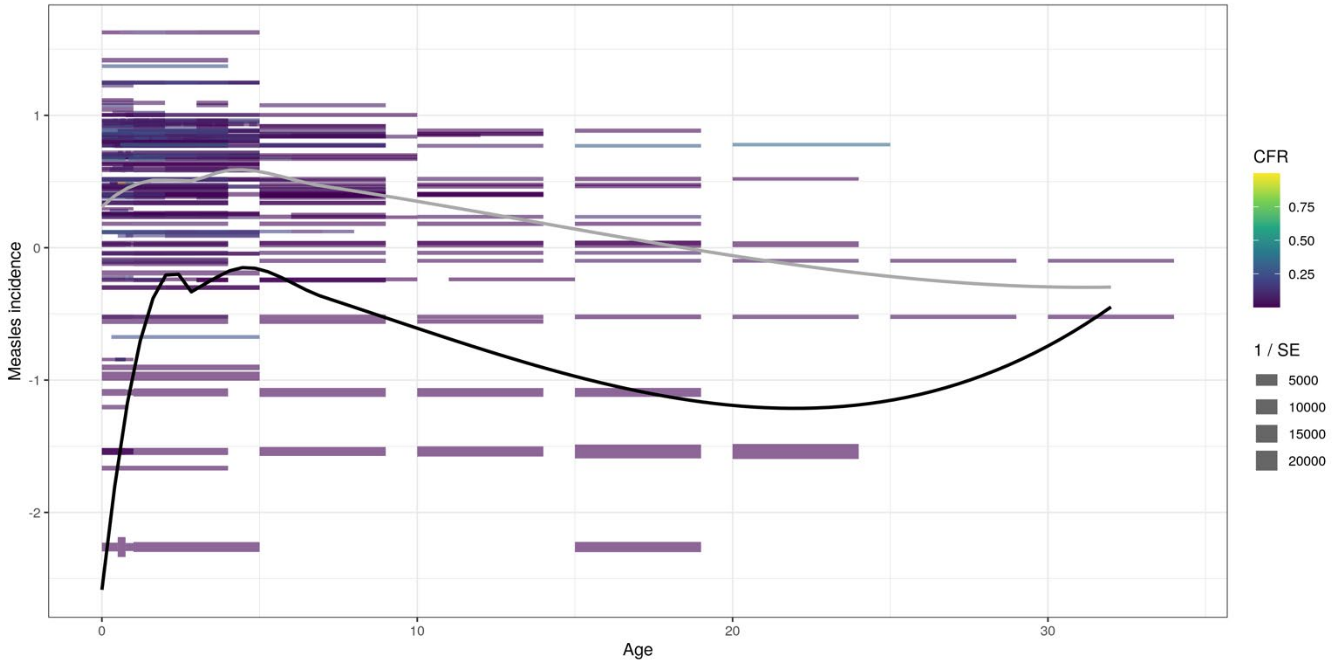

Supplementary Figure 6. Relationship between age of input data and standardized first-dose measles-containing vaccine (MCV1) coverage from country-year input data was collected.
Grey lines represent a smooth loess curve, and black lines represent a loess curve weighted on standard error of each input data.

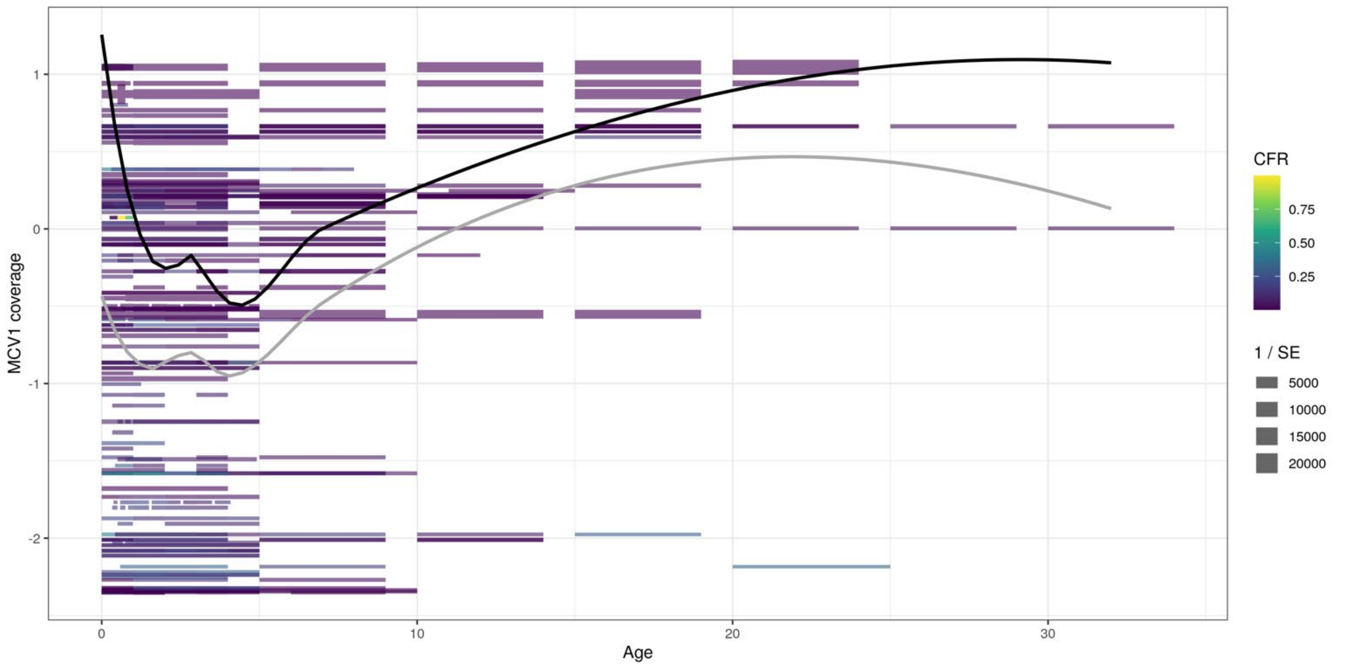

Supplementary Figure 7. Mean age of measles cases by country and year.
Grey lines represent the mean age of cases by year for each country included in analysis, and the red line is a smoothed LOESS curve through individual country lines.

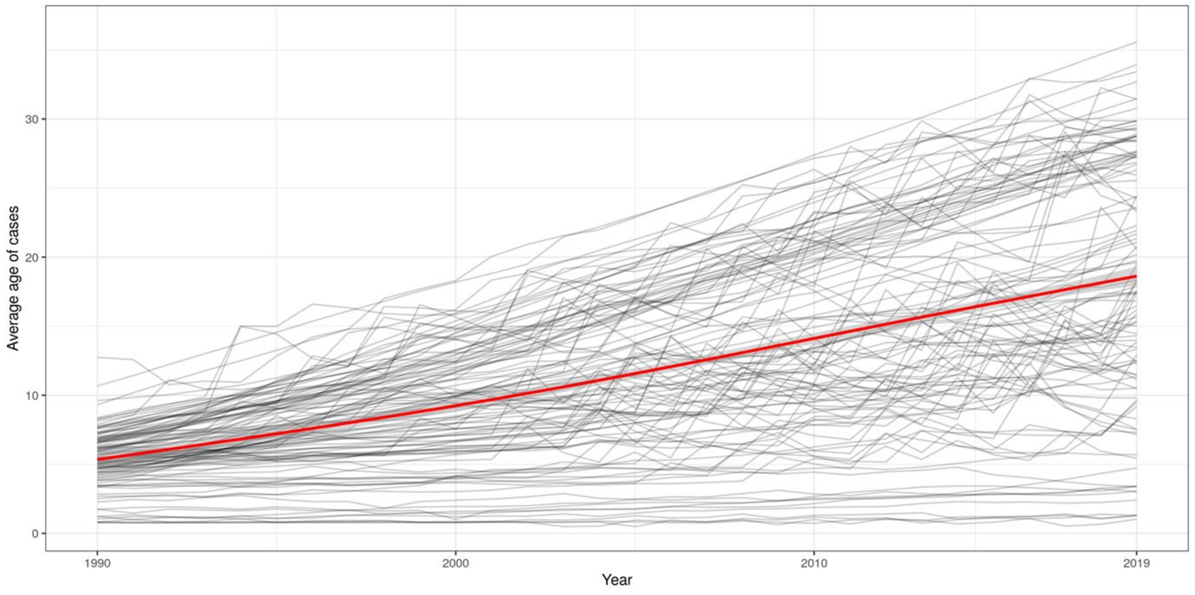

Supplementary Figure 8. Standardized and unstandardized estimates of case-weighted measles CFR across all countries from 1990 to 2019.
Case-weighted mean CFR across LMICs is presented in yellow, by year. Using the UN standard population from 2019, we age-standardized case-weighted mean CFR estimates for LMICs (shown in red). In blue, we additionally age-standardized case-weighted mean CFR estimates using the age distribution across cases in LMICs in 1990 as our "standard population".

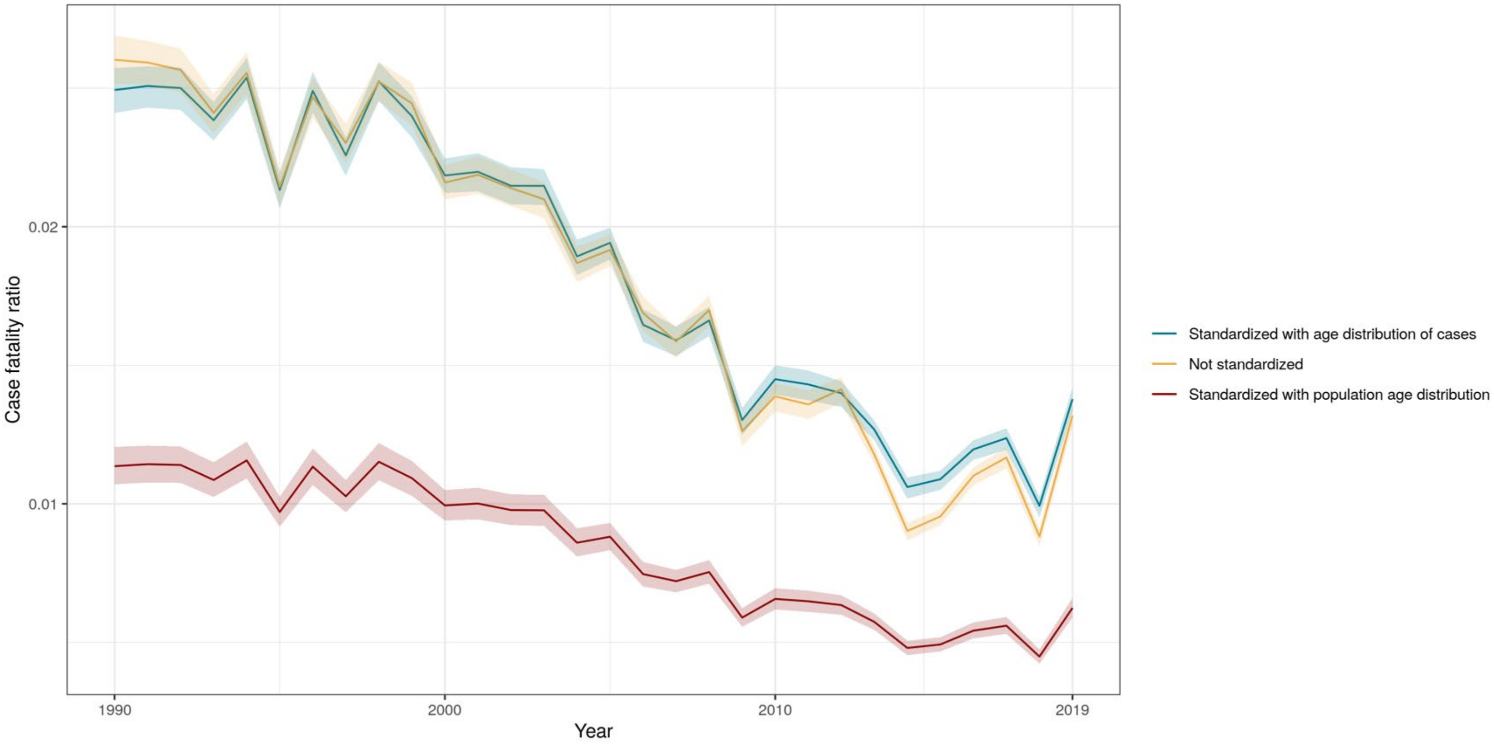

Supplementary Figure 9a-d. Age-specific, community-based, case-weighted case fatality ratio (CFR) estimates among 0–14-year-olds for low- and middle-income countries – for single years 1990, 2000, 2010, and 2019.

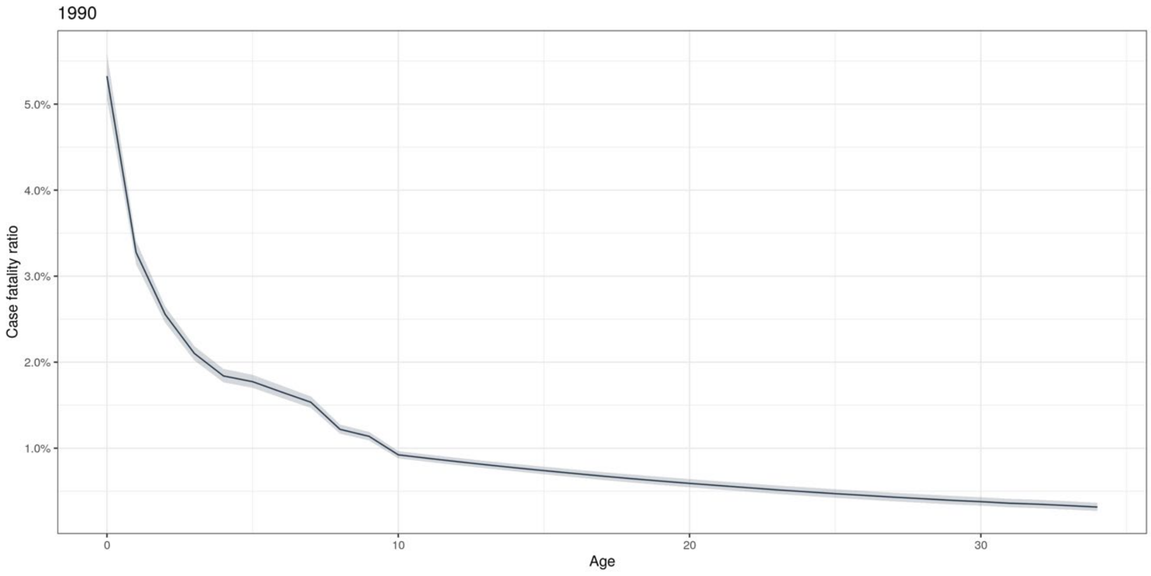

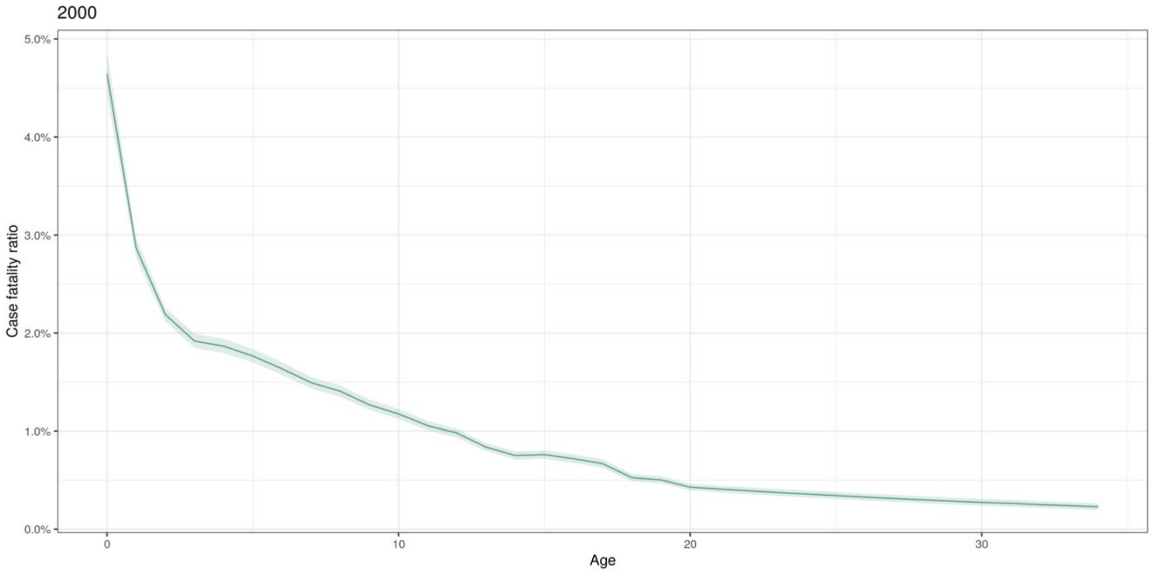

2010

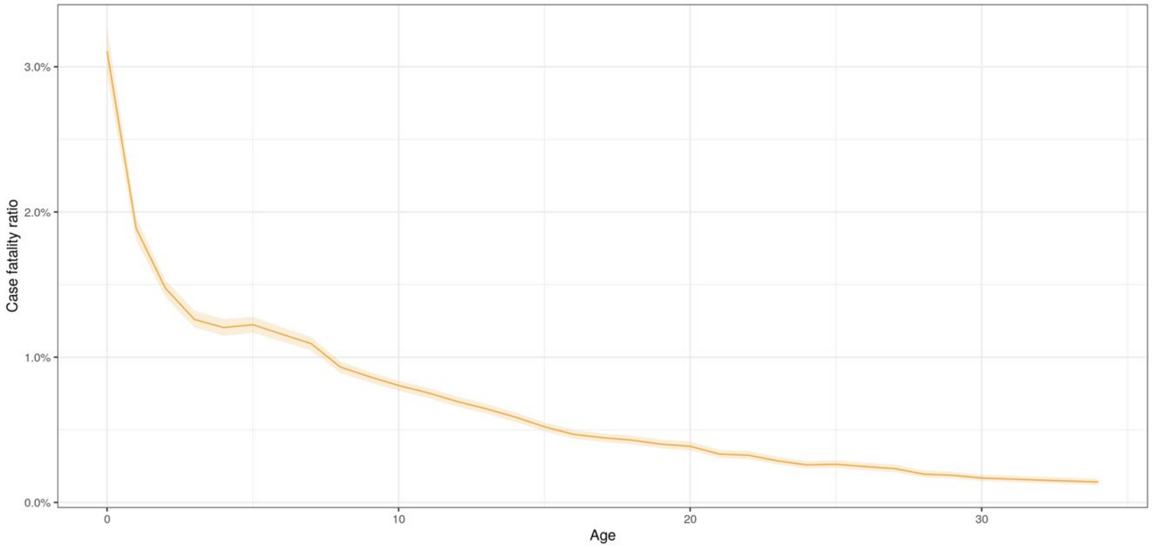

3.0%
2.0%
1.0%
0.0%
Case fatality ratio
0
10
20
30
Age

2019

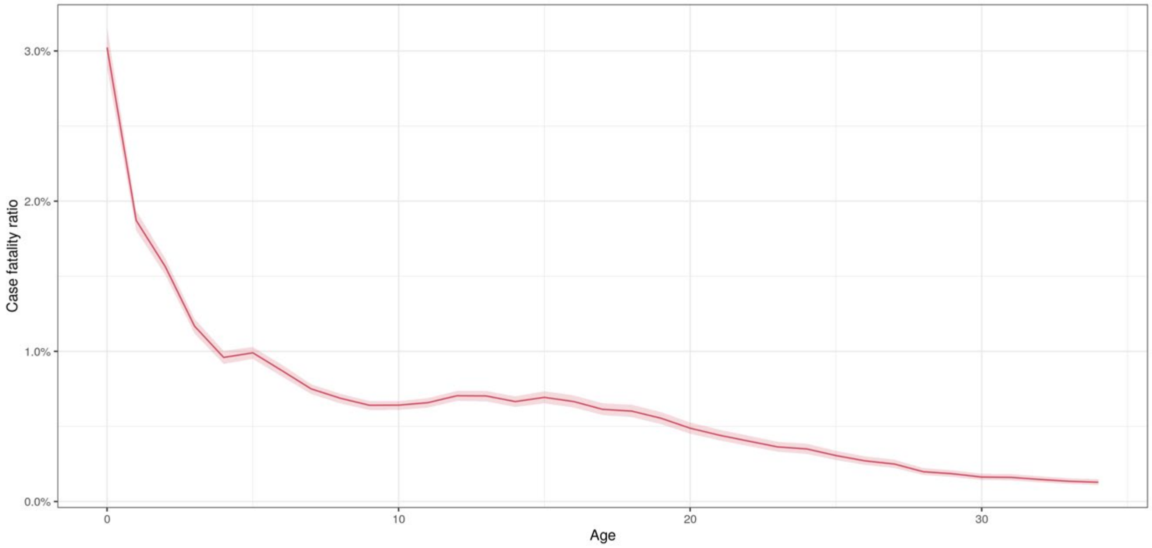

3.0%
2.0%
1.0%
0.0%
Case fatality ratio
0
10
20
30
Age

Supplementary Figure 10a-d. Age-specific, community-based, case-weighted case fatality ratio (CFR) (CFR) estimates among 0–14-year-olds, by region – for single years 1990, 2000, 2010, and 2019.

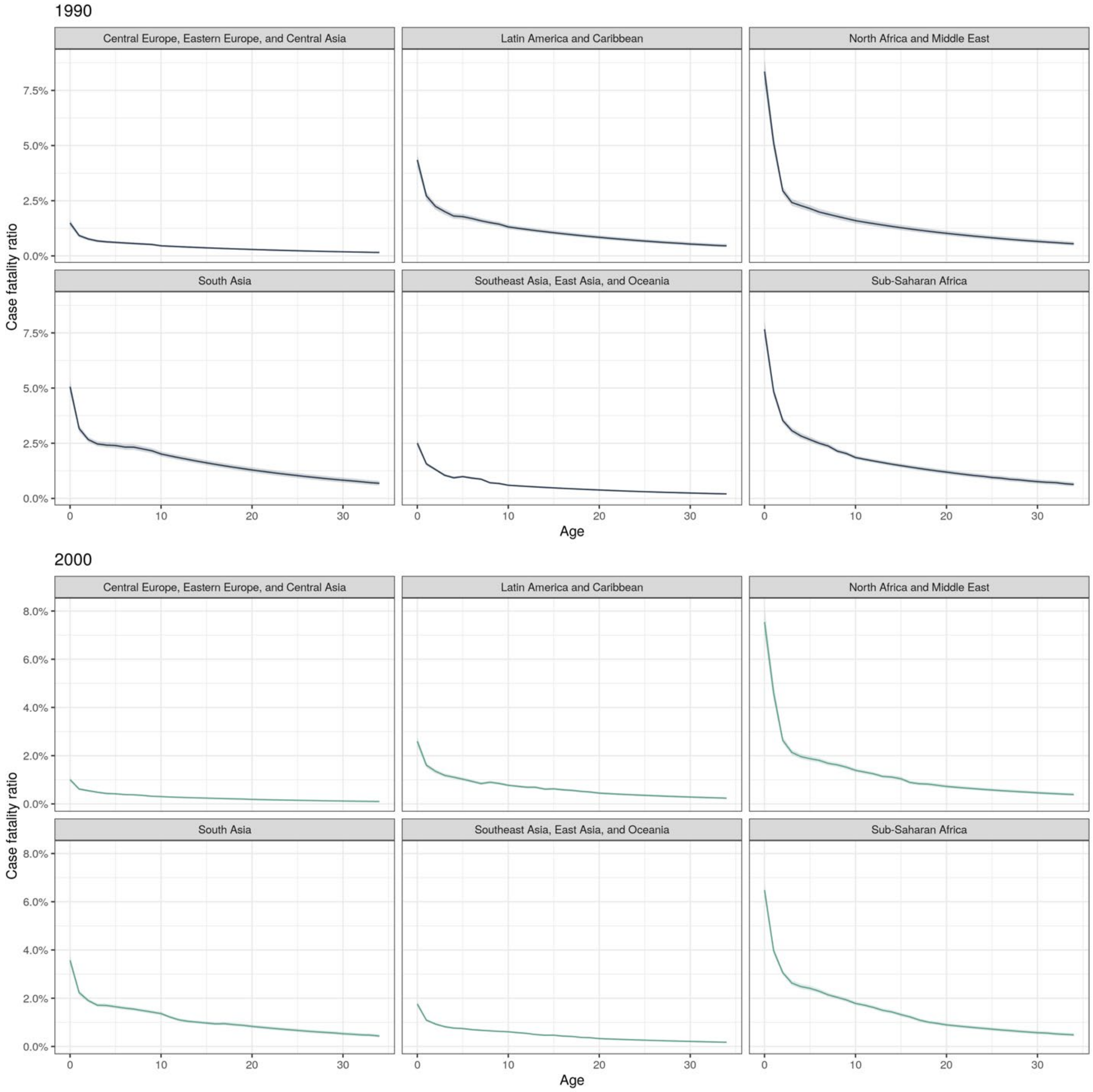

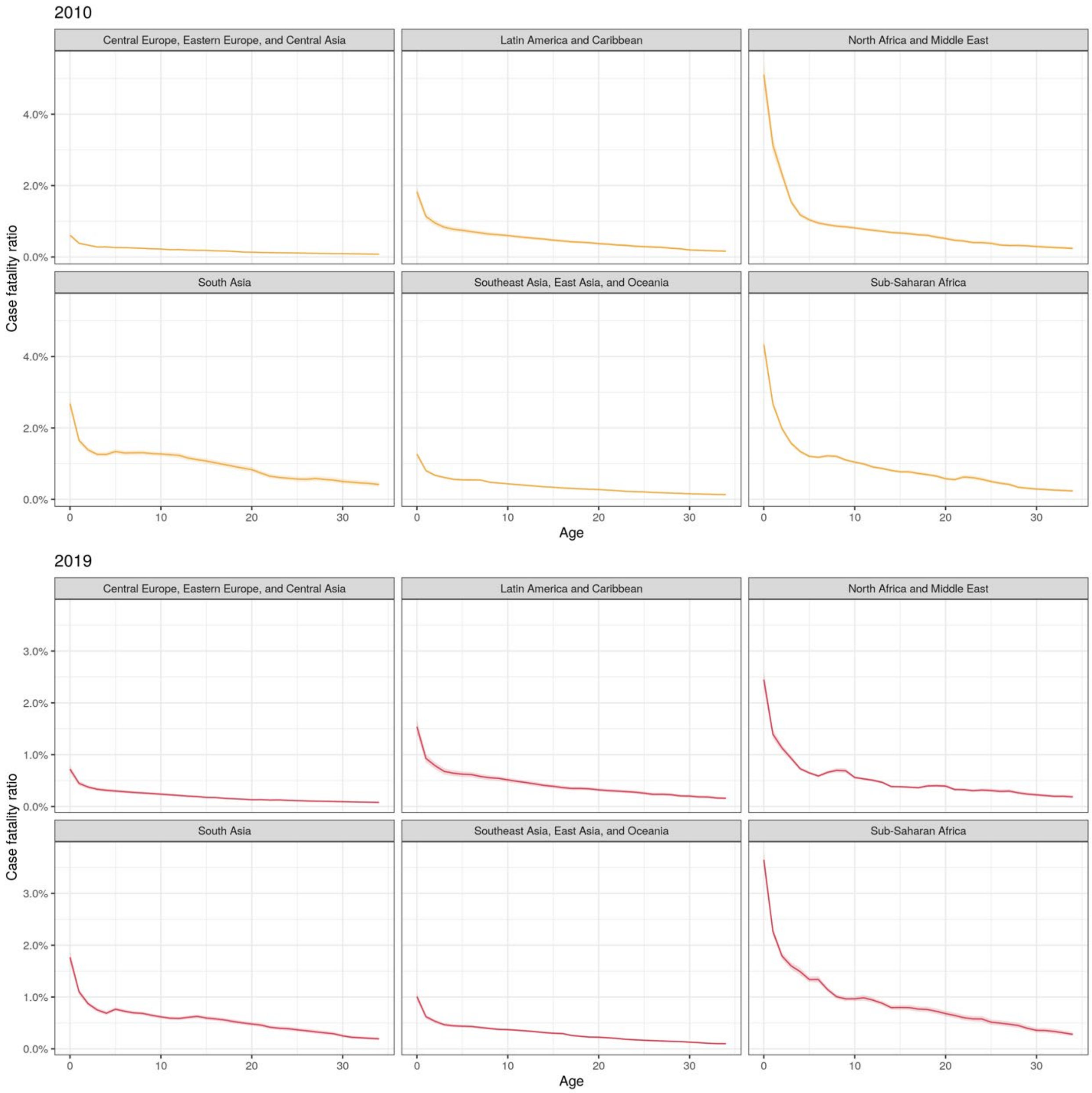

Supplementary Figure 11. Heat maps of age- and year-specific CFR by community- and hospital-based settings by country.

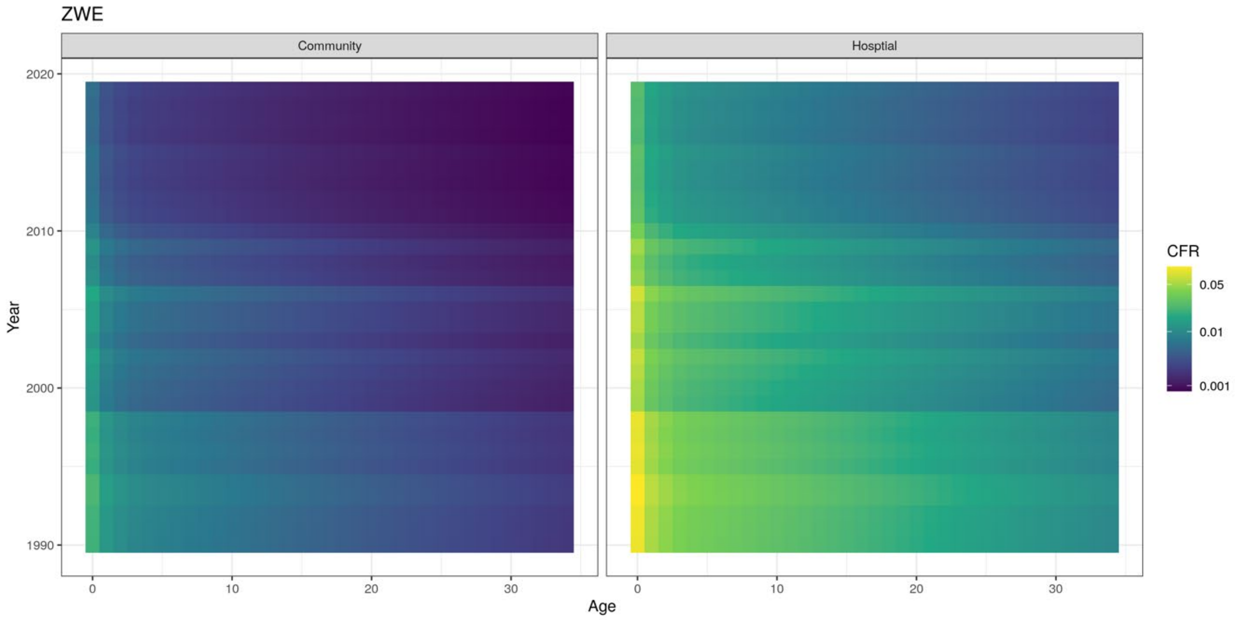
ZWE
Community
Hosptial
Year
2020
2010
2000
1990
Age
0
10
20
30
CFR
0.05
0.01
0.001

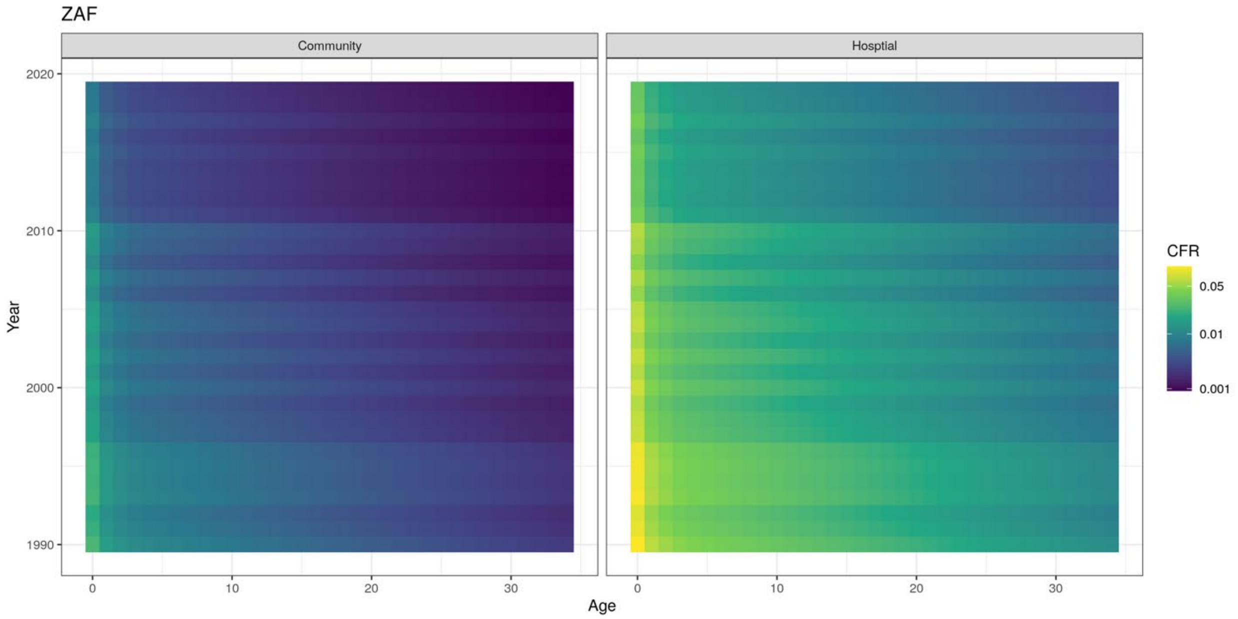
ZAF
Community
Hosptial
Year
2020
2010
2000
1990
Age
0
10
20
30
CFR
0.05
0.01
0.001

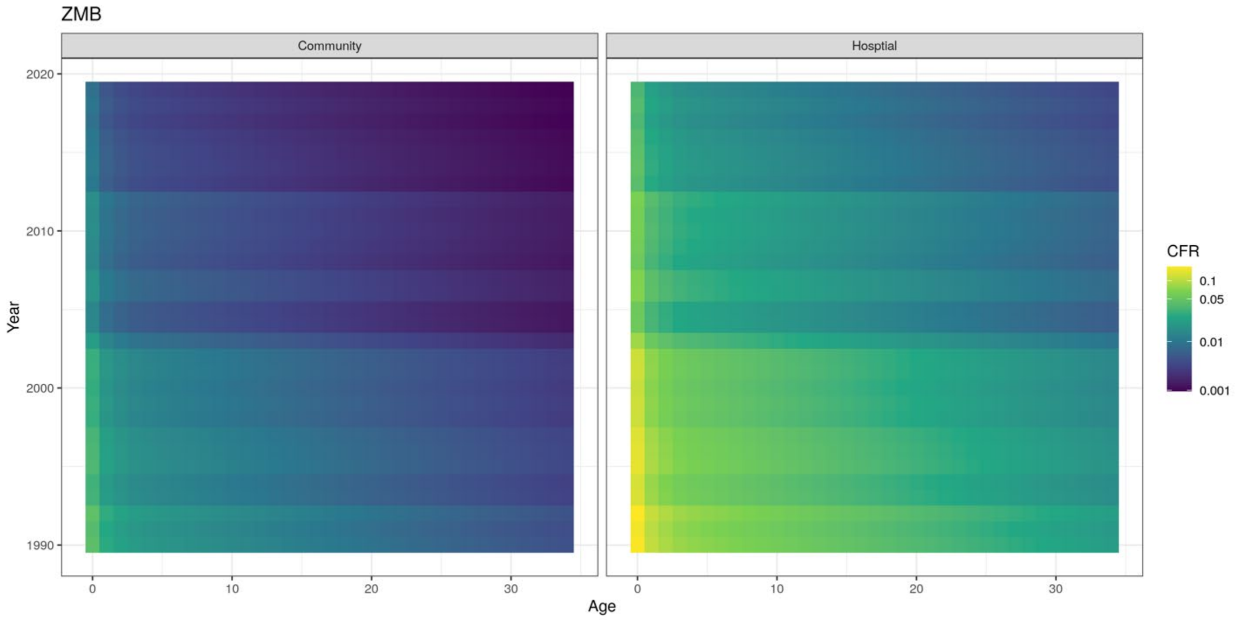
ZMB
Community
Hosptial
Year
Age
CFR
0.1
0.05
0.01
0.001

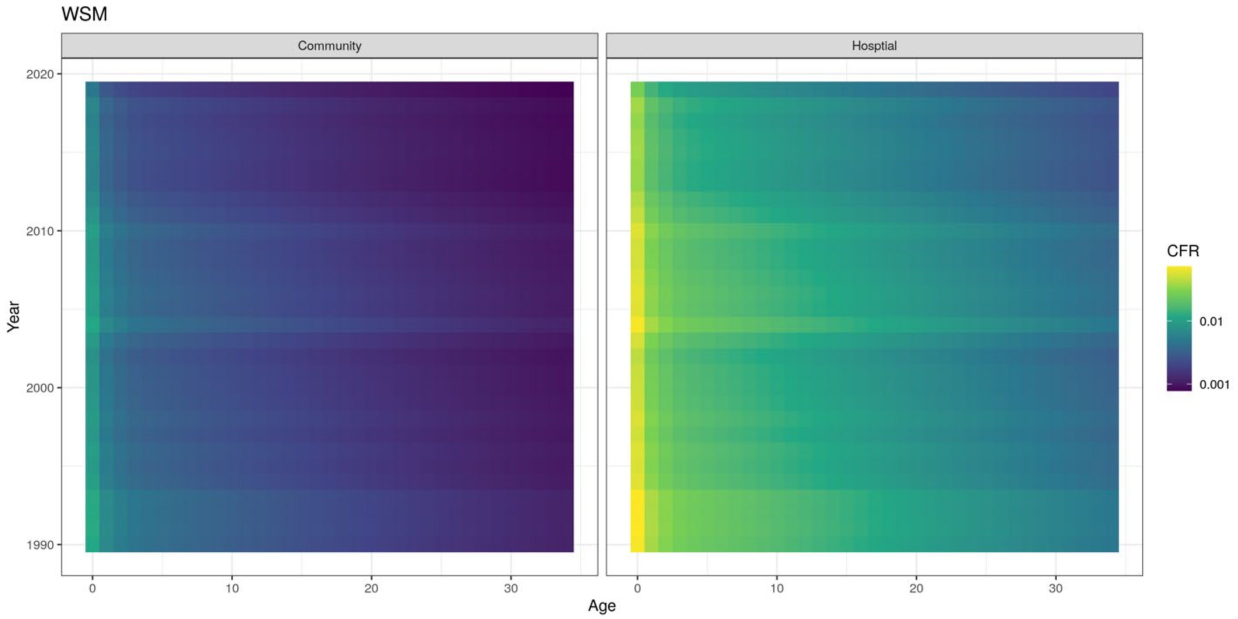
WSM
Community
Hosptial
Year
Age
CFR
0.01
0.001

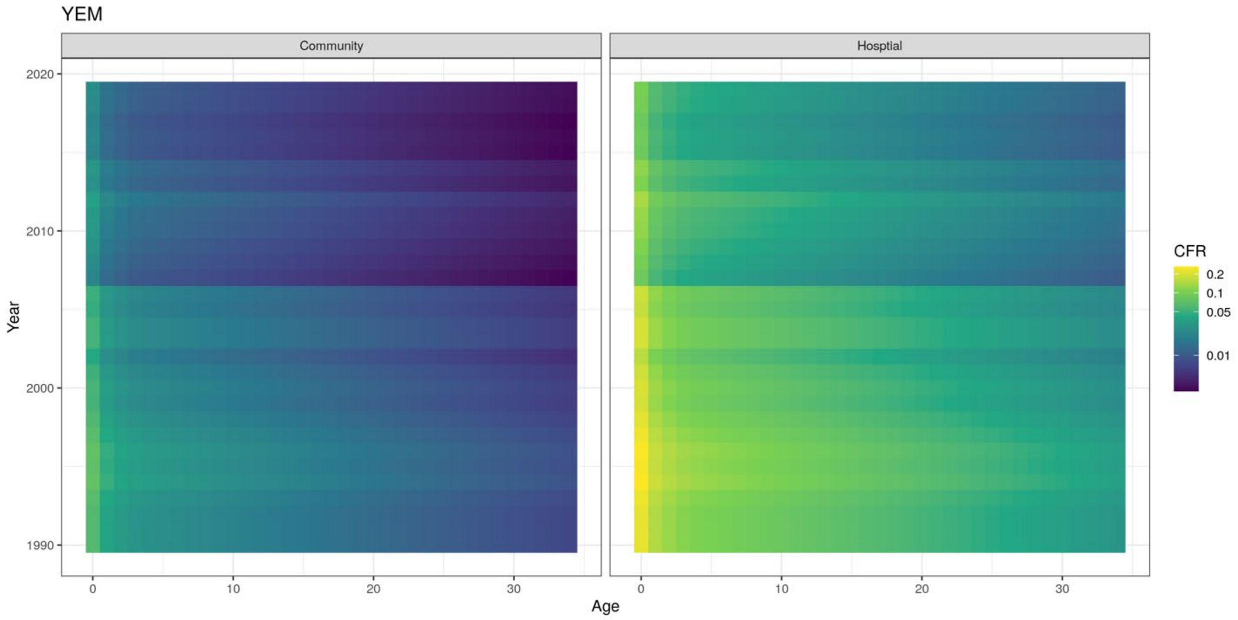
YEM
Community
Hosptial
Year
Age
CFR
0.2
0.1
0.05
0.01
2020
2010
2000
1990
0
10
20
30

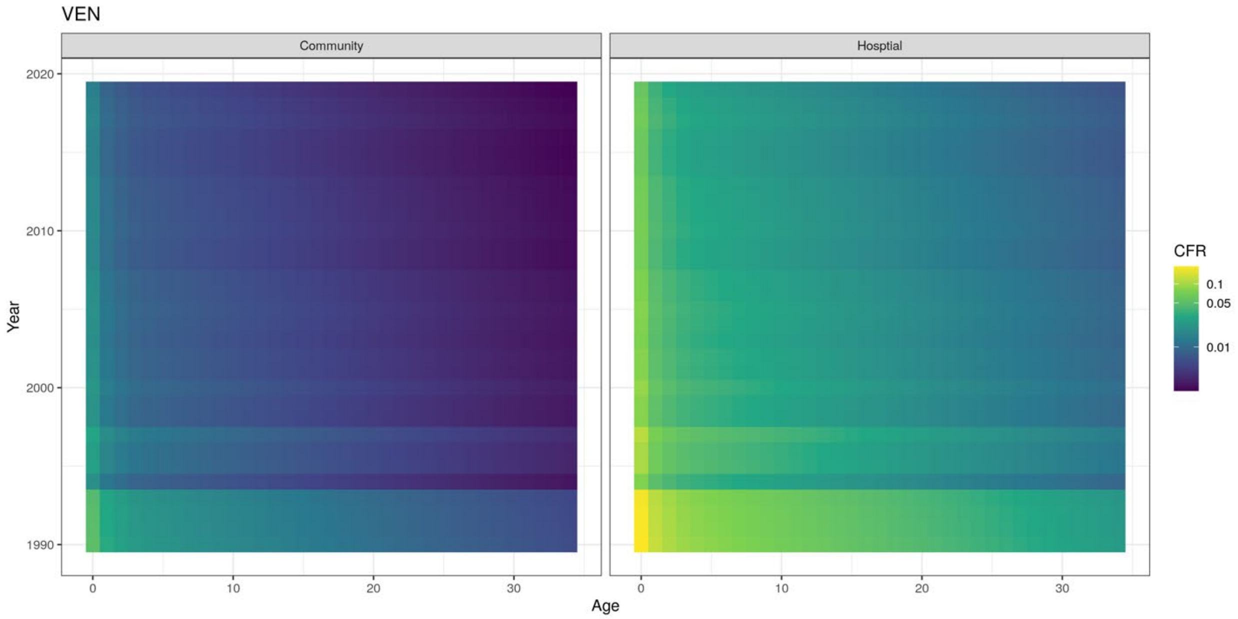
VEN
Community
Hosptial
Year
Age
CFR
0.1
0.05
0.01
2020
2010
2000
1990
0
10
20
30

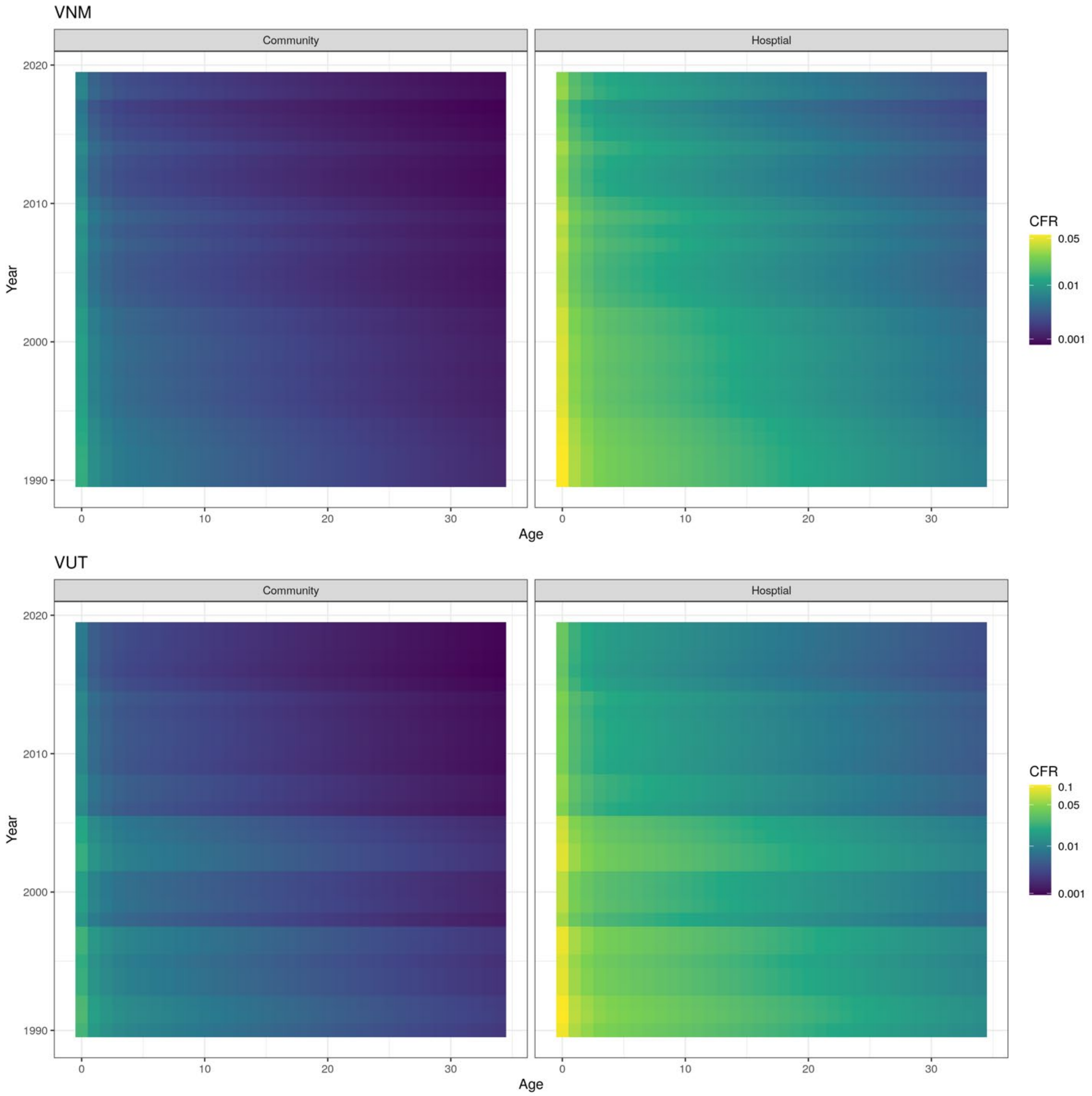
VNM
Community
Hosptial
2020
2010
2000
1990
Year
0
10
20
30
Age
CFR
0.05
0.01
0.001
VUT
Community
Hosptial
CFR
0.1
0.05
0.01
0.001

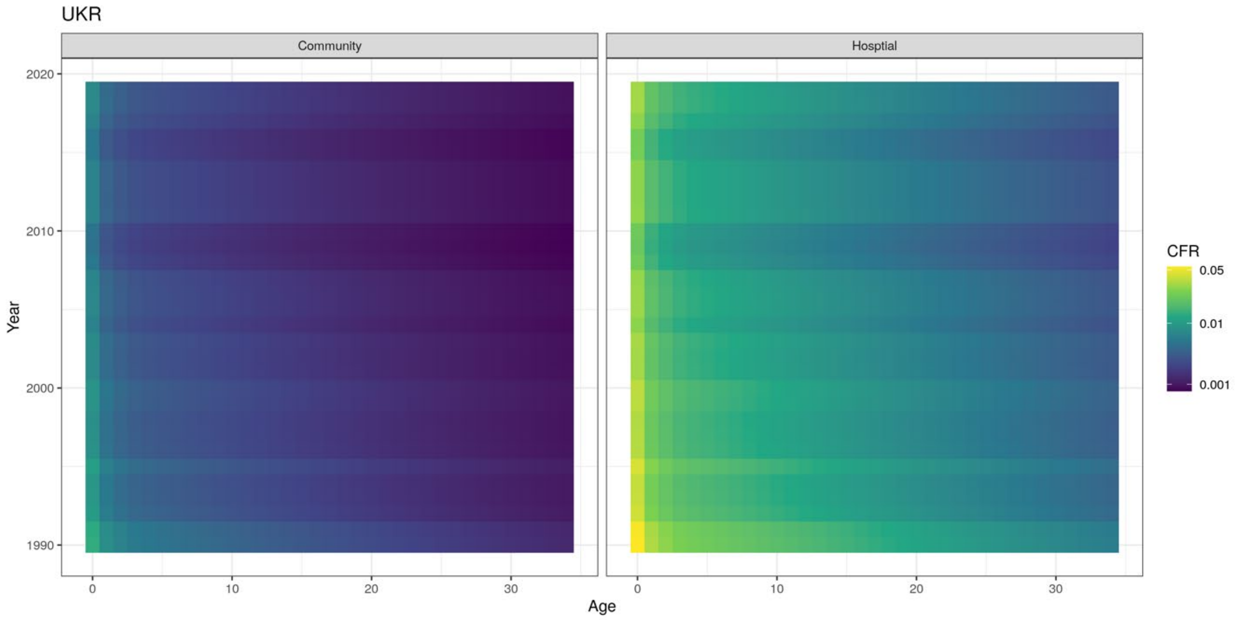
UKR
Community
Hosptial
Year
Age
CFR
0.05
0.01
0.001
2020
2010
2000
1990
0
10
20
30

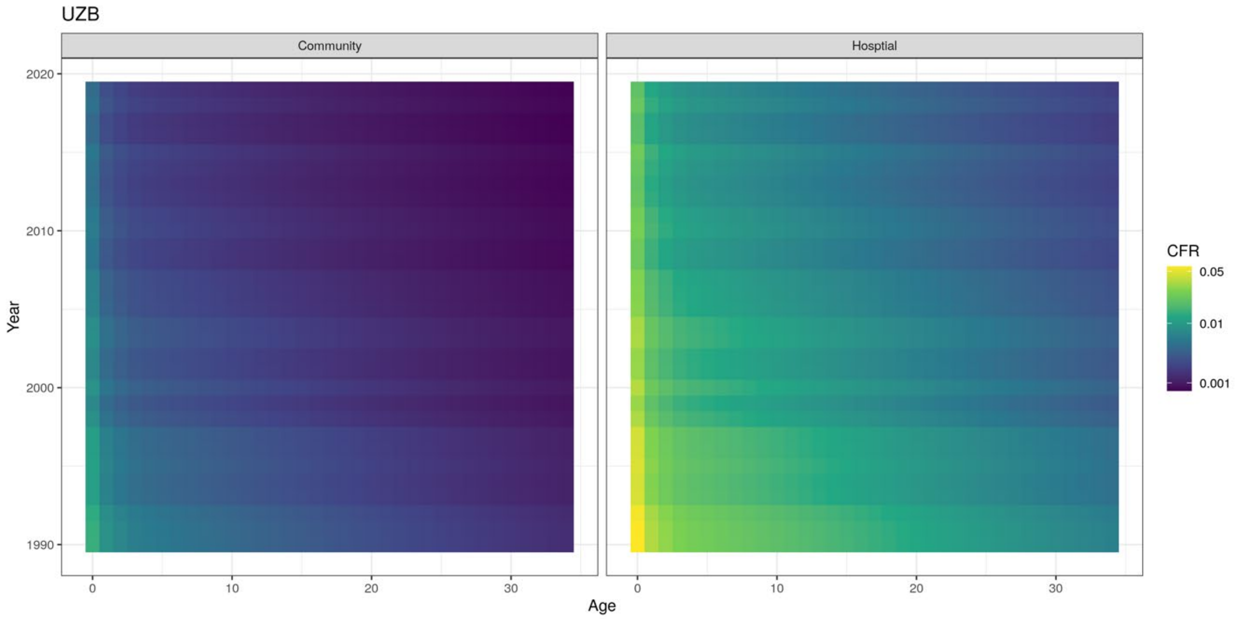
UZB
Community
Hosptial
Year
Age
CFR
0.05
0.01
0.001
2020
2010
2000
1990
0
10
20
30

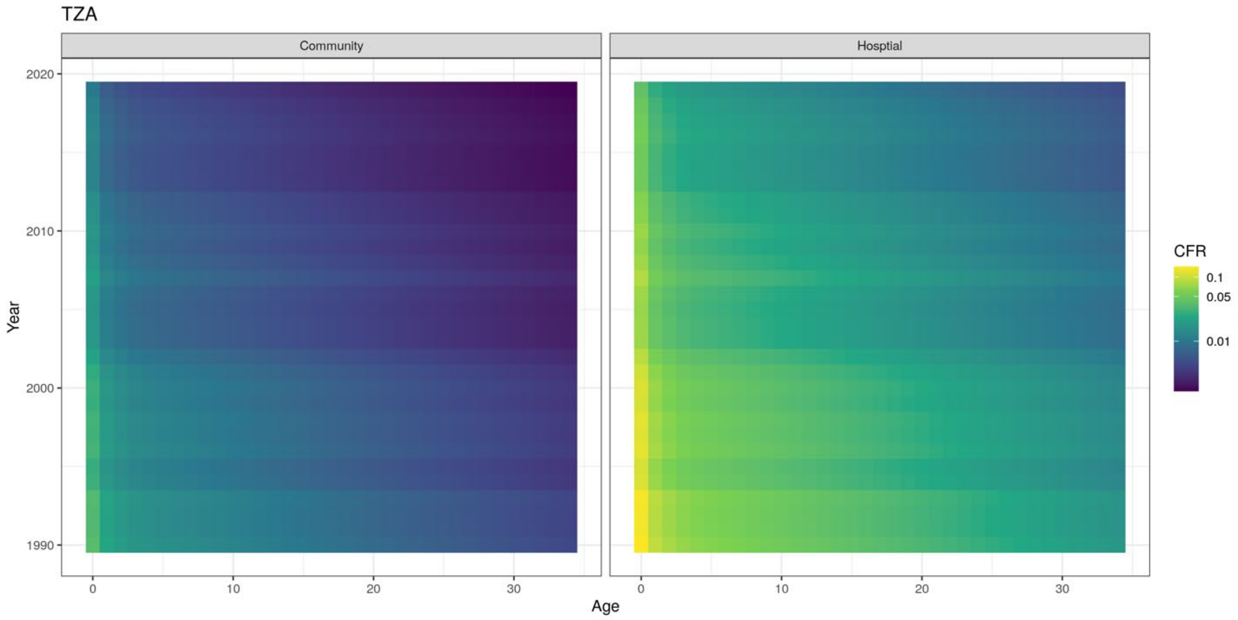
TZA
Community
Hosptial
Year
Age
CFR
0.1
0.05
0.01
2020
2010
2000
1990
0
10
20
30

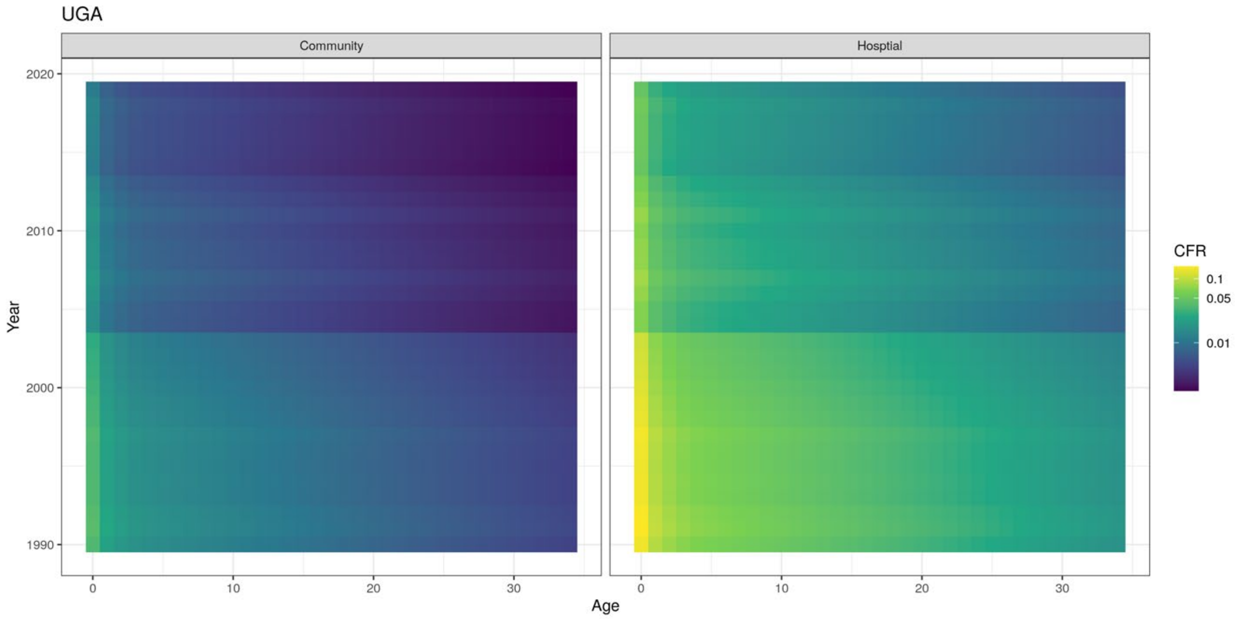
UGA
Community
Hosptial
Year
Age
CFR
0.1
0.05
0.01
2020
2010
2000
1990
0
10
20
30

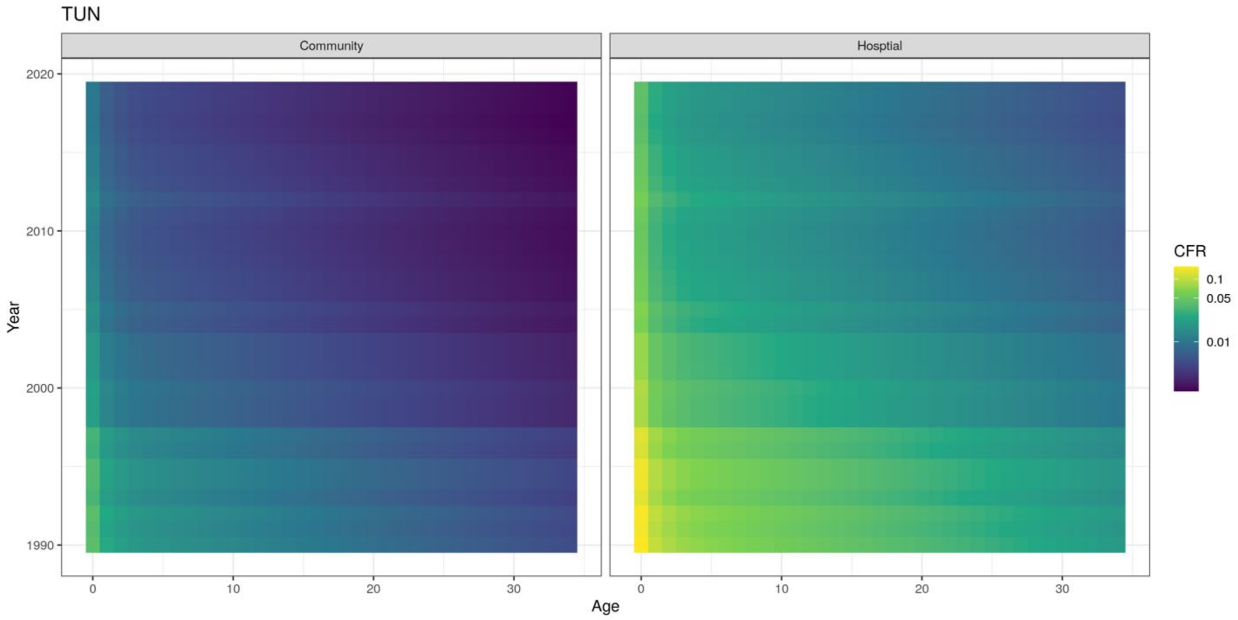
TUN
Community
Hosptial
Year
Age
CFR
0.1
0.05
0.01

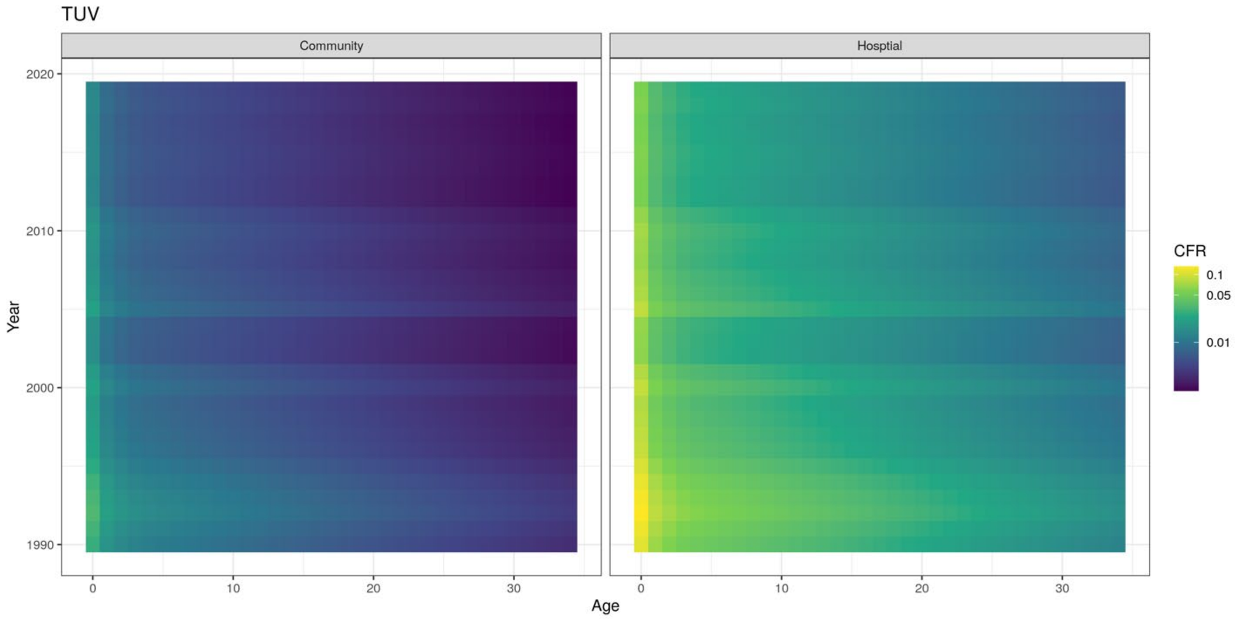
TUV
Community
Hosptial
Year
Age
CFR
0.1
0.05
0.01

TLS

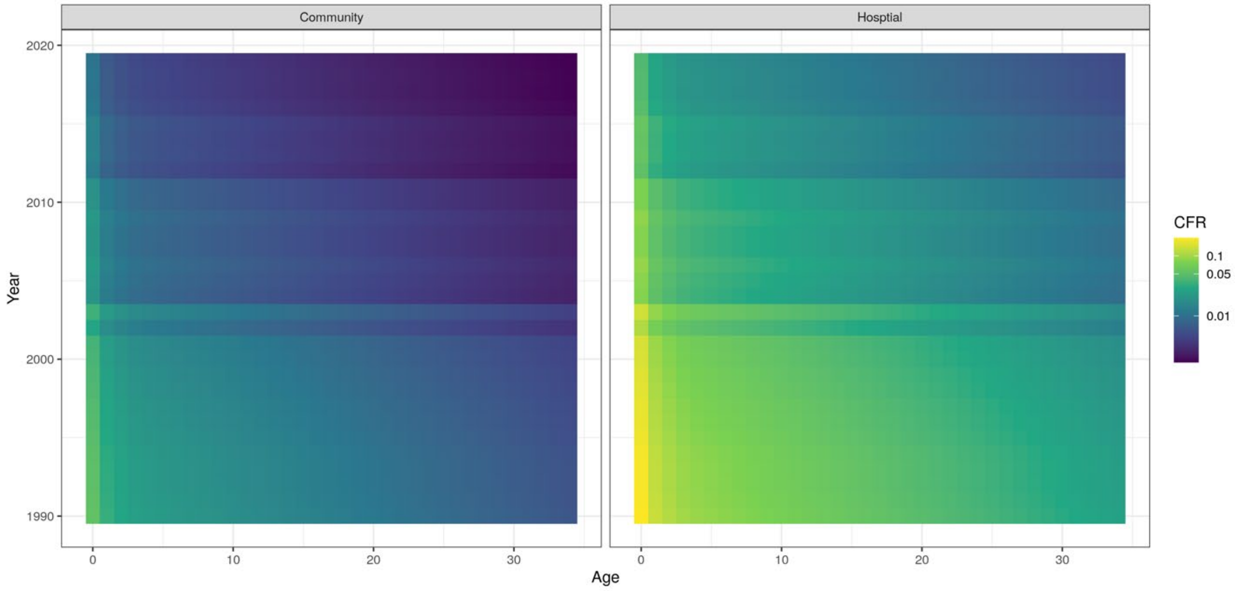
Community
Hosptial
2020
2010
2000
1990
Year
0
10
20
30
Age
CFR
0.1
0.05
0.01

TON

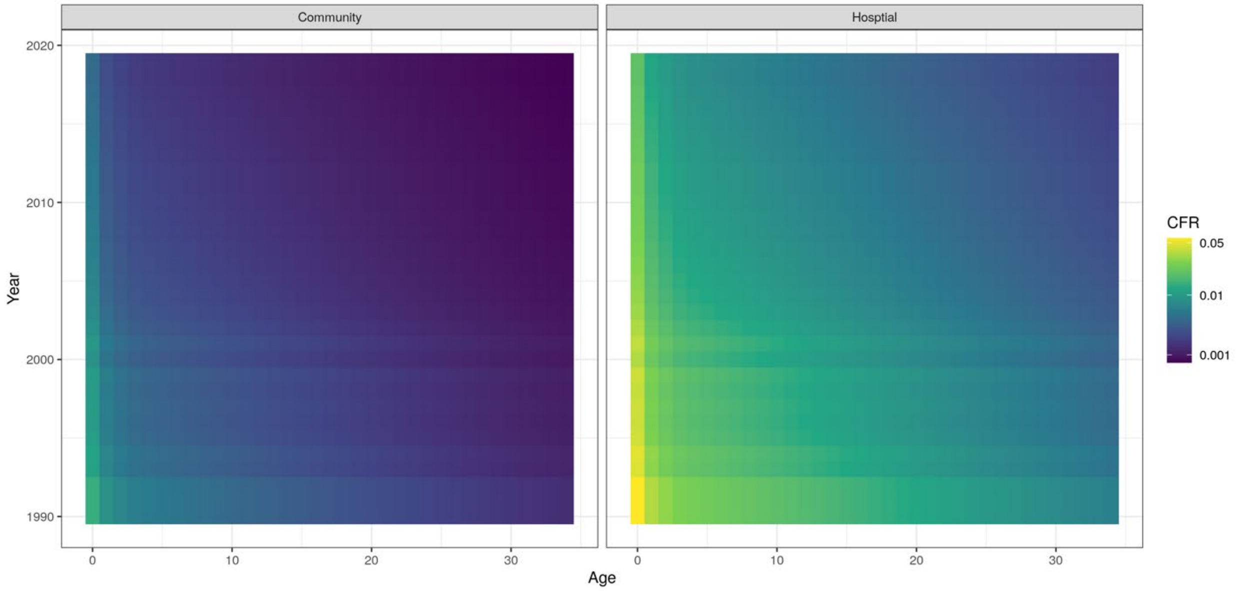
Community
Hosptial
2020
2010
2000
1990
Year
0
10
20
30
Age
CFR
0.05
0.01
0.001

THA

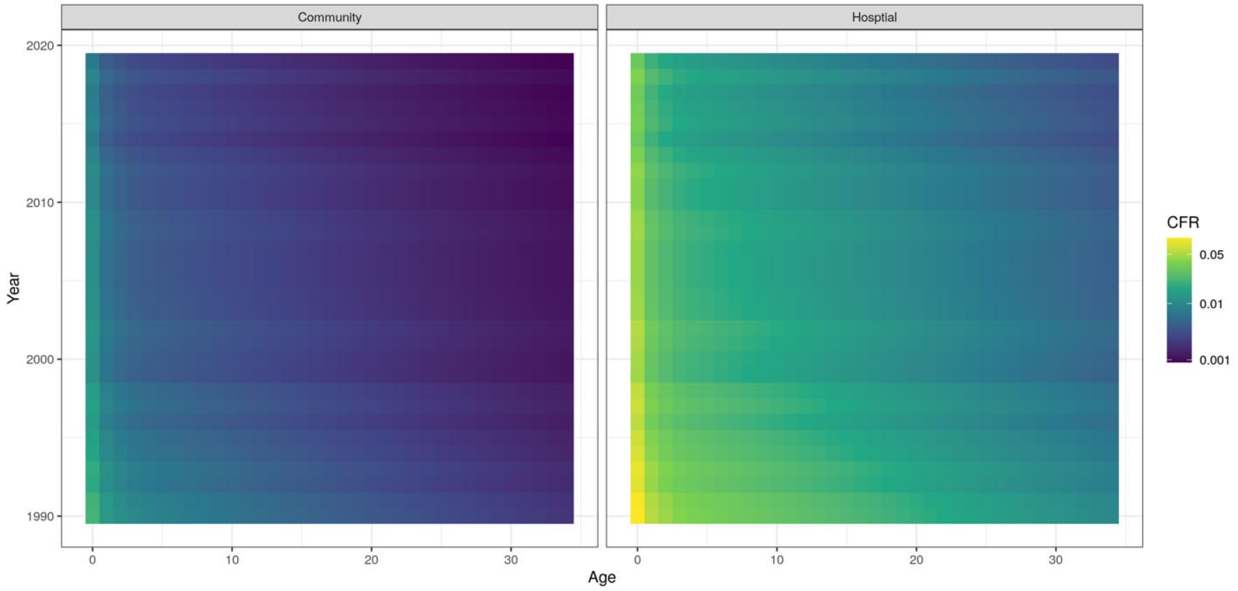
Community
Hosptial
Year
2020
2010
2000
1990
Age
0
10
20
30
CFR
0.05
0.01
0.001

TJK

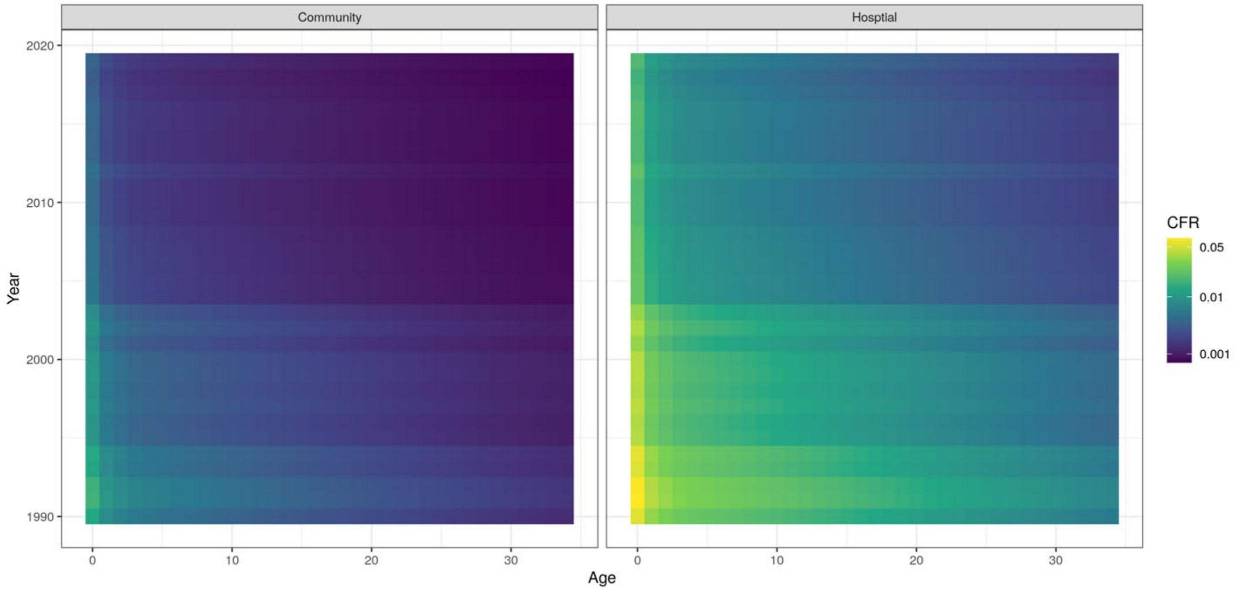
Community
Hosptial
Year
2020
2010
2000
1990
Age
0
10
20
30
CFR
0.05
0.01
0.001

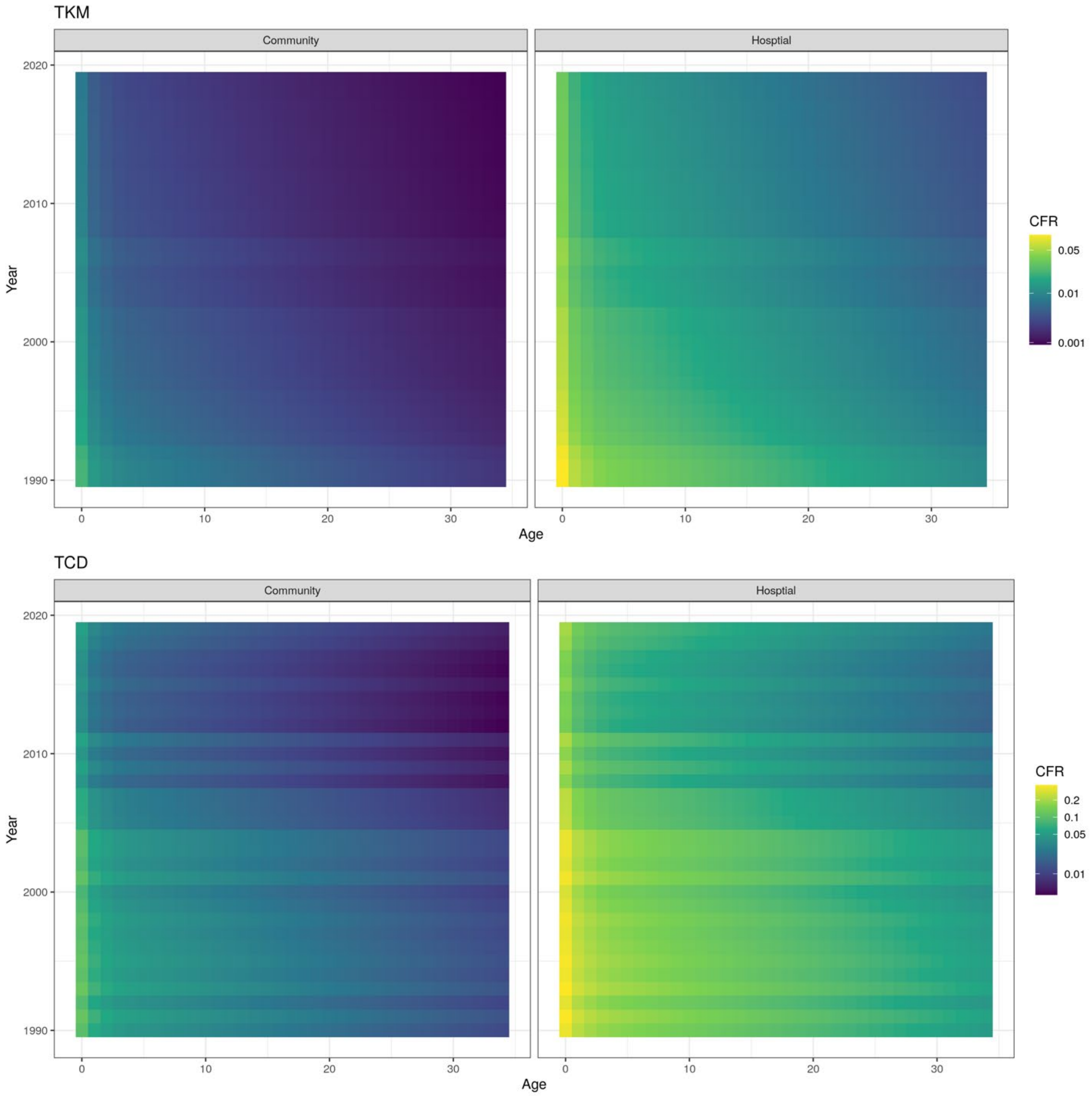

TKM
Community
Hosptial
2020
2010
2000
1990
Year
0
10
20
30
Age
CFR
0.05
0.01
0.001
TCD
Community
Hosptial
2020
2010
2000
1990
Year
0
10
20
30
Age
CFR
0.2
0.1
0.05
0.01

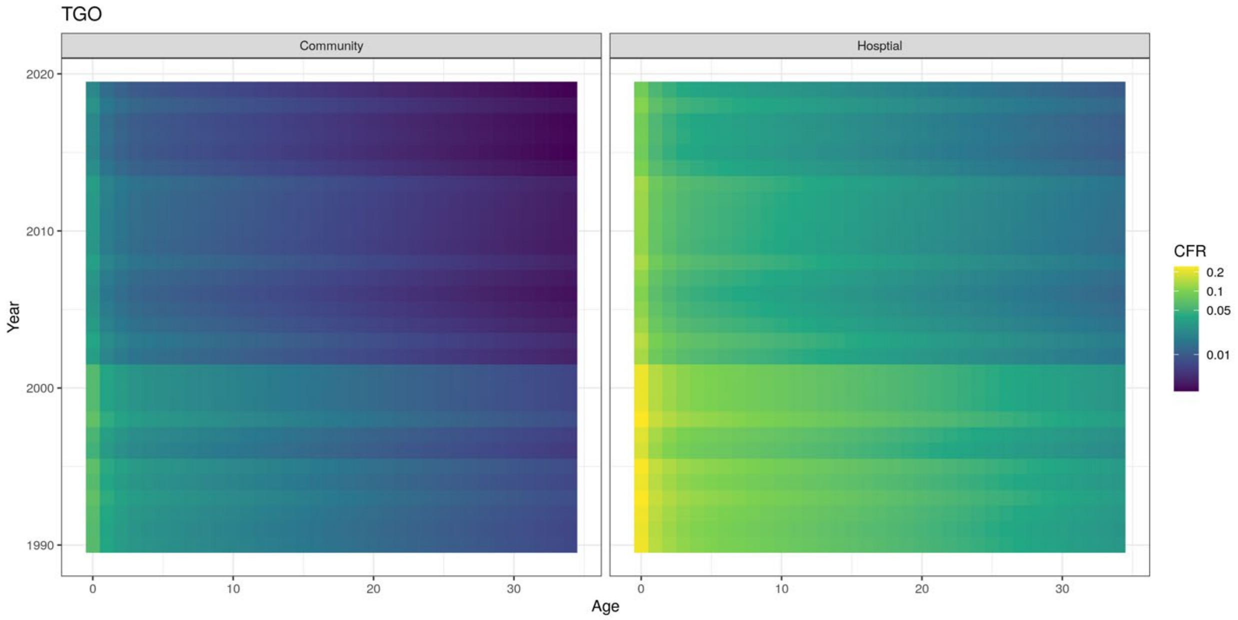
TGO
Community
Hosptial
Year
2020
2010
2000
1990
0
10
20
30
Age
CFR
0.2
0.1
0.05
0.01

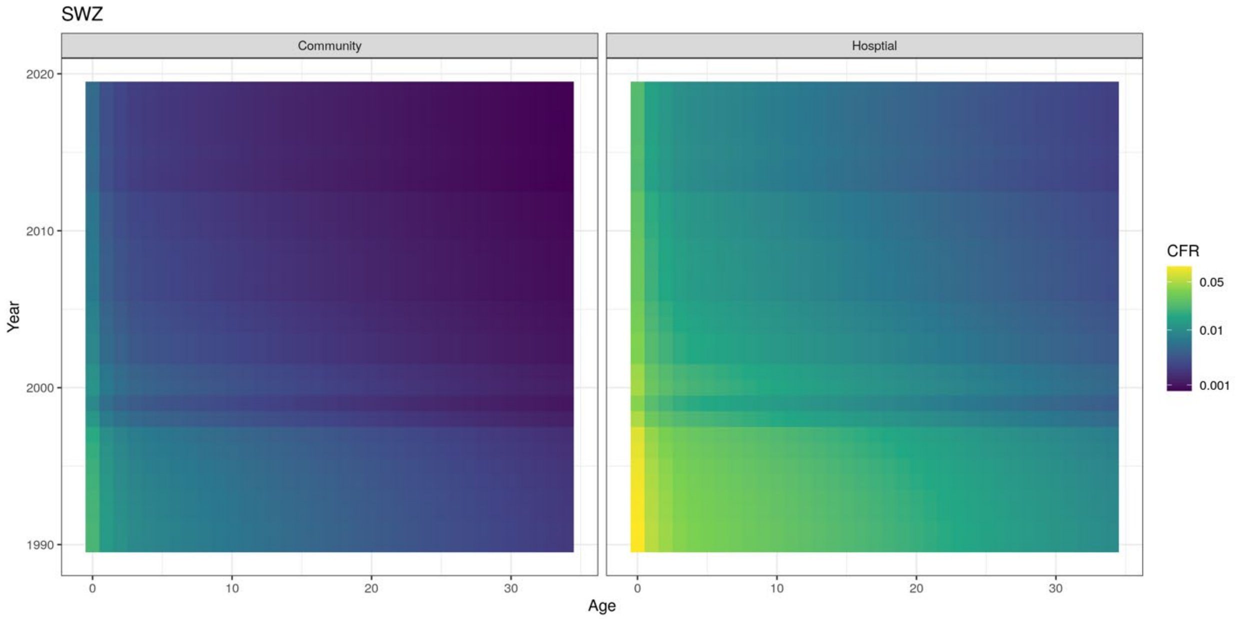
SWZ
Community
Hosptial
Year
2020
2010
2000
1990
0
10
20
30
Age
CFR
0.05
0.01
0.001

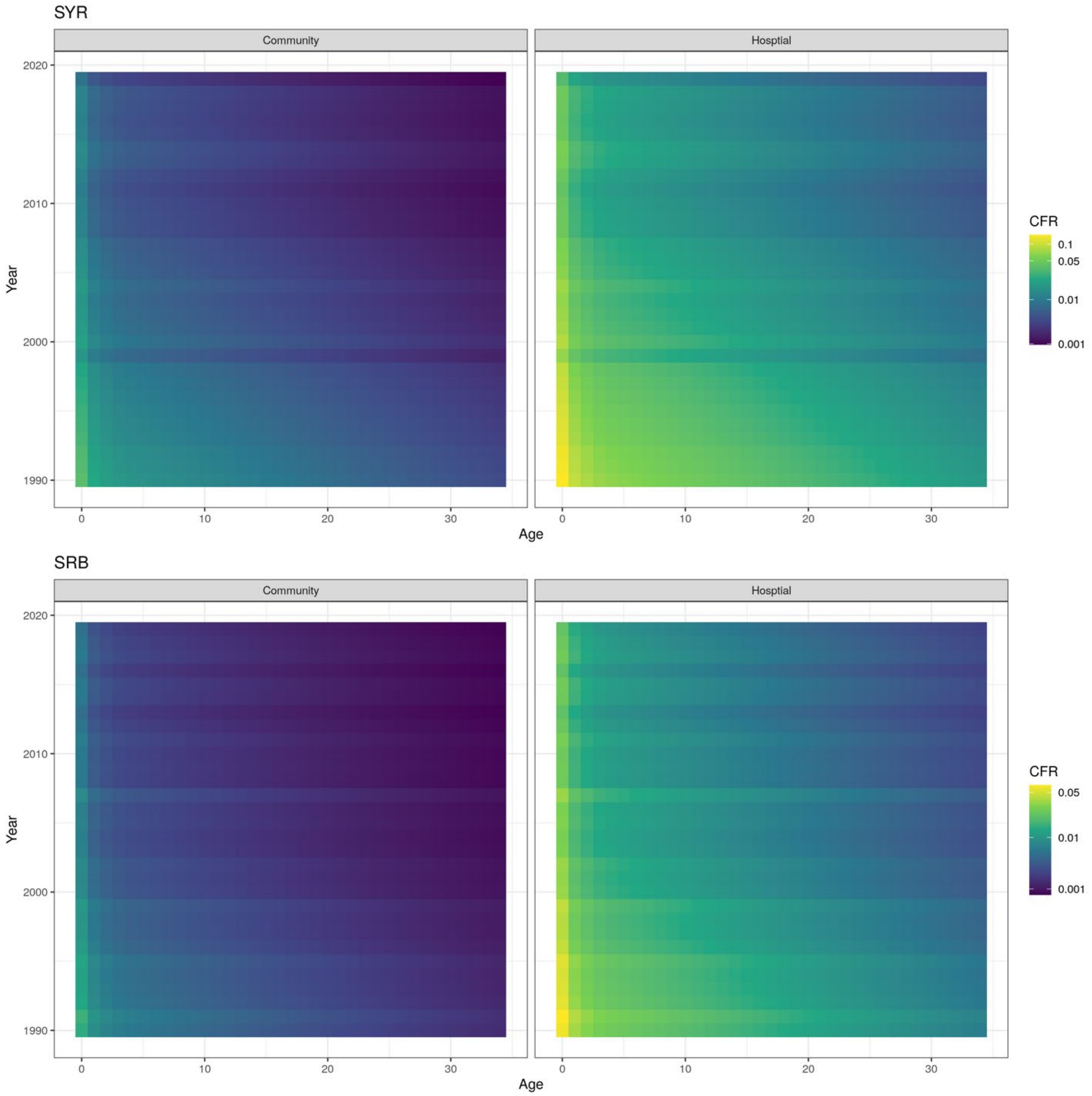

SYR
Community
Hosptial
2020
2010
2000
1990
Year
0
10
20
30
Age
CFR
0.1
0.05
0.01
0.001
SRB
Community
Hosptial
2020
2010
2000
1990
Year
0
10
20
30
Age
CFR
0.05
0.01
0.001

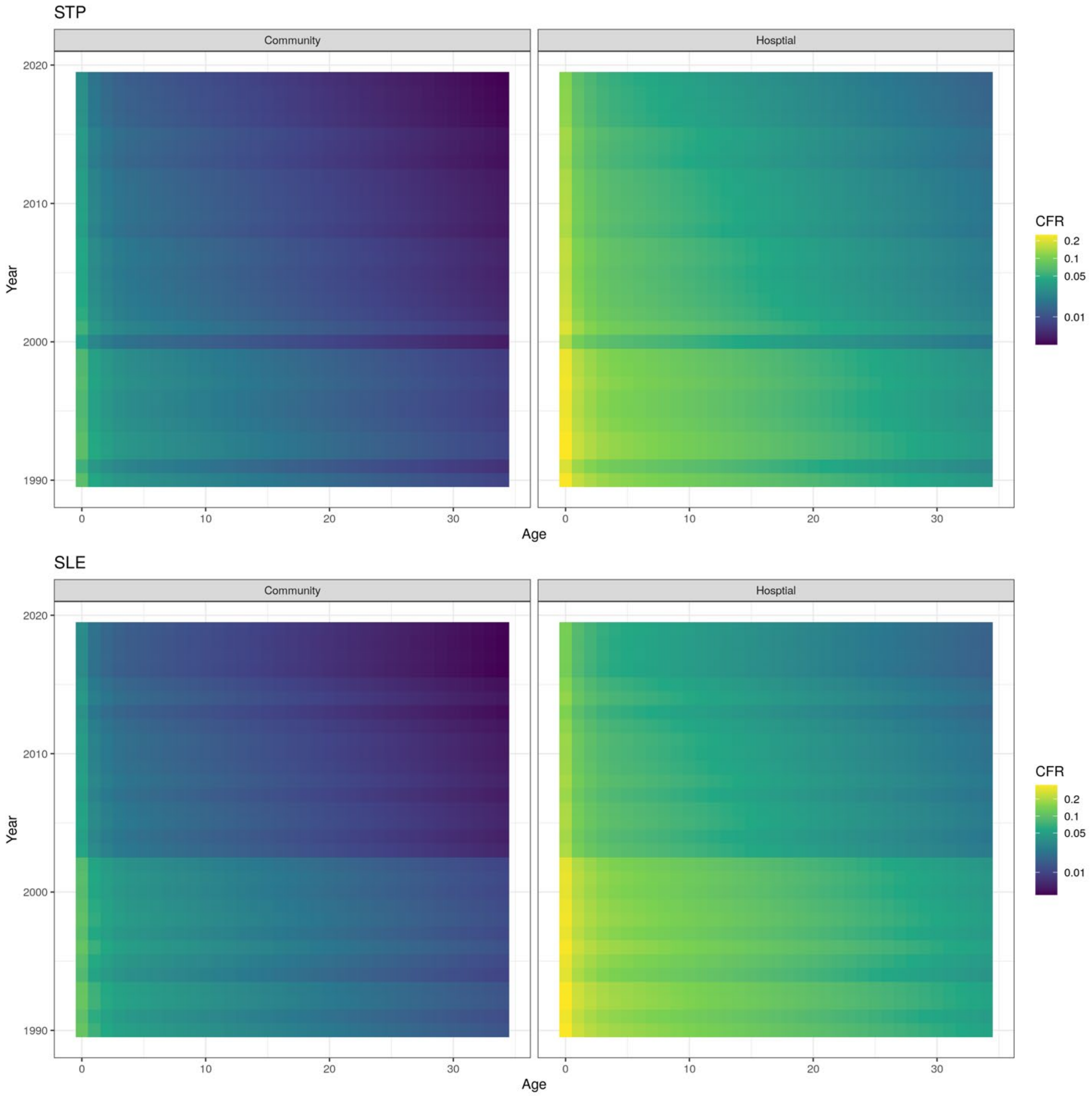
STP
Community
Hosptial
2020
2010
2000
1990
Year
0
10
20
30
Age
CFR
0.2
0.1
0.05
0.01
SLE
Community
Hosptial
2020
2010
2000
1990
Year
0
10
20
30
Age
CFR
0.2
0.1
0.05
0.01

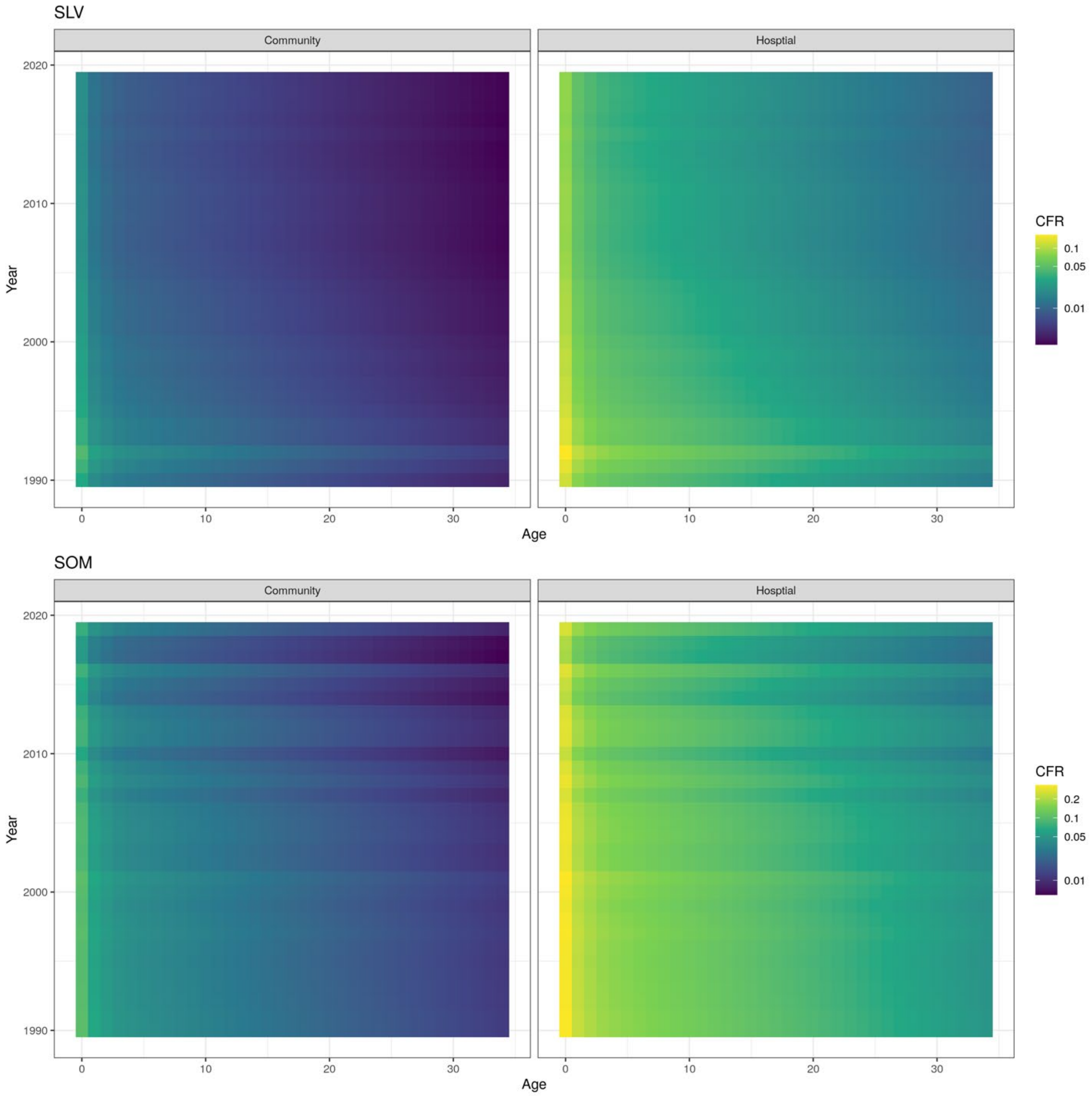

SLV
Community
Hosptial
2020
2010
2000
1990
Year
0
10
20
30
Age
CFR
0.1
0.05
0.01
SOM
Community
Hosptial
2020
2010
2000
1990
Year
0
10
20
30
Age
CFR
0.2
0.1
0.05
0.01

SEN

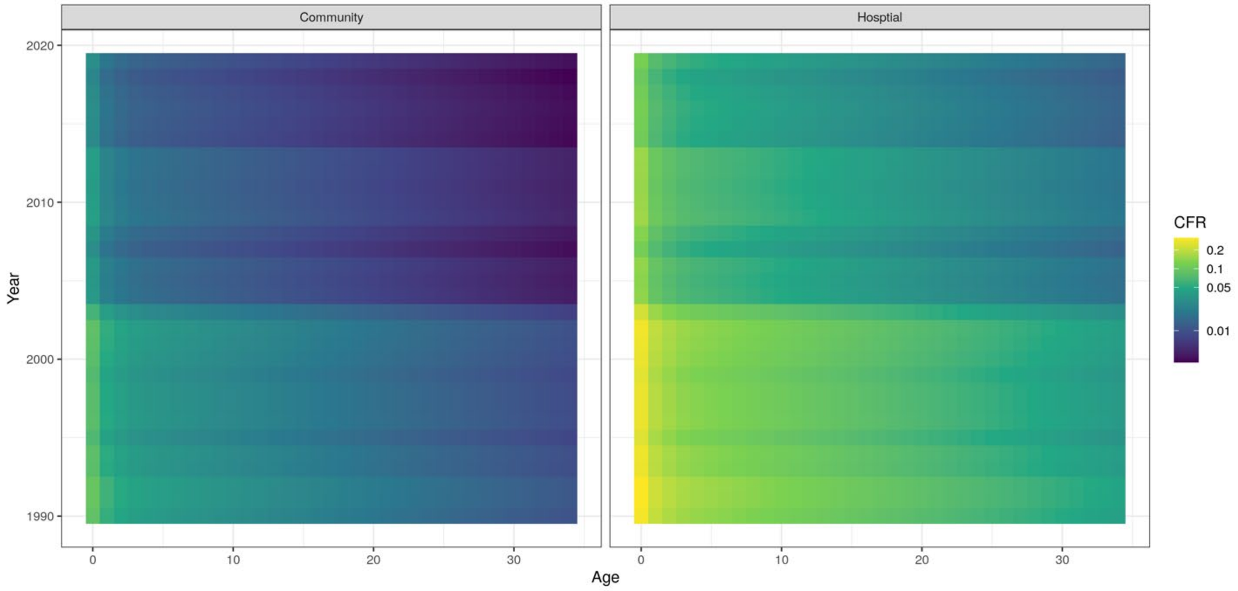
Community
Hosptial
2020
2010
2000
1990
Year
0
10
20
30
Age
CFR
0.2
0.1
0.05
0.01

SLB

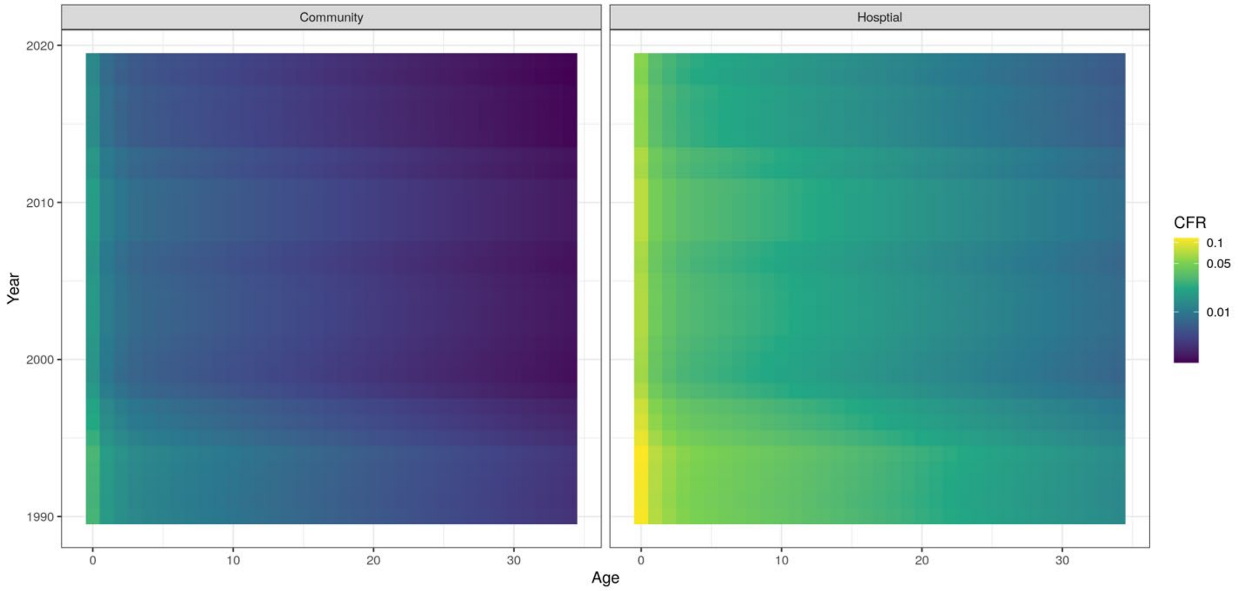
Community
Hosptial
2020
2010
2000
1990
Year
0
10
20
30
Age
CFR
0.1
0.05
0.01

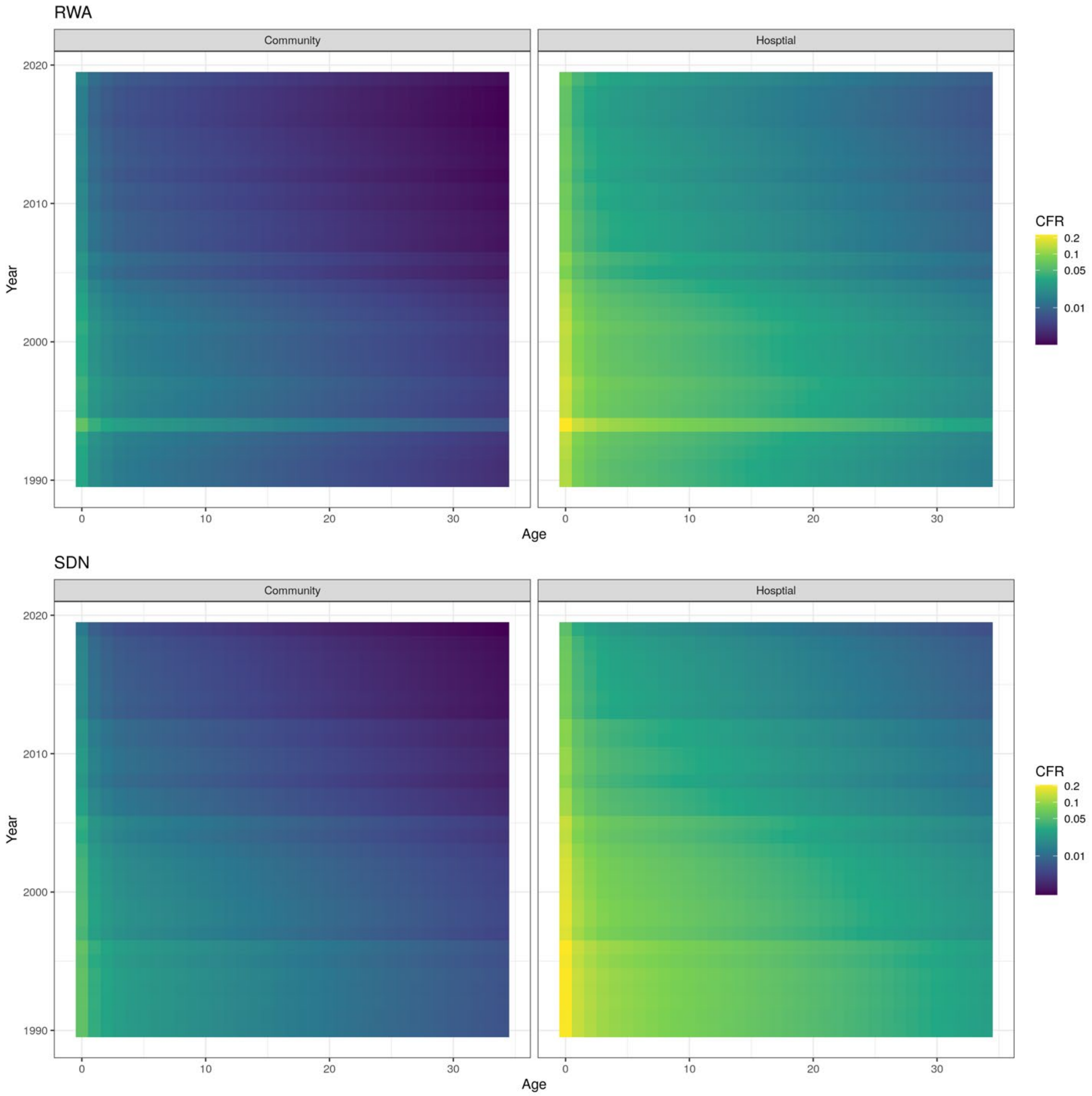
RWA
Community
Hosptial
2020
2010
2000
1990
Year
0
10
20
30
Age
CFR
0.2
0.1
0.05
0.01
SDN
Community
Hosptial
2020
2010
2000
1990
Year
0
10
20
30
Age
CFR
0.2
0.1
0.05
0.01

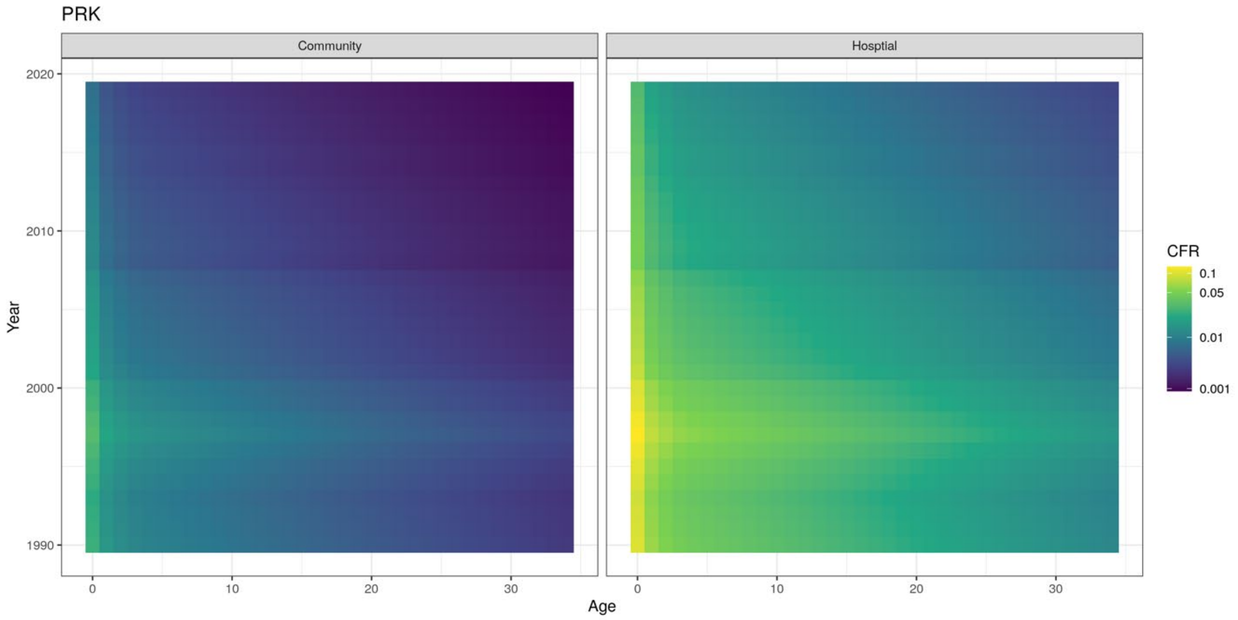
PRK
Community
Hosptial
Year
Age
CFR
0.1
0.05
0.01
0.001

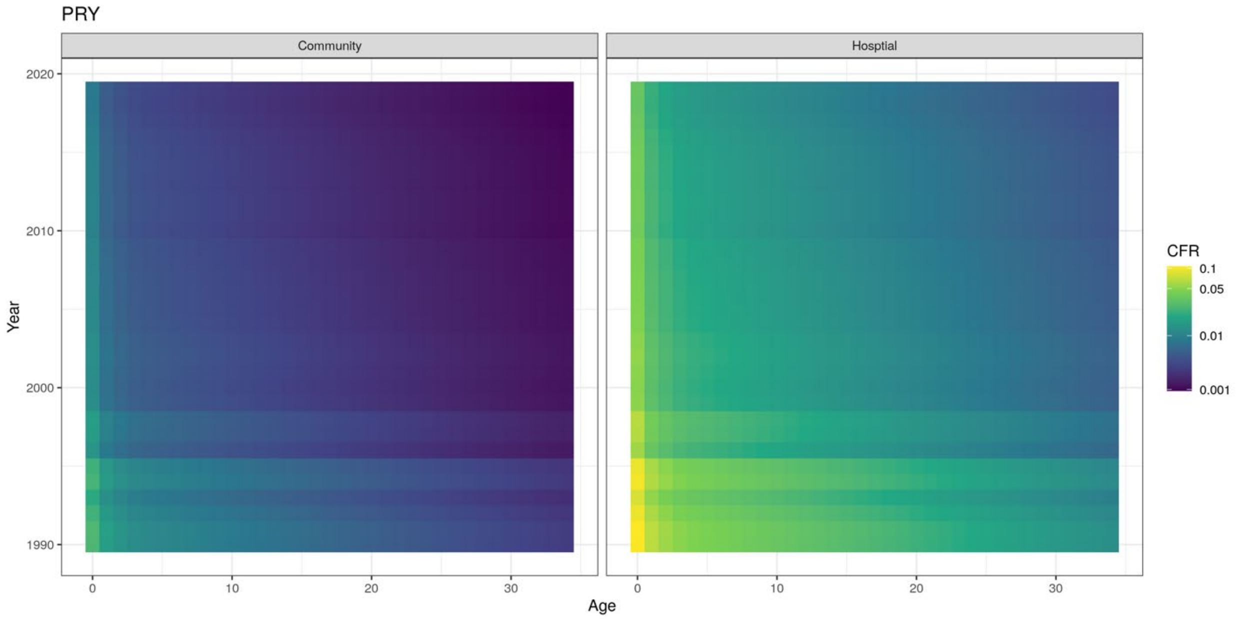
PRY
Community
Hosptial
Year
Age
CFR
0.1
0.05
0.01
0.001

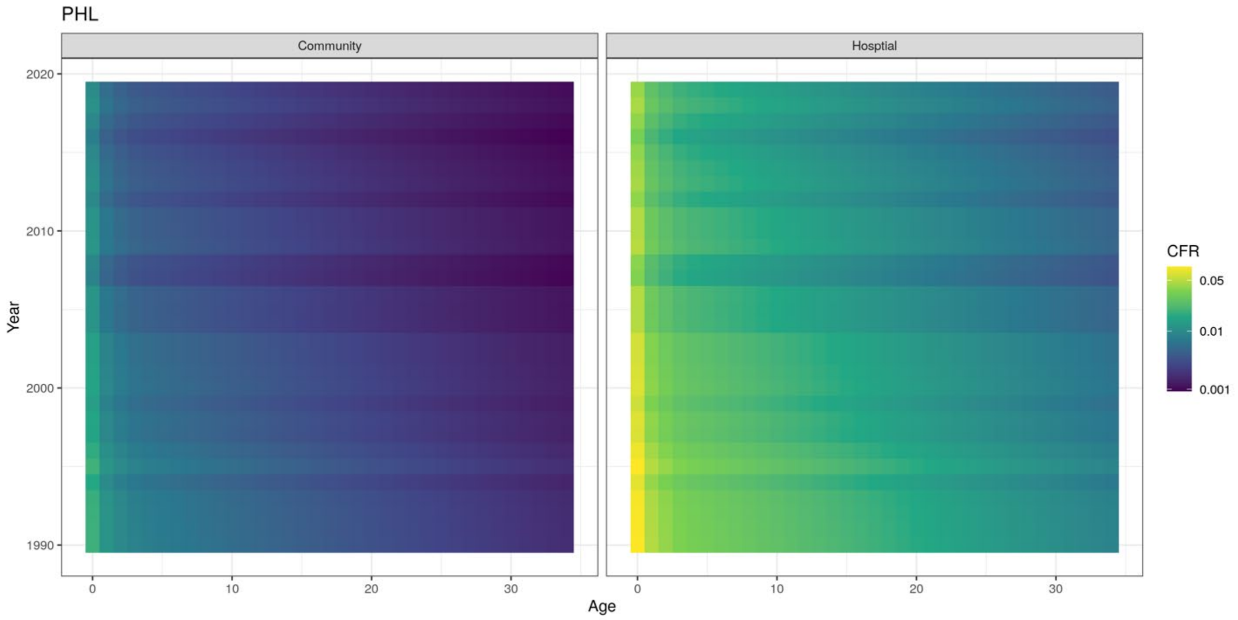
PHL
Community
Hosptial
Year
2020
2010
2000
1990
Age
0
10
20
30
CFR
0.05
0.01
0.001

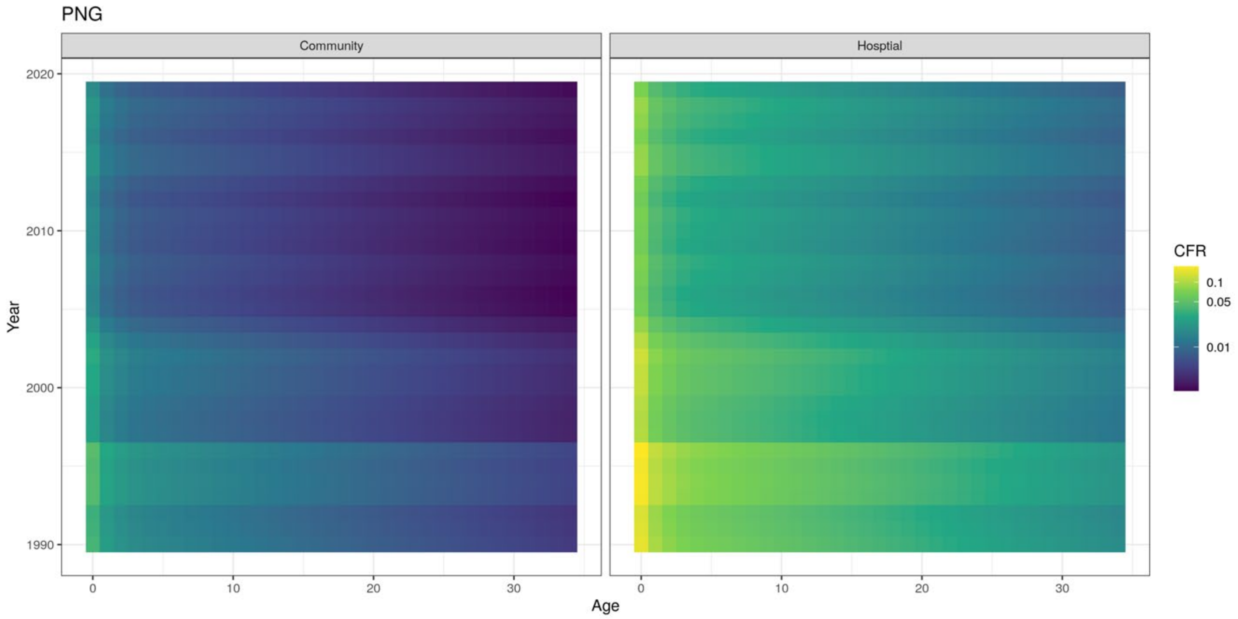
PNG
Community
Hosptial
Year
2020
2010
2000
1990
Age
0
10
20
30
CFR
0.1
0.05
0.01

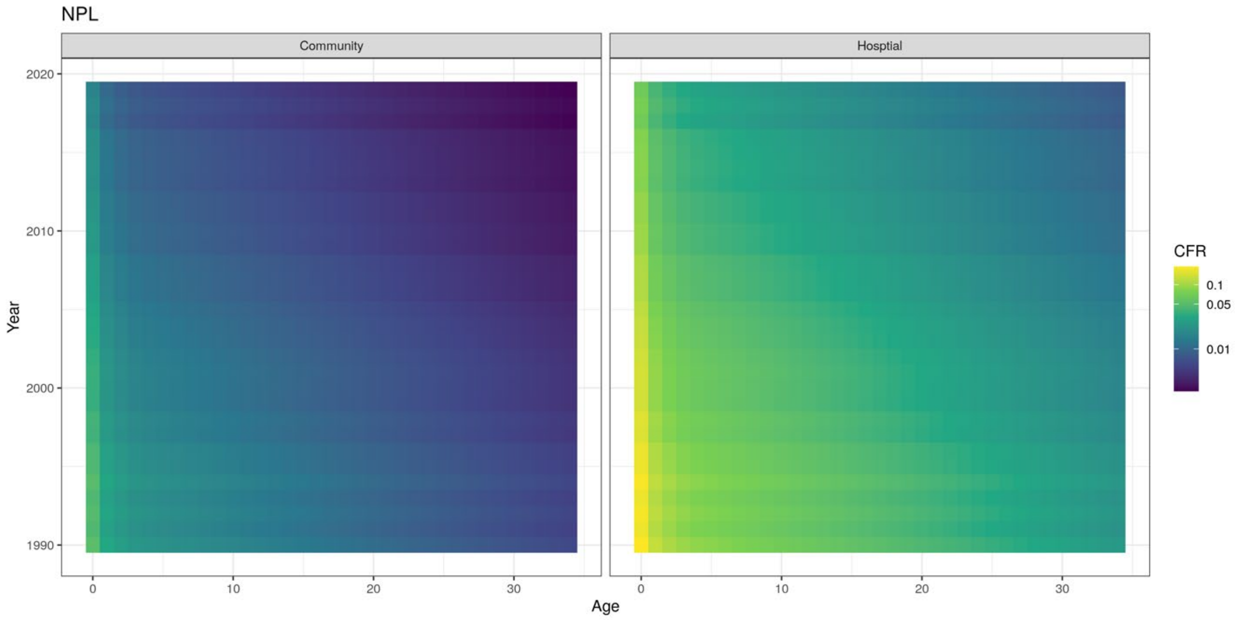
NPL
Community
Hosptial
Year
Age
CFR
0.1
0.05
0.01
2020
2010
2000
1990
0
10
20
30

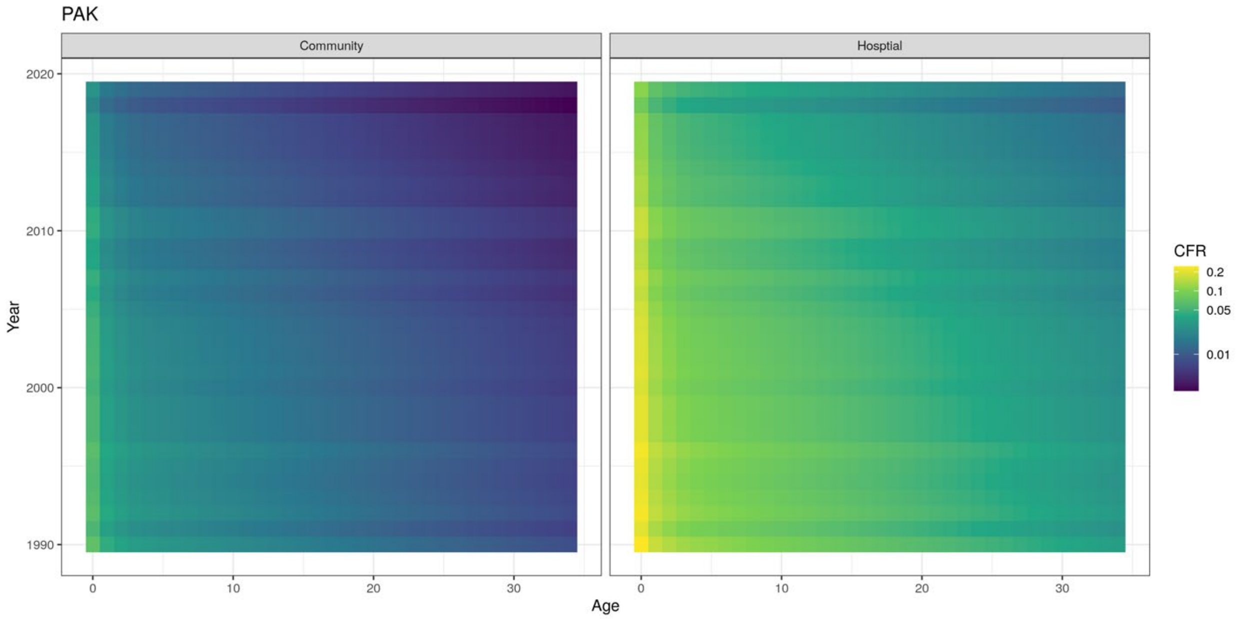
PAK
Community
Hosptial
Year
Age
CFR
0.2
0.1
0.05
0.01
2020
2010
2000
1990
0
10
20
30

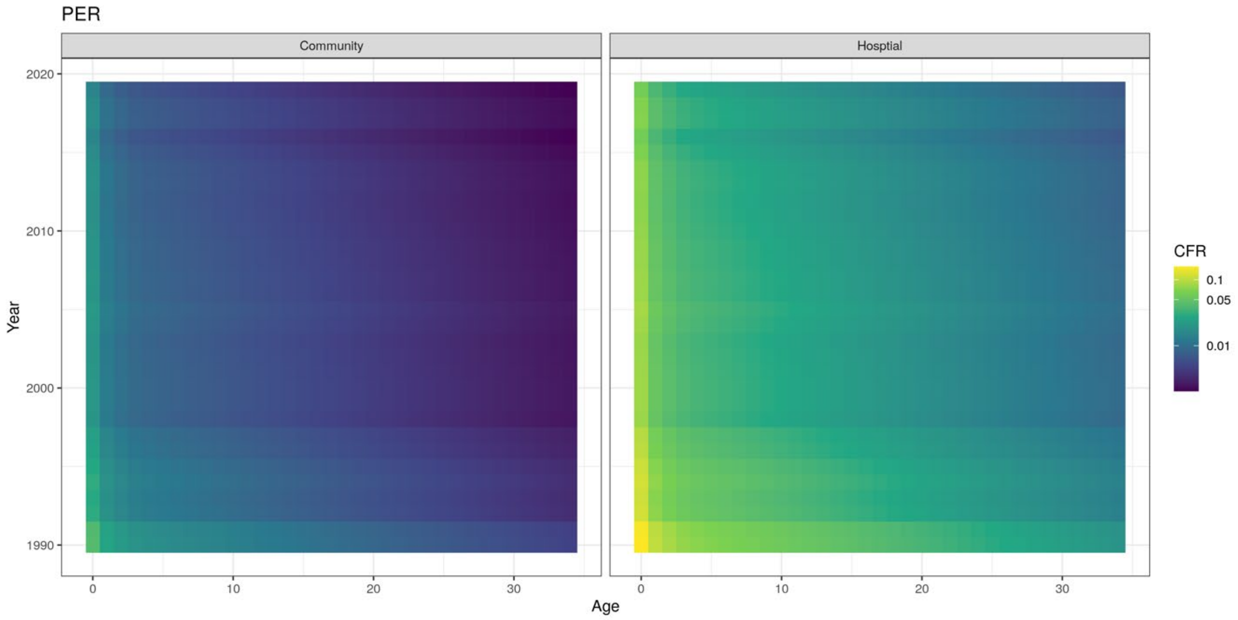
PER
Community
Hosptial
Year
Age
CFR
0.1
0.05
0.01
2020
2010
2000
1990
0
10
20
30

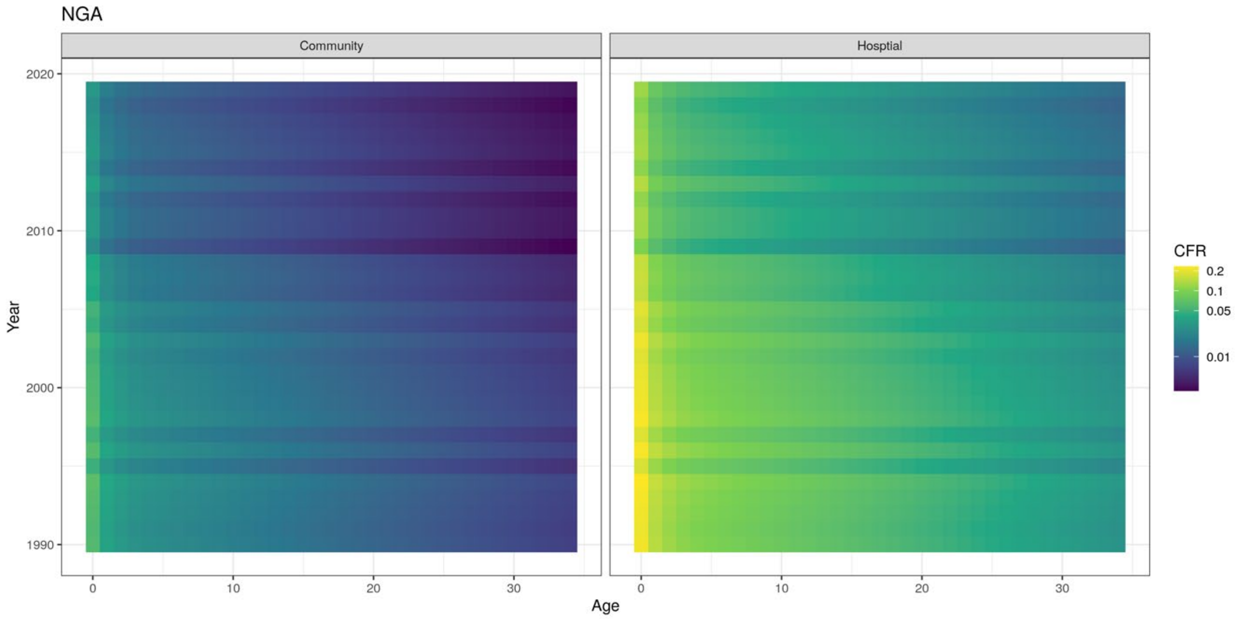
NGA
Community
Hosptial
Year
Age
CFR
0.2
0.1
0.05
0.01
2020
2010
2000
1990
0
10
20
30

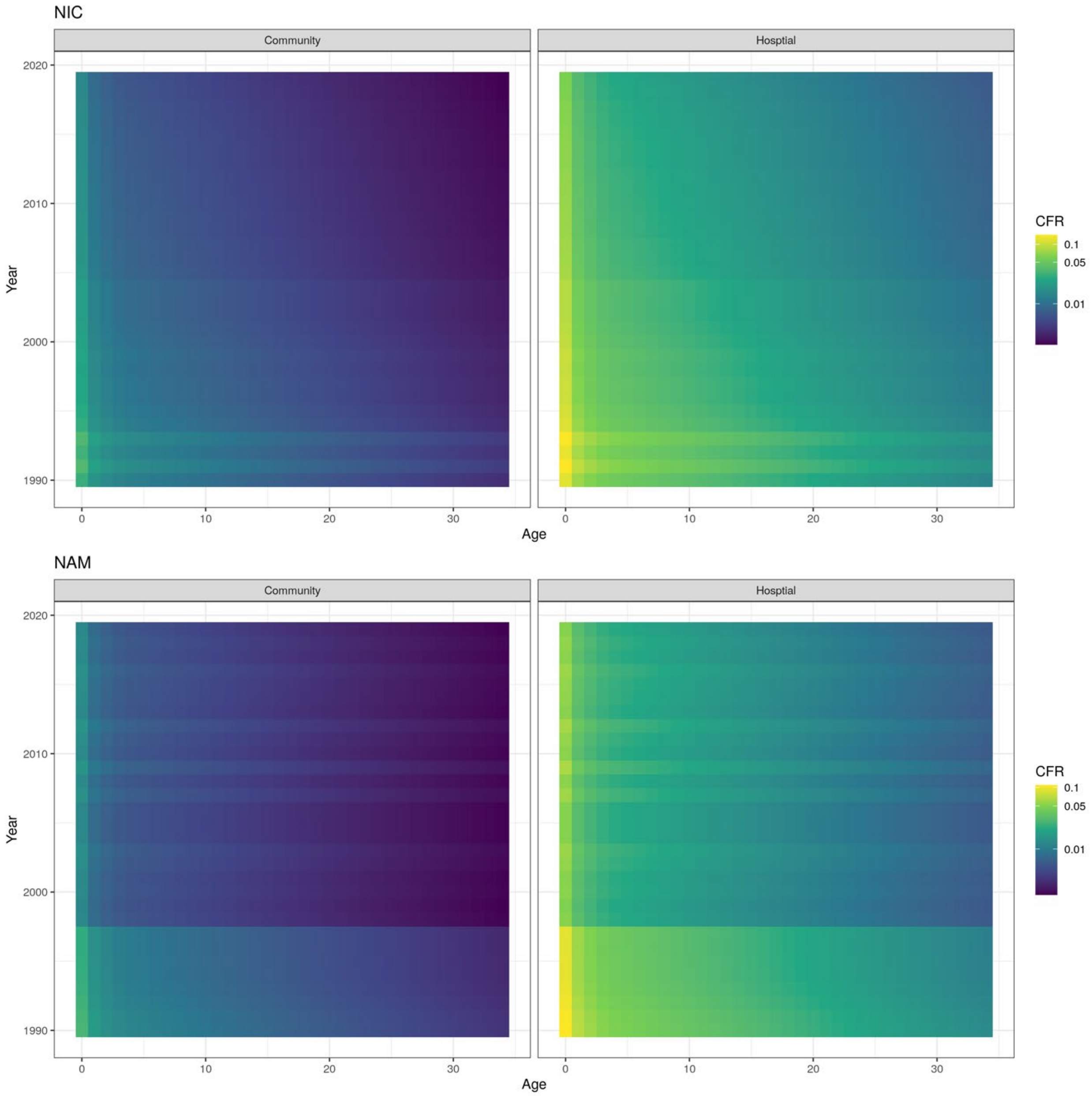
NIC
Community
Hosptial
Year
Age
CFR
0.1
0.05
0.01
NAM
Community
Hosptial
Year
Age
CFR
0.1
0.05
0.01

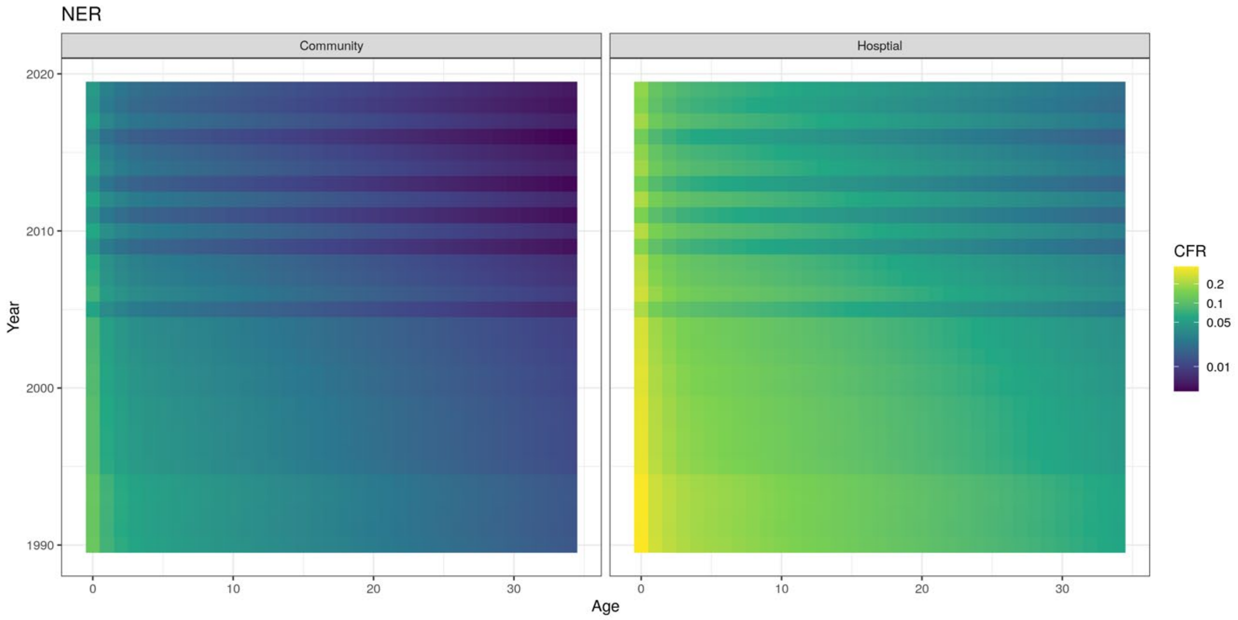
NER
Community
Hosptial
Year
Age
CFR
0.2
0.1
0.05
0.01

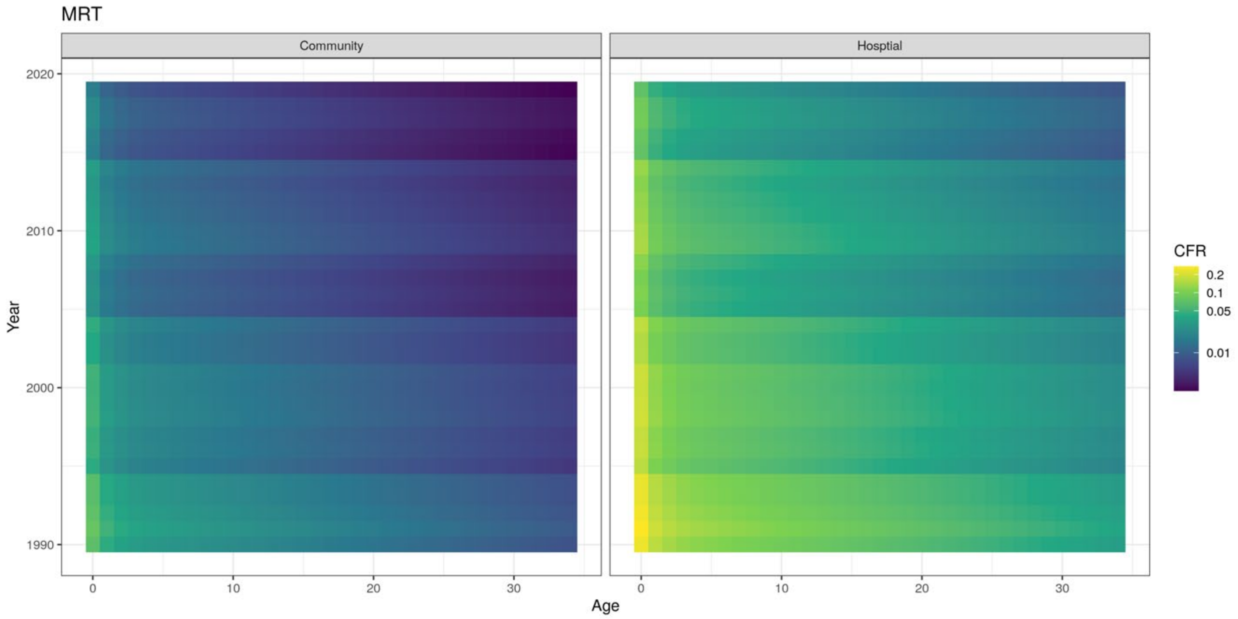
MRT
Community
Hosptial
Year
Age
CFR
0.2
0.1
0.05
0.01

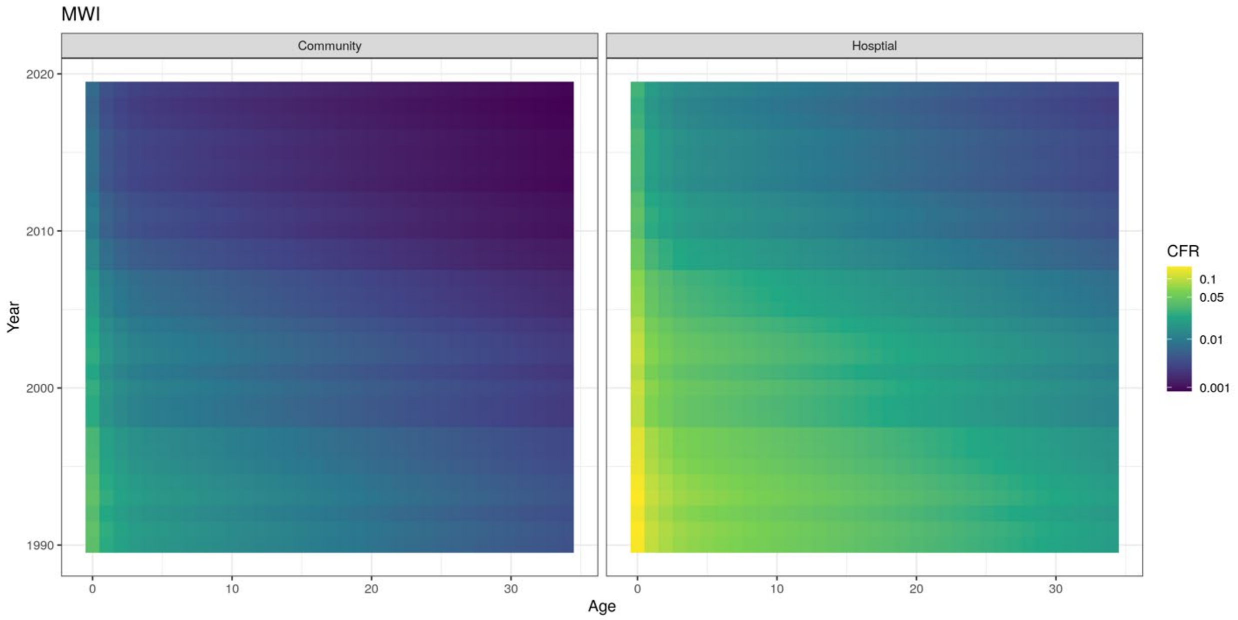
MWI
Community
Hosptial
Year
2020
2010
2000
1990
Age
0
10
20
30
CFR
0.1
0.05
0.01
0.001

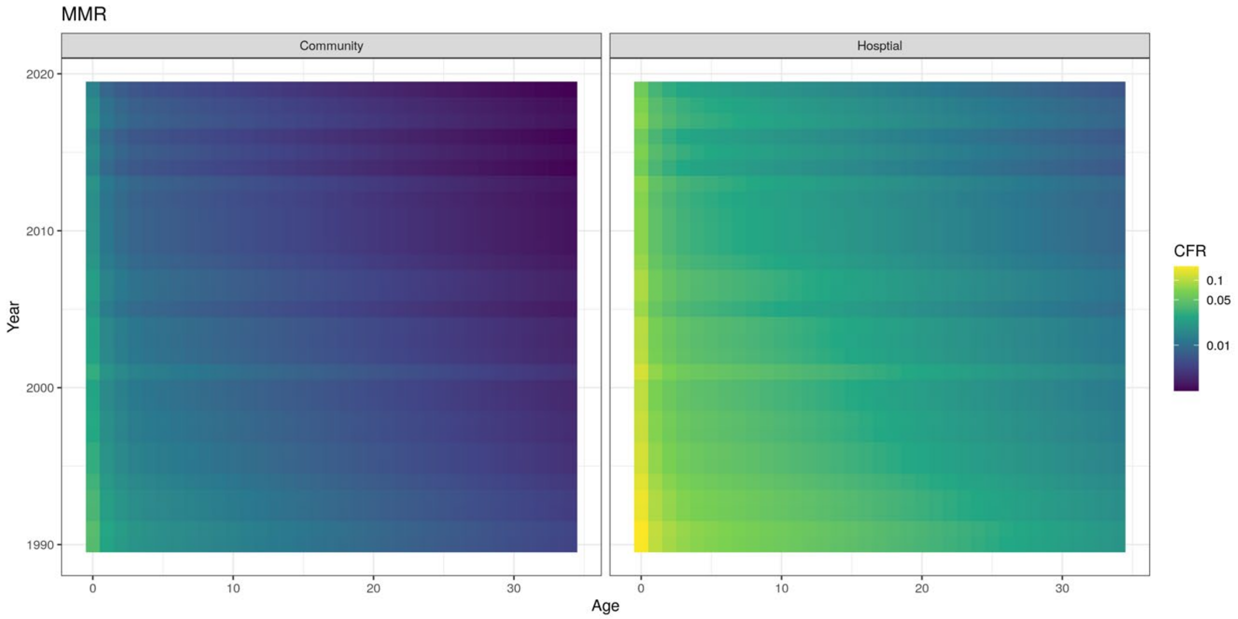
MMR
Community
Hosptial
Year
2020
2010
2000
1990
Age
0
10
20
30
CFR
0.1
0.05
0.01

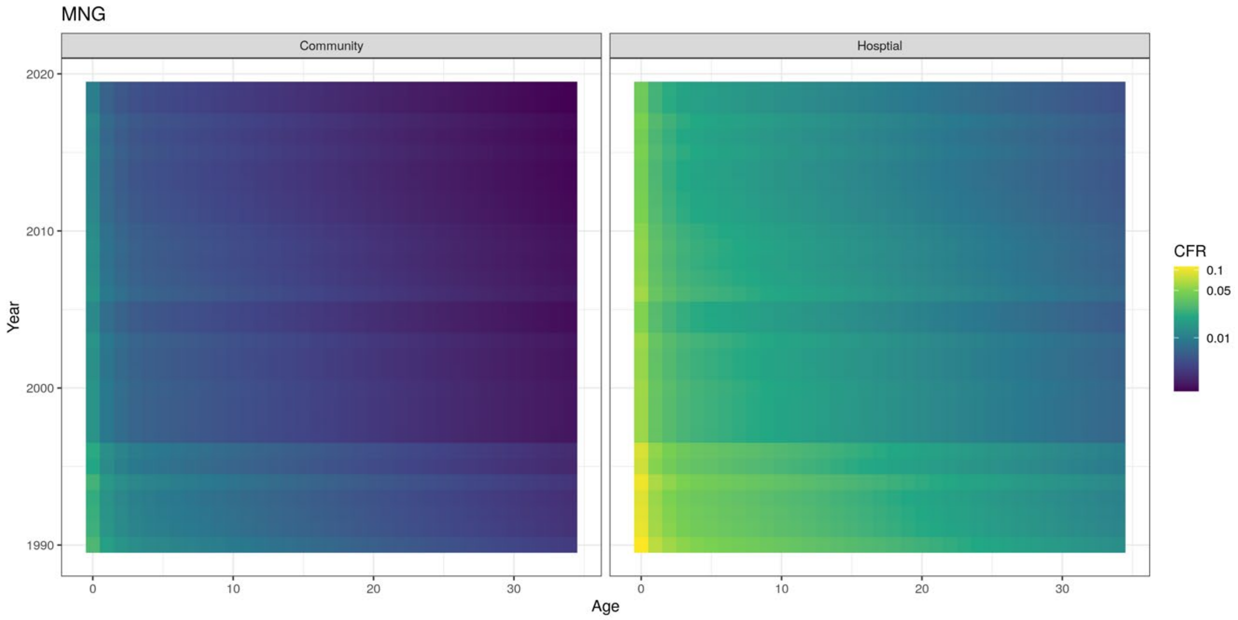
MNG
Community
Hosptial
Year
Age
CFR
0.1
0.05
0.01
2020
2010
2000
1990
0
10
20
30

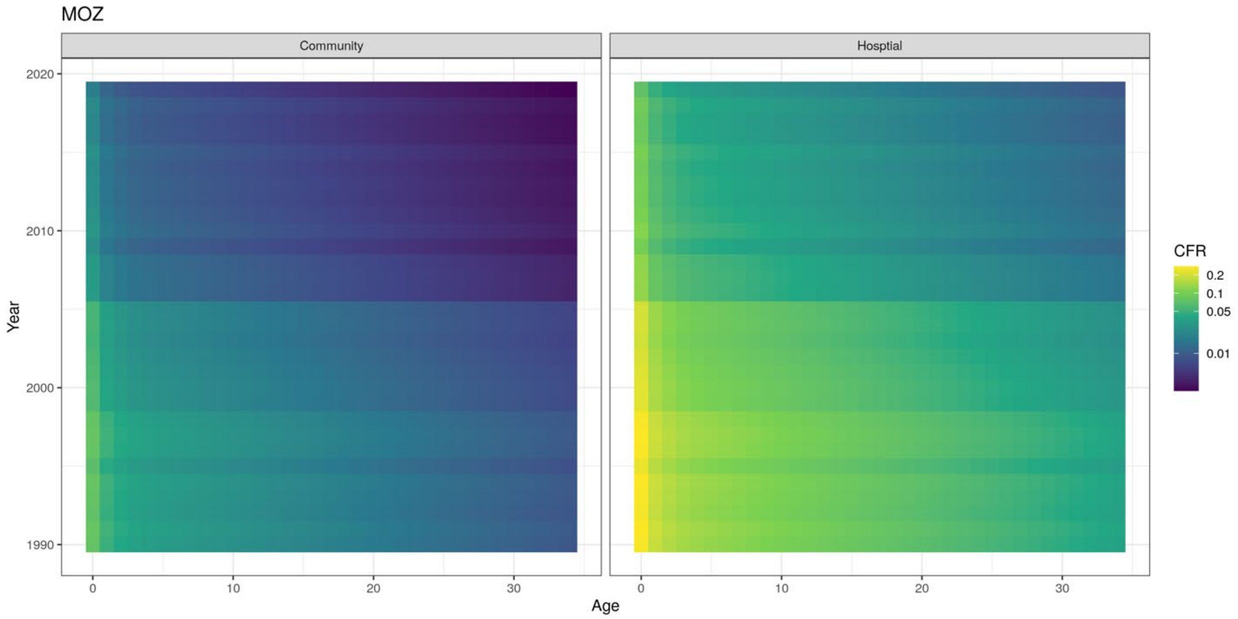
MOZ
Community
Hosptial
Year
Age
CFR
0.2
0.1
0.05
0.01
2020
2010
2000
1990
0
10
20
30

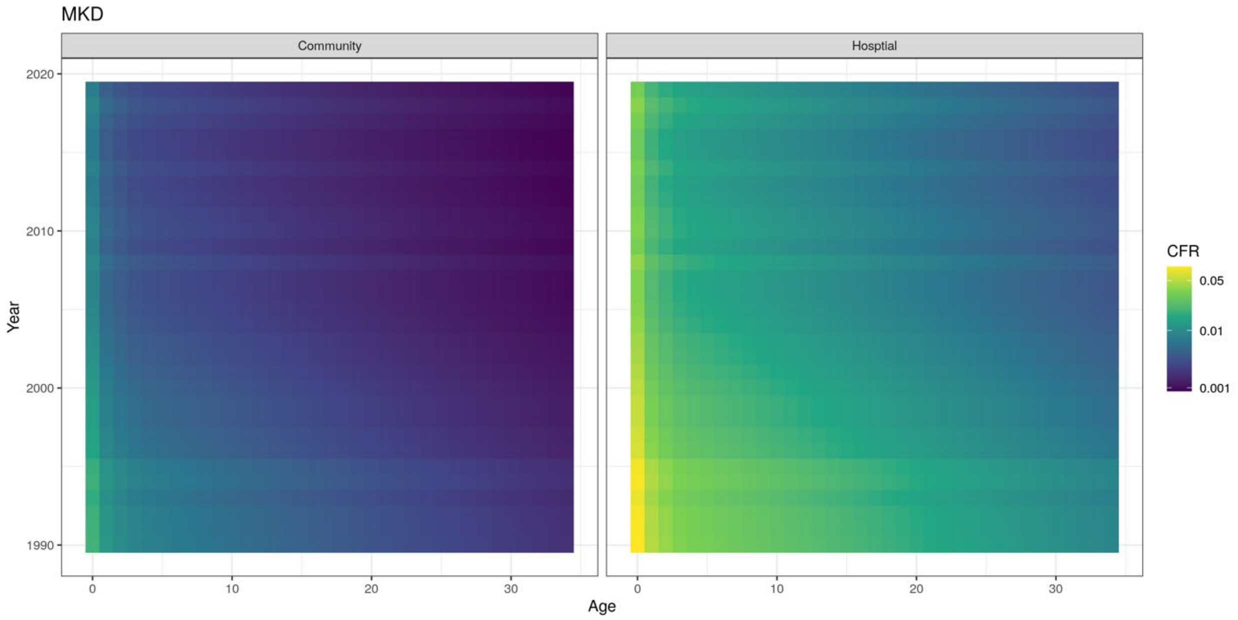
MKD
Community
Hosptial
Year
2020
2010
2000
1990
0
10
20
30
Age
CFR
0.05
0.01
0.001

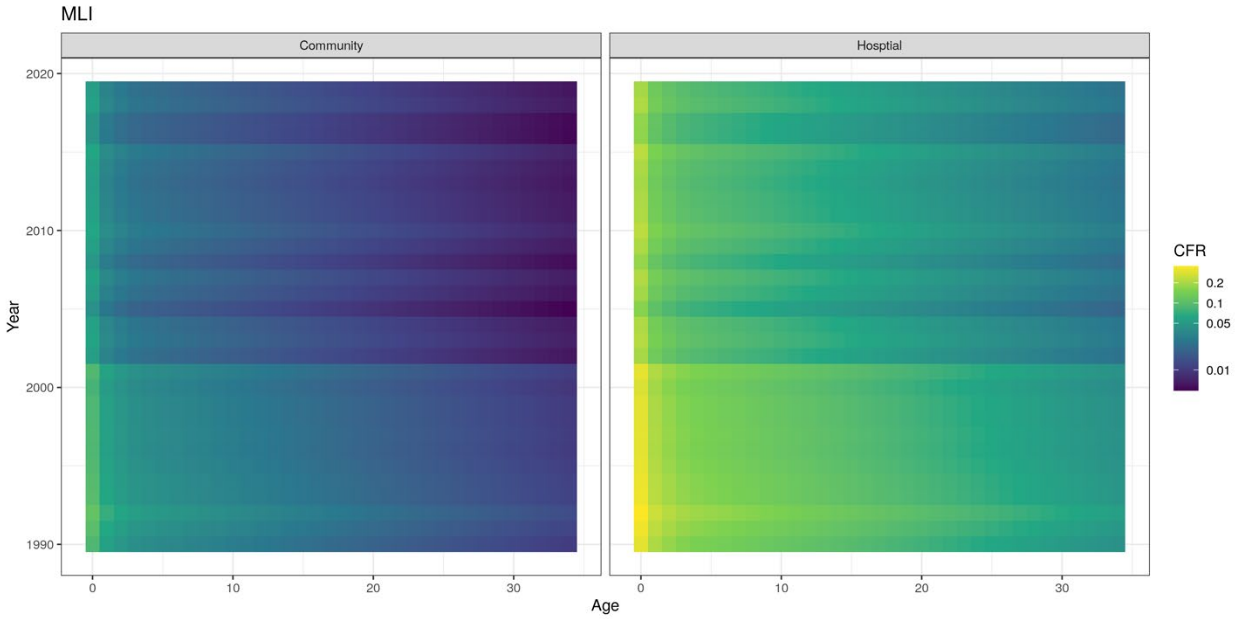
MLI
Community
Hosptial
Year
2020
2010
2000
1990
0
10
20
30
Age
CFR
0.2
0.1
0.05
0.01

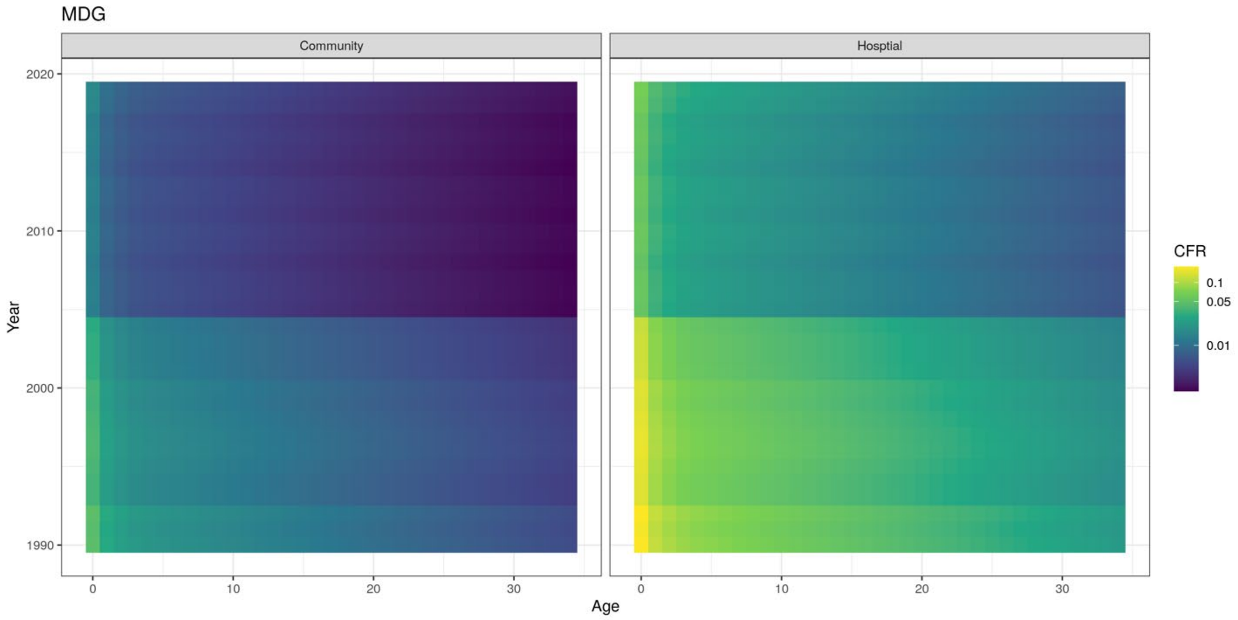
MDG
Community
Hosptial
2020
2010
2000
1990
Year
0
10
20
30
Age
CFR
0.1
0.05
0.01

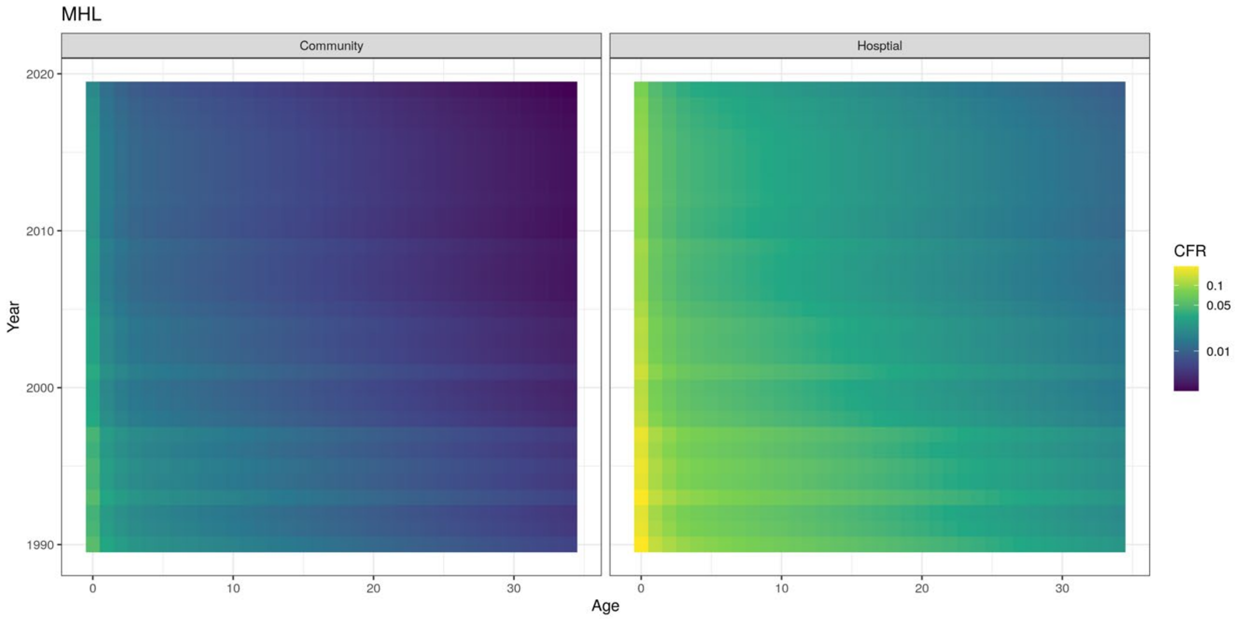
MHL
Community
Hosptial
2020
2010
2000
1990
Year
0
10
20
30
Age
CFR
0.1
0.05
0.01

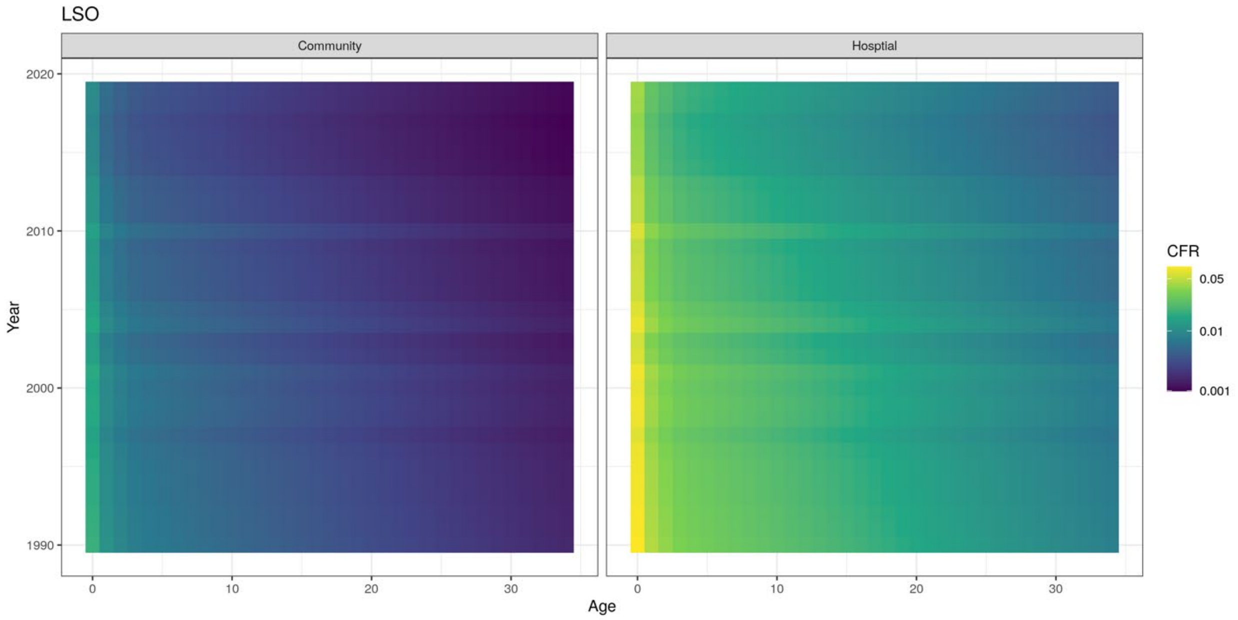
LSO
Community
Hosptial
Year
Age
CFR
0.05
0.01
0.001

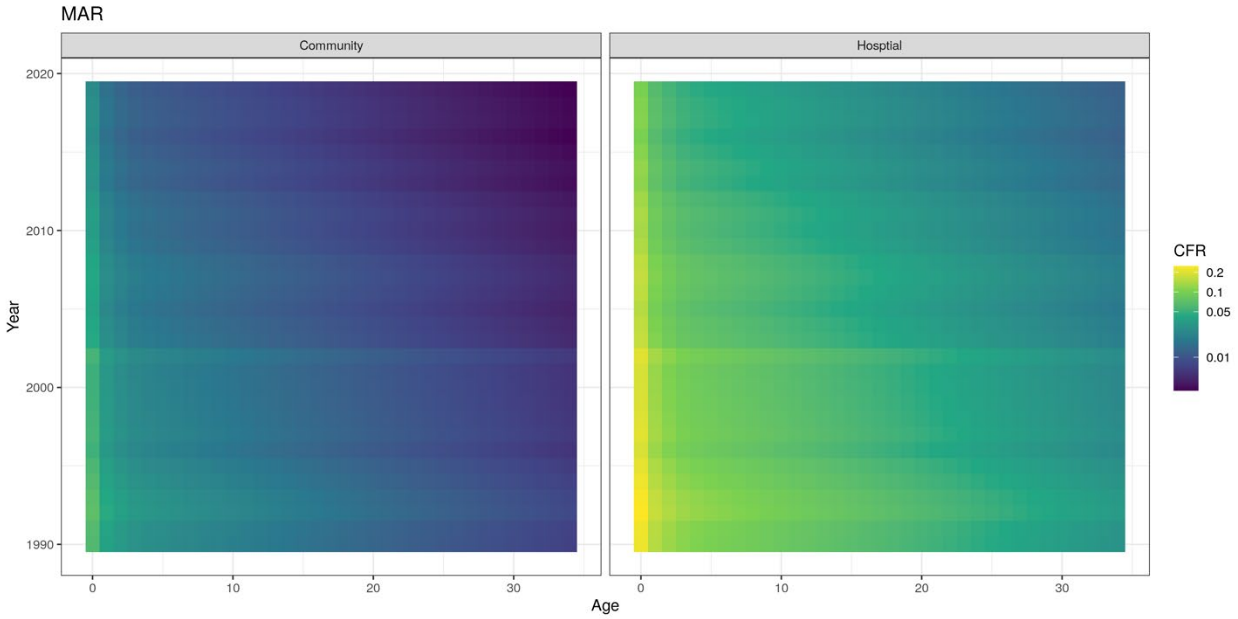
MAR
Community
Hosptial
Year
Age
CFR
0.2
0.1
0.05
0.01

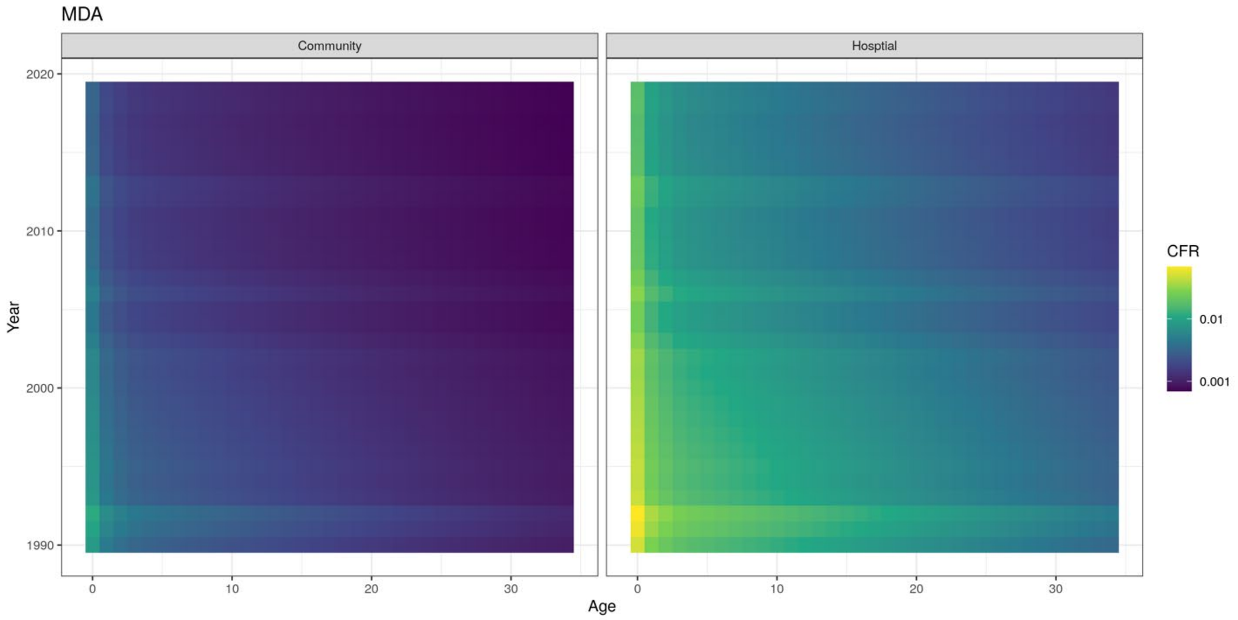
MDA
Community
Hosptial
Year
2020
2010
2000
1990
Age
0
10
20
30
CFR
0.01
0.001

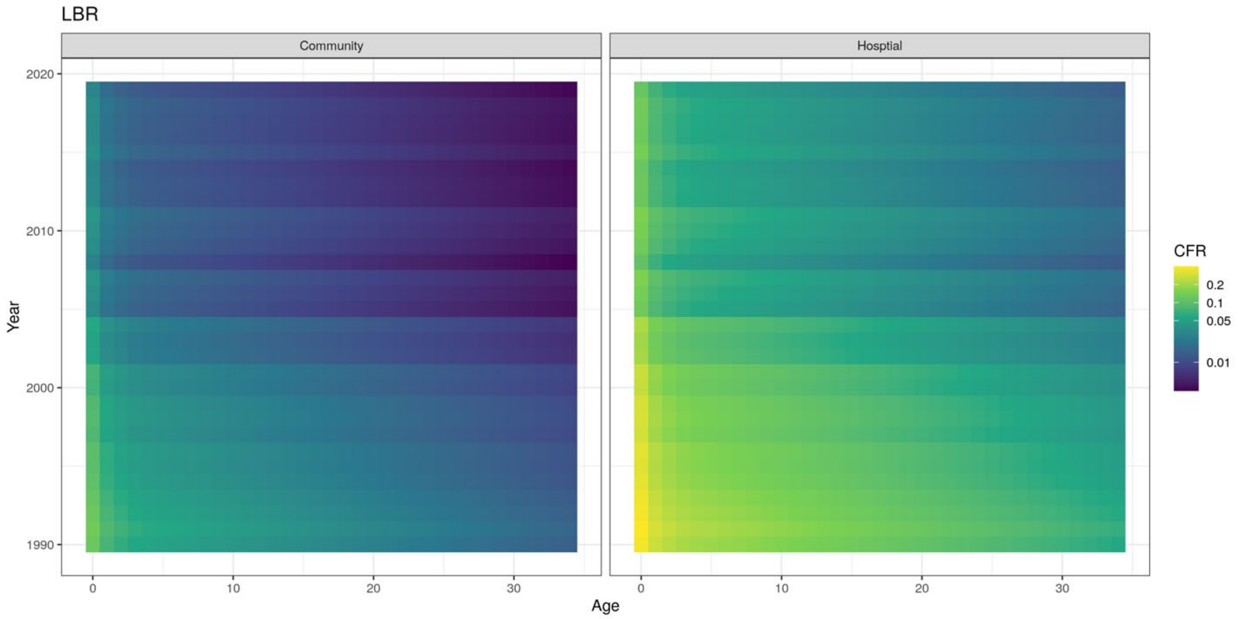
LBR
Community
Hosptial
Year
2020
2010
2000
1990
Age
0
10
20
30
CFR
0.2
0.1
0.05
0.01

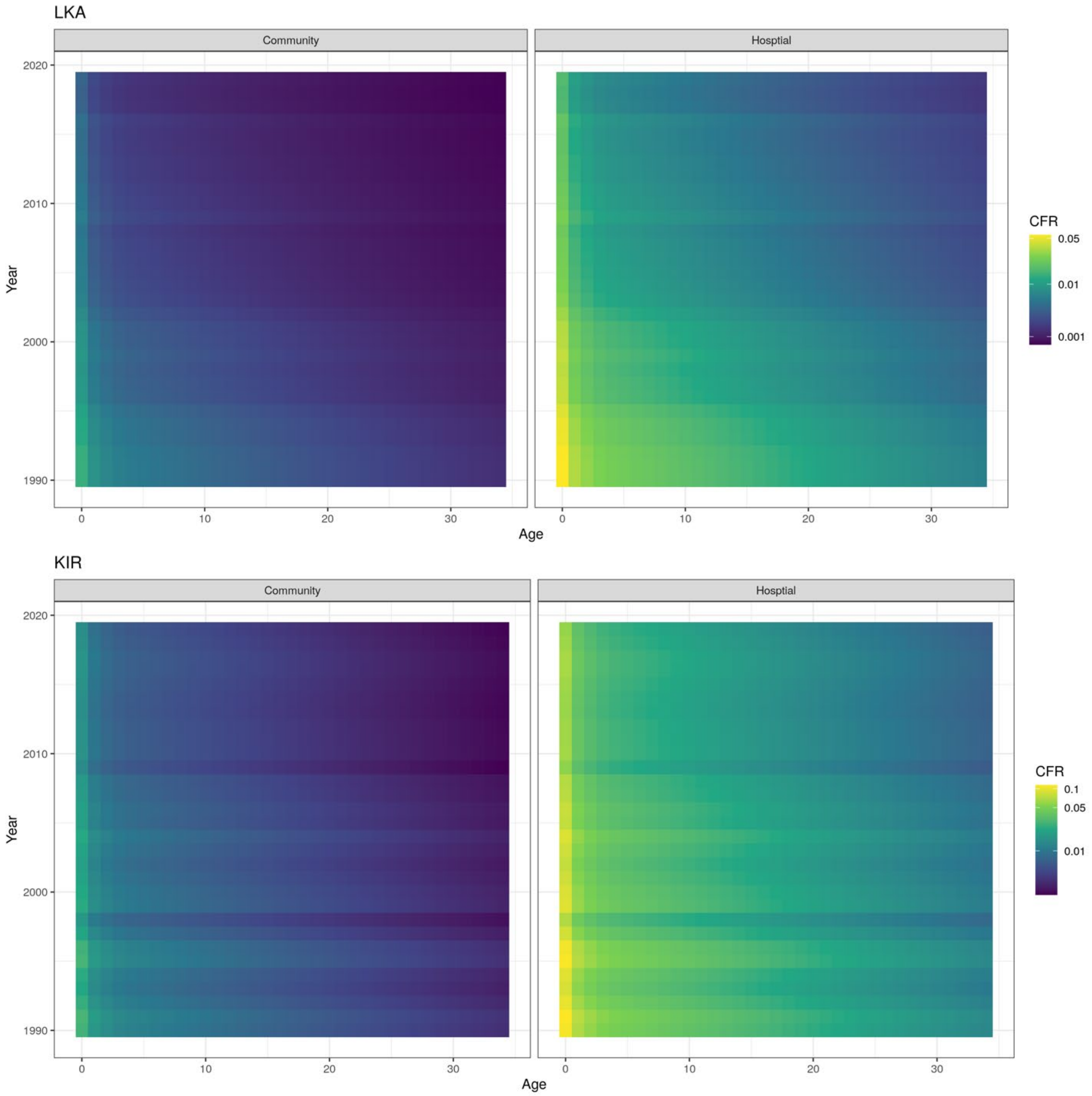
LKA
Community
Hosptial
Year
Age
CFR
0.05
0.01
0.001
KIR
Community
Hosptial
Year
Age
CFR
0.1
0.05
0.01

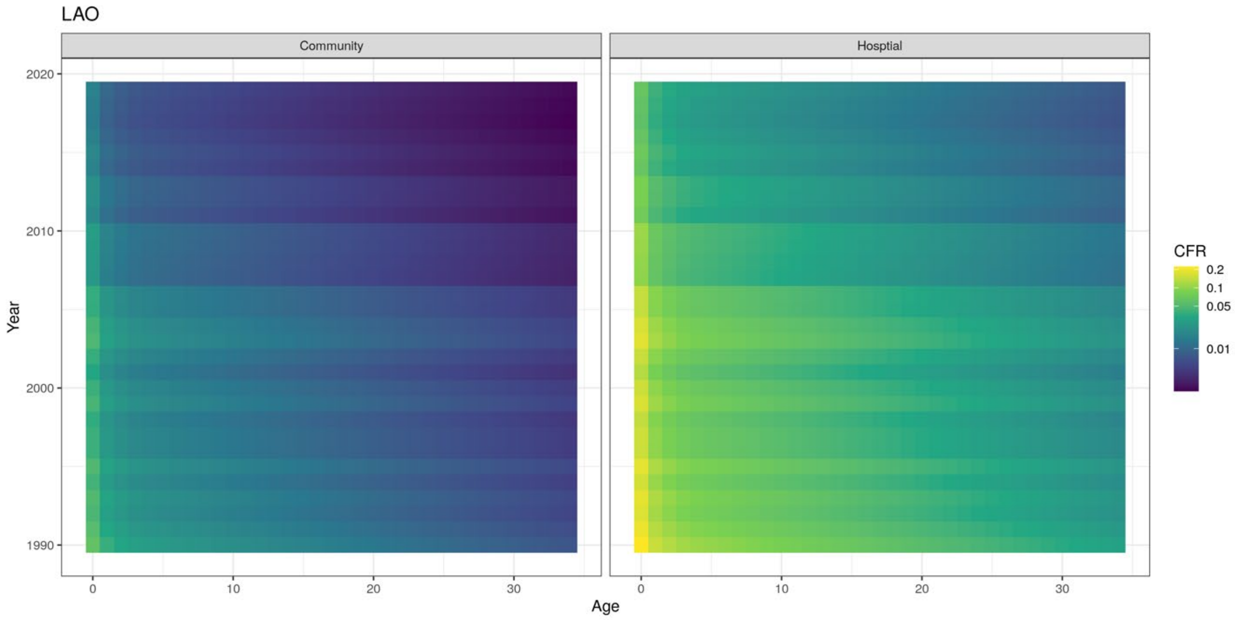
LAO
Community
Hosptial
Year
Age
CFR
0.2
0.1
0.05
0.01
2020
2010
2000
1990
0
10
20
30

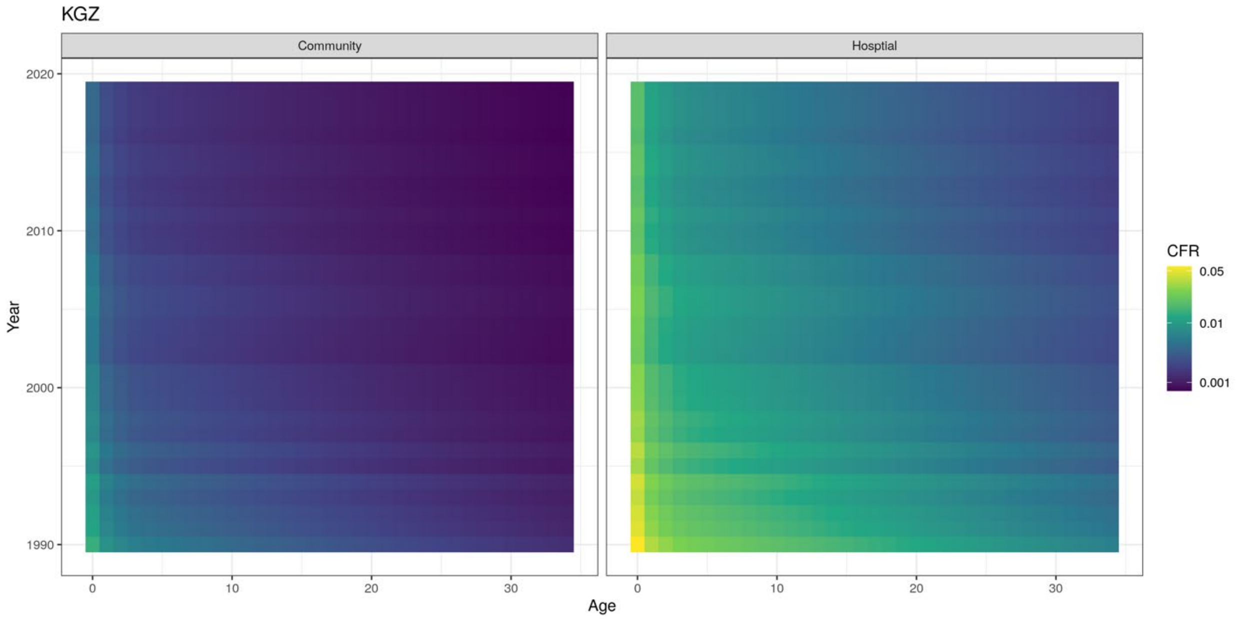
KGZ
Community
Hosptial
Year
Age
CFR
0.05
0.01
0.001
2020
2010
2000
1990
0
10
20
30

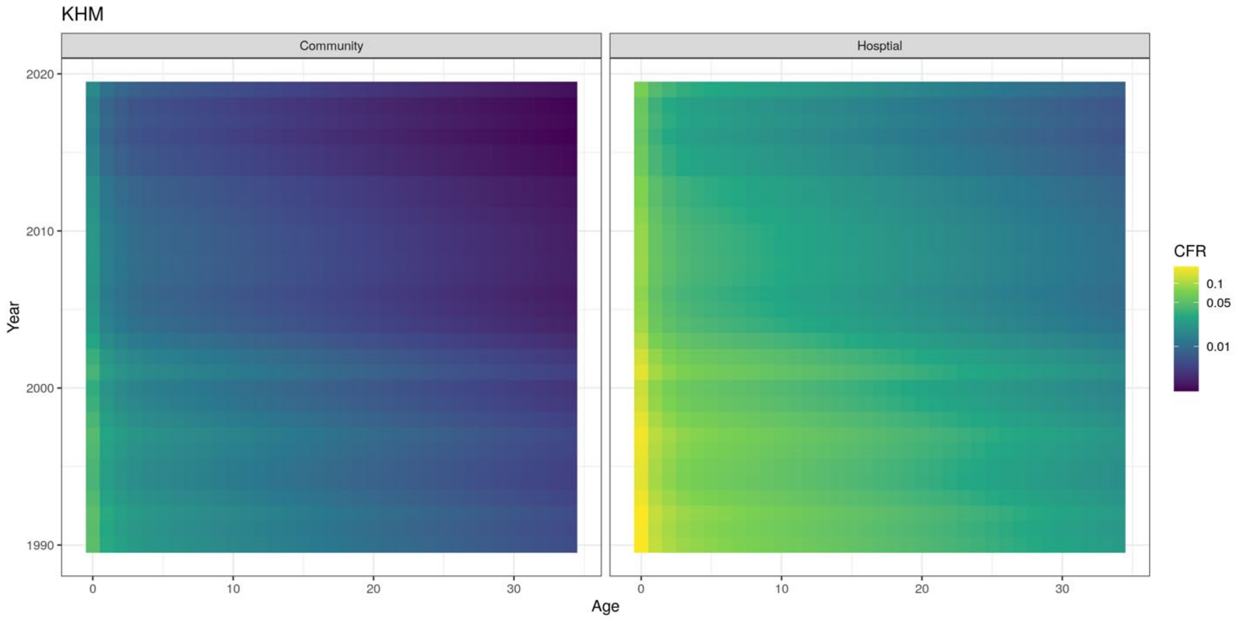
KHM
Community
Hosptial
Year
2020
2010
2000
1990
Age
0
10
20
30
CFR
0.1
0.05
0.01

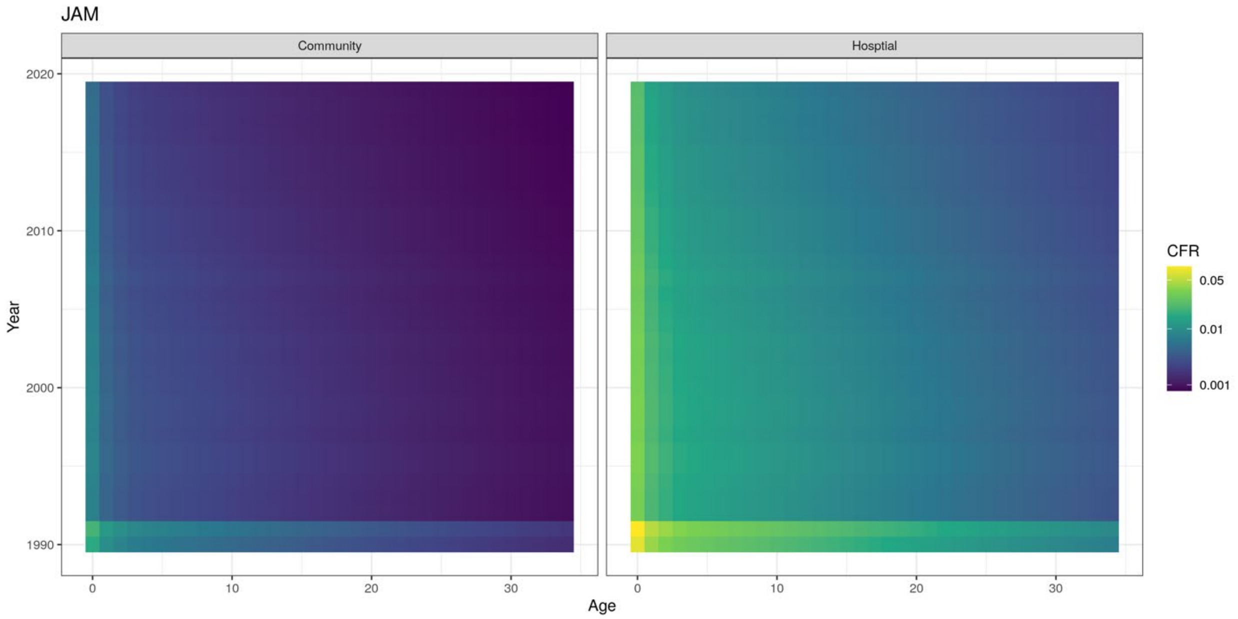
JAM
Community
Hosptial
Year
2020
2010
2000
1990
Age
0
10
20
30
CFR
0.05
0.01
0.001

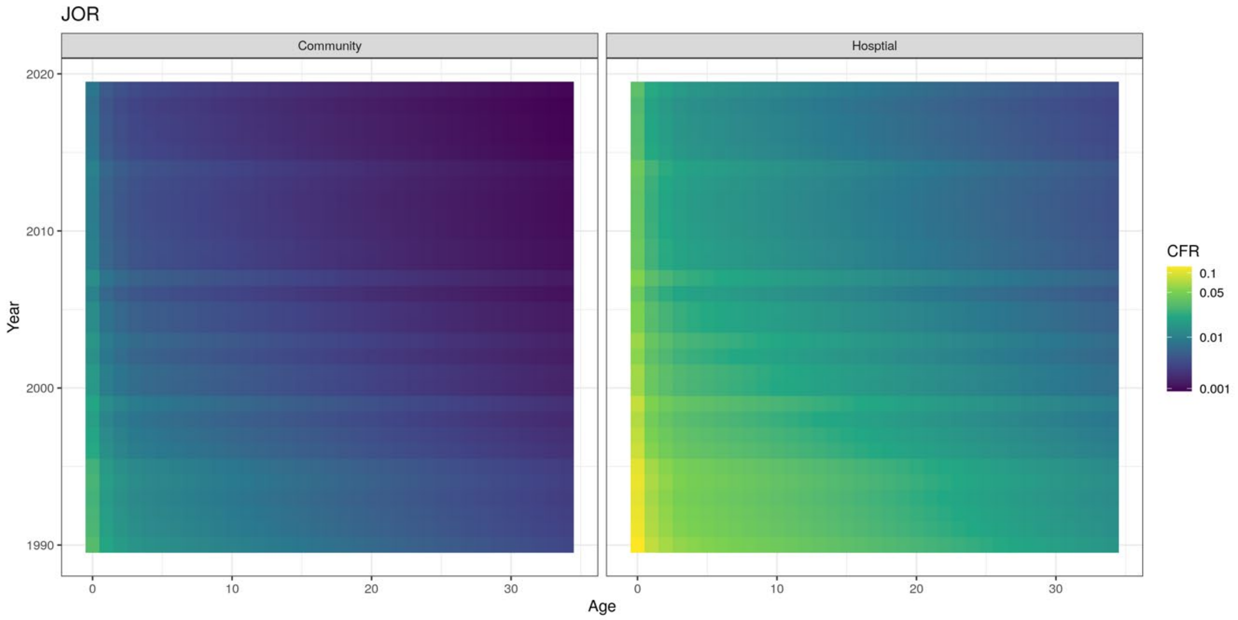
JOR
Community
Hosptial
Year
2020
2010
2000
1990
0
10
20
30
Age
CFR
0.1
0.05
0.01
0.001

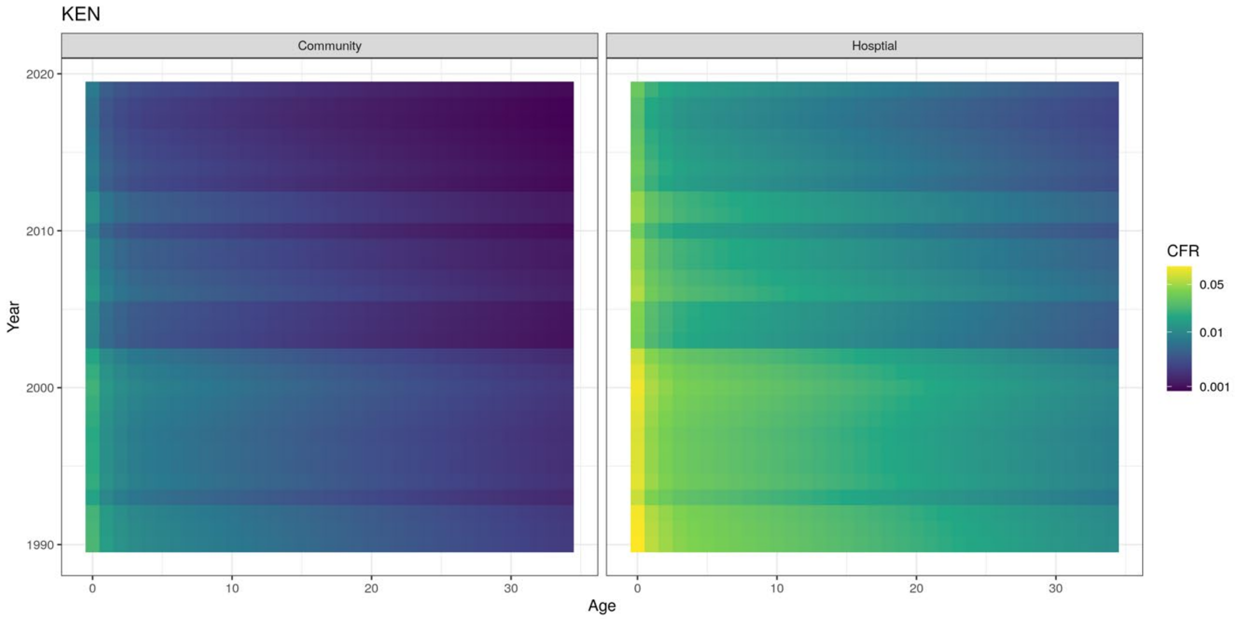
KEN
Community
Hosptial
Year
2020
2010
2000
1990
0
10
20
30
Age
CFR
0.05
0.01
0.001

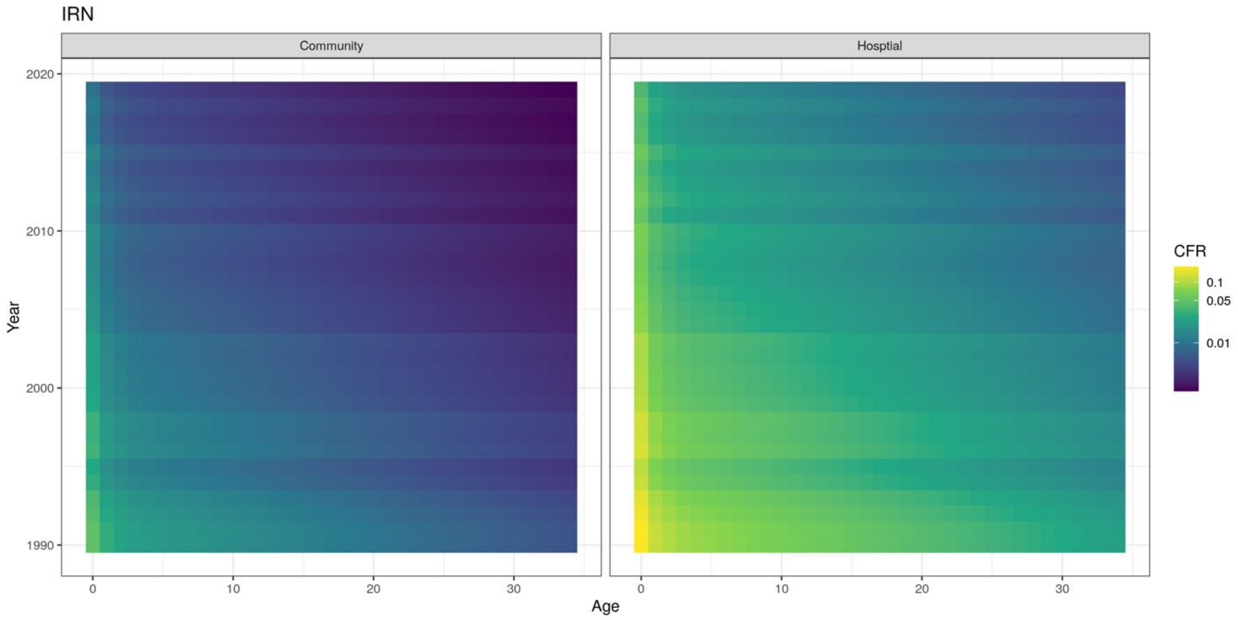
IRN
Community
Hosptial
2020
2010
2000
1990
Year
0
10
20
30
Age
CFR
0.1
0.05
0.01

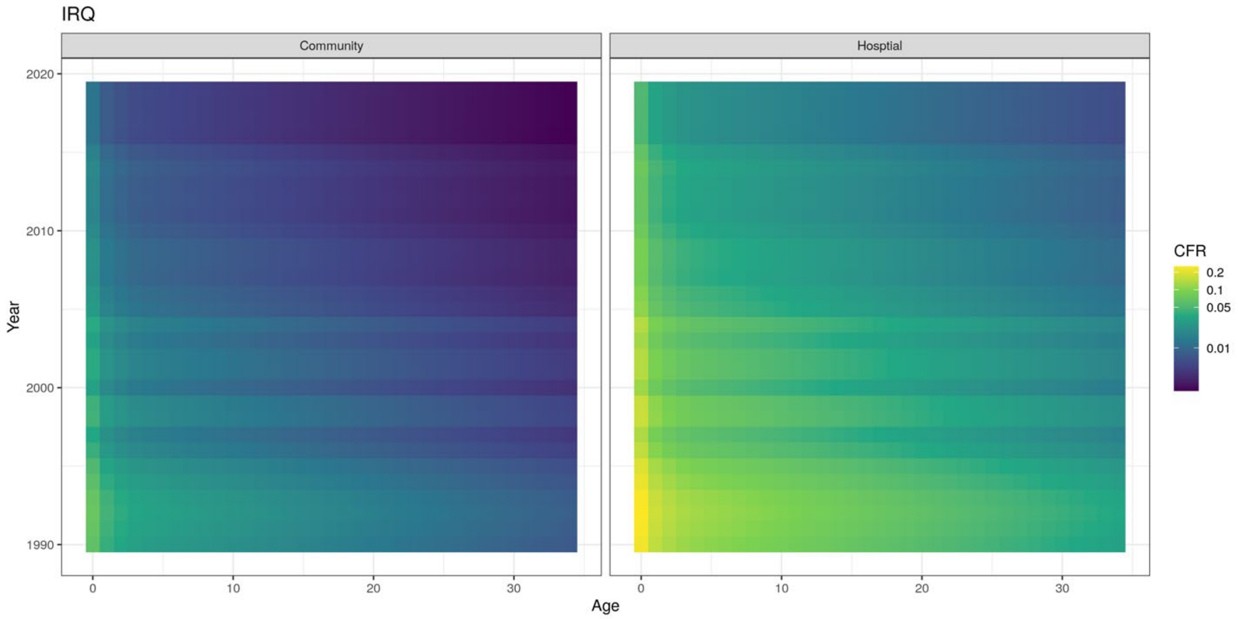
IRQ
Community
Hosptial
2020
2010
2000
1990
Year
0
10
20
30
Age
CFR
0.2
0.1
0.05
0.01

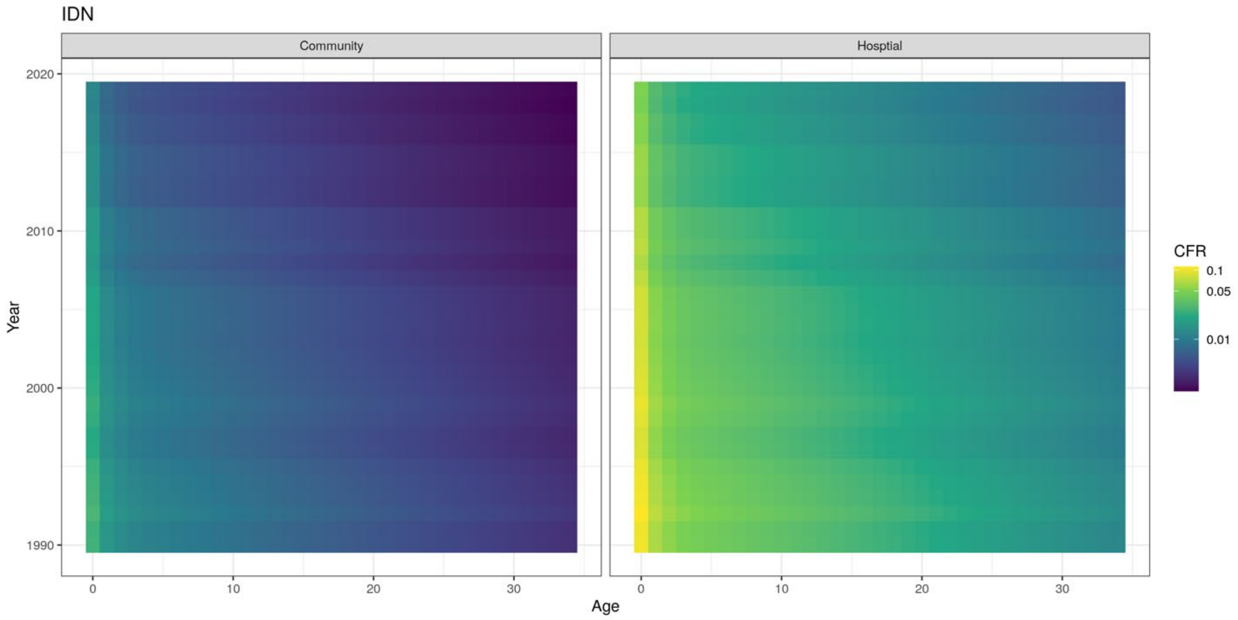
IDN
Community
Hosptial
Year
2020
2010
2000
1990
Age
0
10
20
30
CFR
0.1
0.05
0.01

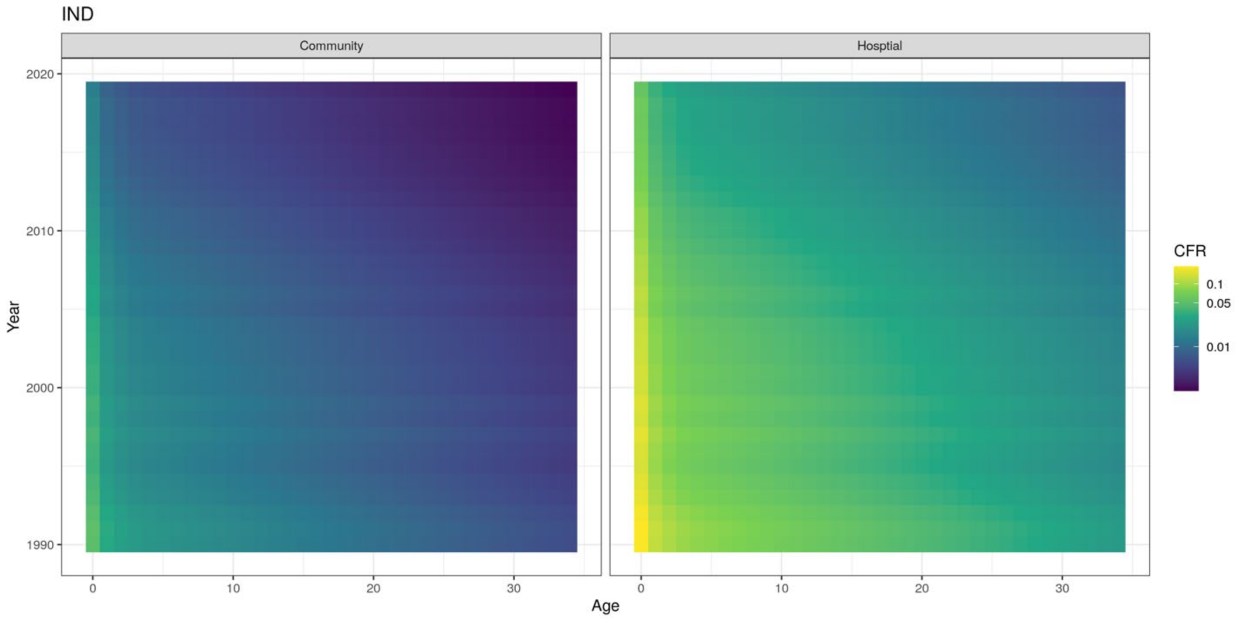
IND
Community
Hosptial
Year
2020
2010
2000
1990
Age
0
10
20
30
CFR
0.1
0.05
0.01

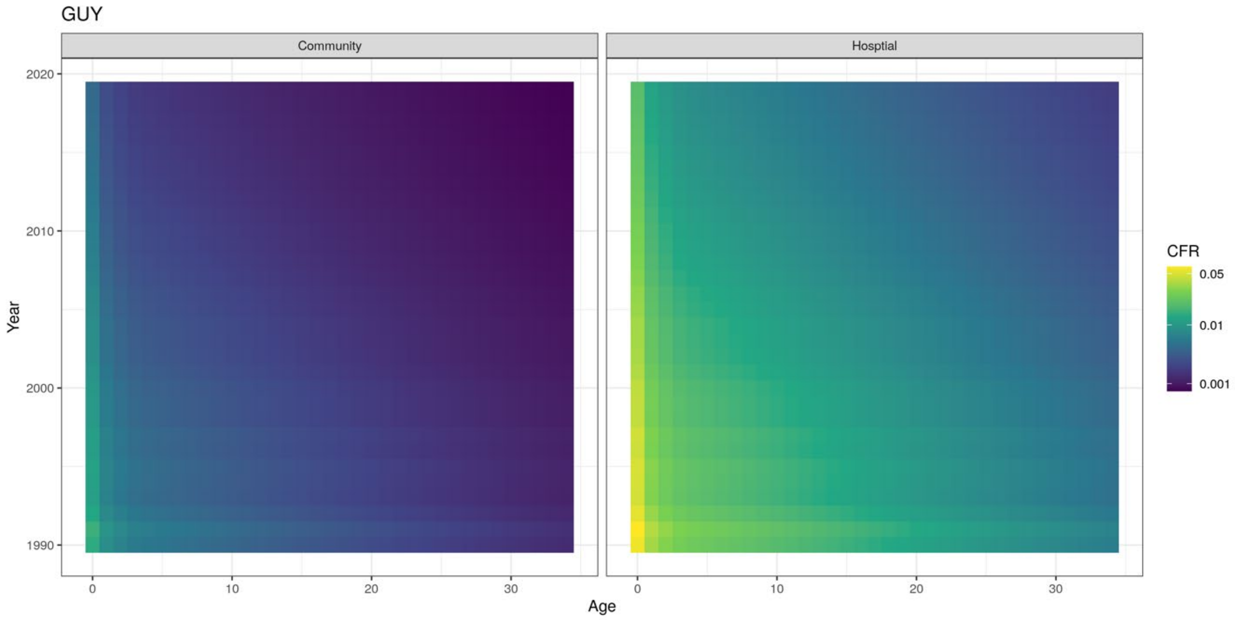
GUY
Community
Hosptial
2020
2010
2000
1990
Year
0
10
20
30
Age
CFR
0.05
0.01
0.001

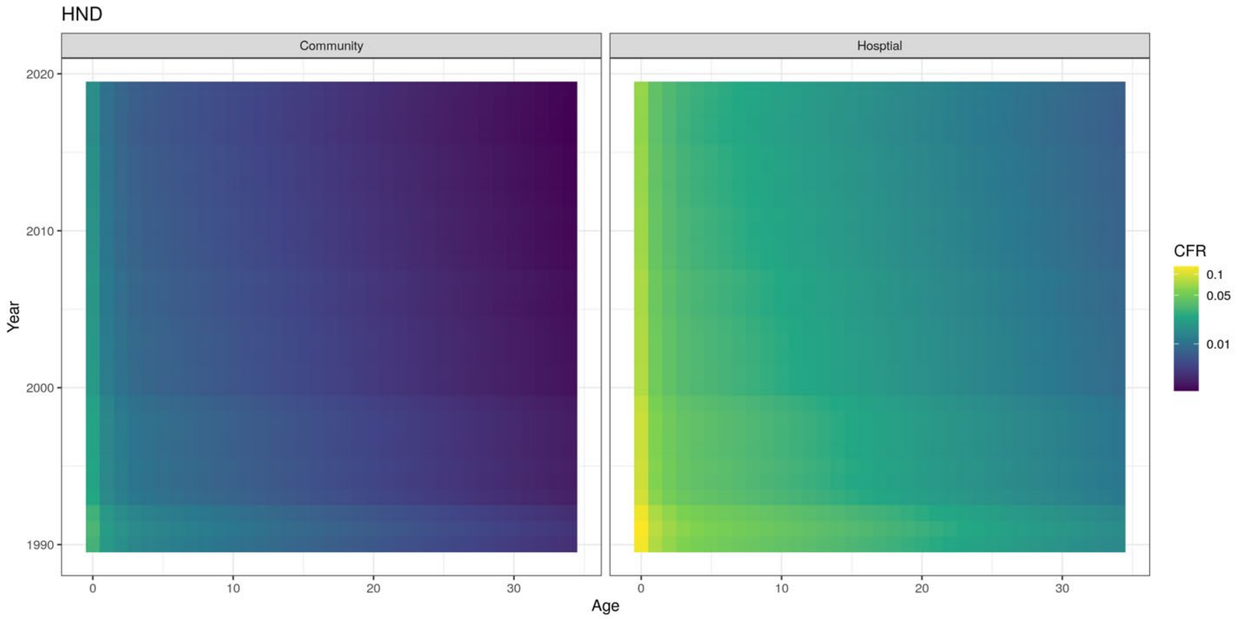
HND
Community
Hosptial
2020
2010
2000
1990
Year
0
10
20
30
Age
CFR
0.1
0.05
0.01

HTI

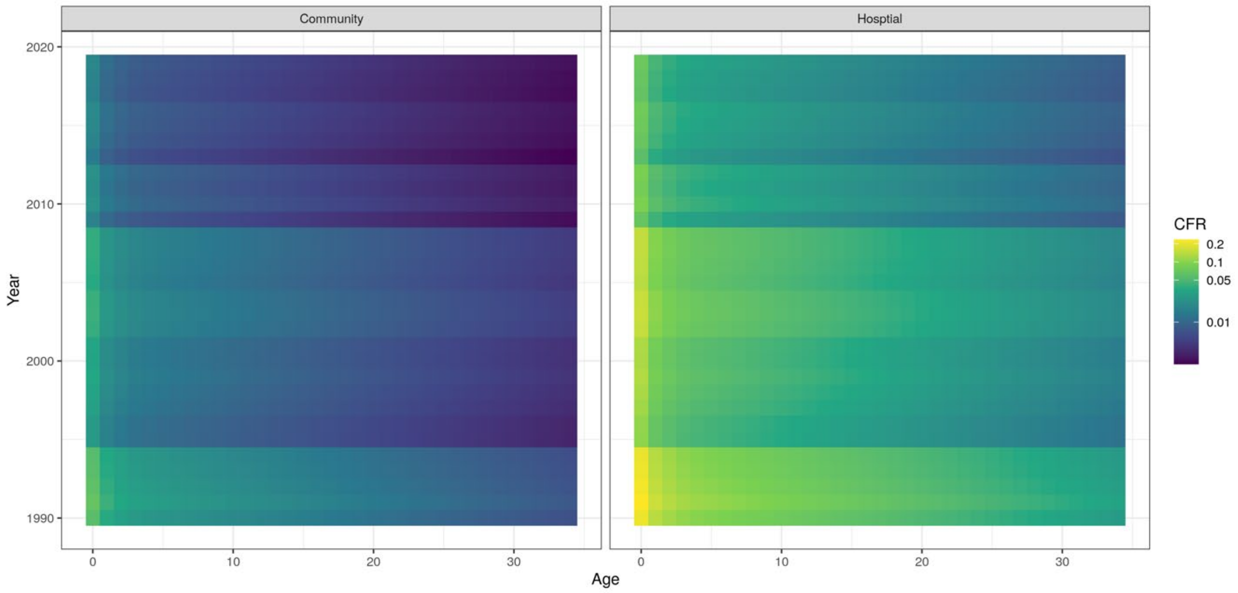
Community
Hosptial
2020
2010
2000
1990
Year
0
10
20
30
Age
CFR
0.2
0.1
0.05
0.01

GNB

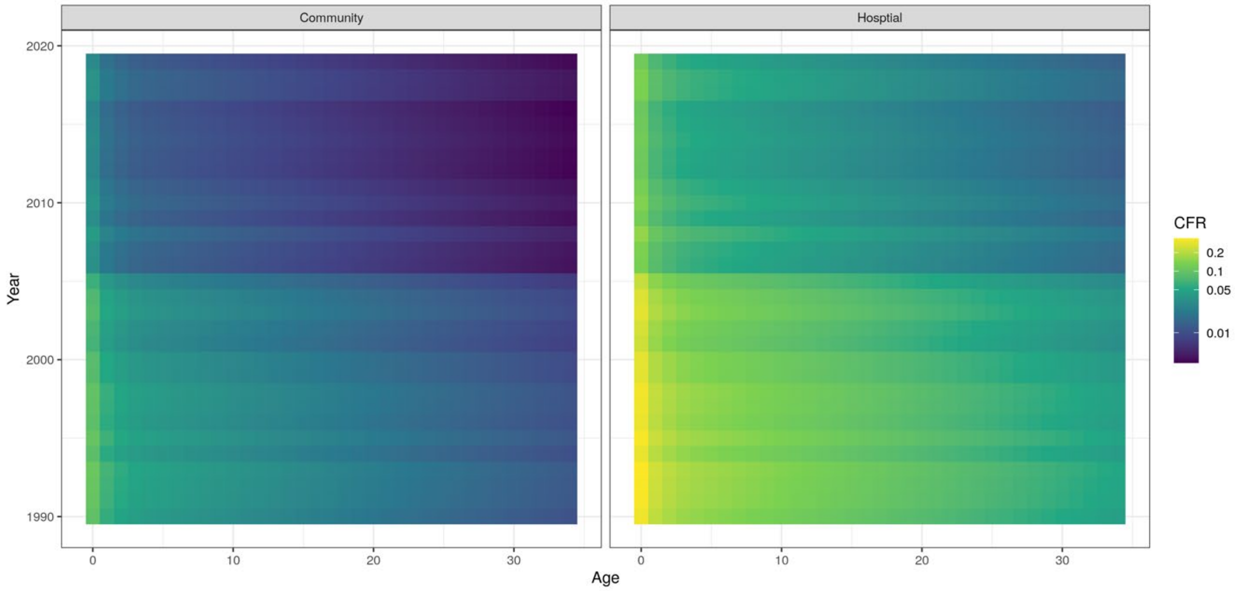
Community
Hosptial
2020
2010
2000
1990
Year
0
10
20
30
Age
CFR
0.2
0.1
0.05
0.01

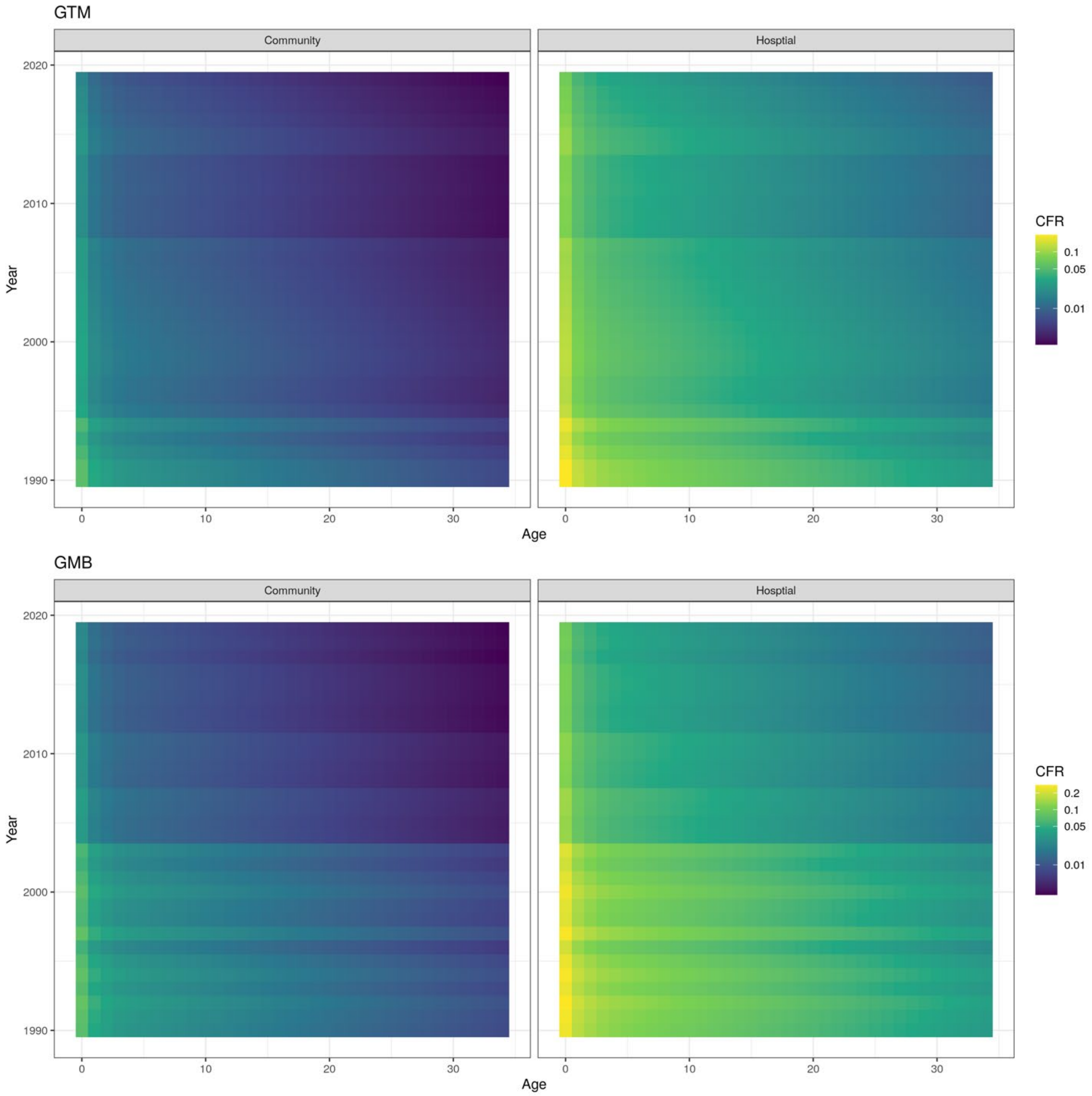
GTM
Community
Hosptial
Year
Age
CFR
0.1
0.05
0.01
GMB
Community
Hosptial
Year
Age
CFR
0.2
0.1
0.05
0.01

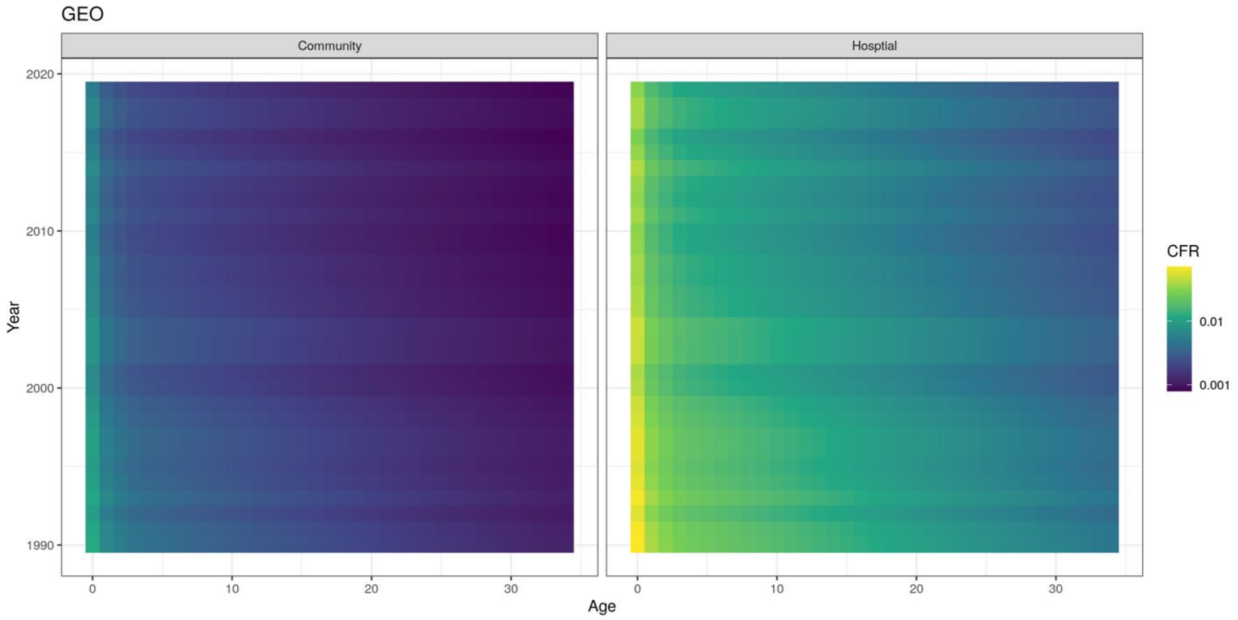
GEO
Community
Hosptial
Year
2020
2010
2000
1990
Age
0
10
20
30
CFR
0.01
0.001

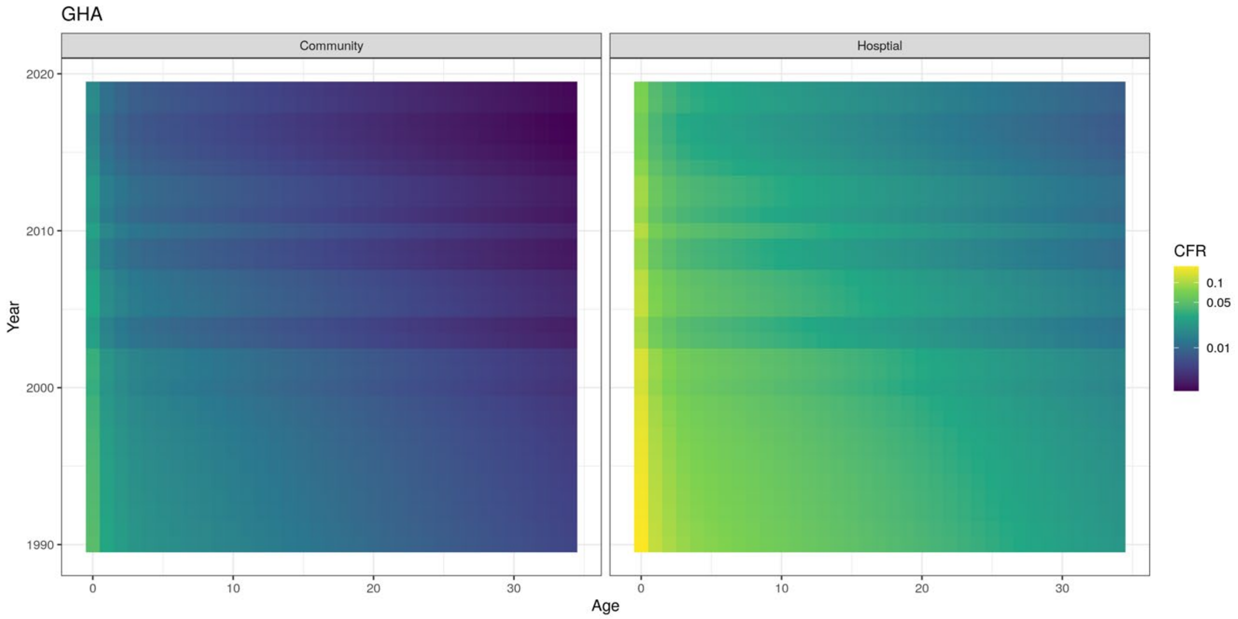
GHA
Community
Hosptial
Year
2020
2010
2000
1990
Age
0
10
20
30
CFR
0.1
0.05
0.01

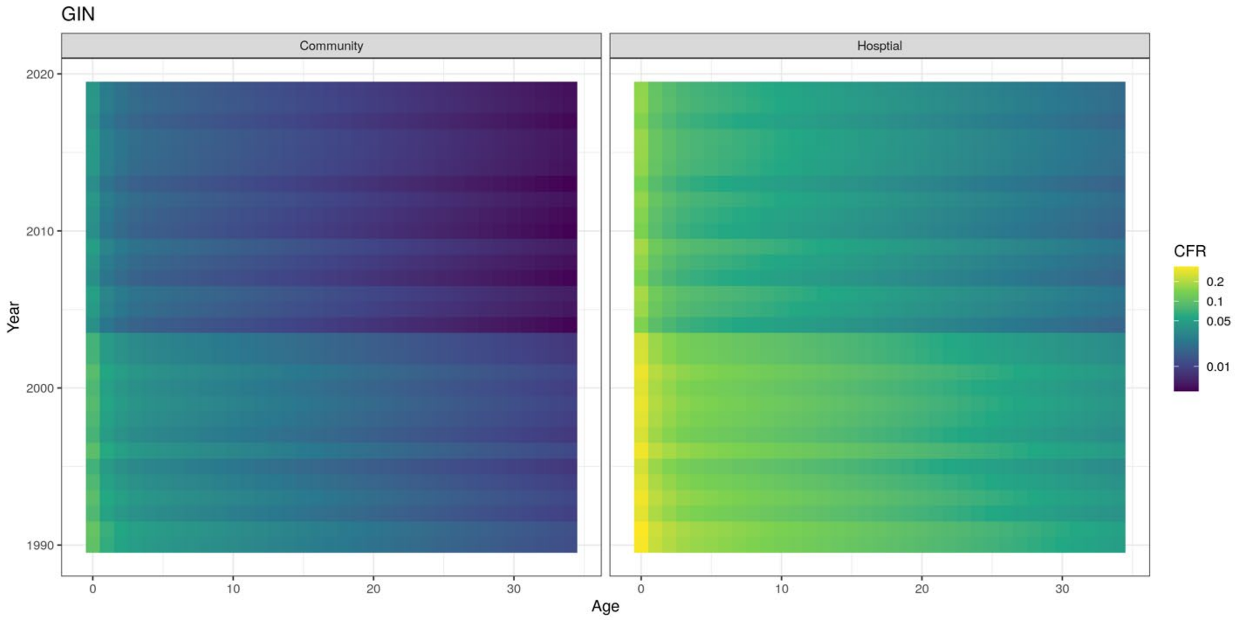
GIN
Community
Hosptial
Year
2020
2010
2000
1990
0
10
20
30
Age
CFR
0.2
0.1
0.05
0.01

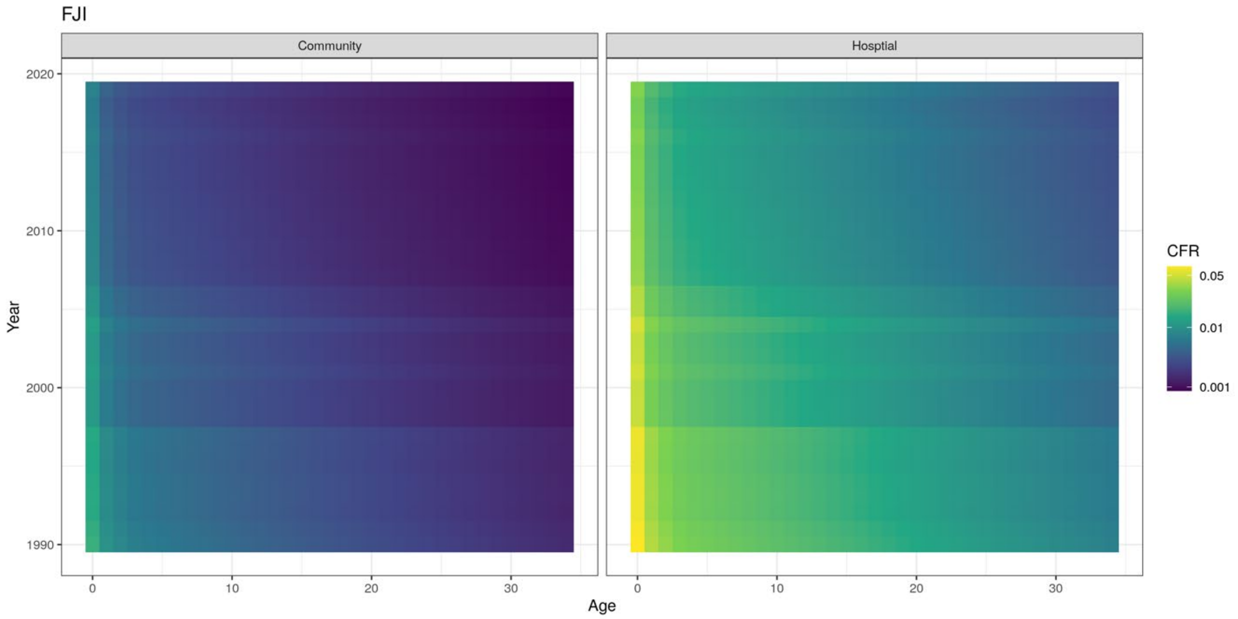
FJI
Community
Hosptial
Year
2020
2010
2000
1990
0
10
20
30
Age
CFR
0.05
0.01
0.001

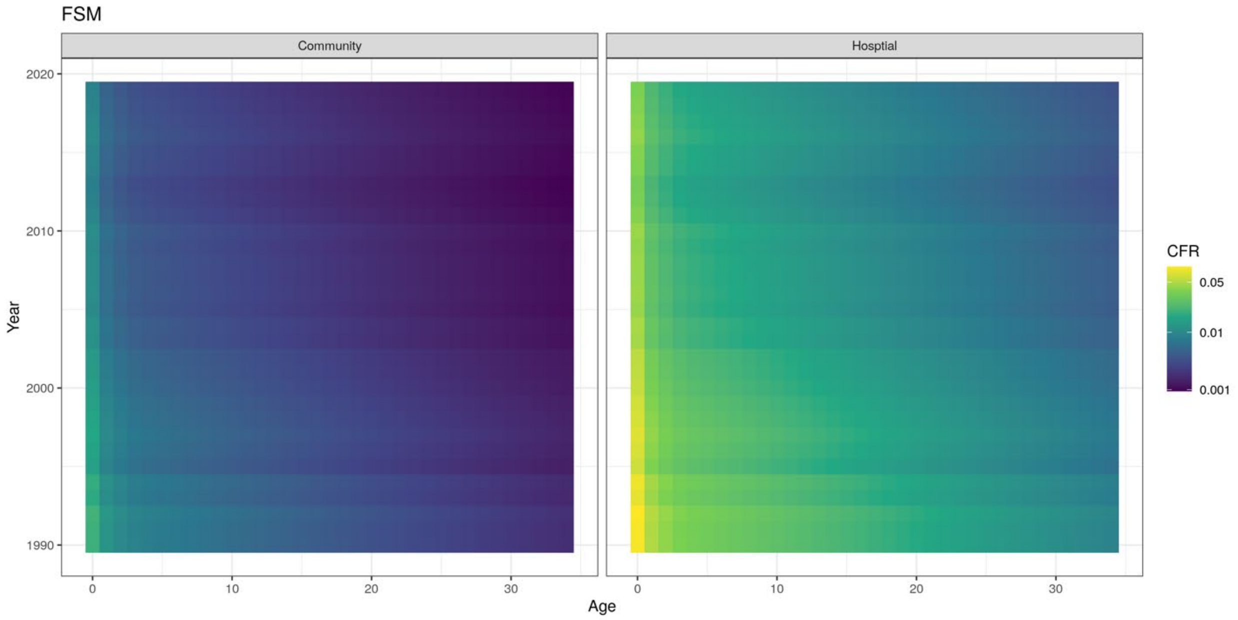
FSM
Community
Hosptial
Year
Age
2020
2010
2000
1990
0
10
20
30
CFR
0.05
0.01
0.001

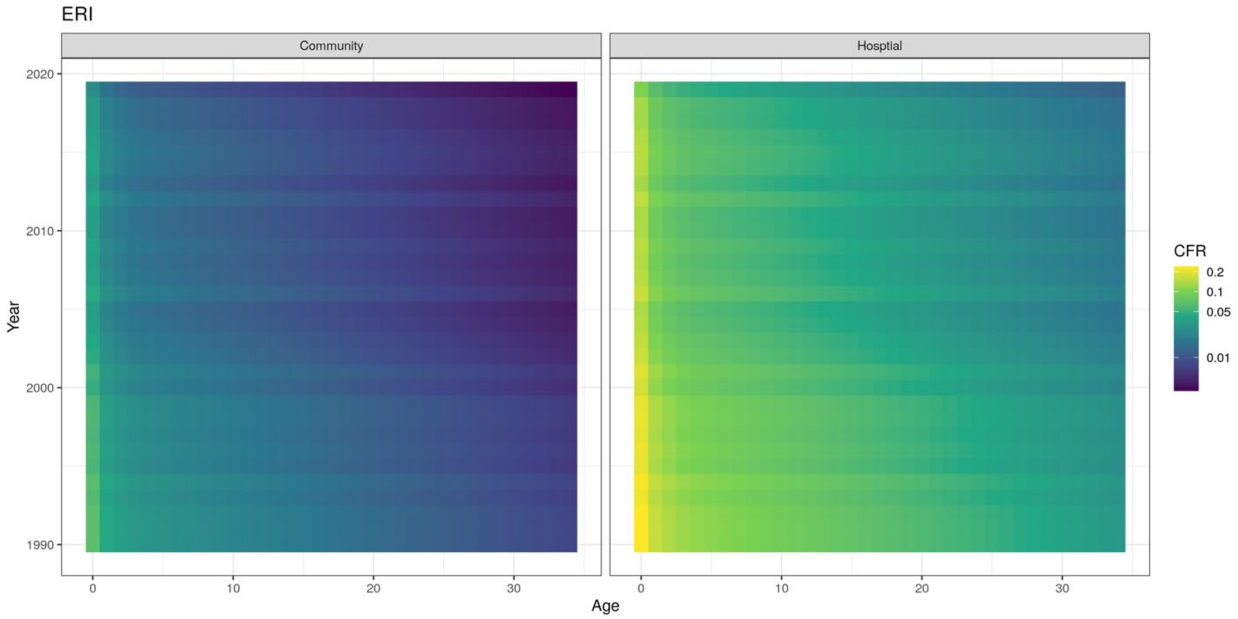
ERI
Community
Hosptial
Year
Age
2020
2010
2000
1990
0
10
20
30
CFR
0.2
0.1
0.05
0.01

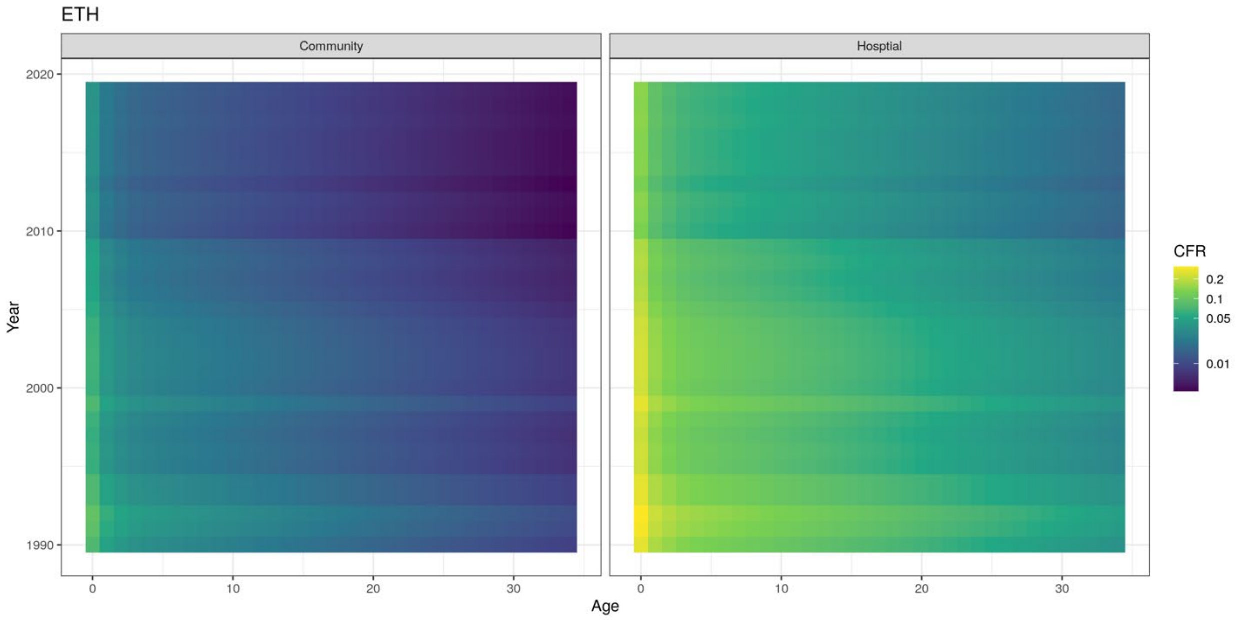
ETH
Community
Hosptial
Year
2020
2010
2000
1990
Age
0
10
20
30
CFR
0.2
0.1
0.05
0.01

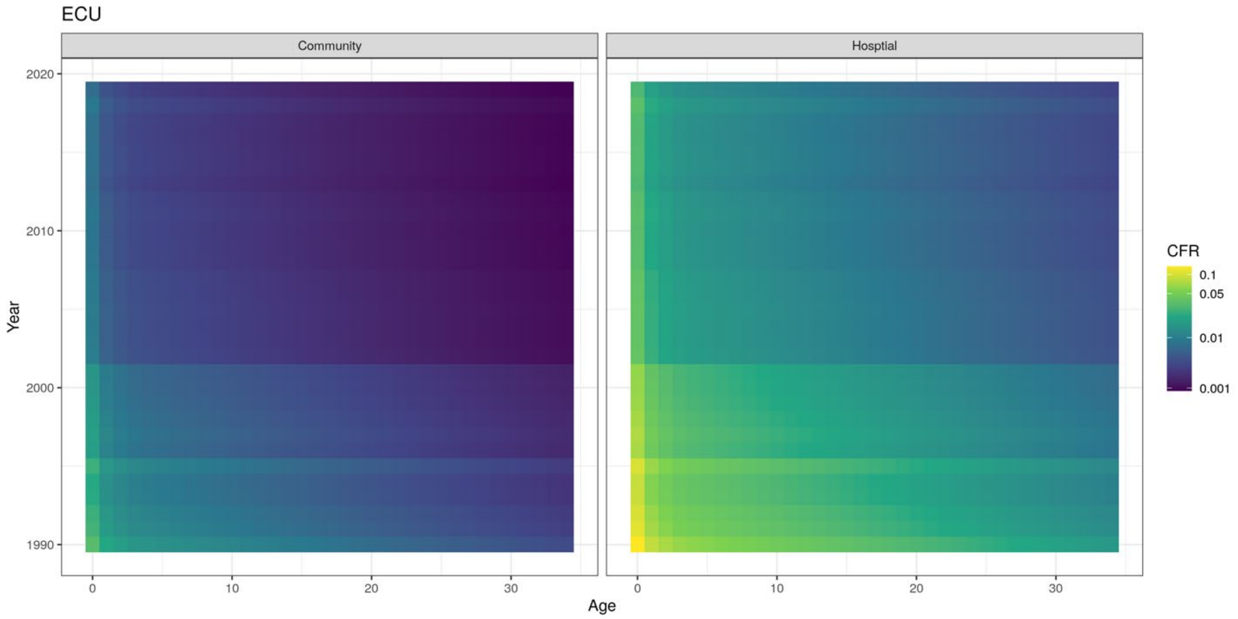
ECU
Community
Hosptial
Year
2020
2010
2000
1990
Age
0
10
20
30
CFR
0.1
0.05
0.01
0.001

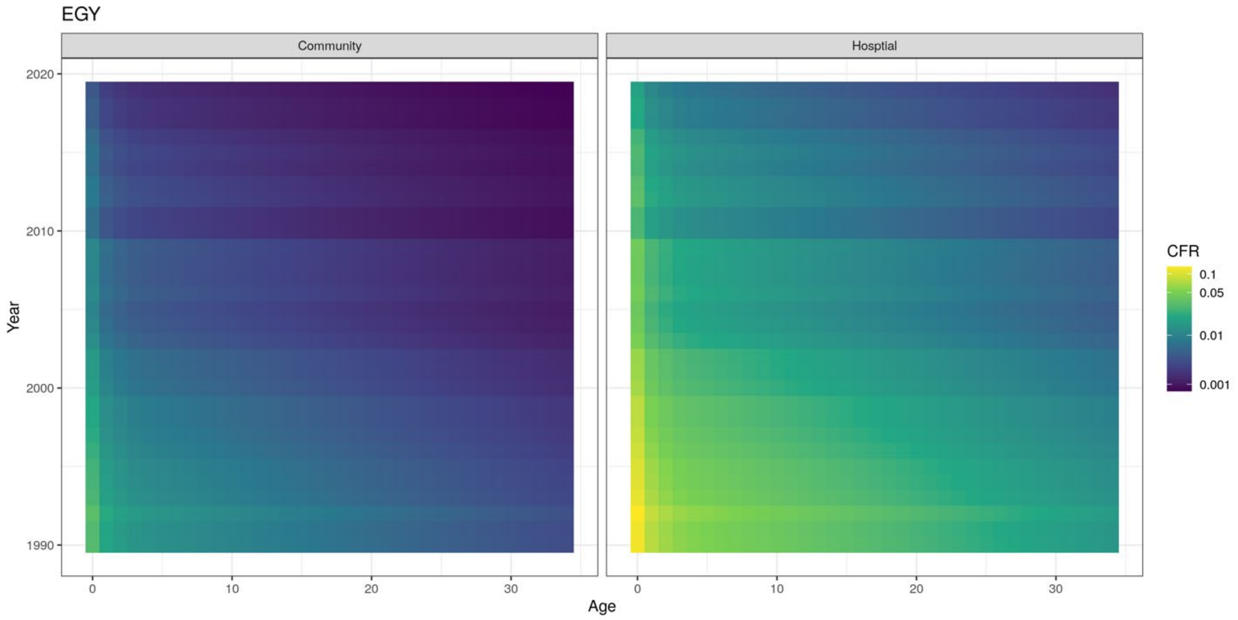
EGY
Community
Hosptial
Year
2020
2010
2000
1990
Age
0
10
20
30
CFR
0.1
0.05
0.01
0.001

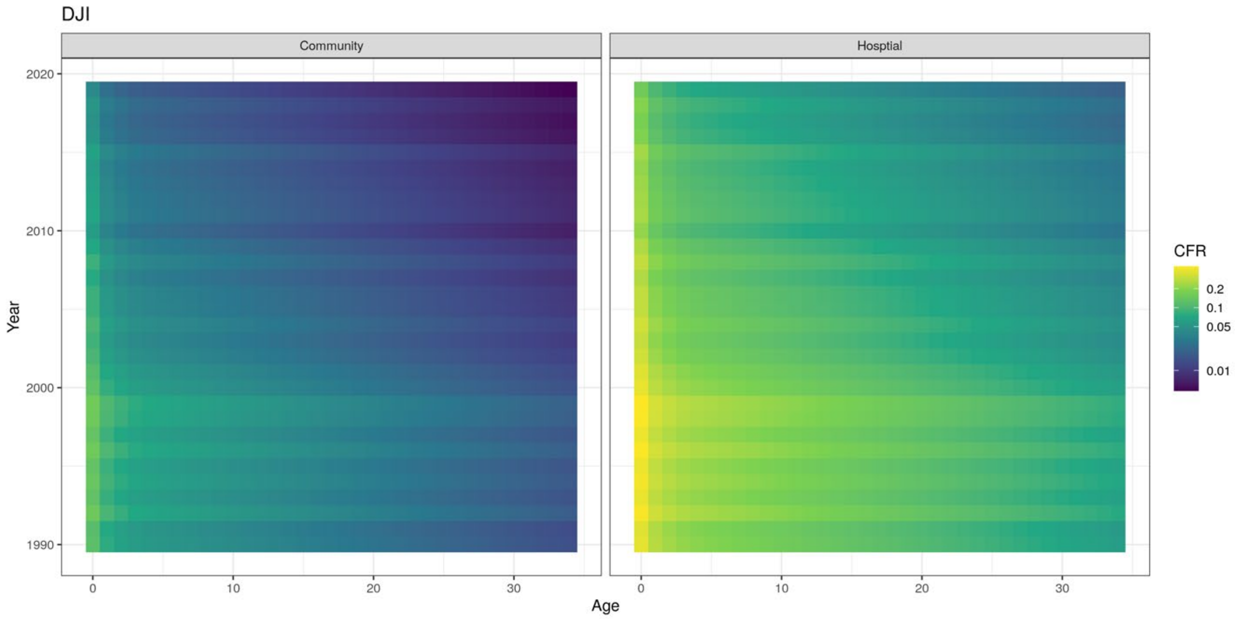
DJI
Community
Hosptial
Year
2020
2010
2000
1990
Age
0
10
20
30
CFR
0.2
0.1
0.05
0.01

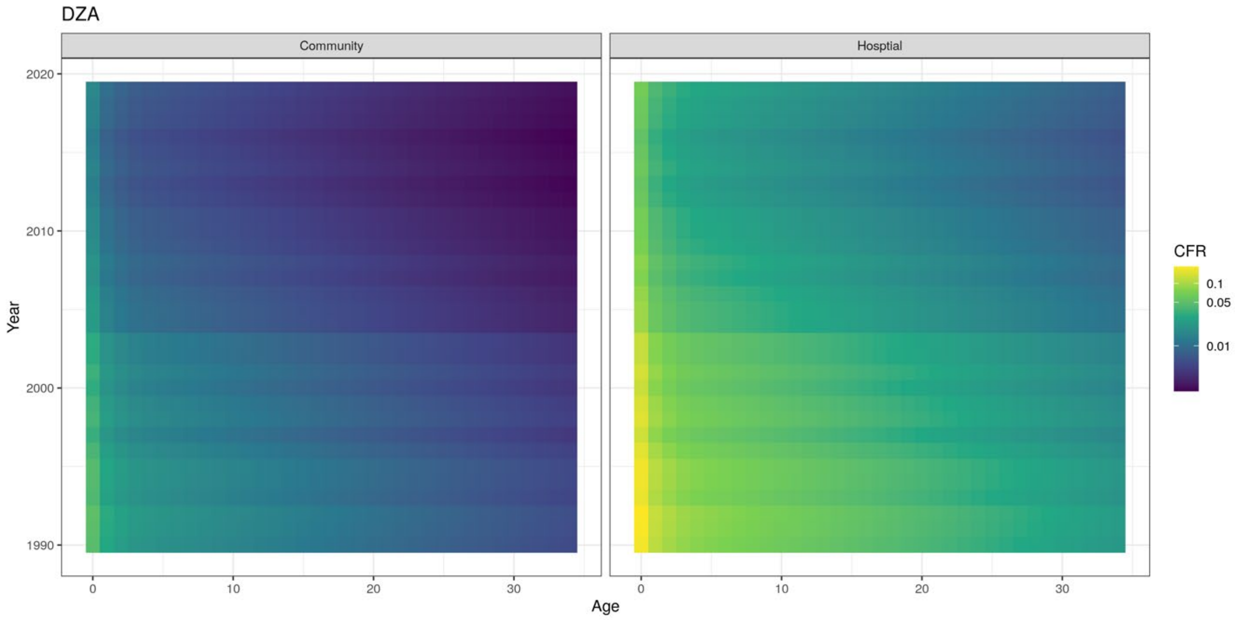
DZA
Community
Hosptial
Year
Age
CFR
0.1
0.05
0.01
2020
2010
2000
1990
0
10
20
30

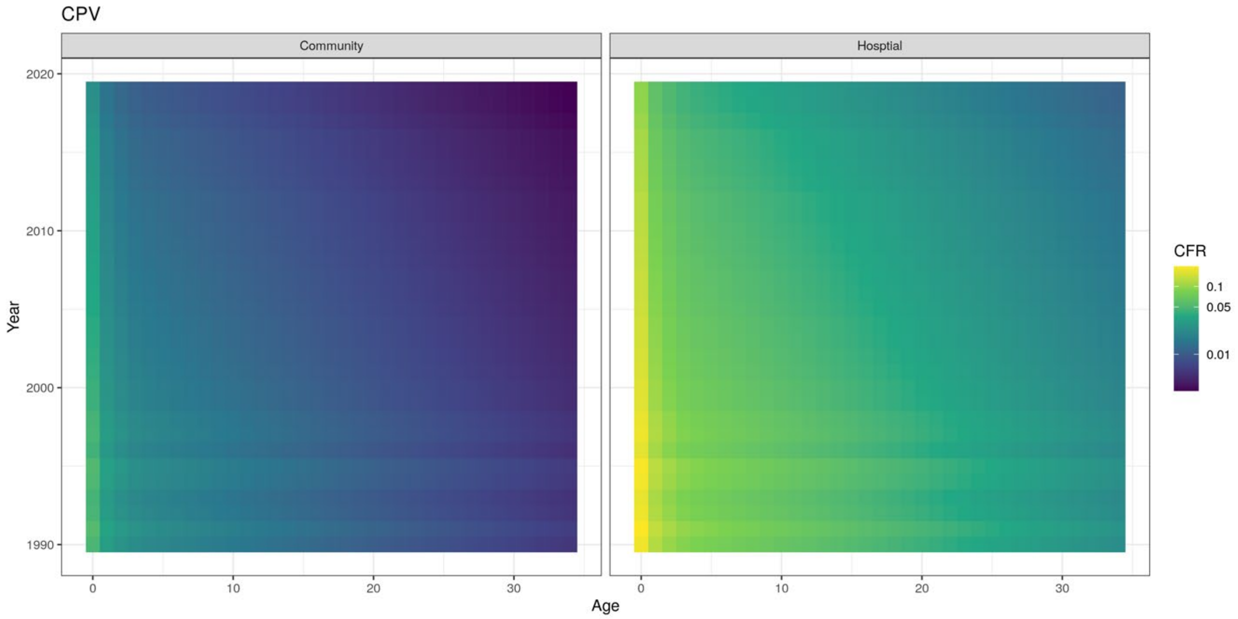
CPV
Community
Hosptial
Year
Age
CFR
0.1
0.05
0.01
2020
2010
2000
1990
0
10
20
30

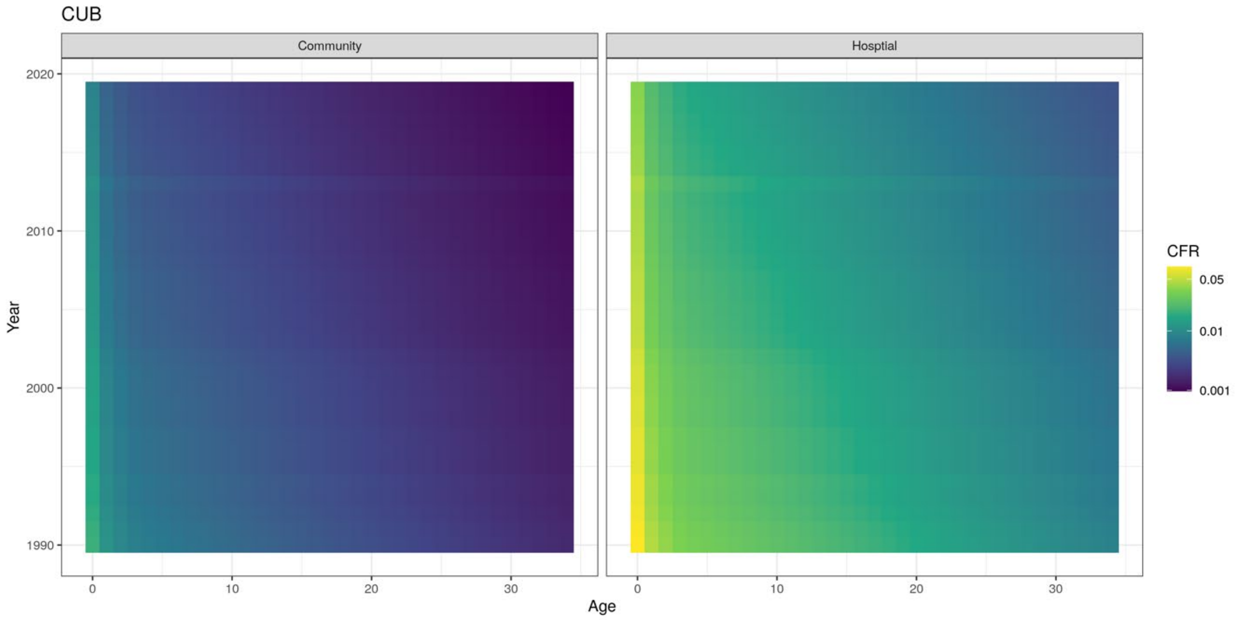

CUB
Community
Hosptial
Year
2020
2010
2000
1990
Age
0
10
20
30
CFR
0.05
0.01
0.001

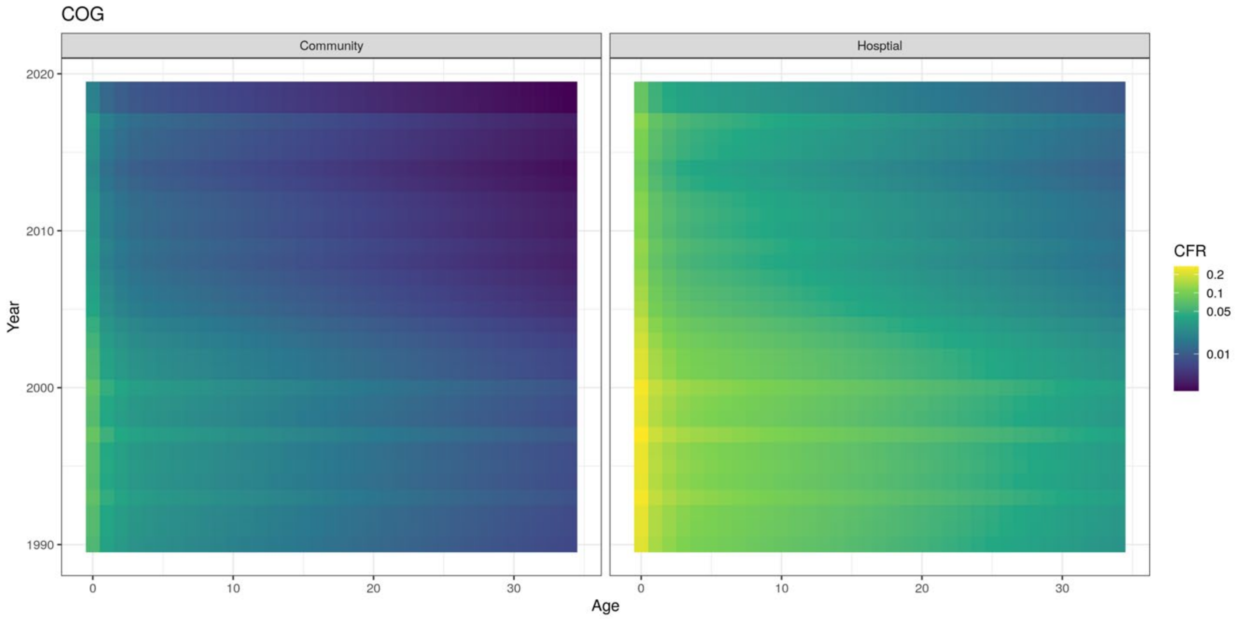

COG
Community
Hosptial
Year
2020
2010
2000
1990
Age
0
10
20
30
CFR
0.2
0.1
0.05
0.01

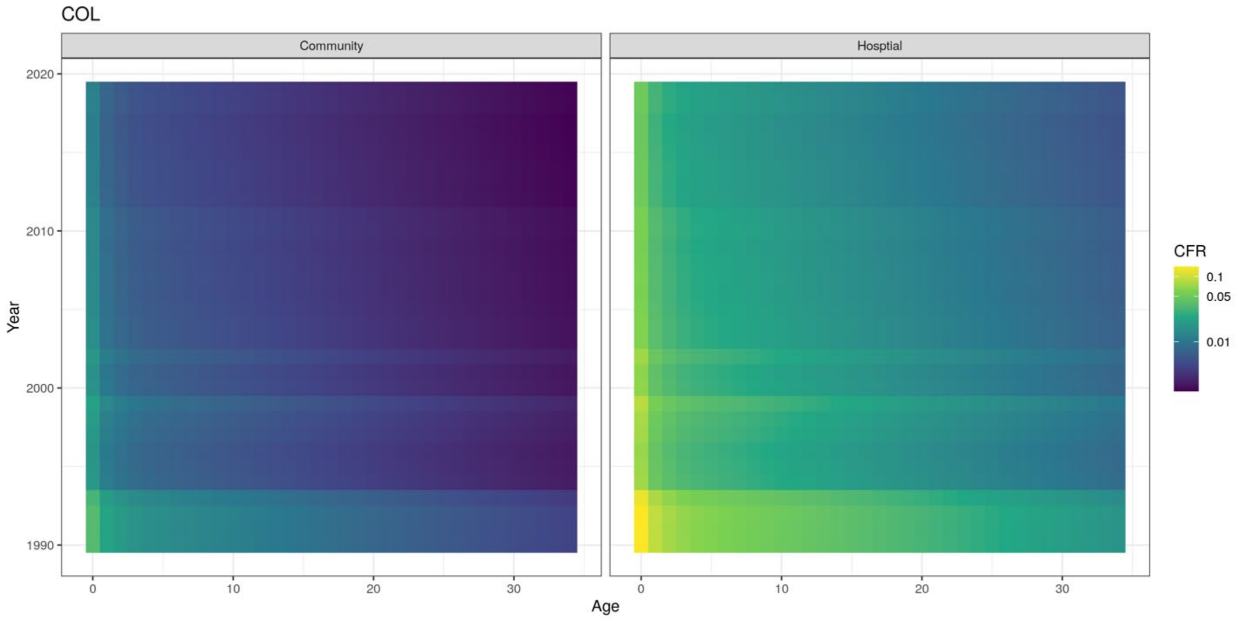
COL
Community
Hosptial
Year
Age
CFR
0.1
0.05
0.01

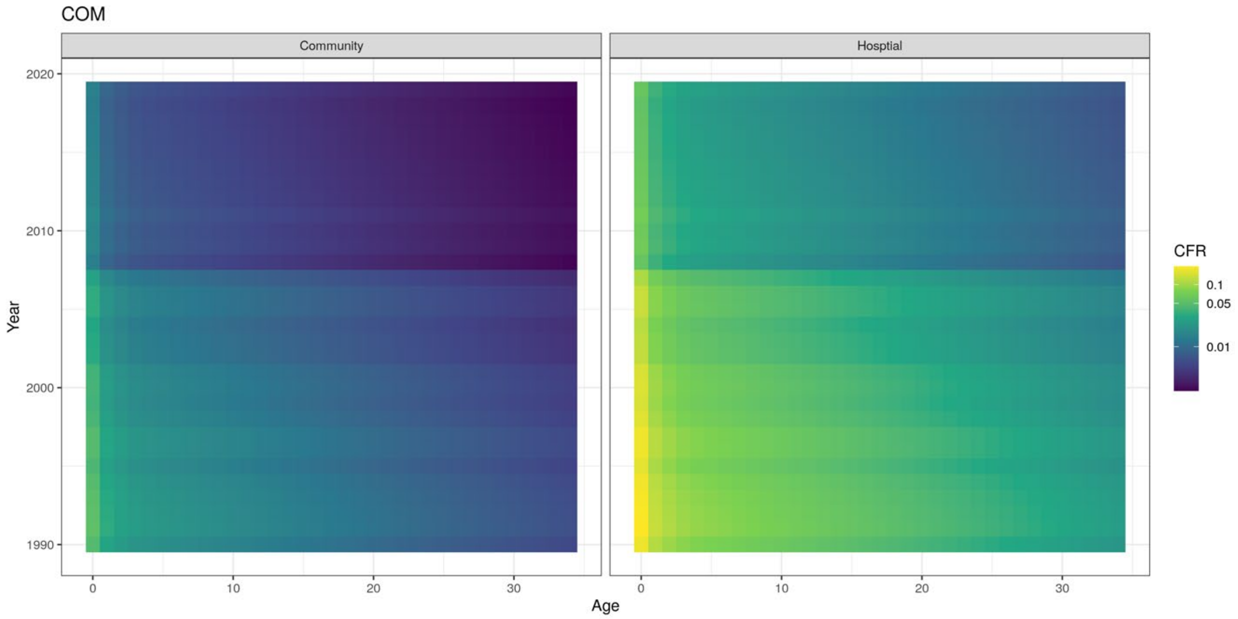
COM
Community
Hosptial
Year
Age
CFR
0.1
0.05
0.01

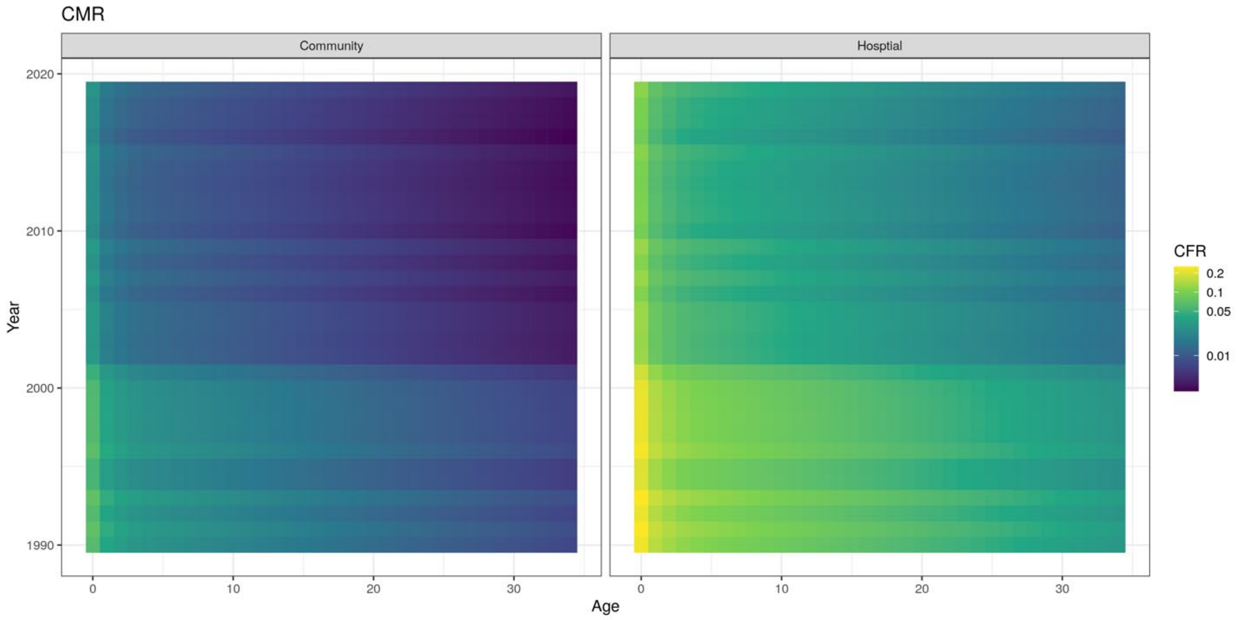
CMR
Community
Hosptial
2020
2010
2000
1990
Year
0
10
20
30
Age
CFR
0.2
0.1
0.05
0.01

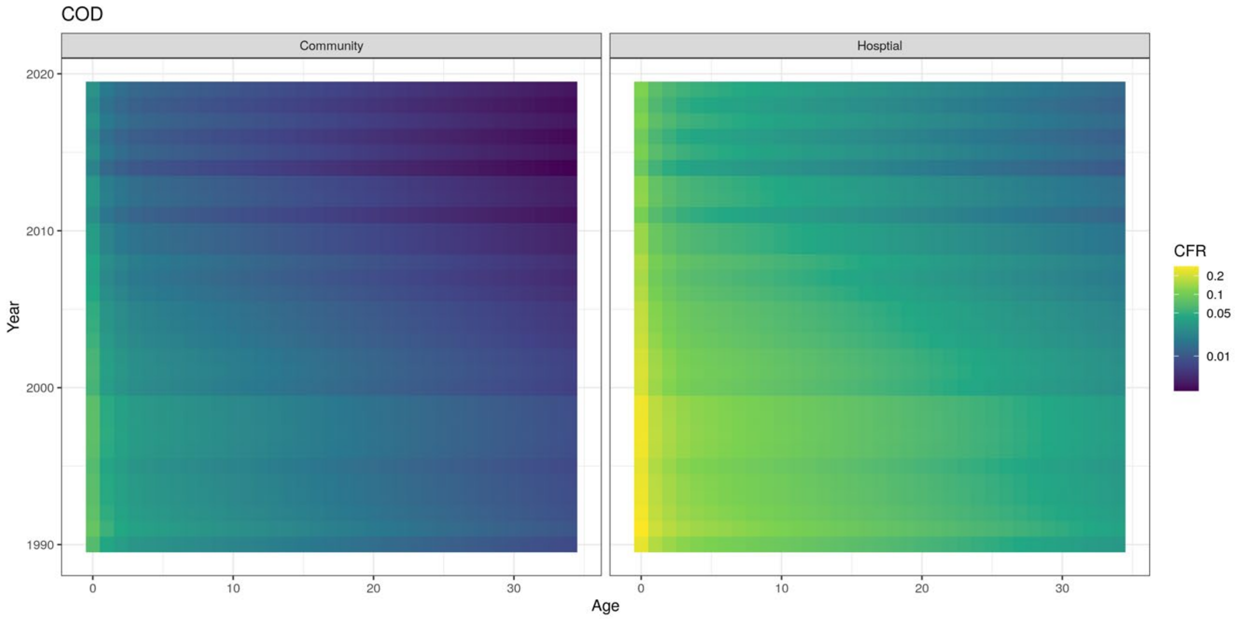
COD
Community
Hosptial
2020
2010
2000
1990
Year
0
10
20
30
Age
CFR
0.2
0.1
0.05
0.01

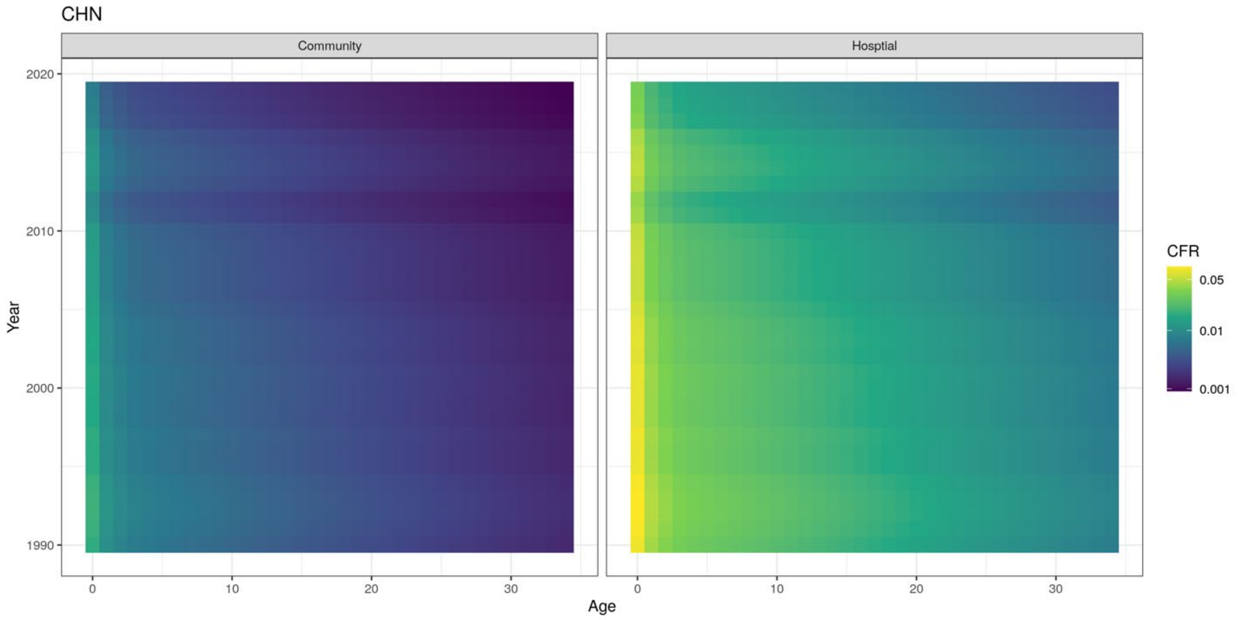
CHN
Community
Hosptial
Year
Age
CFR
0.05
0.01
0.001

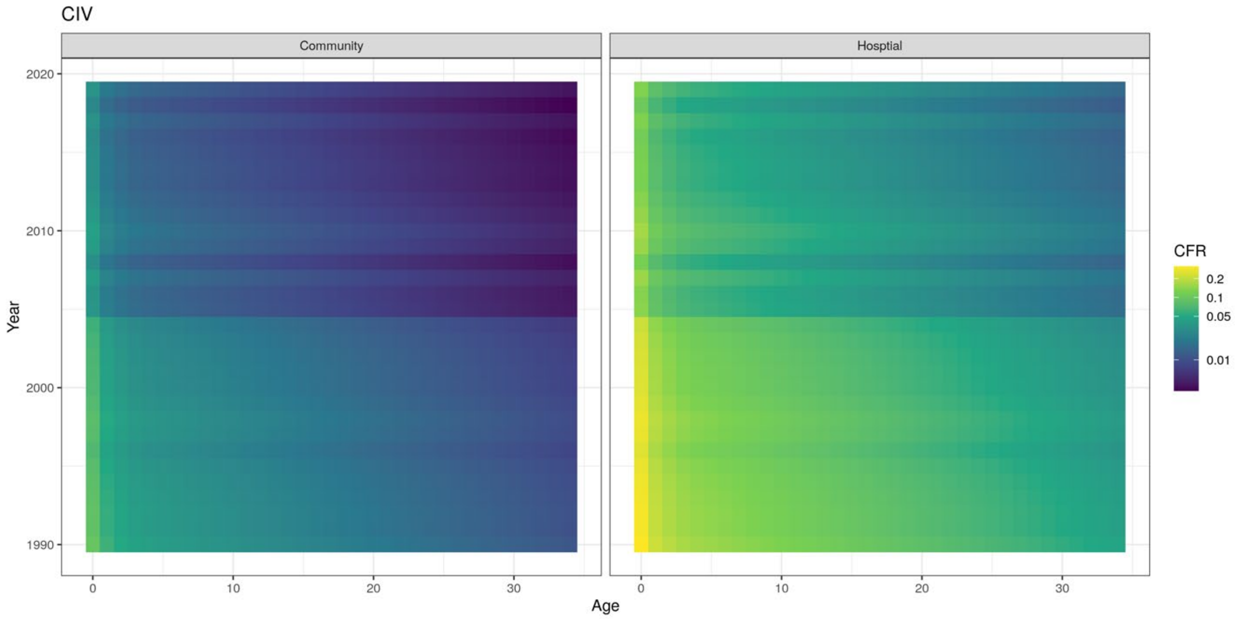
CIV
Community
Hosptial
Year
Age
CFR
0.2
0.1
0.05
0.01

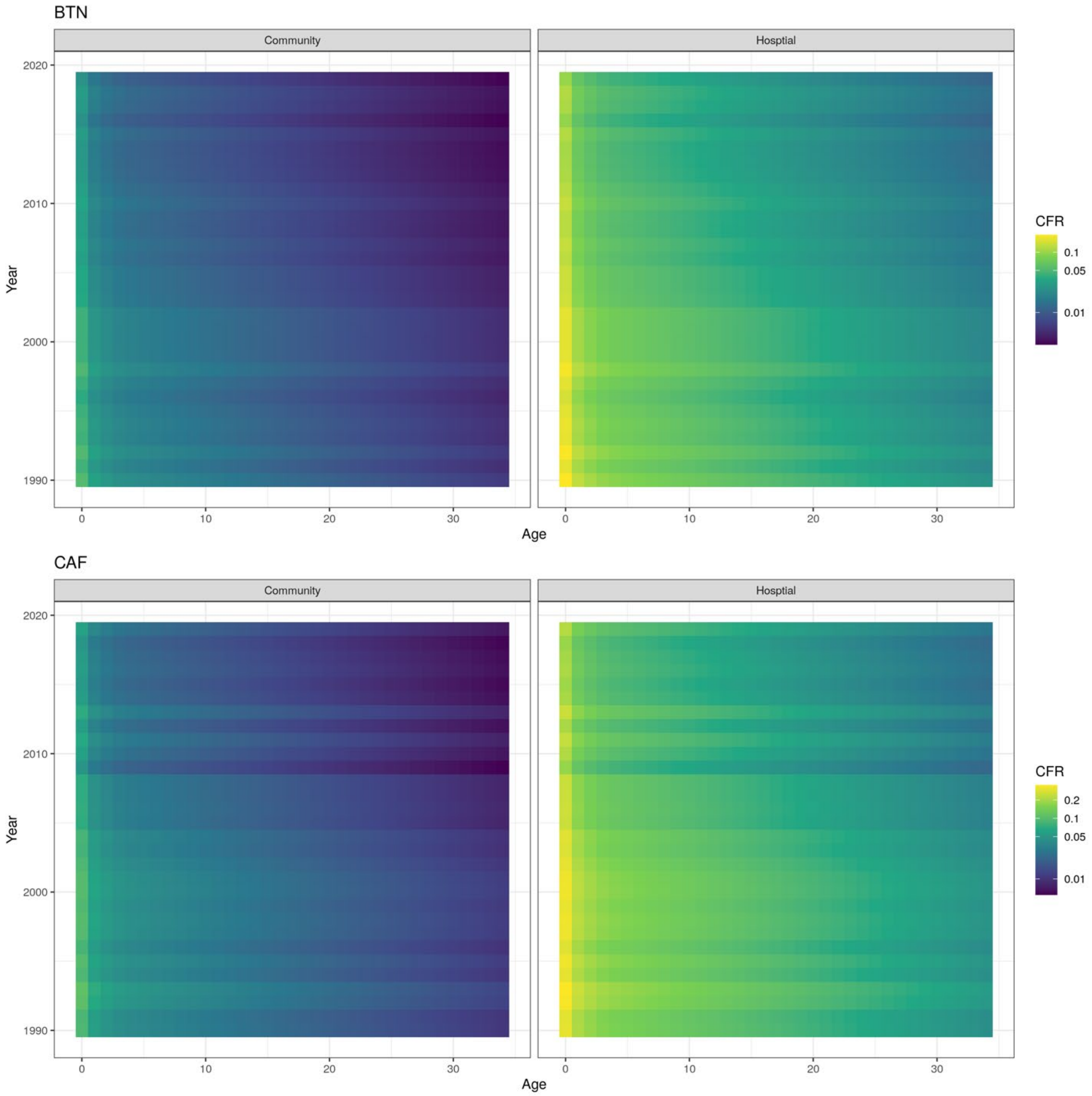
BTN
Community
Hosptial
CFR
0.1
0.05
0.01
Year
Age
CAF
Community
Hosptial
CFR
0.2
0.1
0.05
0.01
Year
Age

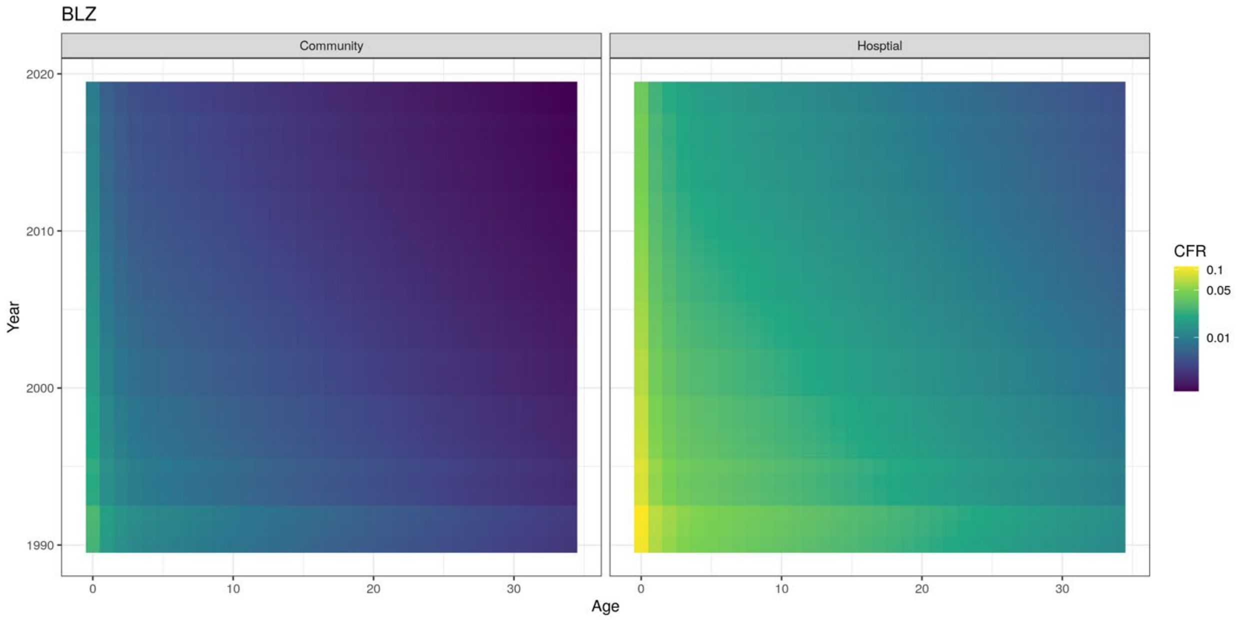
BLZ
Community
Hosptial
Year
2020
2010
2000
1990
Age
0
10
20
30
CFR
0.1
0.05
0.01

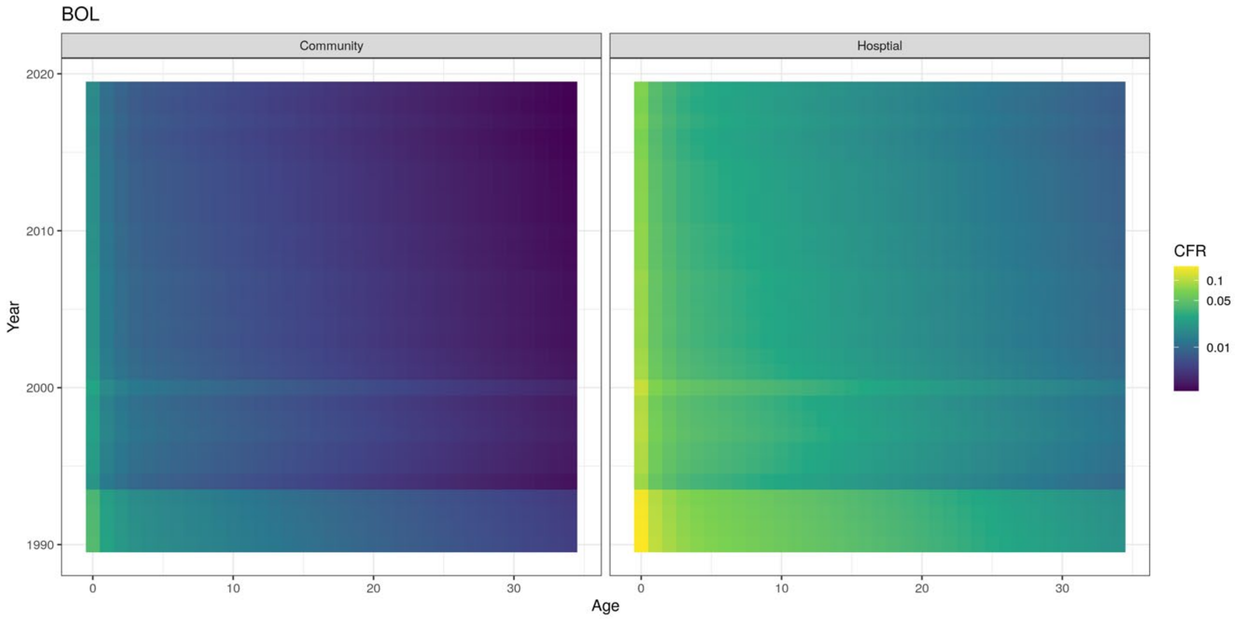
BOL
Community
Hosptial
Year
2020
2010
2000
1990
Age
0
10
20
30
CFR
0.1
0.05
0.01

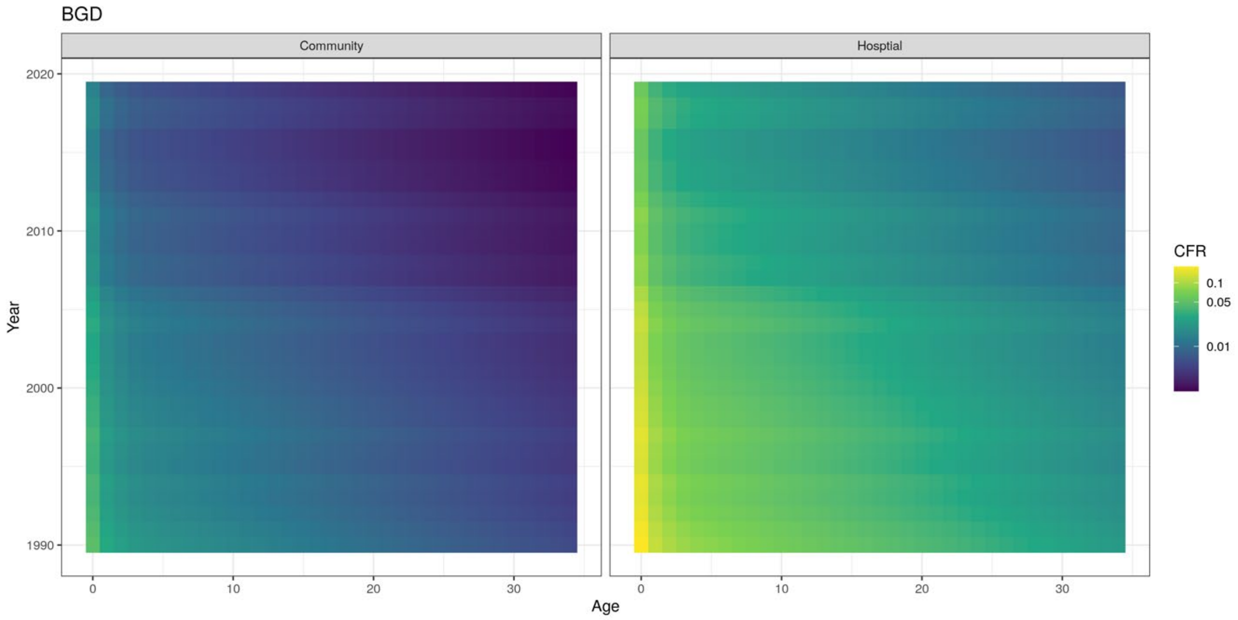
BGD
Community
Hosptial
Year
Age
CFR
0.1
0.05
0.01
2020
2010
2000
1990
0
10
20
30

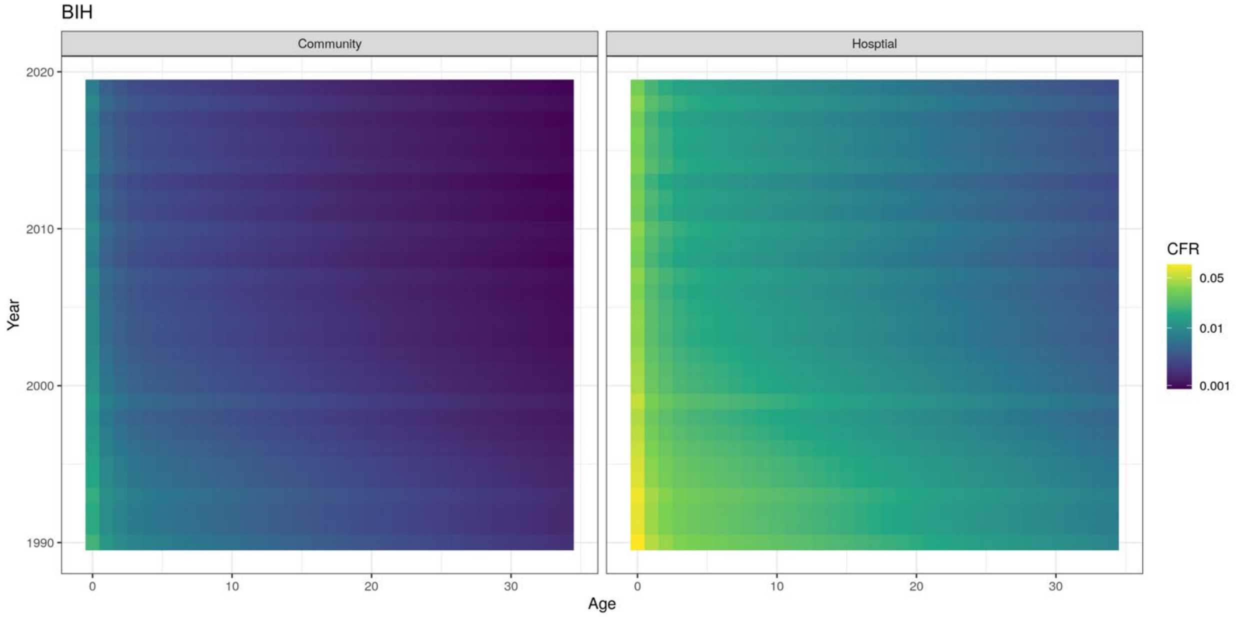
BIH
Community
Hosptial
Year
Age
CFR
0.05
0.01
0.001
2020
2010
2000
1990
0
10
20
30

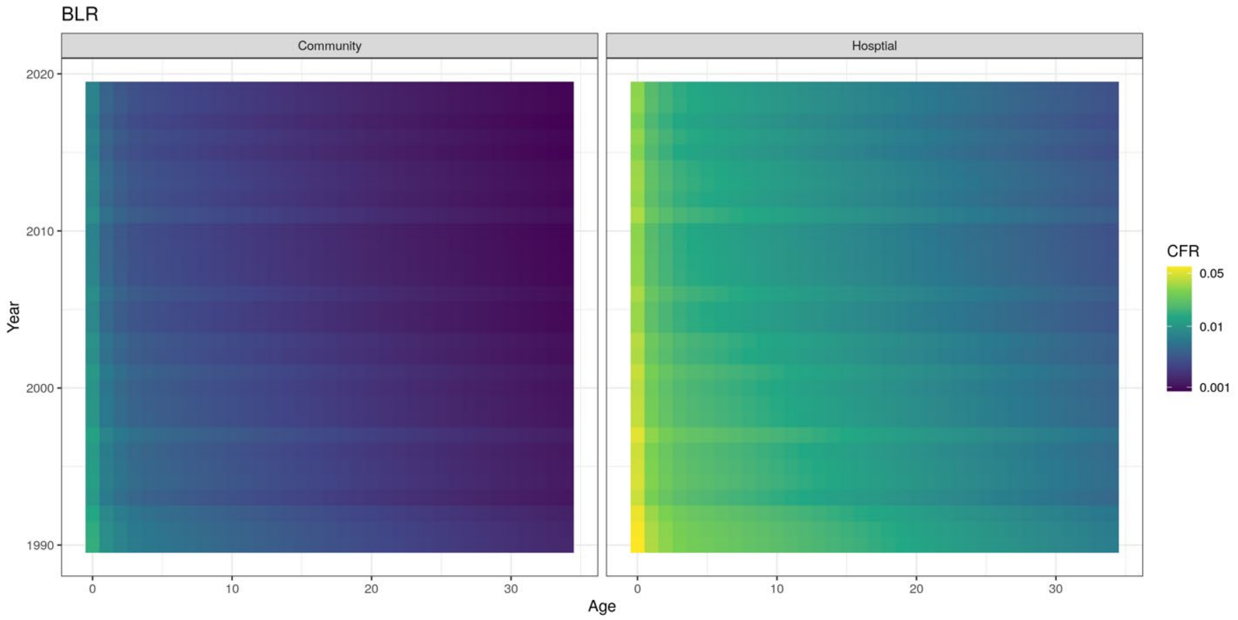
BLR
Community
Hosptial
2020
2010
2000
1990
Year
0
10
20
30
Age
CFR
0.05
0.01
0.001

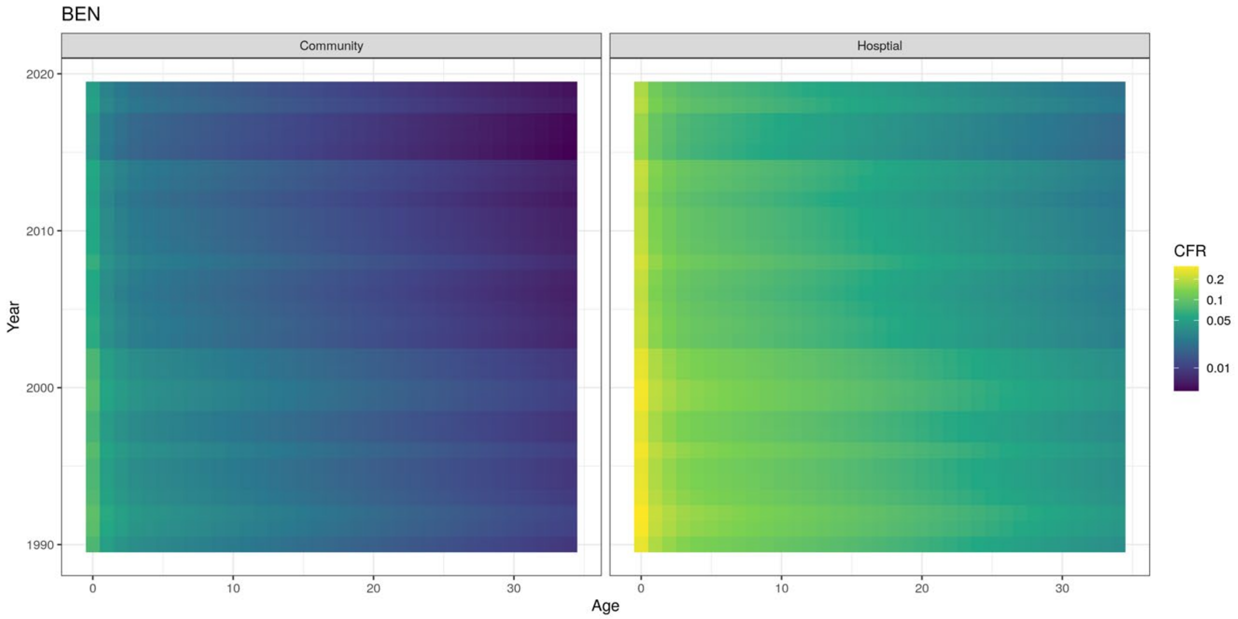
BEN
Community
Hosptial
2020
2010
2000
1990
Year
0
10
20
30
Age
CFR
0.2
0.1
0.05
0.01

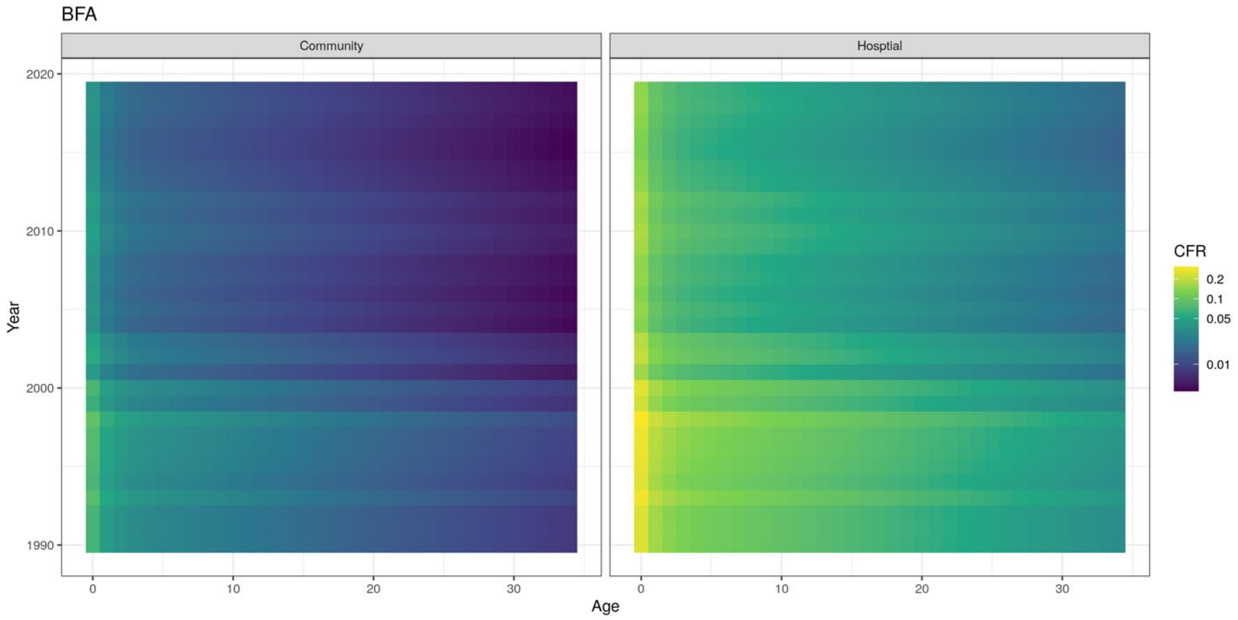
BFA
Community
Hosptial
2020
2010
2000
1990
Year
0
10
20
30
Age
CFR
0.2
0.1
0.05
0.01

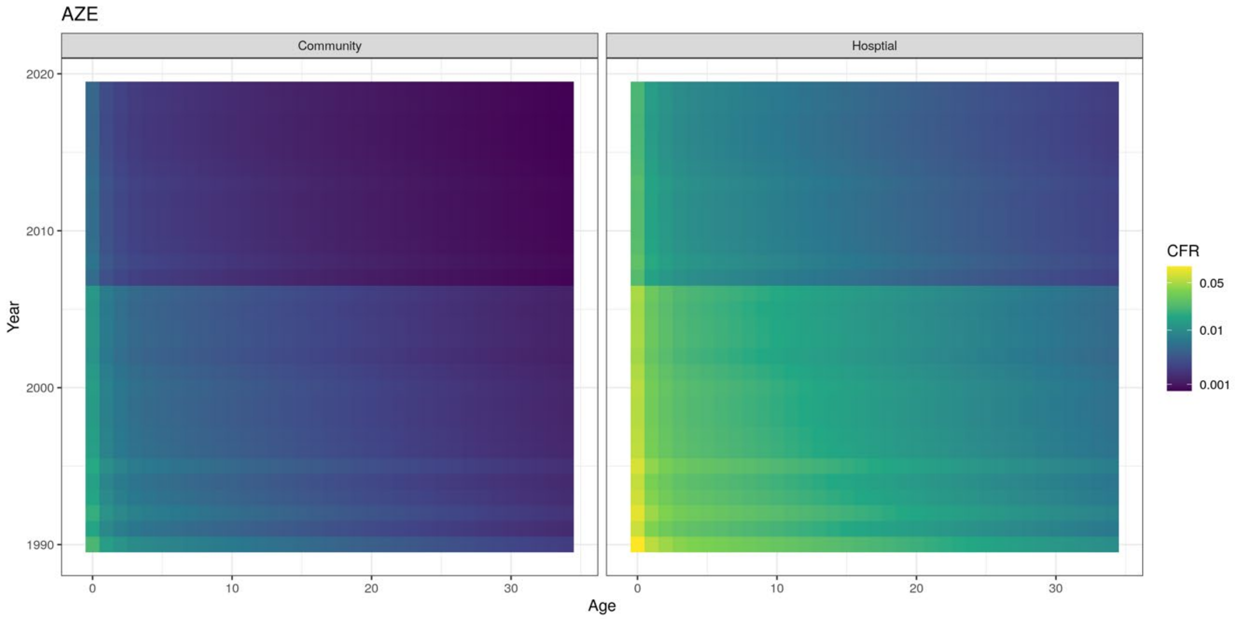
AZE
Community
Hosptial
2020
2010
2000
1990
Year
0
10
20
30
Age
CFR
0.05
0.01
0.001

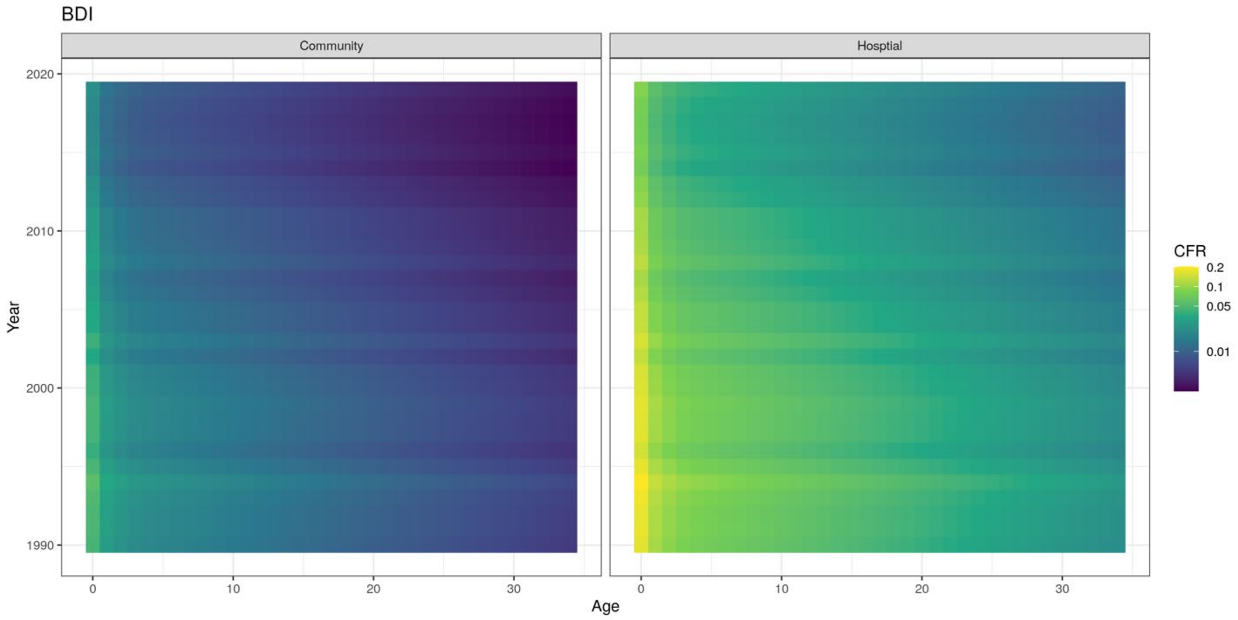
BDI
Community
Hosptial
Year
2020
2010
2000
1990
Age
0
10
20
30
CFR
0.2
0.1
0.05
0.01

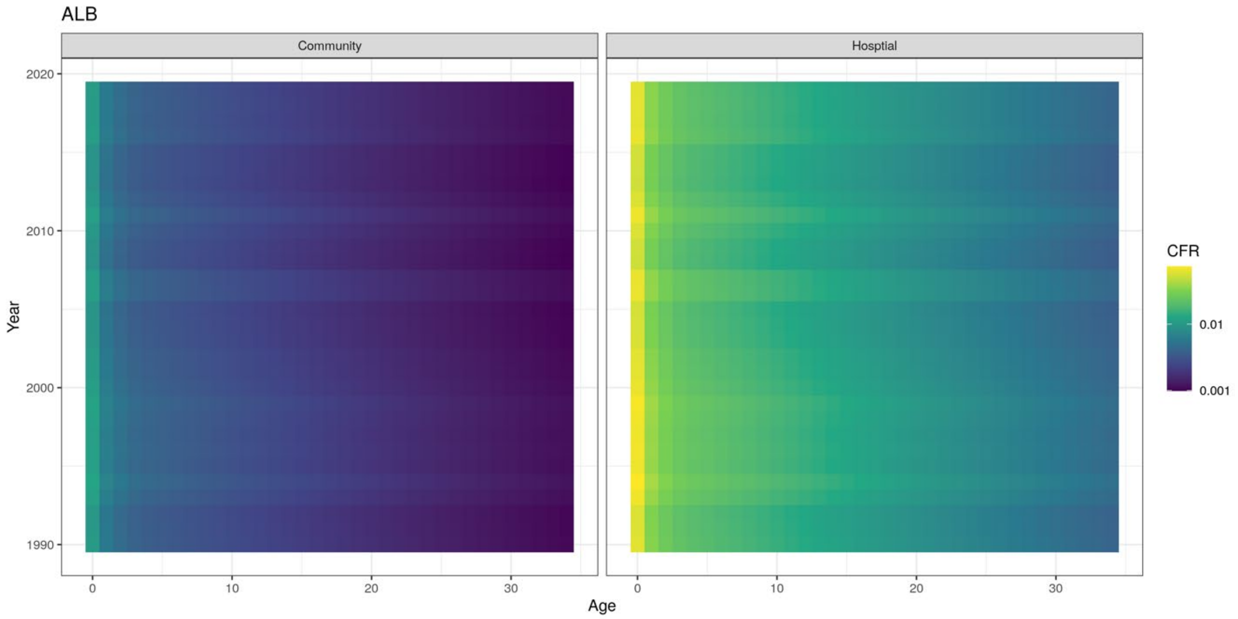
ALB
Community
Hosptial
Year
2020
2010
2000
1990
Age
0
10
20
30
CFR
0.01
0.001

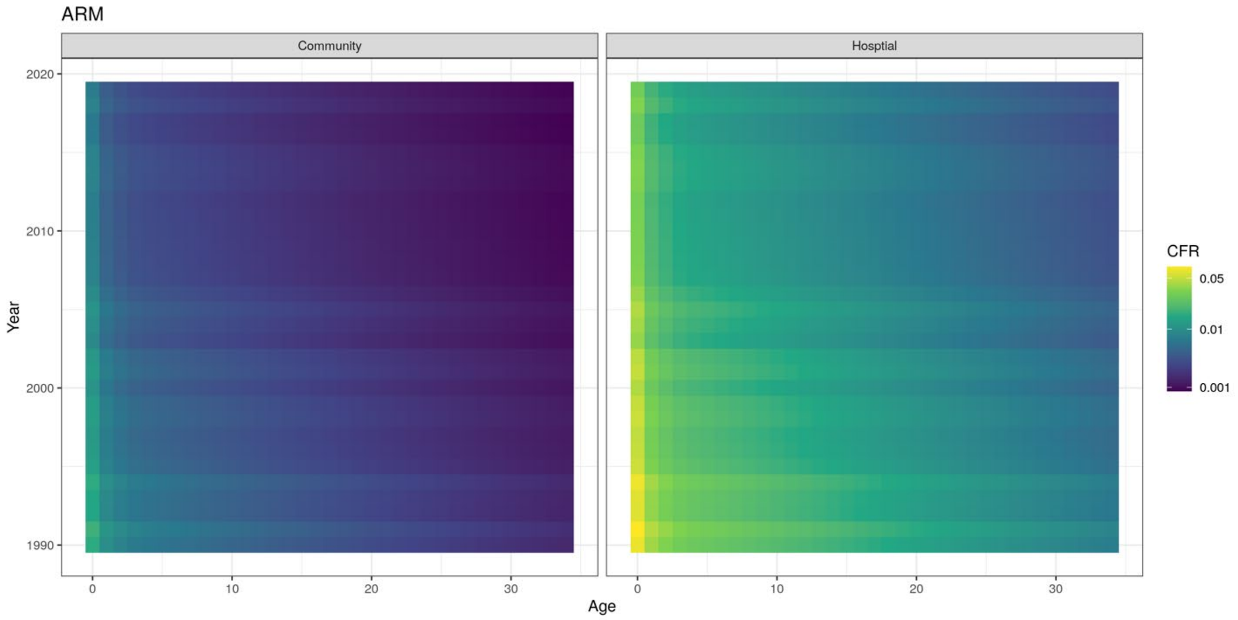
ARM
Community
Hosptial
2020
2010
2000
1990
Year
0
10
20
30
Age
CFR
0.05
0.01
0.001

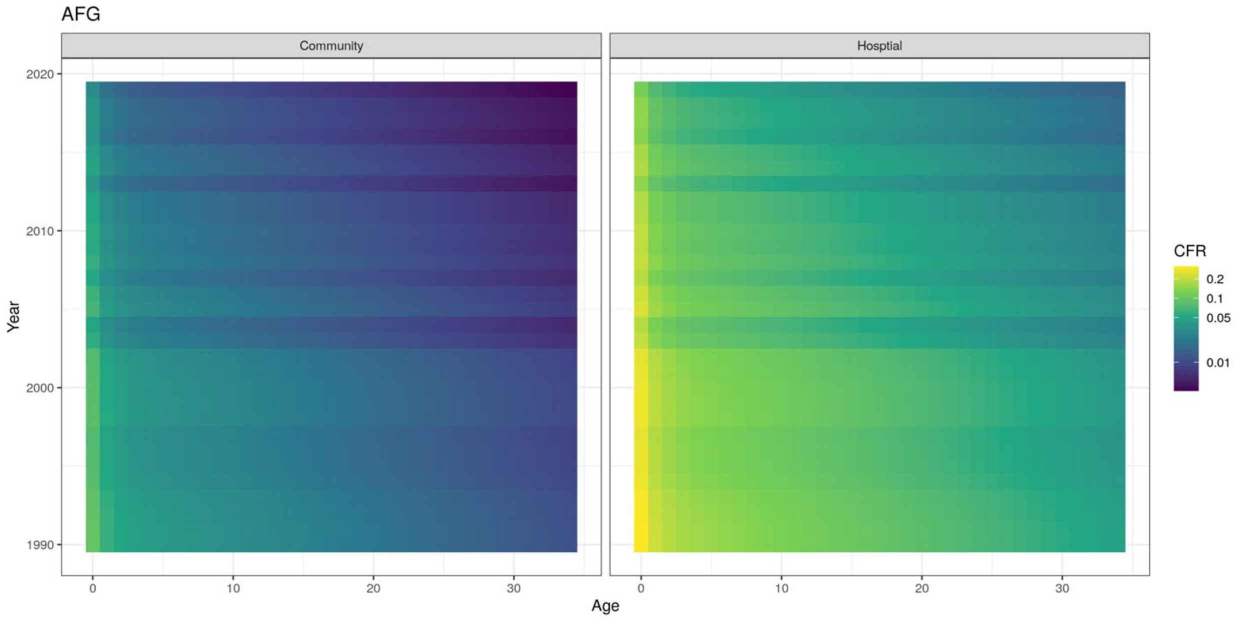
AFG
Community
Hosptial
2020
2010
2000
1990
Year
0
10
20
30
Age
CFR
0.2
0.1
0.05
0.01

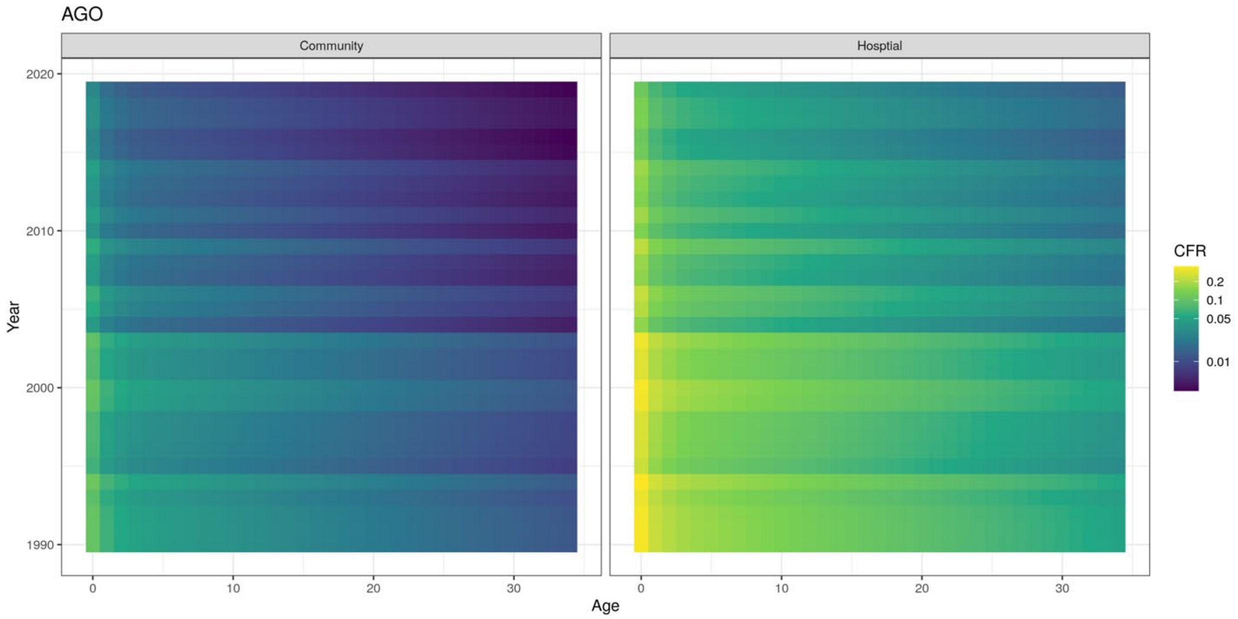
AGO
Community
Hosptial
Year
2020
2010
2000
1990
0
10
20
30
Age
CFR
0.2
0.1
0.05
0.01

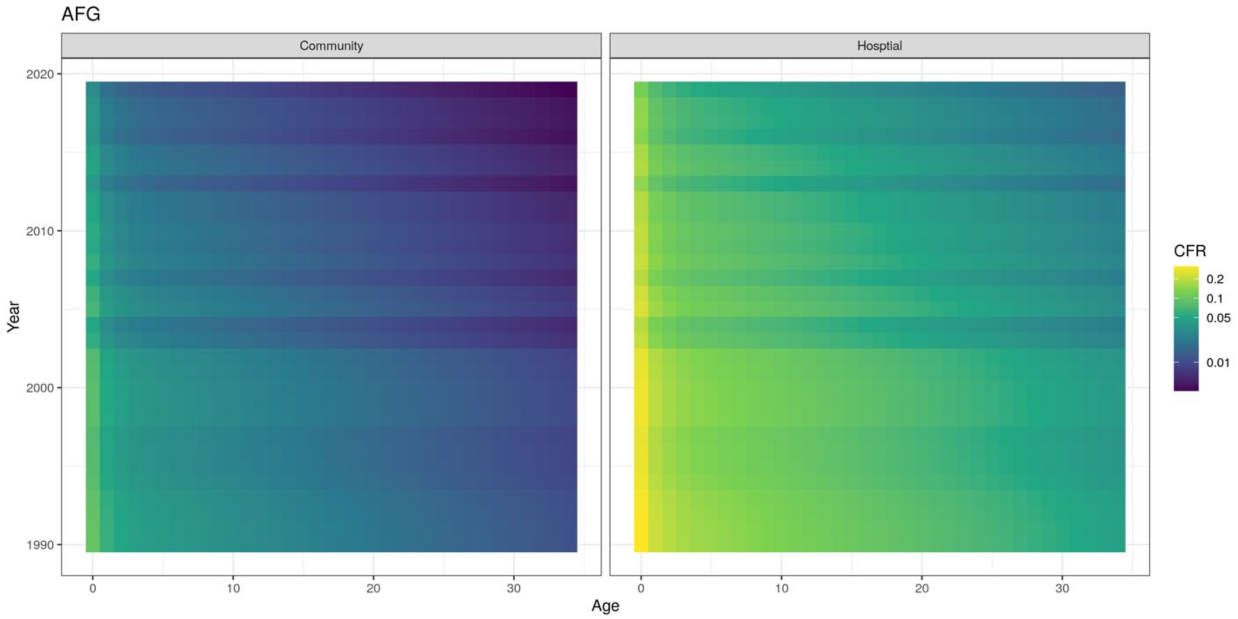
AFG
Community
Hosptial
Year
2020
2010
2000
1990
0
10
20
30
Age
CFR
0.2
0.1
0.05
0.01

Supplementary Figure 12. Distribution of CFR values for studies providing information on laboratory confirmation of cases (1) versus not providing information on laboratory confirmation of cases (0).

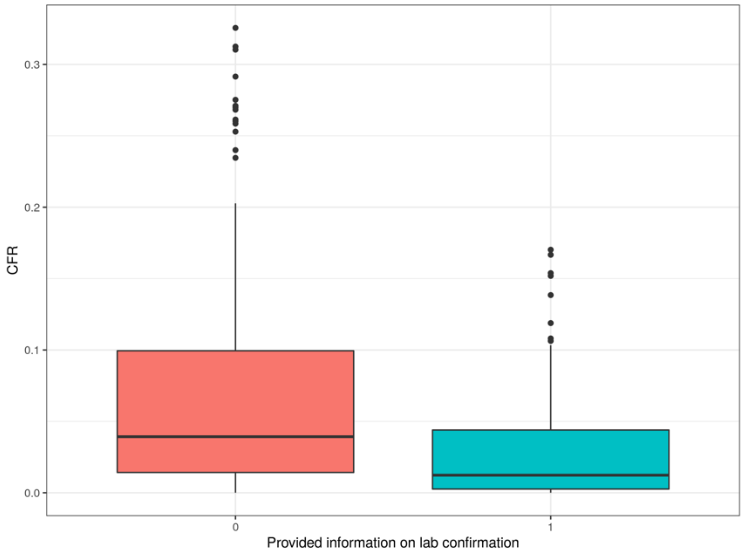

Supplementary Figure 13. Distribution measles incidence values used for covariates of country-years for studies providing information on laboratory confirmation of cases (1) versus not providing information on laboratory confirmation of cases (0).

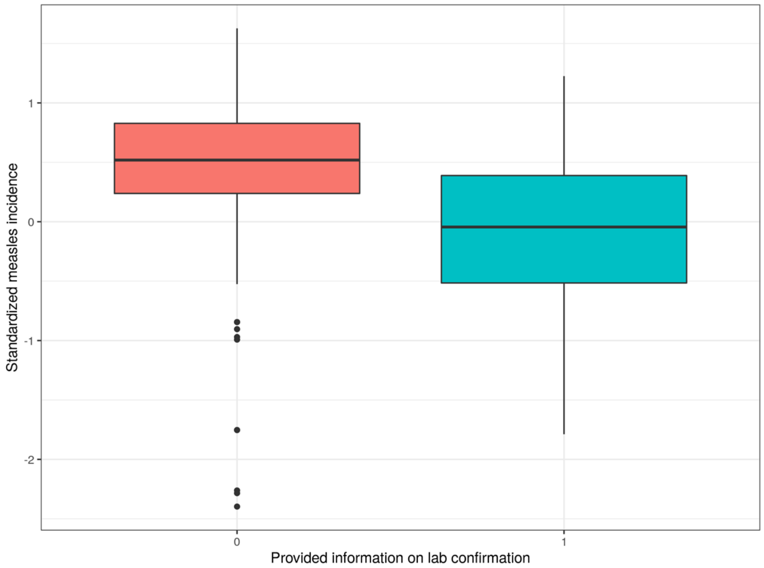

Supplementary Figure 14. Distribution MCV1 coverage values used for covariates of country-years for studies providing information on laboratory confirmation of cases (1) versus not providing information on laboratory confirmation of cases (0).

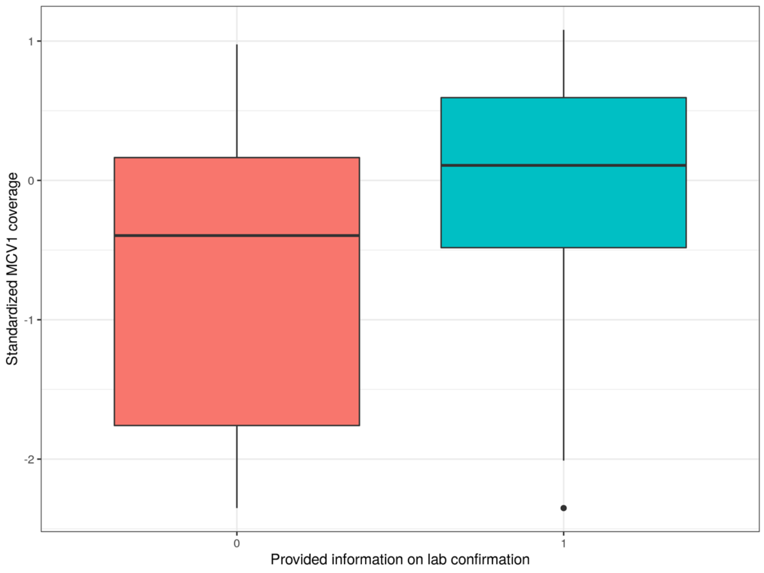

Supplementary Figure 15. CFR estimates from framework using all studies versus only studies providing information on laboratory confirmation of cases, for select years.

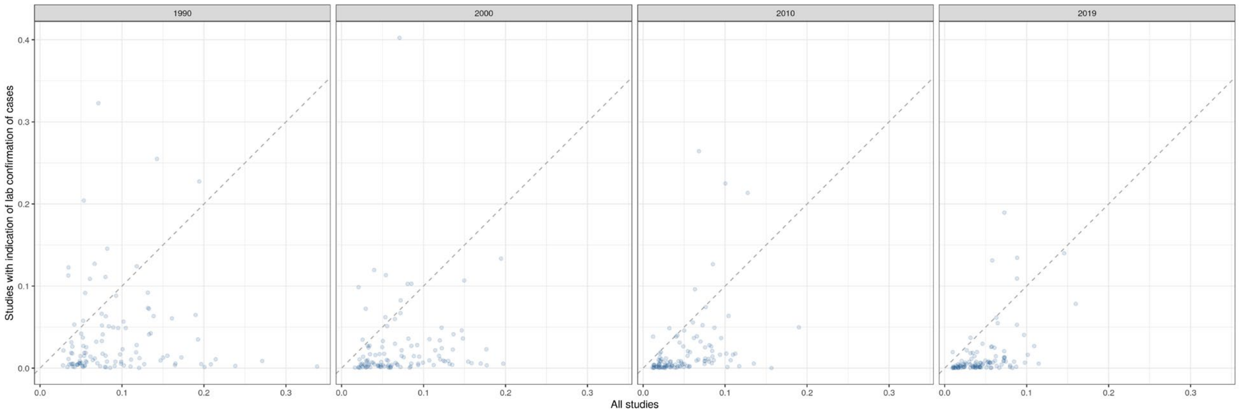

Supplementary Figure 16. CFR estimates from framework using all studies versus only studies providing definition of death attributable to measles, for select years.

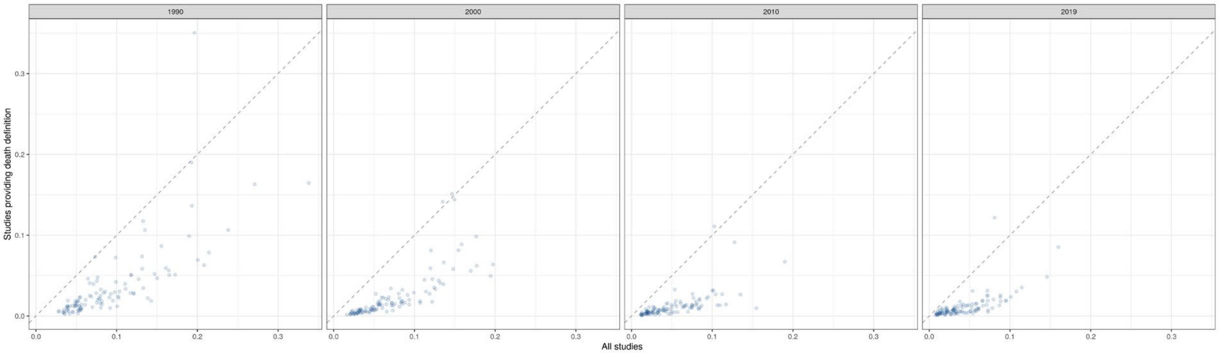
1990
2000
2010
2019
Studies providing death definition
All studies
0.0
0.1
0.2
0.3

References

1. Zheng P, Barber R, Sorensen RJ, Murray CJ. Trimmed constrained mixed effects models: formulations and algorithms. *Journal of Computational and Graphical Statistics* 2021; **30**(3): 1-13.

2. Sbarra AN, Jit M, Mosser JF, et al. Population-level risk factors related to measles case fatality: a conceptual framework and systematic review of evidence. *medRxiv* 2022.

3. cyipopt. 2022. https://cyipopt.readthedocs.io/en/stable/.

4. Portnoy A, Jit M, Ferrari M, Hanson M, Brenzel L, Verguet S. Estimates of case-fatality ratios of measles in low-income and middle-income countries: a systematic review and modelling analysis. *Lancet Glob Health* 2019; **7**(4): E472-E81.
